# Supplementary material for: Enantioselective radical conjugate additions driven by a photoactive intramolecular iminium-ion-based EDA complex
Source: Nat Commun. 2018 Aug 16;9:3274. doi: 10.1038/s41467-018-05375-2 (PMC6095928; doi:10.1038/s41467-018-05375-2)
Supplement: Supplementary file 1 — Supplementary Information [file 41467_2018_5375_MOESM1_ESM.pdf]

Supplementary Information for

Enantioselective Radical Conjugate Additions Driven by a Photoactive Intramolecular Iminium-Ion-Based EDA Complex

Zhong-Yan Cao,[1] Tamal Ghosh,[1] Paolo Melchiorre[1,2]*

1 – *ICIQ*, Institute of Chemical Research of Catalonia - the Barcelona Institute of Science and Technology, Avenida Països Catalans 16, 43007, Tarragona, Spain,

2 – *ICREA*, Catalan Institution for Research and Advanced Studies, Passeig Lluís Companys 23 – 08010, Barcelona, Spain.

*Correspondence to: pmelchiorre@iciq.es

Supplementary Figures

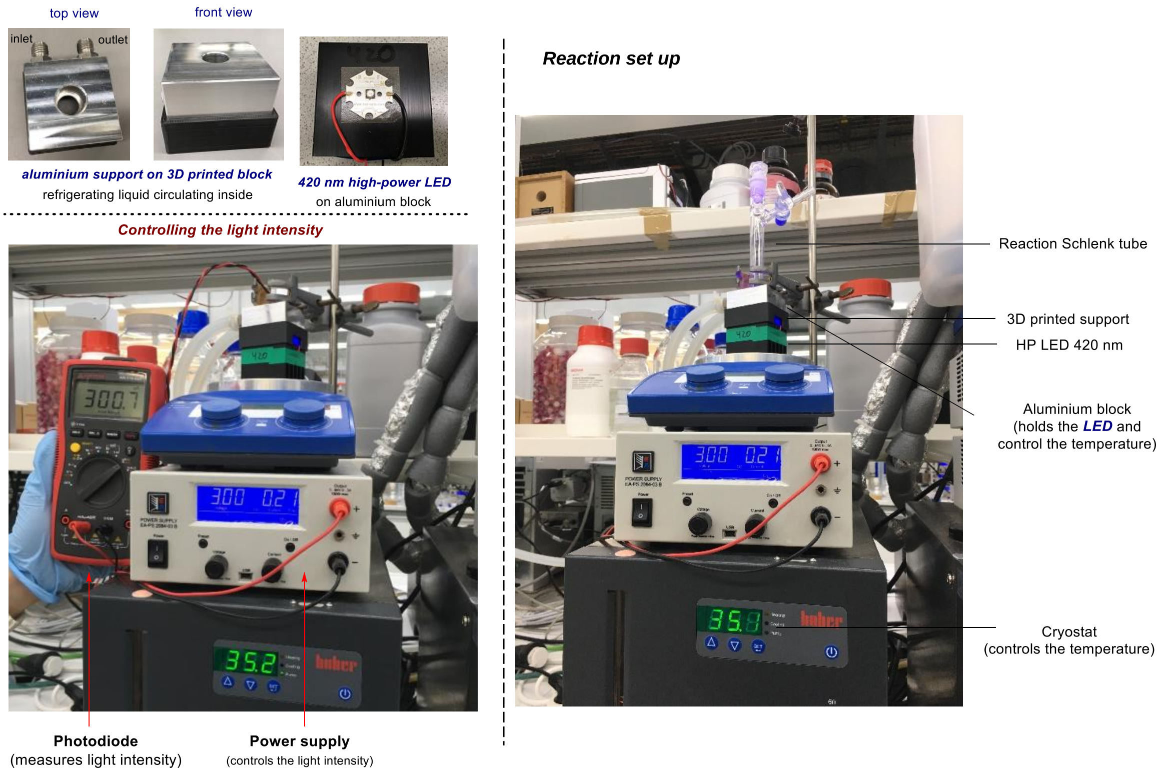

Supplementary Figure 1. Detailed set-up and illumination system. The light sources for illuminating the reaction vessel consisted in a 420 nm high-power single LED (OCU-440 UE420-X-T) purchased from OSA OPTO (For more information on the used LED, visit https://www.osa-opto.com/tl_files/osa_opto/inhalte/files/datasheets/ocl-440/440_UE420.pdf.

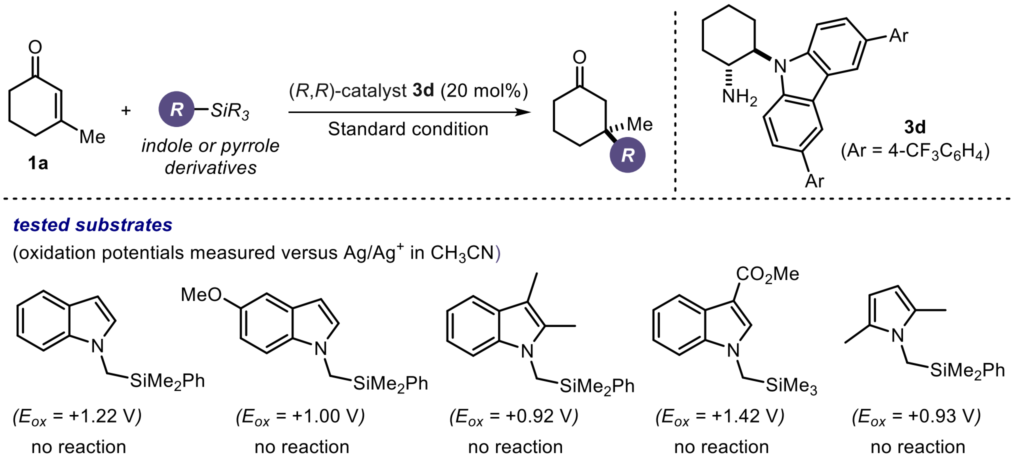

Supplementary Figure 2. Unsuccessful indole- or pyrrole-derived silanes tested in the photochemical reaction.

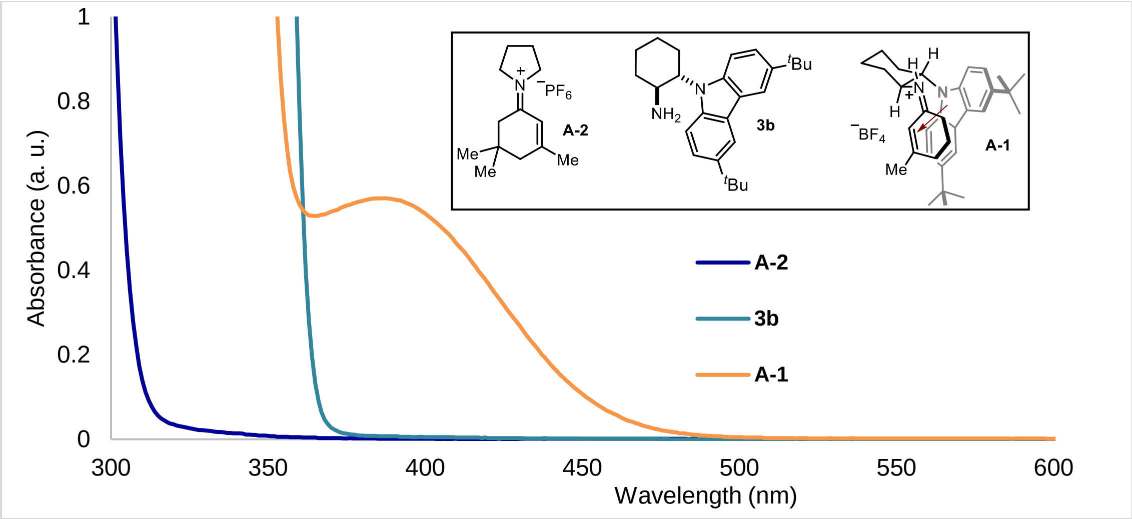

Supplementary Figure 3. UV-vis absorption spectra of iminium ion **A-2**, **A-1** and the carbazole catalyst **3b** (1.0 mM in CH_3CN). The preparation and full characterization of the iminium ion **A-1** was reported in previous literature.[1]

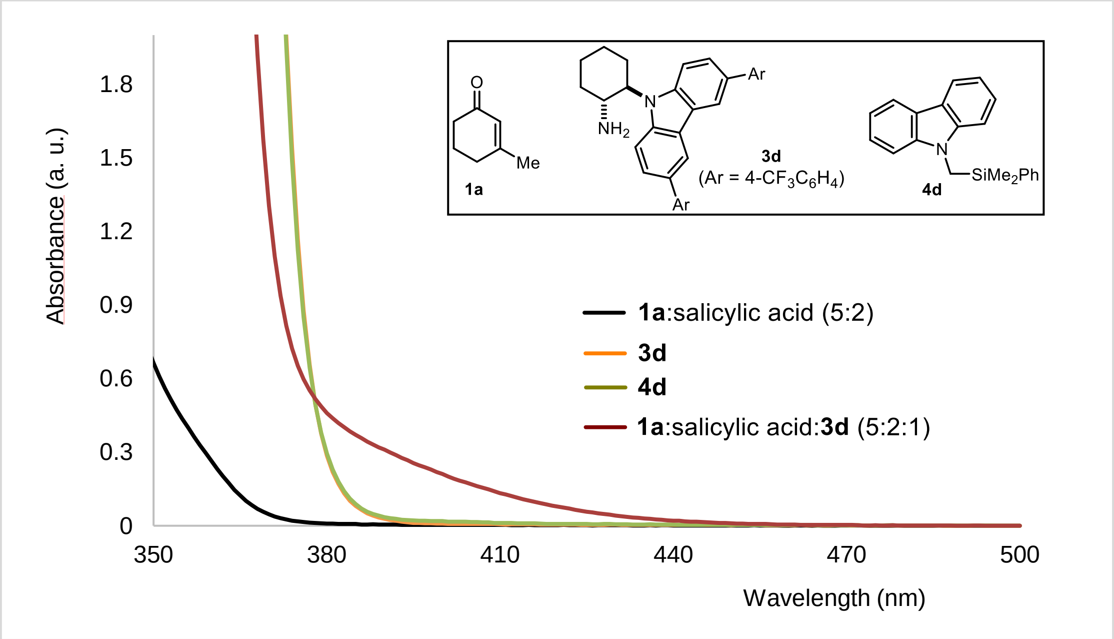

Supplementary Figure 4. UV-vis absorption spectra of the reaction mixture and the individual components, recorded in CH_3CN; [**1a**] = 0.5 M; [**3d**] = 0.1 M; [salicylic acid] = 0.2 M; [**4d**] = 0.75 M. None of the individual reaction components can absorb in the visible region. However, the *in situ* formed iminium ion, generated upon condensation of enone **1a** and the carbazole amine catalyst **3d** in the presence of salicylic acid, can absorb in the visible region (magenta line).

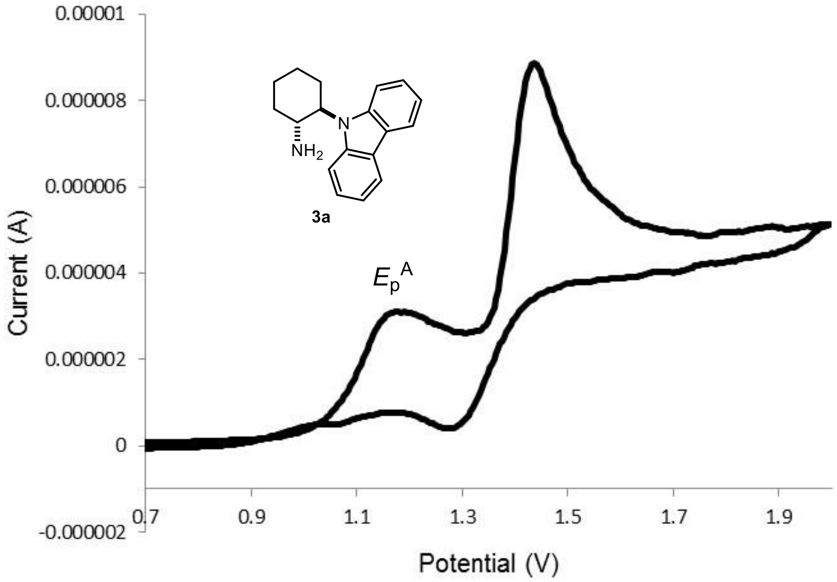

Supplementary Figure 5. Cyclic voltammogram of catalyst **3a** [0.001 M] in [0.1 M] $TBAPF_6$ in CH_3CN. Sweep rate: 10 mV/s. Pt electrode working electrode, Ag/AgCl (NaCl saturated) reference electrode, Pt wire auxiliary electrode. Irreversible oxidation peak, $E_p^A = E_{ox} = +1.15$ V; E_p^A is the anodic peak potential, which has been used to describe the electrochemical properties (E_{ox}) of the carbazole moiety within catalyst **3a**.

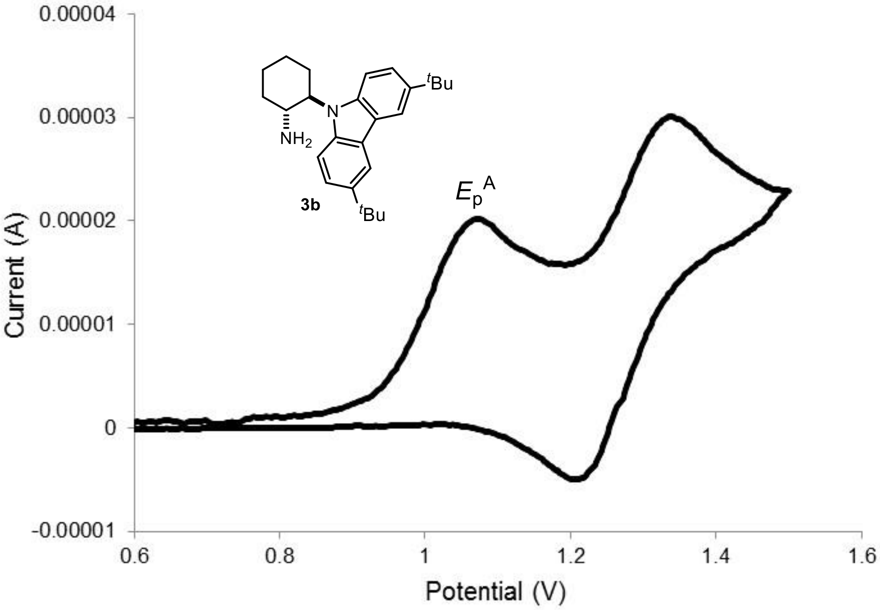

Supplementary Figure 6. Cyclic voltammogram of catalyst **3b** [0.001 M] in [0.1 M] $TBAPF_6$ in CH_3CN. Sweep rate: 10 mV/s. Pt electrode working electrode, Ag/AgCl (NaCl saturated) reference electrode, Pt wire auxiliary electrode. Irreversible oxidation peak, $E_p^A = E_{ox} = +1.05$ V; E_p^A is the anodic peak potential, which has been used to describe the electrochemical properties (E_{ox}) of the carbazole moiety within catalyst **3b**.

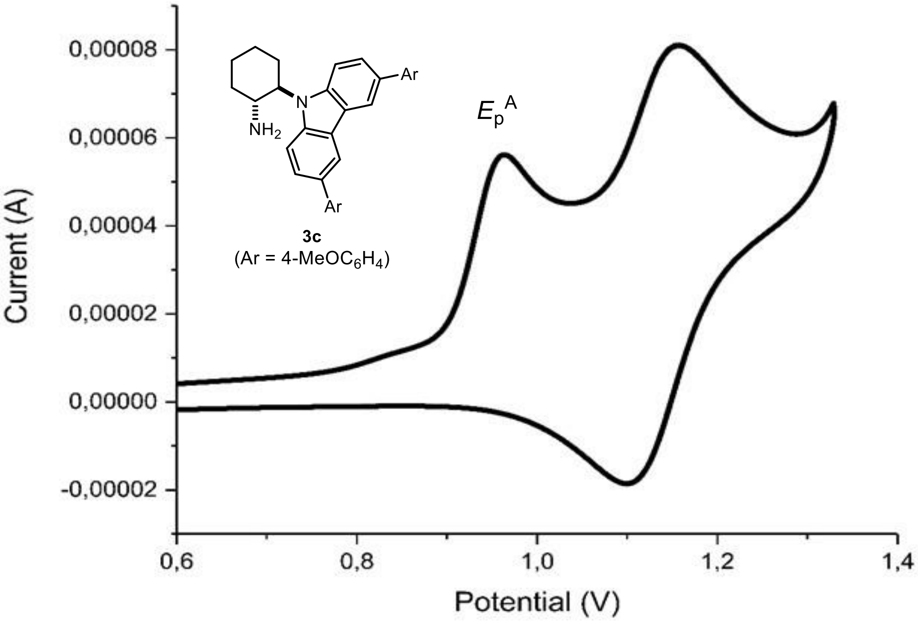

Supplementary Figure 7. Cyclic voltammogram of the catalyst **3c** [0.001 M] in [0.1 M] TBAPF$_6$ in CH_3CN. Sweep rate: 100 mV/s. Pt electrode working electrode, Ag/AgCl (NaCl saturated) reference electrode, Pt wire auxiliary electrode. Irreversible oxidation peak, $E_p^A = E_{ox} = +0.96$ V; E_p^A is the anodic peak potential, which has been used to describe the electrochemical properties (E_{ox}) of the carbazole moiety within catalyst **3c**.

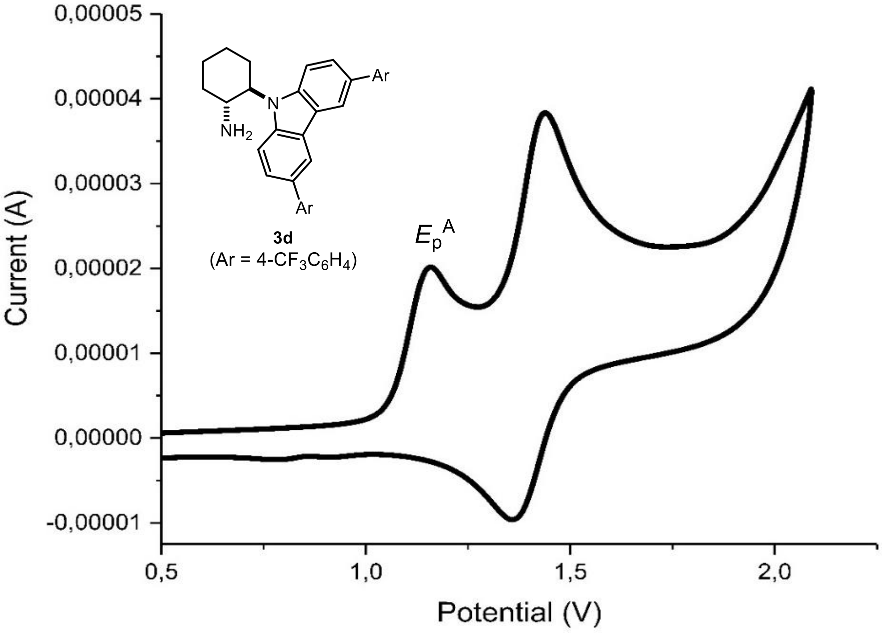

Supplementary Figure 8. Cyclic voltammogram of catalyst **3d** [0.001 M] in [0.1 M] TBAPF$_6$ in CH_3CN. Sweep rate: 100 mV/s. Pt electrode working electrode, Ag/AgCl (NaCl saturated) reference electrode, Pt wire auxiliary electrode. Irreversible oxidation peak, $E_p^A = E_{ox} = +1.16$ V; E_p^A is the anodic peak potential, which has been used to describe the electrochemical properties (E_{ox}) of the carbazole moiety within catalyst **3d**.

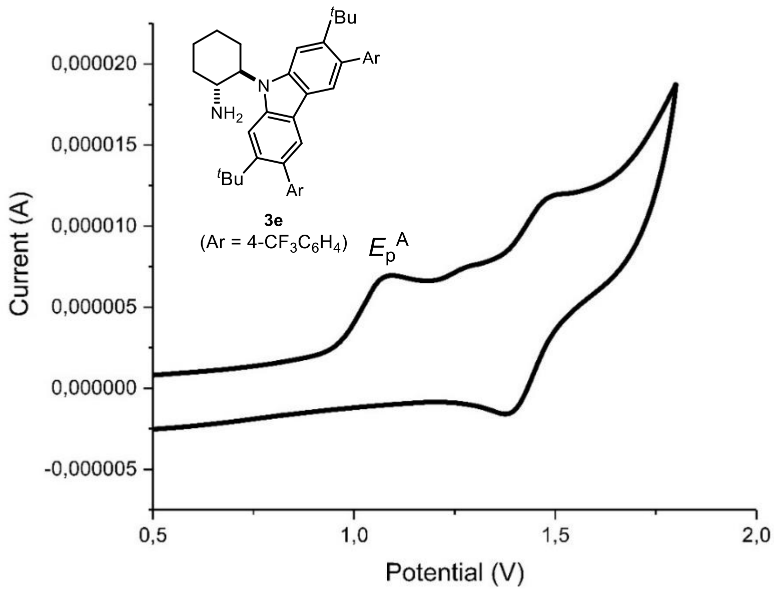

Supplementary Figure 9. Cyclic voltammogram of the catalyst **3e** [0.001 M] in [0.1 M] $TBAPF_6$ in CH_3CN. Sweep rate: 100 mV/s. Pt electrode working electrode, Ag/AgCl (NaCl saturated) reference electrode, Pt wire auxiliary electrode. Irreversible oxidation peak, $E_p^A = E_{ox} = +1.09$ V; E_p^A is the anodic peak potential, which has been used to describe the electrochemical properties (E_{ox}) of the carbazole moiety within catalyst **3e**.

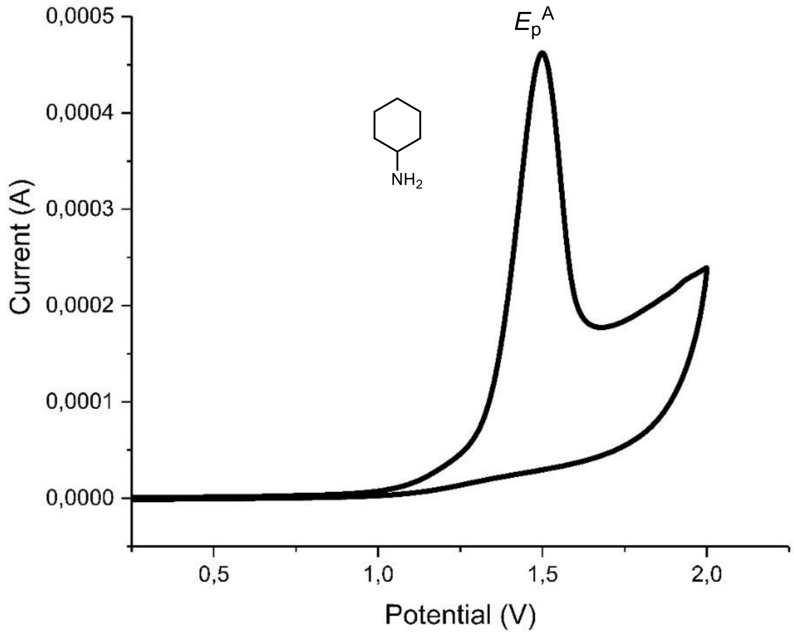

Supplementary Figure 10. Cyclic voltammogram of cyclohexylamine [0.001 M] in [0.1 M] $TBAPF_6$ in CH_3CN. Sweep rate: 100 mV/s. Pt electrode working electrode, Ag/AgCl (NaCl saturated) reference electrode, Pt wire auxiliary electrode. Irreversible oxidation peak, $E_p^A = E_{ox} = +1.49$ V; E_p^A is the anodic peak potential, which has been used to describe the electrochemical properties (E_{ox}) of cyclohexylamine. This indicates that the second oxidation event in the voltammogram of the carbazole catalysts **3** is ascribable to the primary amine oxidation (irreversible oxidation at about +1.5 V, see Supplementary Figures 5-9).

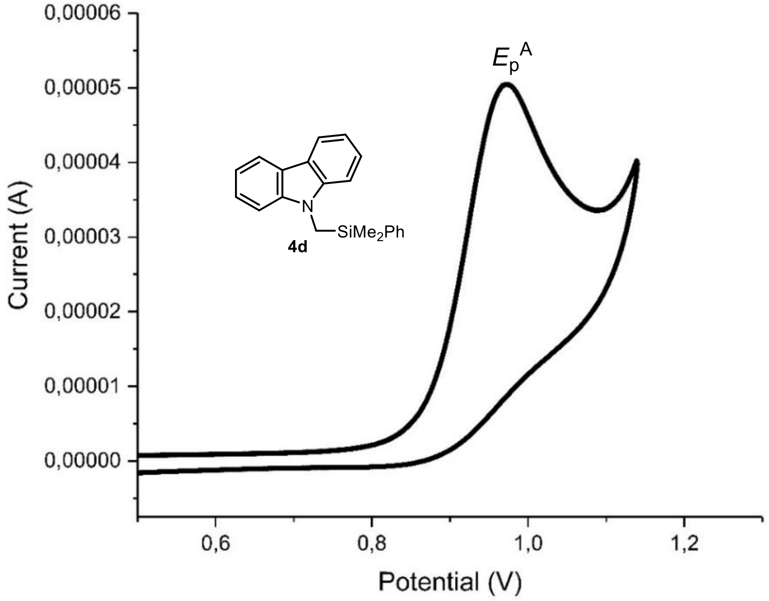

Supplementary Figure 11. Cyclic voltammogram of the α-carbazole substituted silane **4d** [0.001 M] in [0.1 M] $TBAPF_6$ in CH_3CN. Sweep rate: 100 mV/s. Pt electrode working electrode, Ag/AgCl (NaCl saturated) reference electrode, Pt wire auxiliary electrode. Irreversible oxidation peak, $E_p^A = E_{ox} = +0.97$ V; E_p^A is the anodic peak potential, while E_{ox} value describes the electrochemical properties of **4d**.

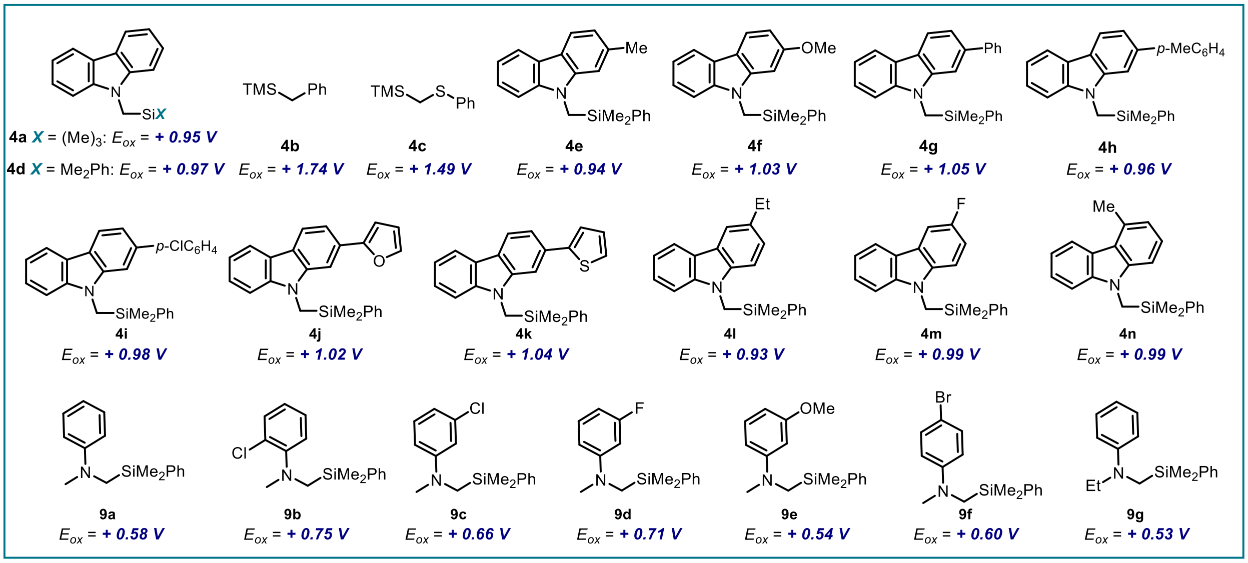

Supplementary Figure 12. Oxidation potentials of the organic silanes used in this study. All the oxidation potentials are given as E_{ox} *vs* Ag/Ag^+ in CH_3CN and they have been measured by cyclic voltammetry, following the same method described in Supplementary Figure 11.

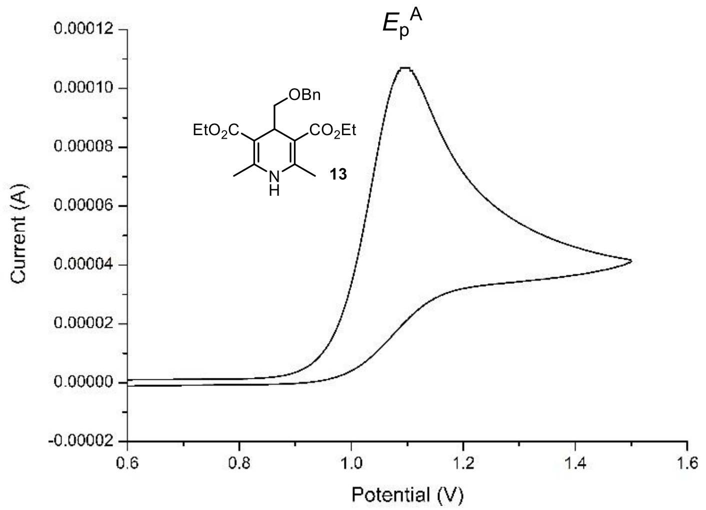

Supplementary Figure 13. Cyclic voltammogram of **13** [0.001 M] in [0.1 M] $TBAPF_6$ in CH_3CN. Sweep rate: 100 mV/s. Pt electrode working electrode, Ag/AgCl (NaCl saturated) reference electrode, Pt wire auxiliary electrode. Irreversible oxidation peak, $E_p^A = E_{ox} = +1.08$ V; E_p^A is the anodic peak potential, while E_{ox} value describes the electrochemical properties of **13**.

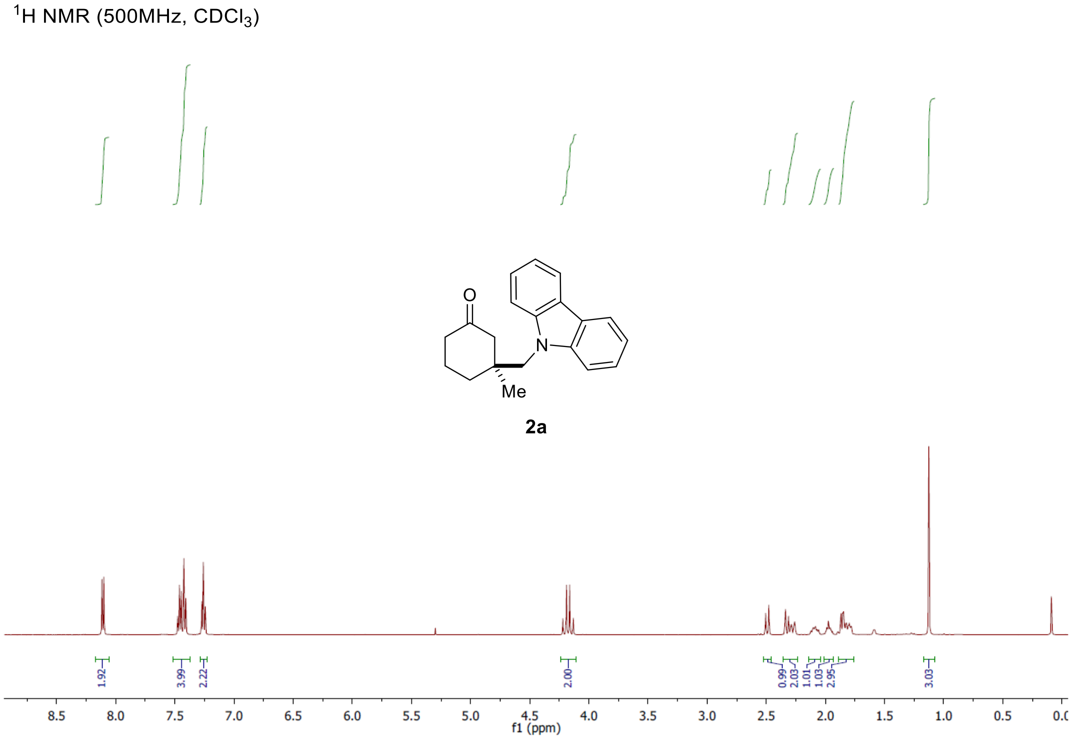

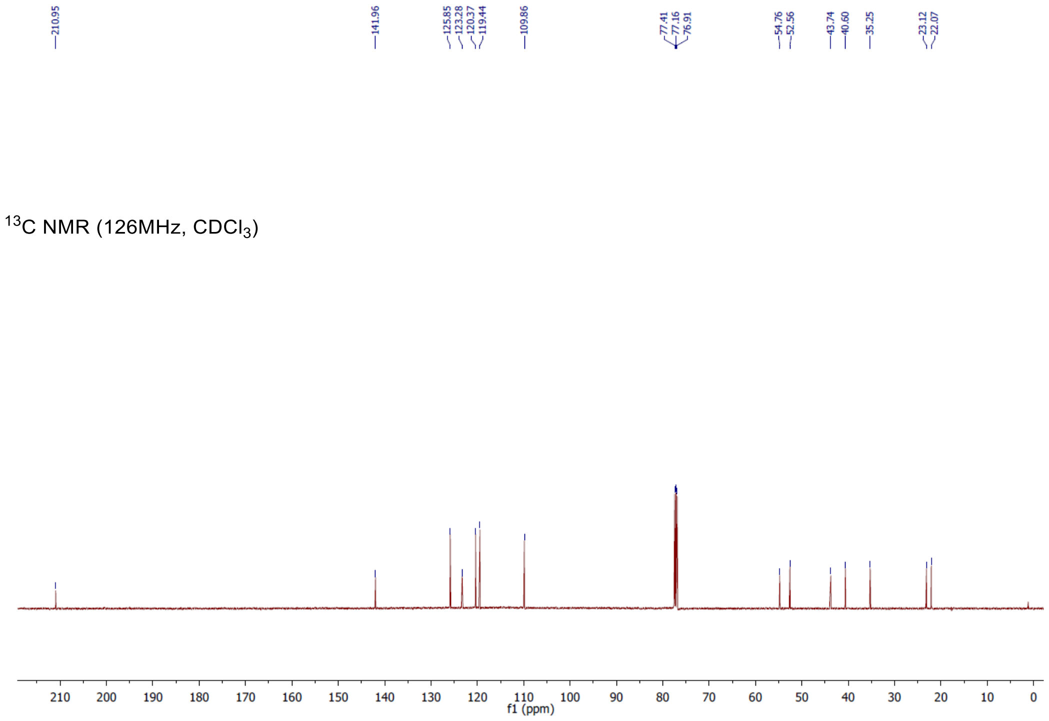

Supplementary Figure 14. ^{1}H and ^{13}C NMR spectra for compound **2a**

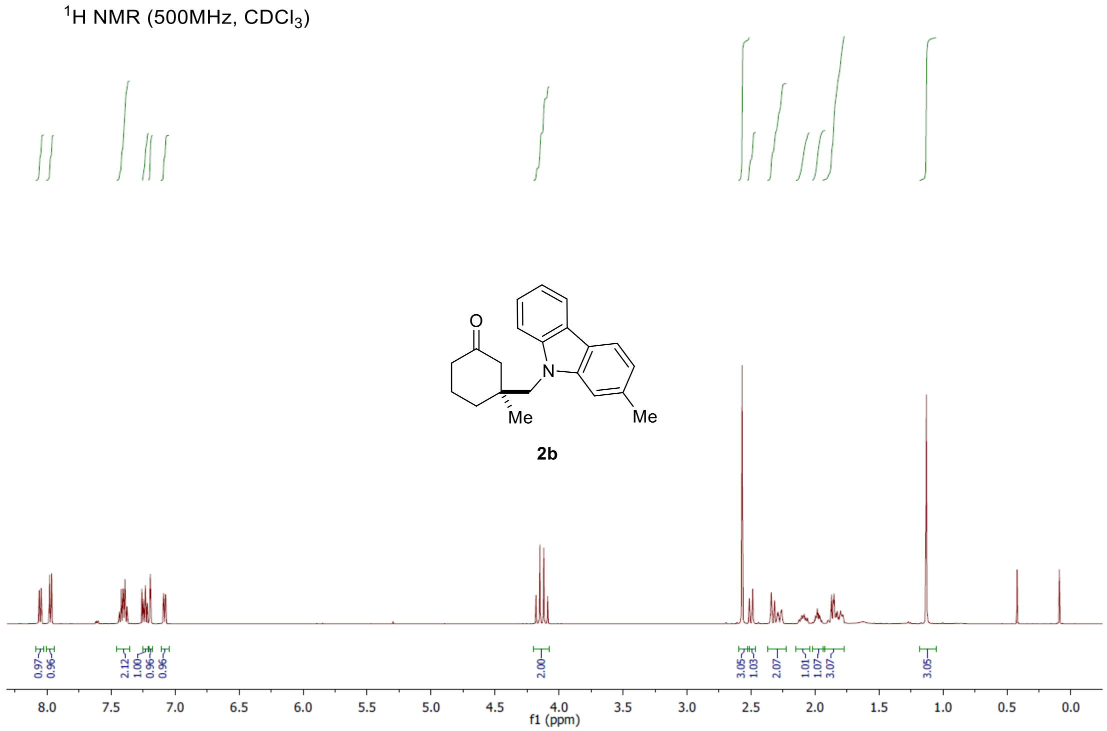

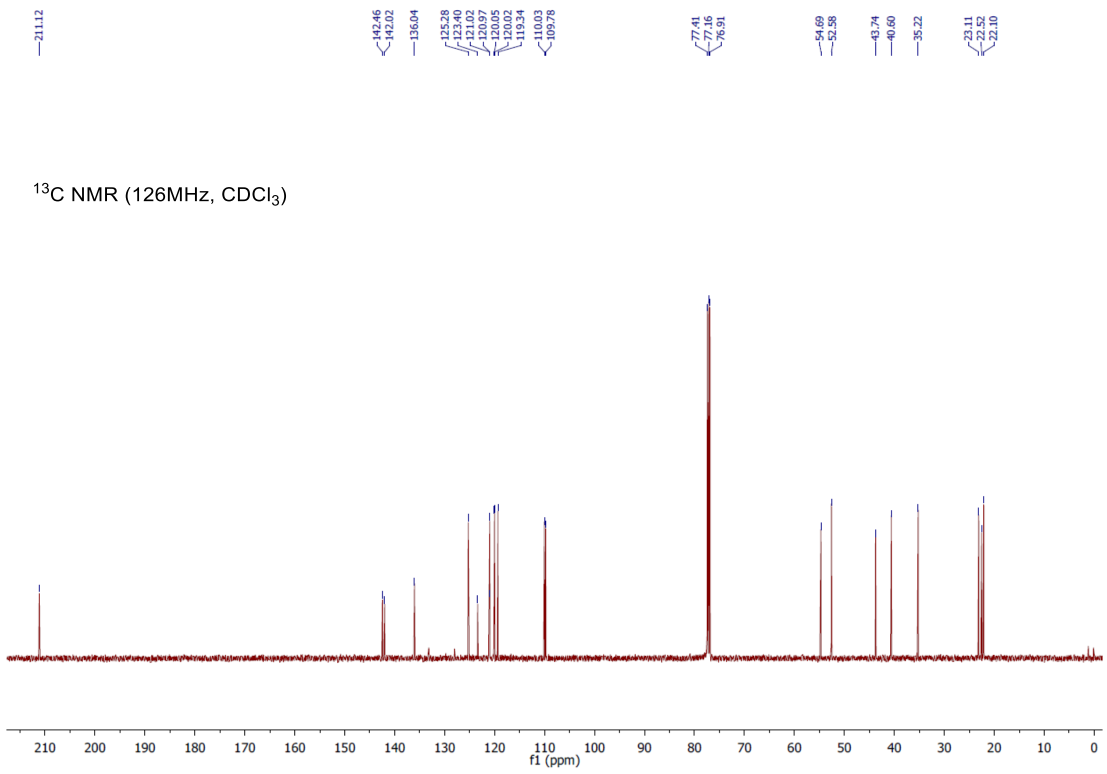

Supplementary Figure 15. ^{1}H and ^{13}C NMR spectra for compound **2b**

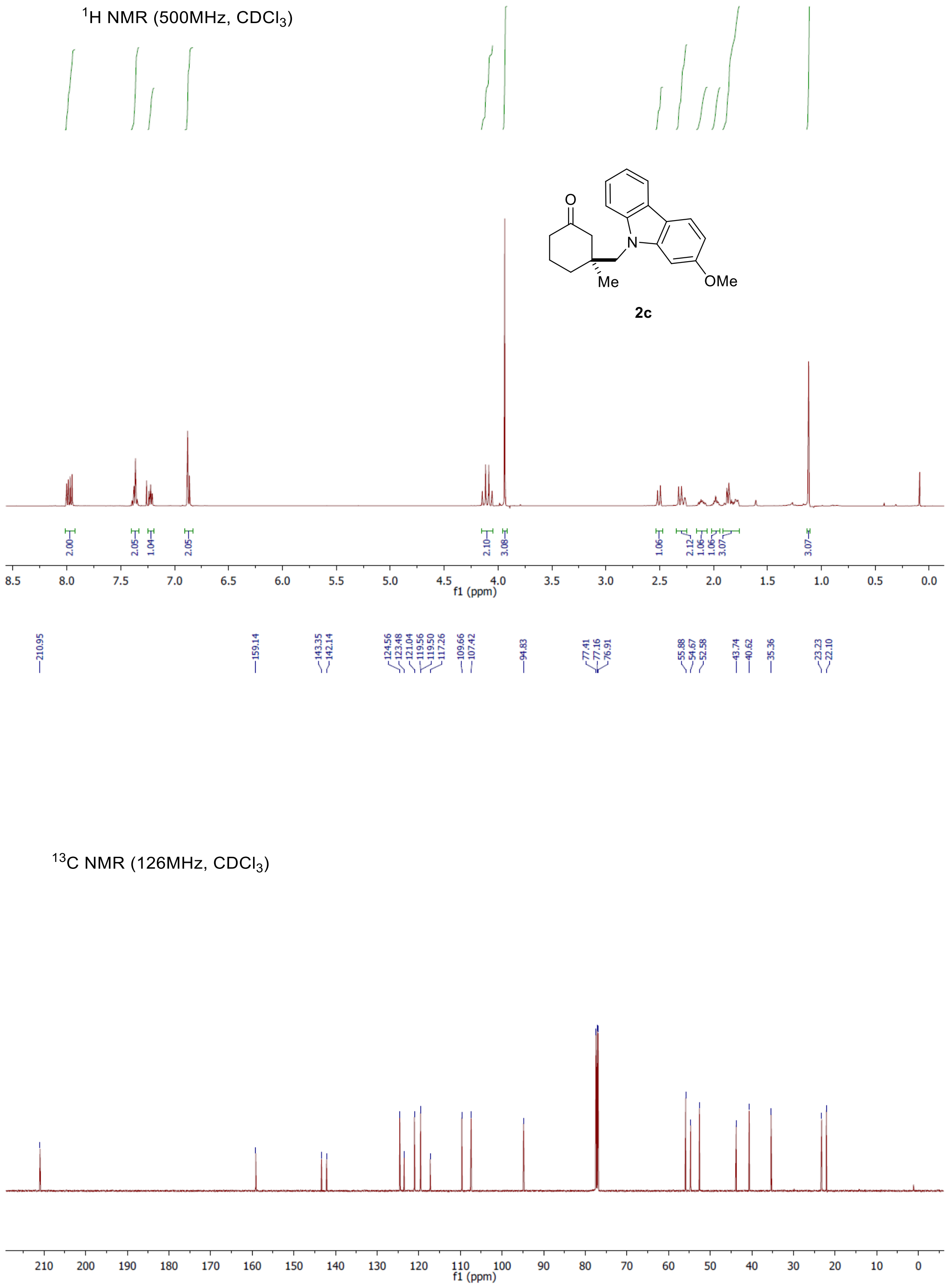

Supplementary Figure 16. ^{1}H and ^{13}C NMR spectra for compound **2c**

^{1}H NMR (500MHz, $CDCl_3$)

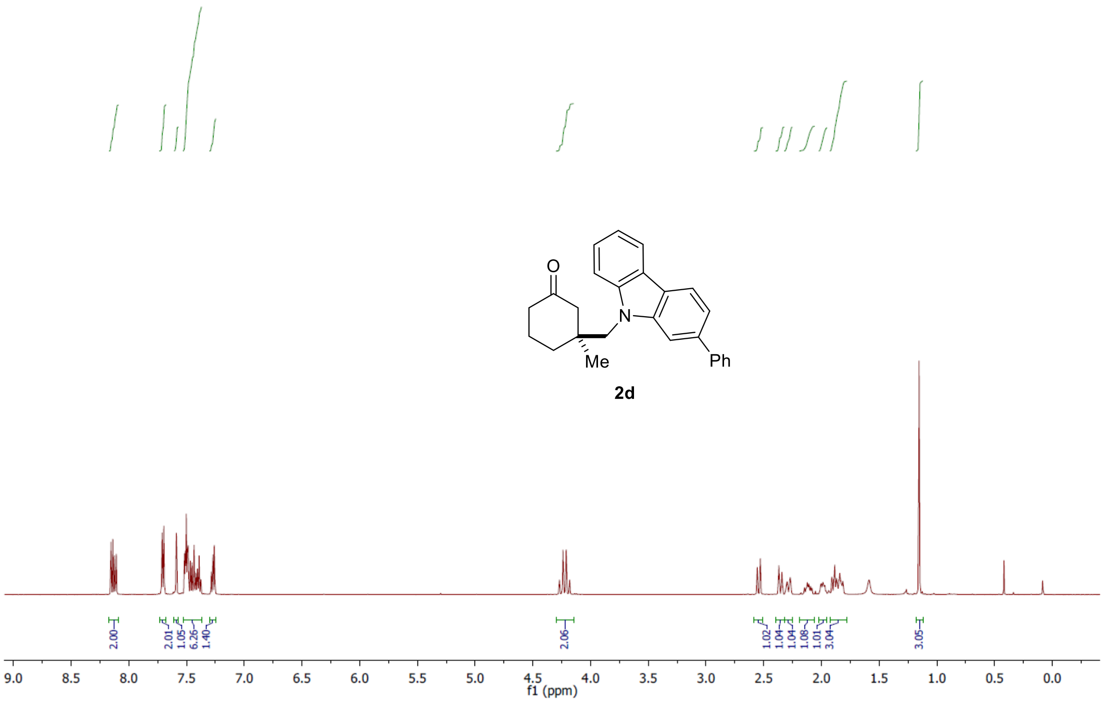

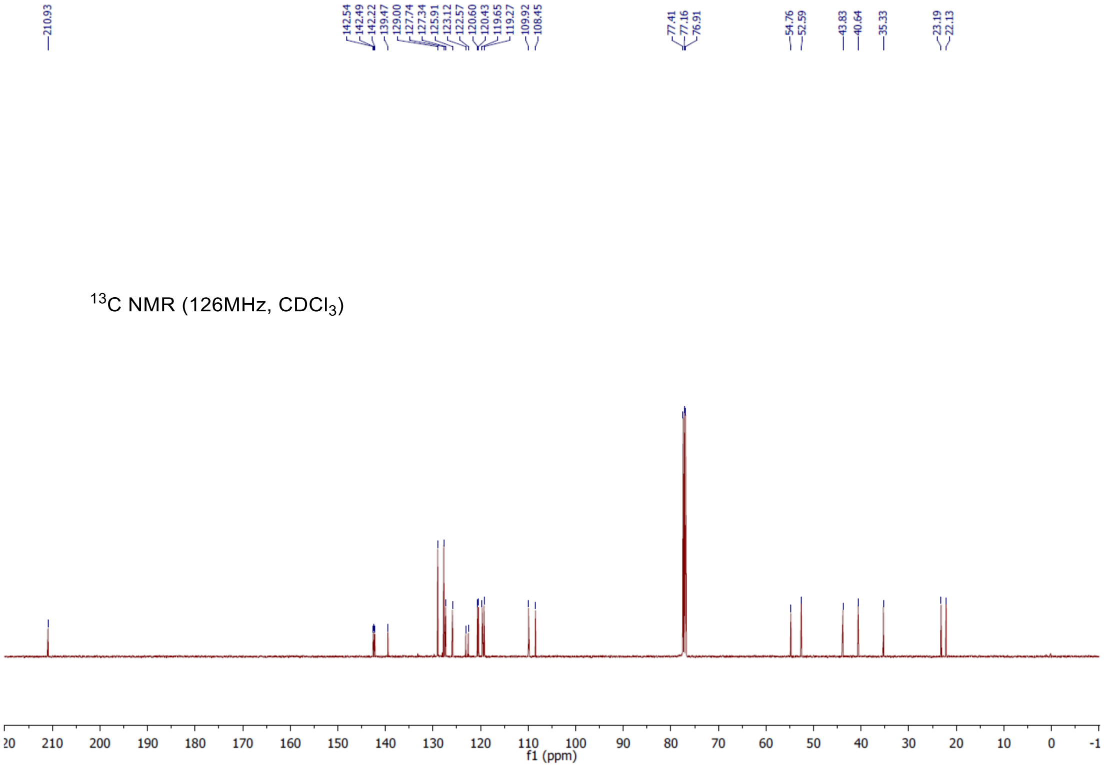

Supplementary Figure 17. ^{1}H and ^{13}C NMR spectra for compound **2d**

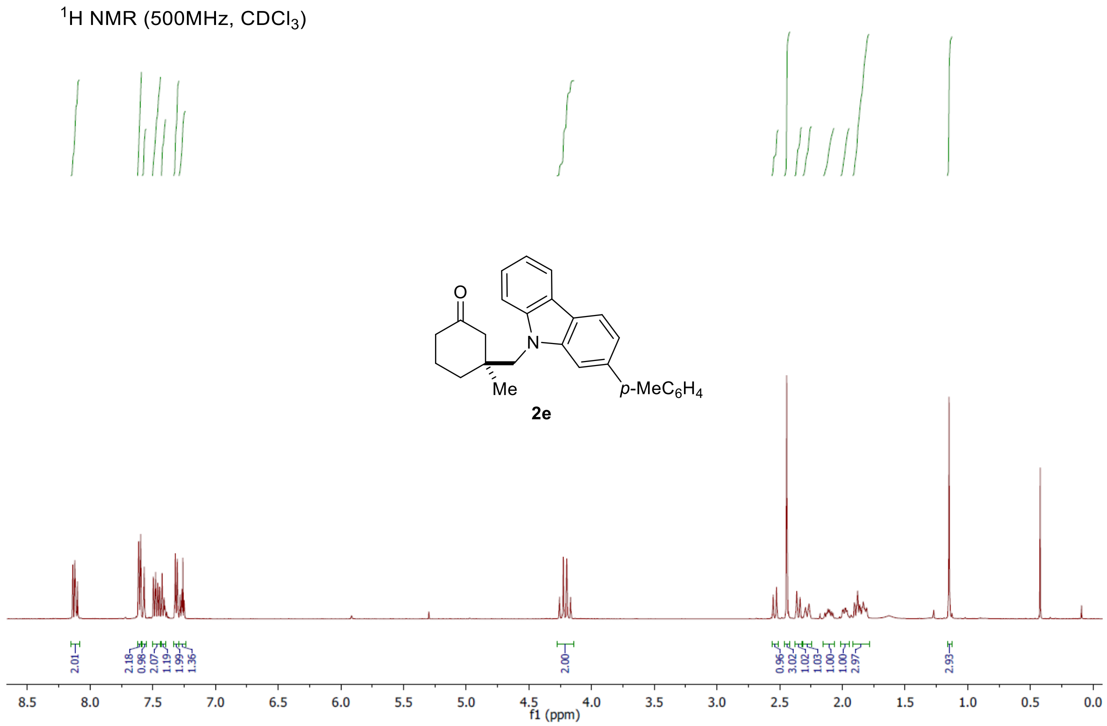

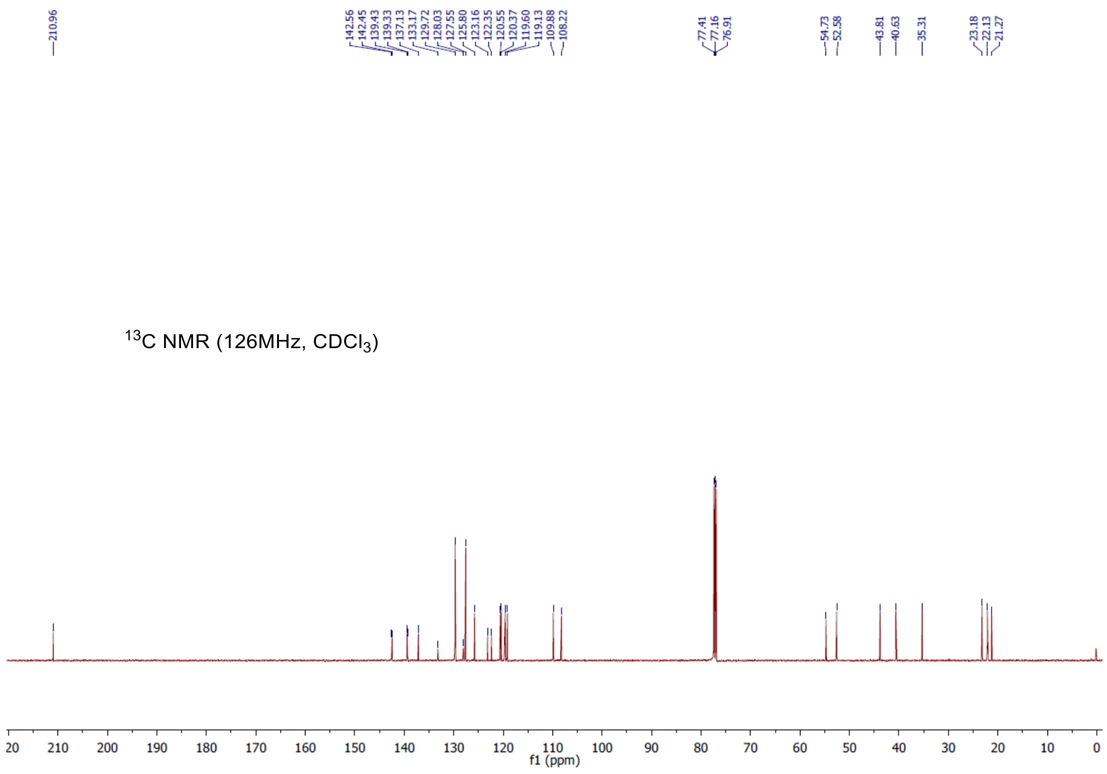

Supplementary Figure 18. ^{1}H and ^{13}C NMR spectra for compound **2e**

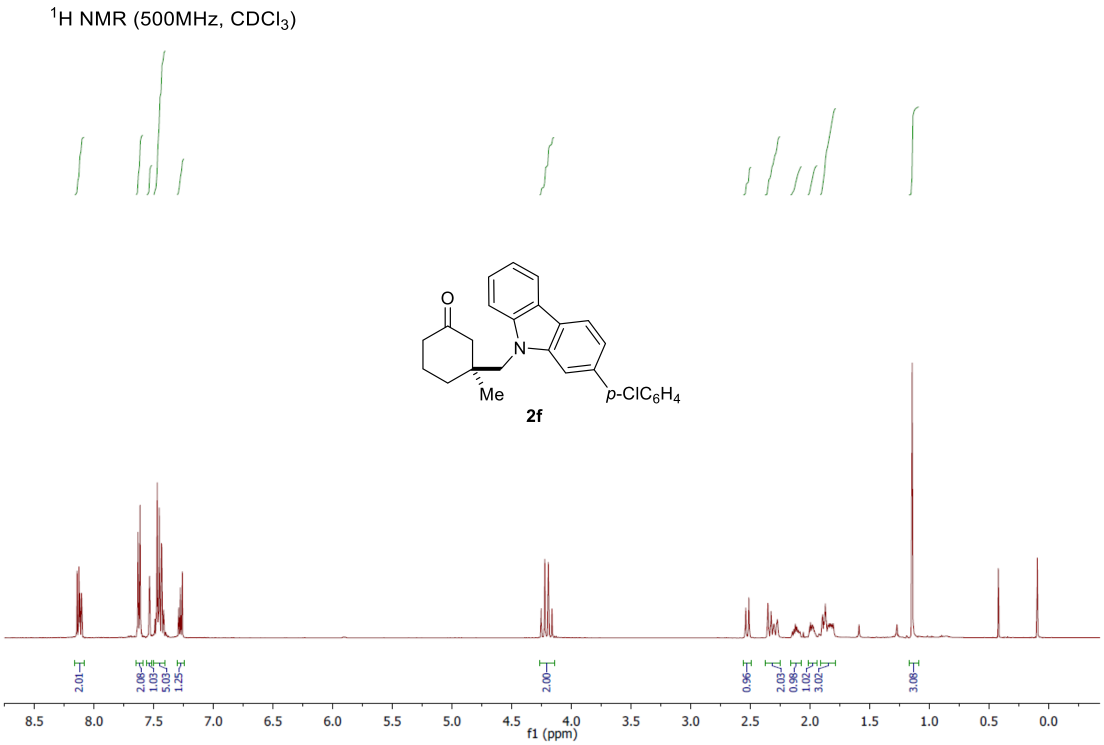

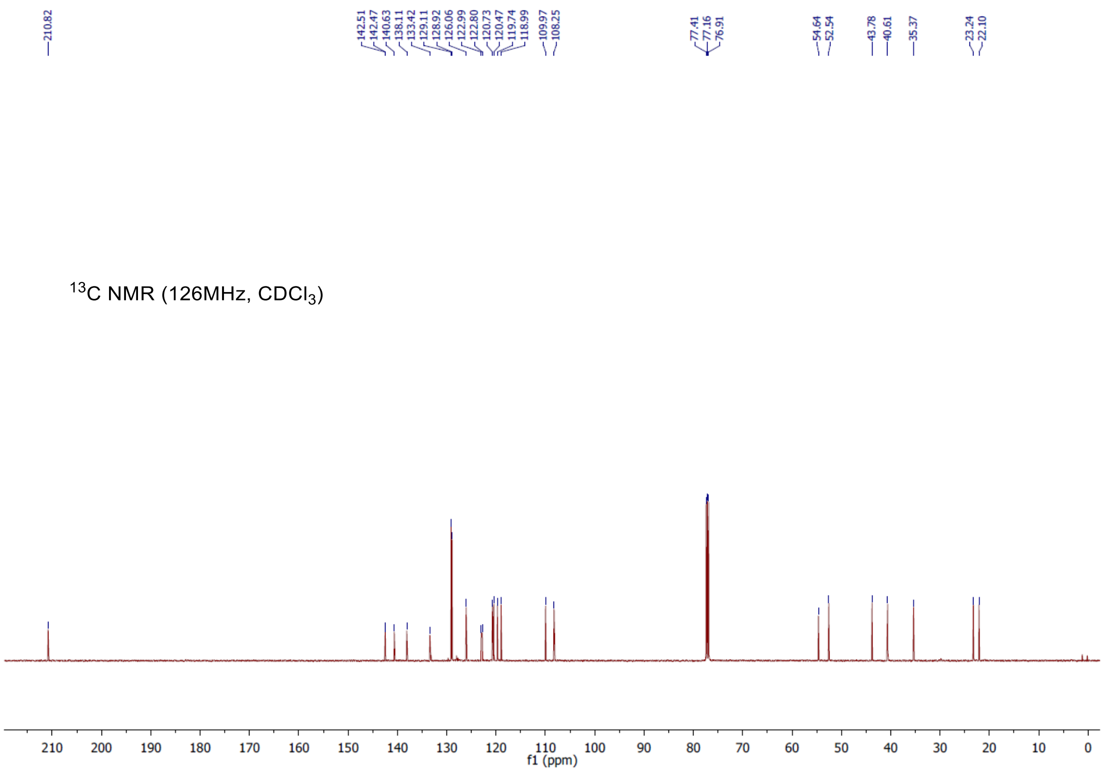

Supplementary Figure 19. ^{1}H and ^{13}C NMR spectra for compound **2f**

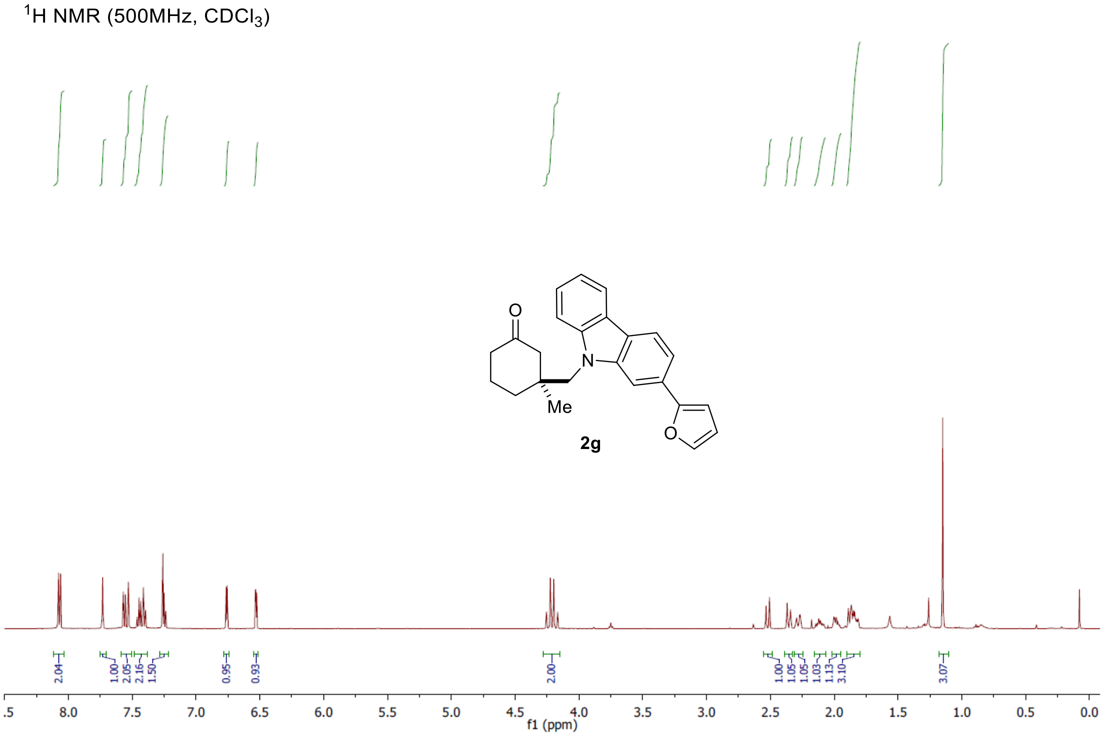

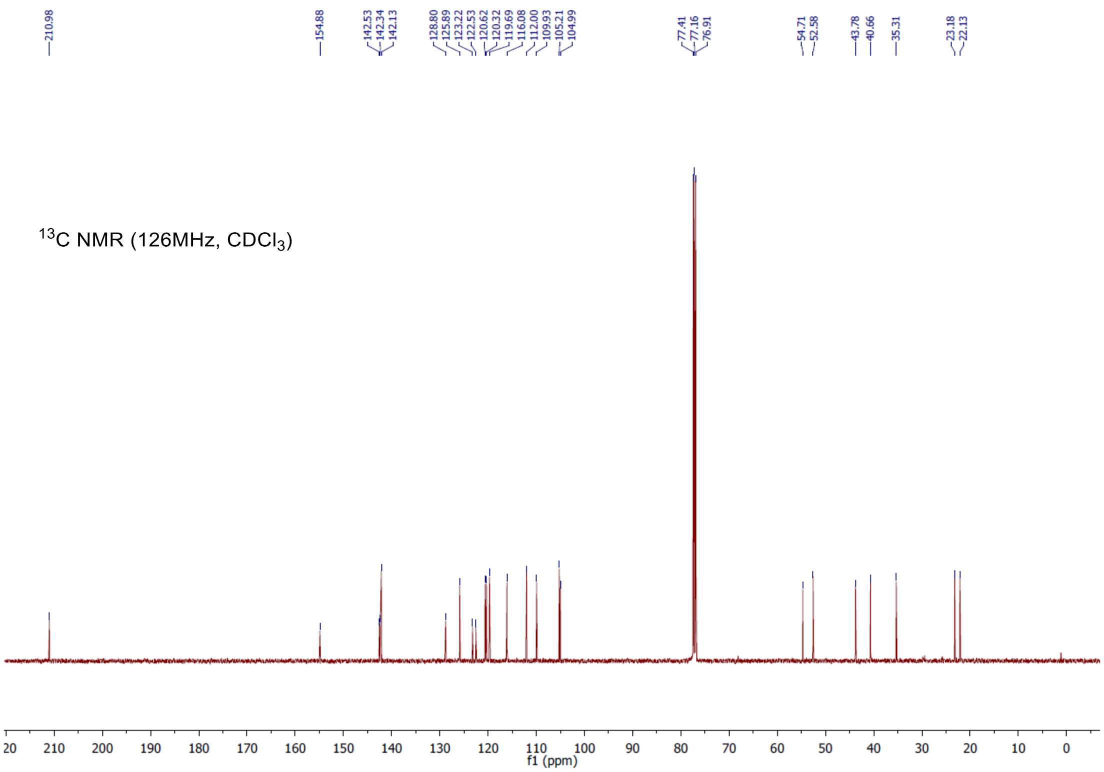

Supplementary Figure 20. ^{1}H and ^{13}C NMR spectra for compound **2g**

^{1}H NMR (500MHz, $CDCl_3$)

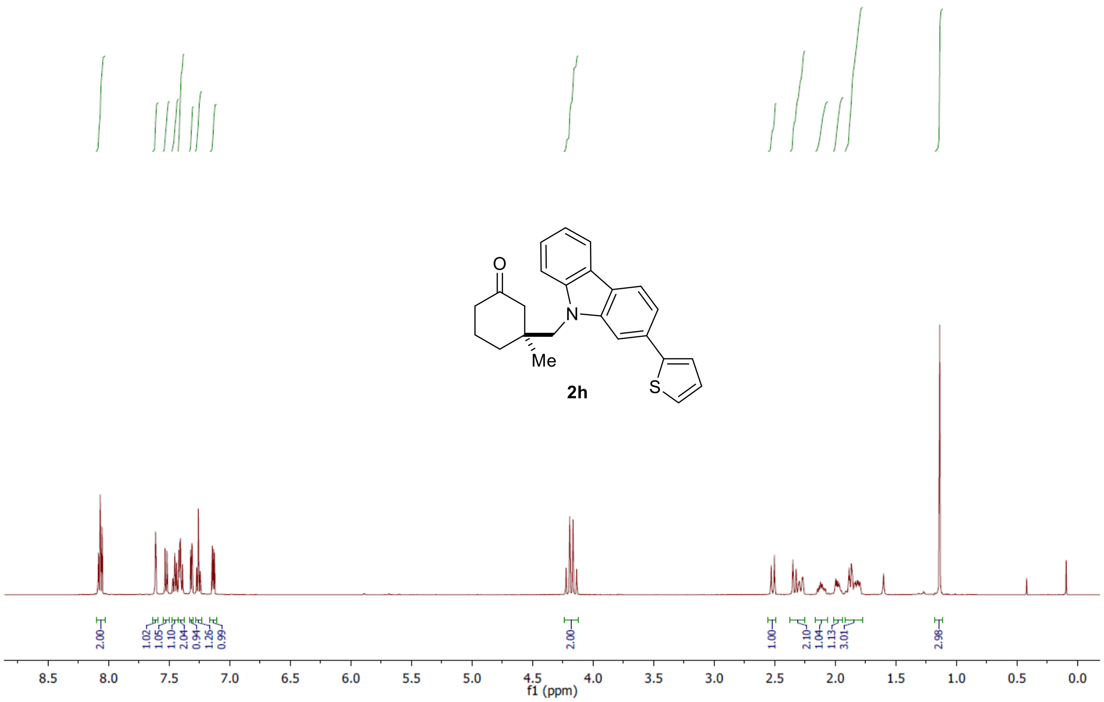

^{13}C NMR (126MHz, $CDCl_3$)

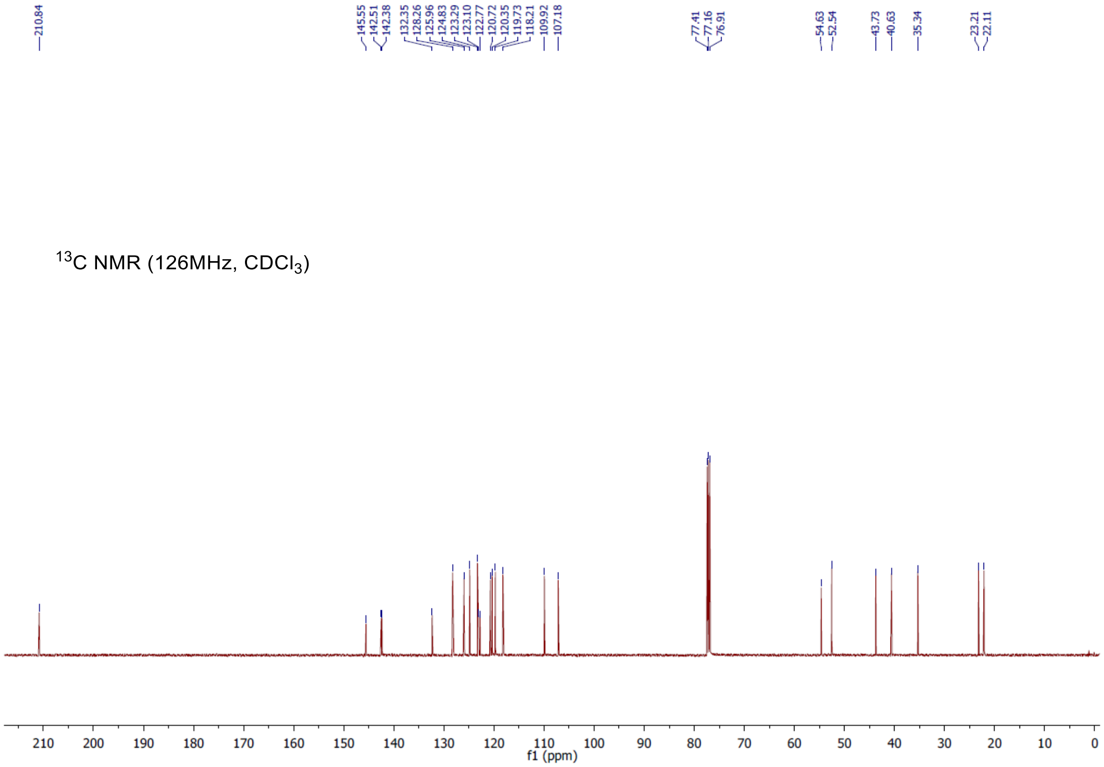

Supplementary Figure 21. ^{1}H and ^{13}C NMR spectra for compound **2h**

^{1}H NMR (500MHz, $CDCl_3$)

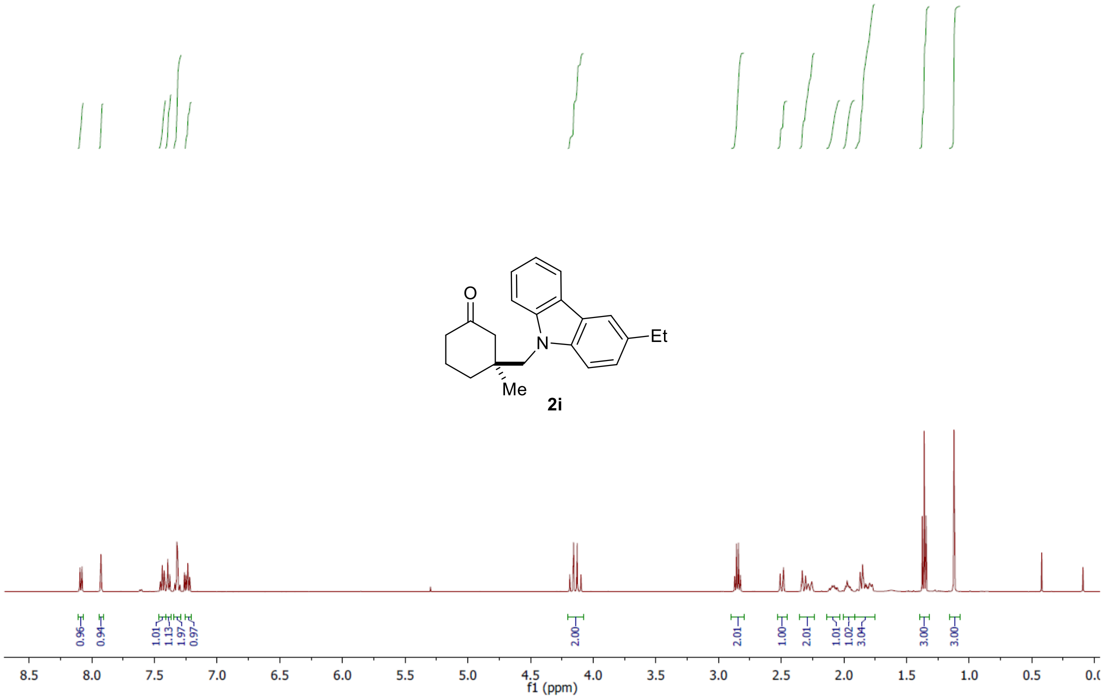

^{13}C NMR (126MHz, $CDCl_3$)

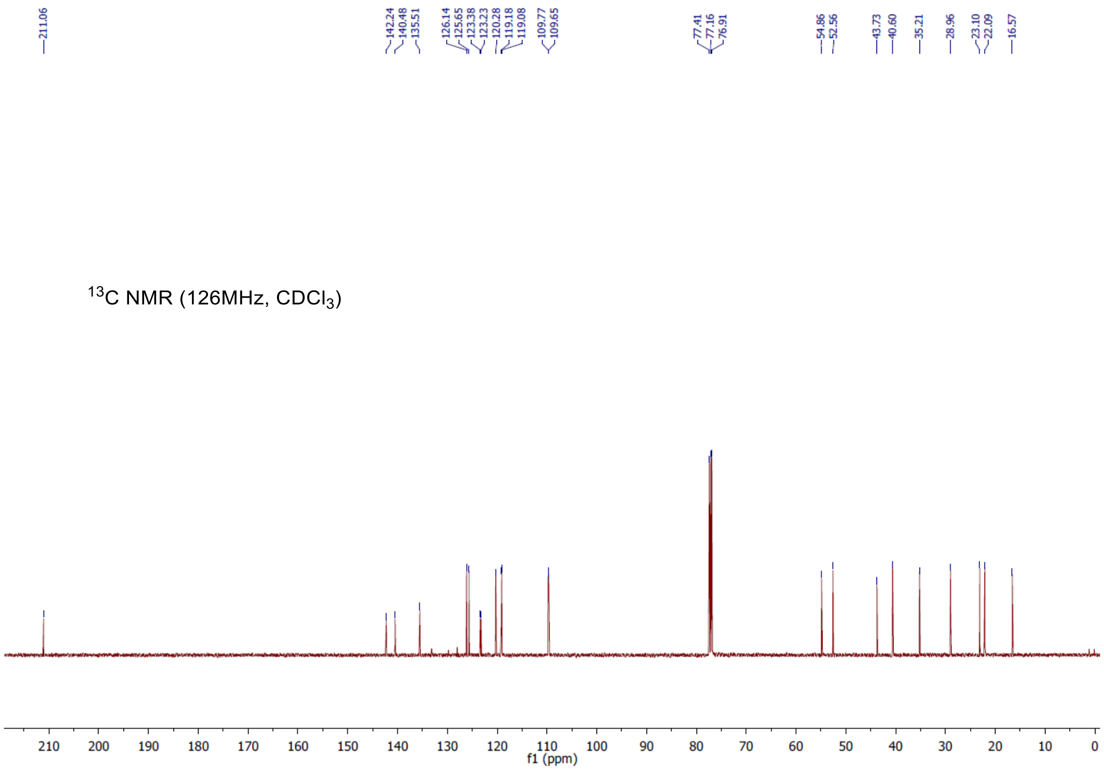

Supplementary Figure 22. ^{1}H and ^{13}C NMR spectra for compound **2i**

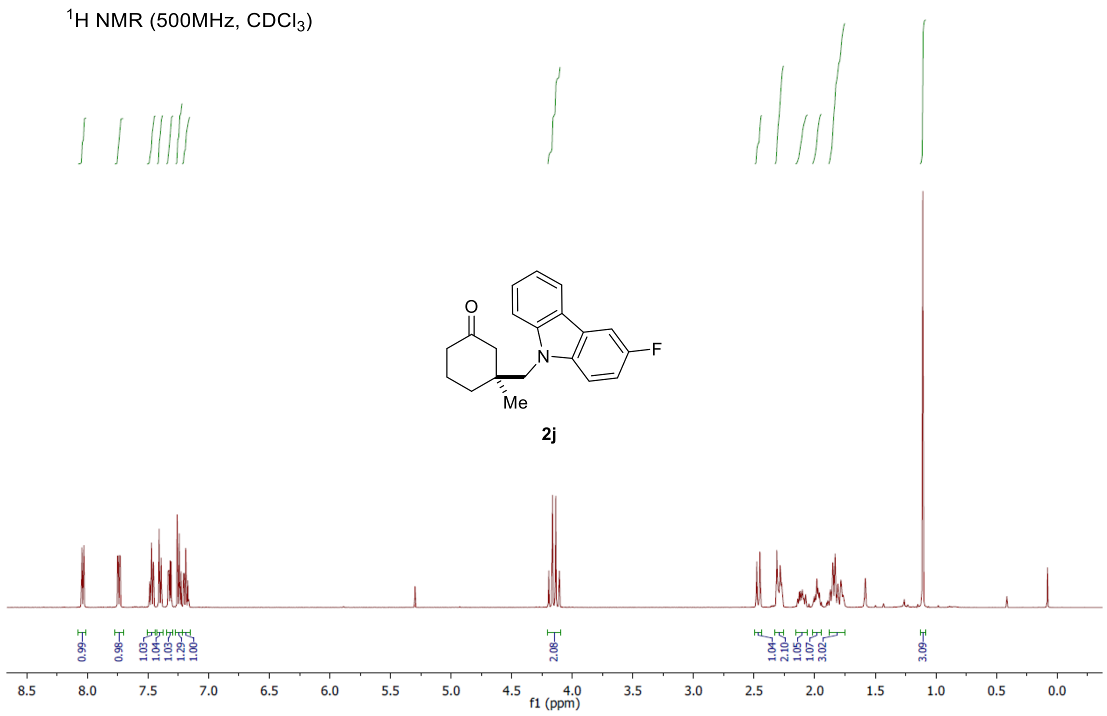

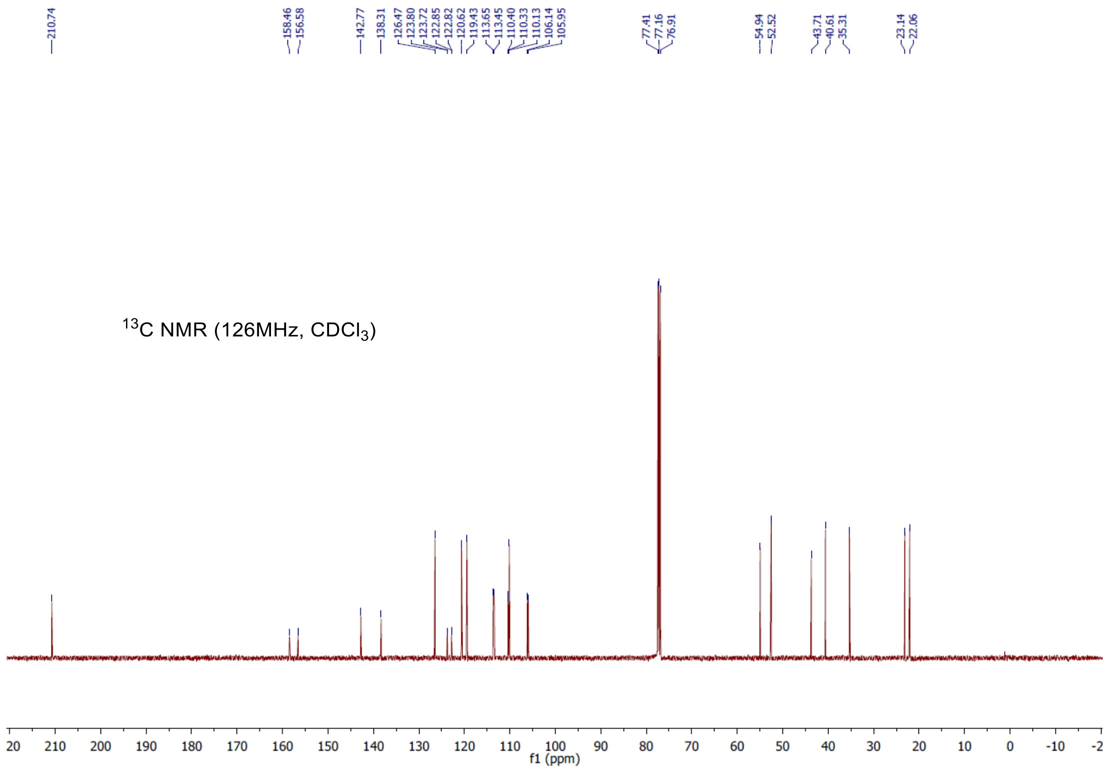

Supplementary Figure 23. ^{1}H and ^{13}C NMR spectra for compound **2j**

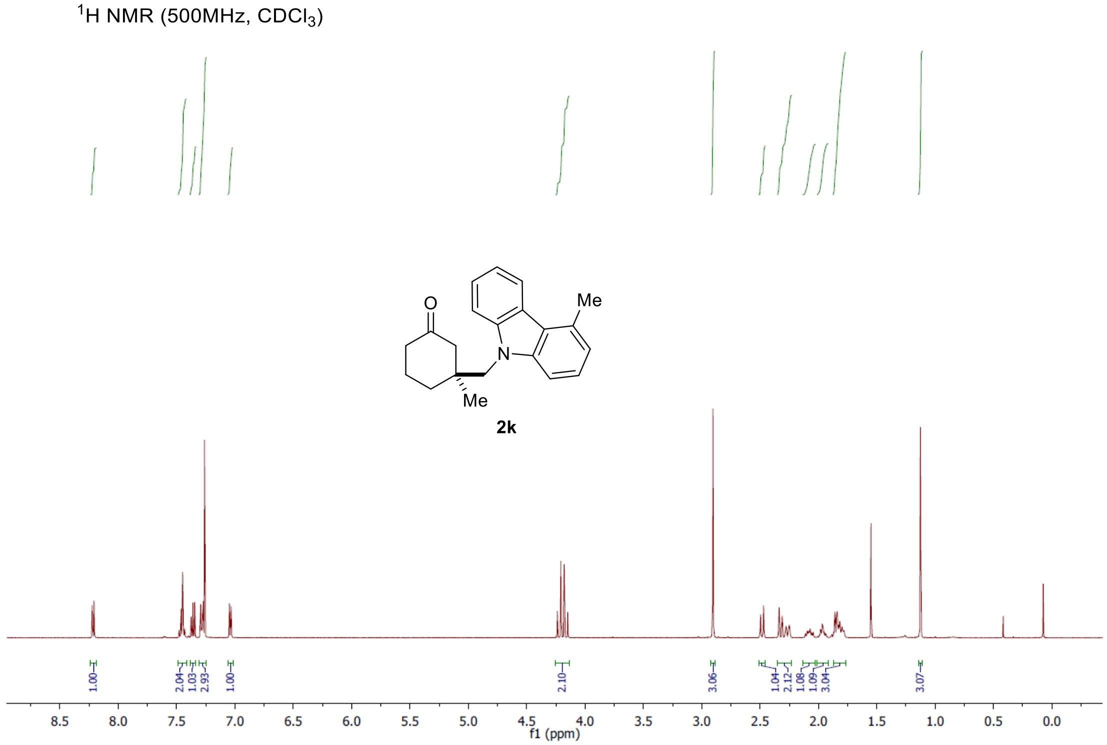

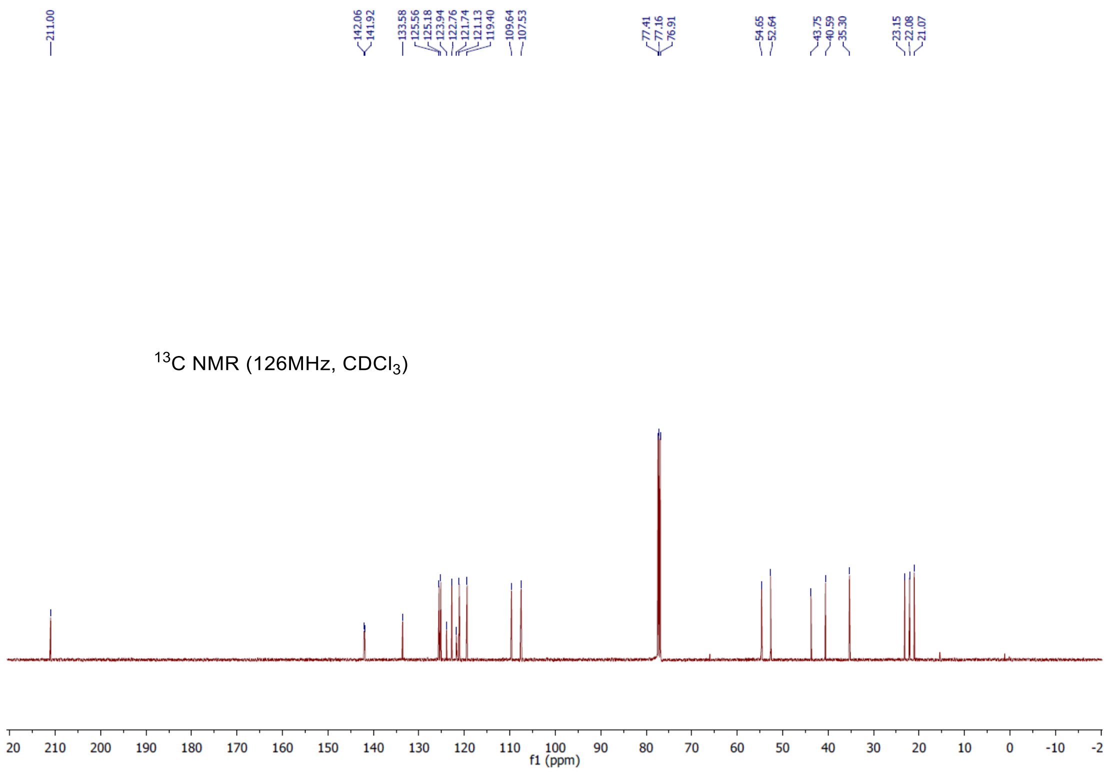

Supplementary Figure 24. ^{1}H and ^{13}C NMR spectra for compound **2k**

^{1}H NMR (500MHz, $CDCl_3$)

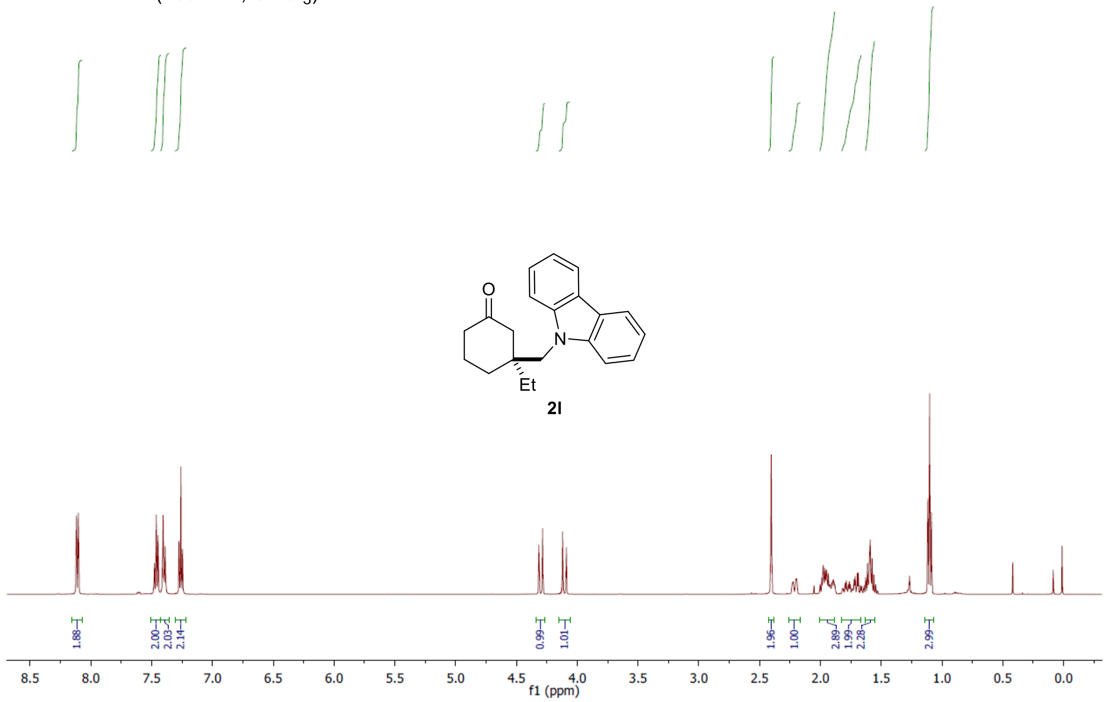

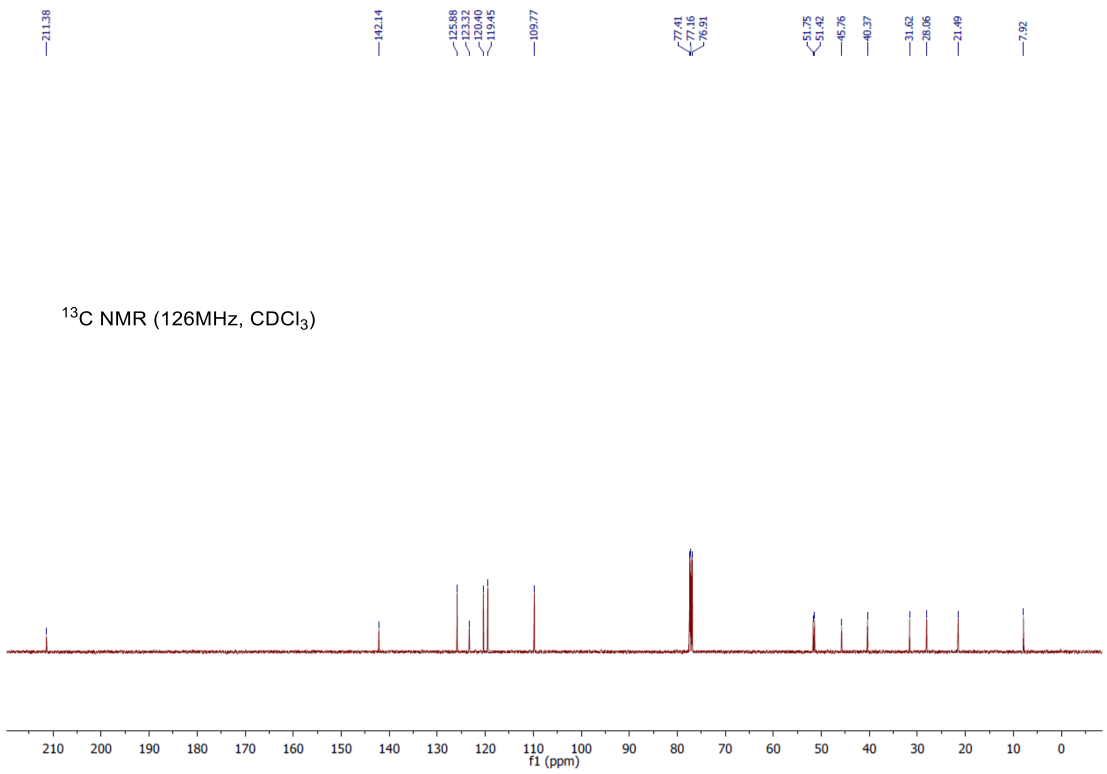

Supplementary Figure 25. ^{1}H and ^{13}C NMR spectra for compound **2l**

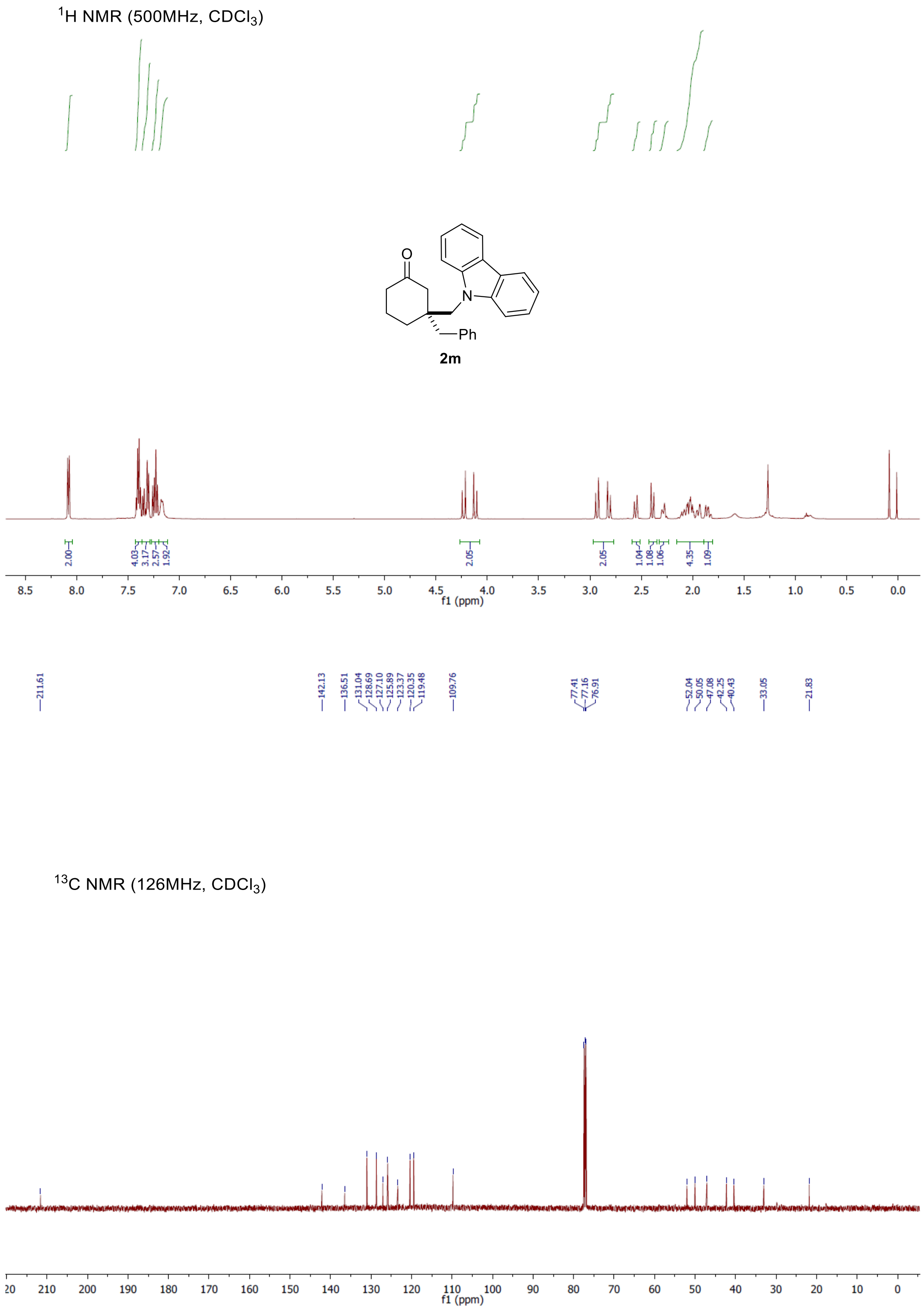

Supplementary Figure 26. ^{1}H and ^{13}C NMR spectra for compound **2m**

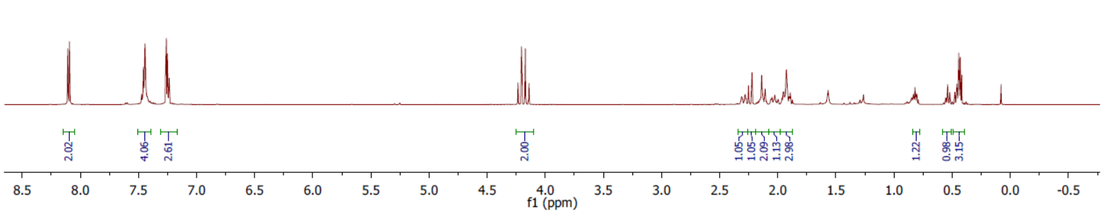

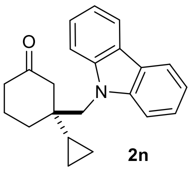

2n

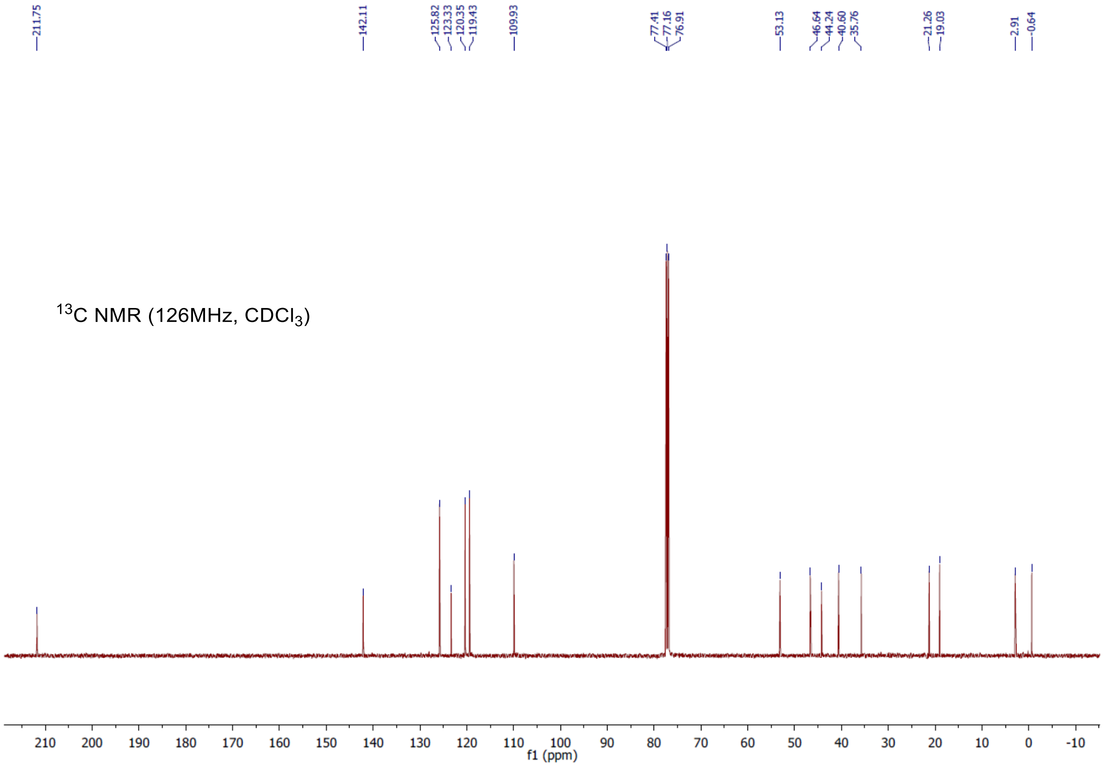

Supplementary Figure 27. ^{1}H and ^{13}C NMR spectra for compound **2n**

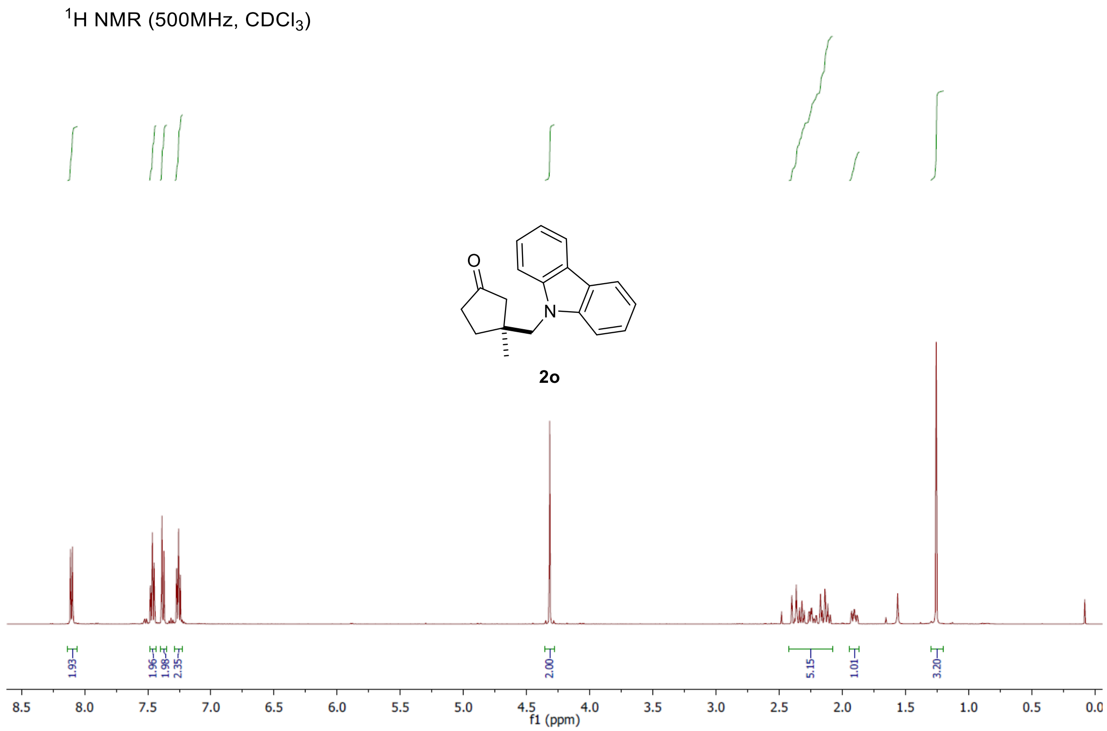

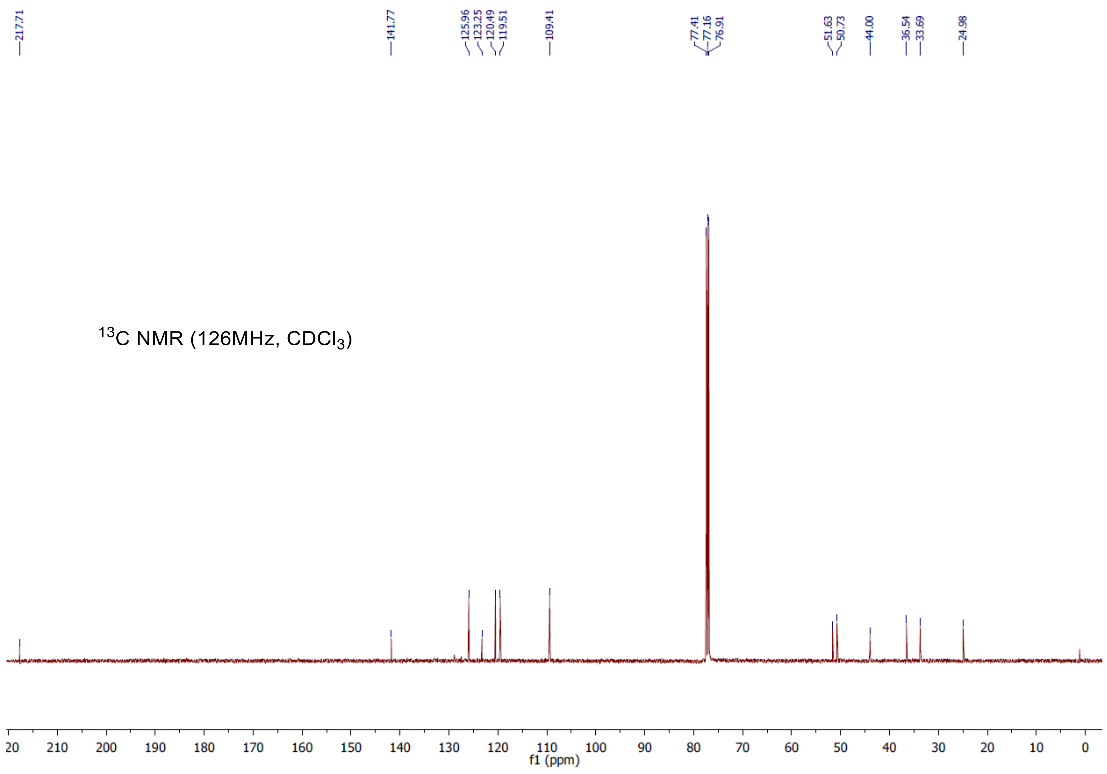

Supplementary Figure 28. ^{1}H and ^{13}C NMR spectra for compound **2o**

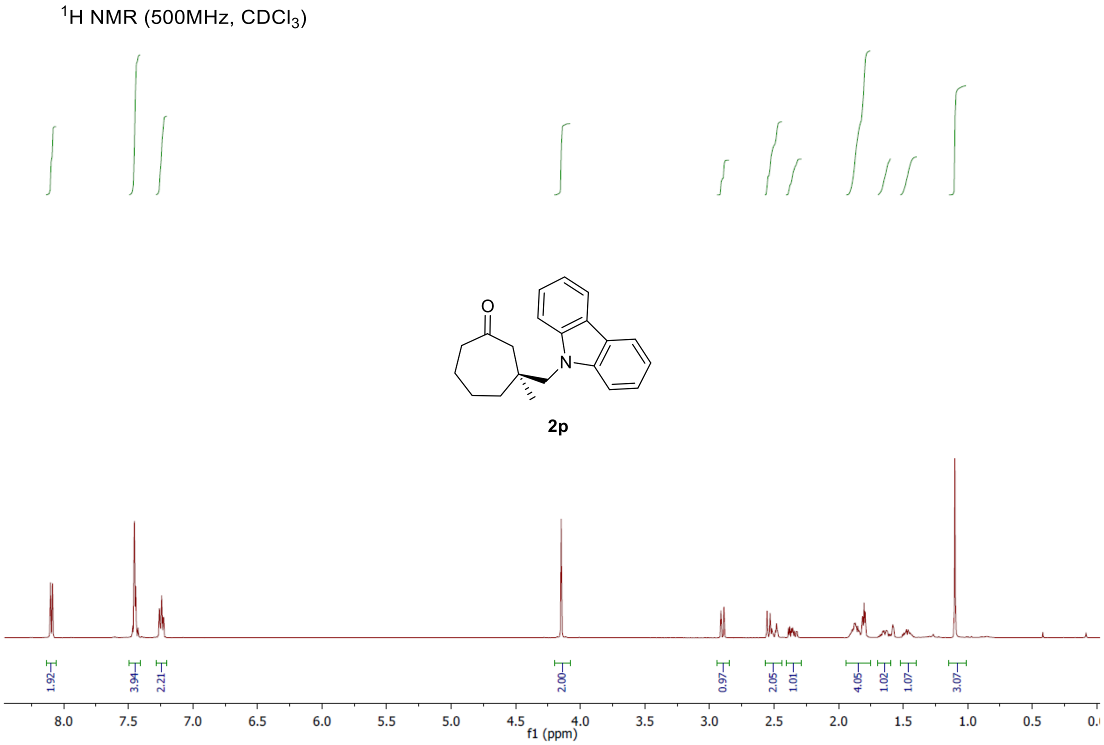

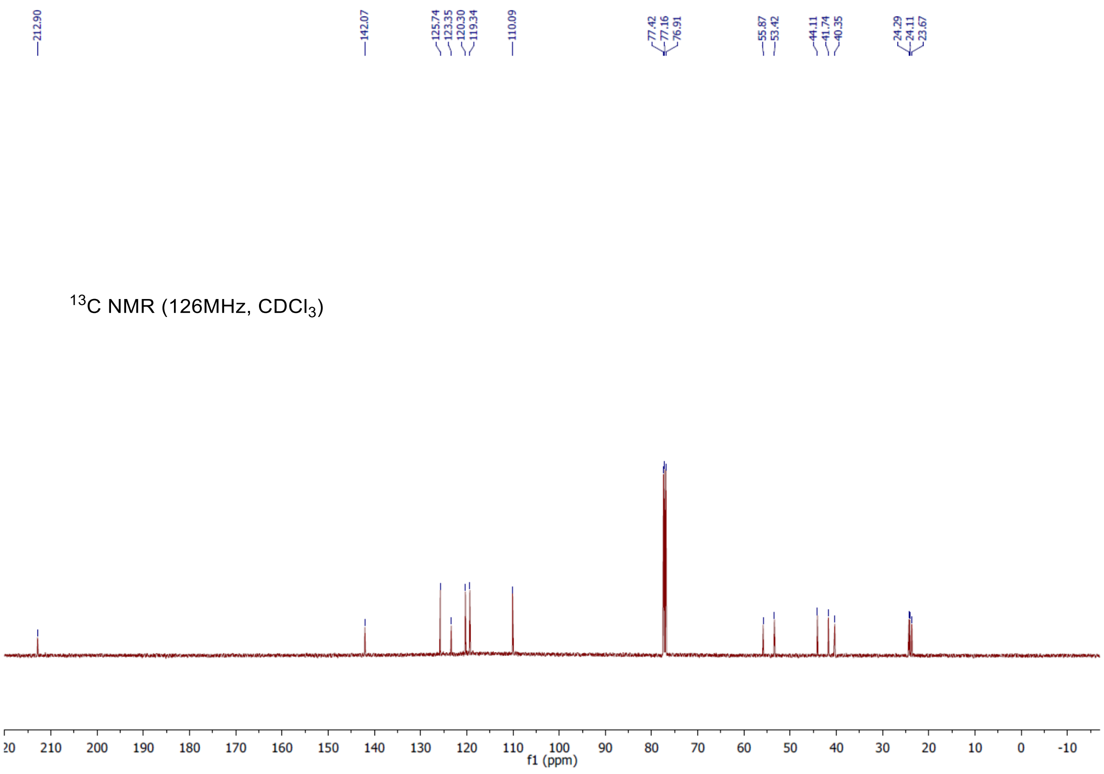

Supplementary Figure 29. ^{1}H and ^{13}C NMR spectra for compound **2p**

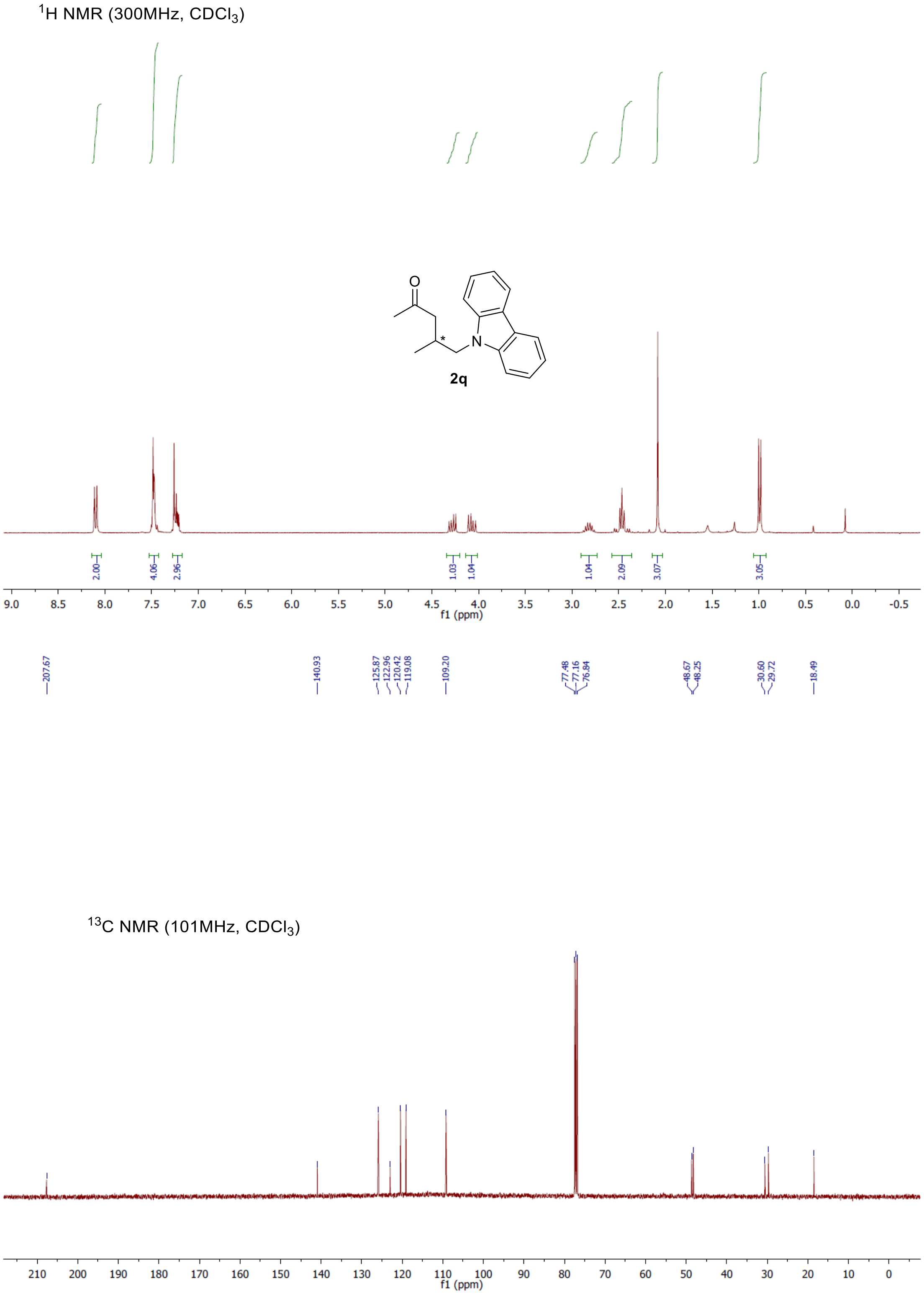

Supplementary Figure 30. ^{1}H and ^{13}C NMR spectra for compound **2q**

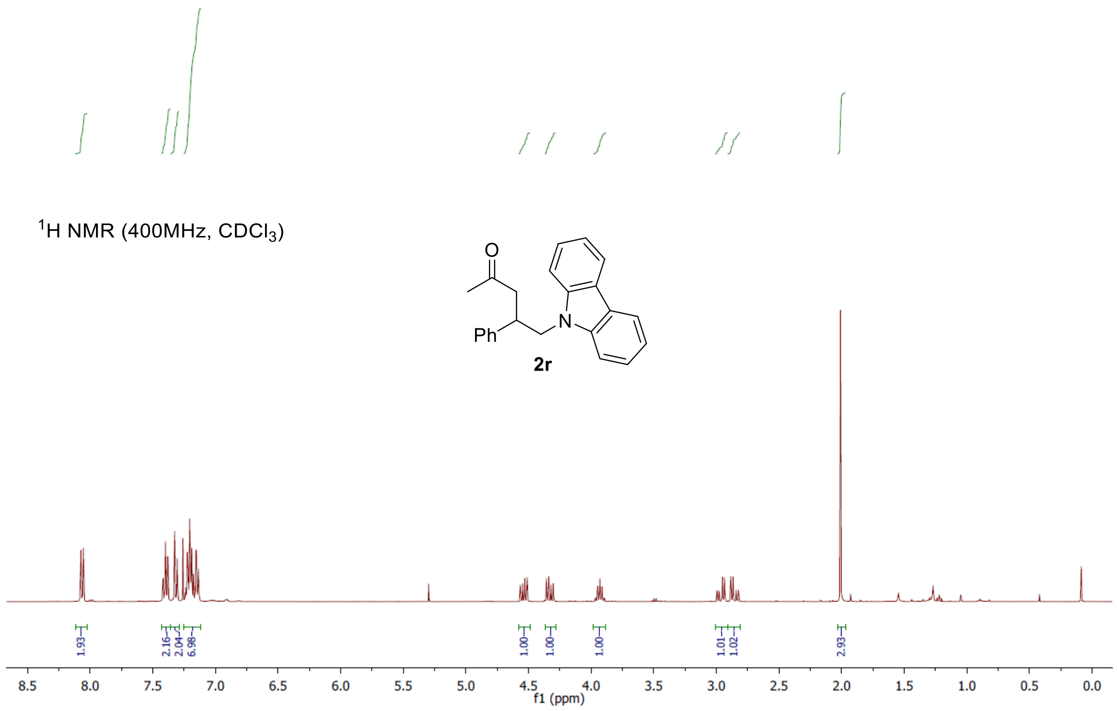

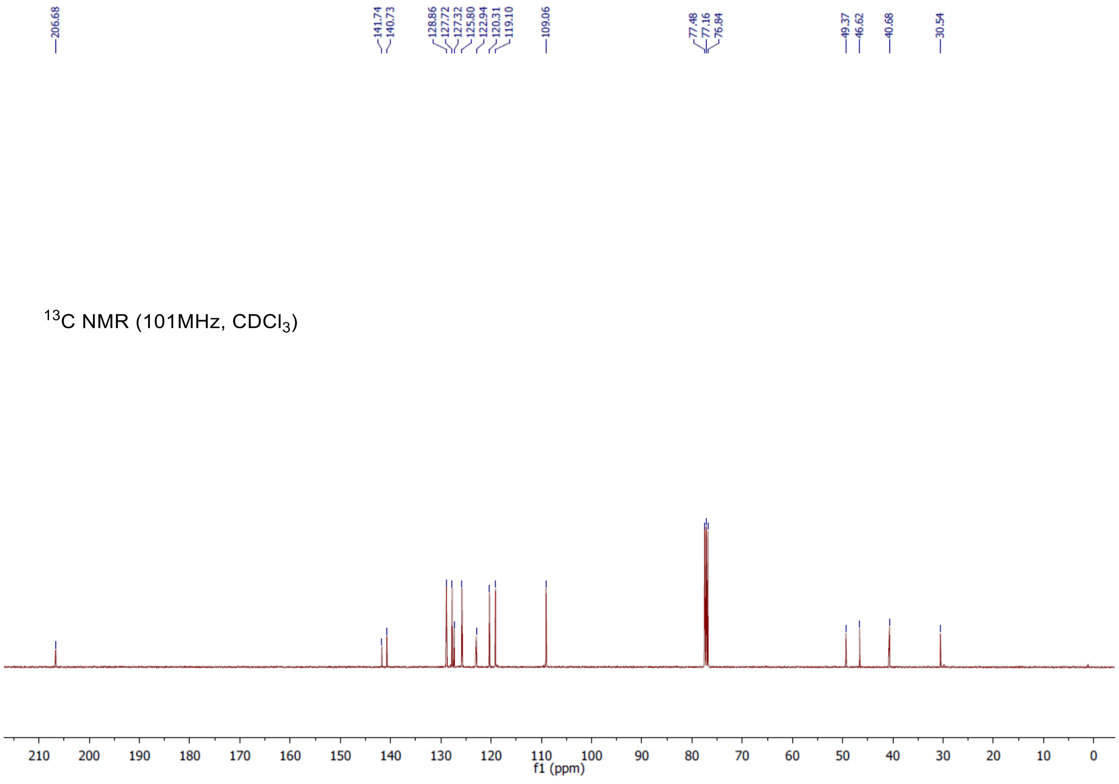

Supplementary Figure 31. ^{1}H and ^{13}C NMR spectra for compound **2r**

^{1}H NMR (500MHz, $CDCl_3$)

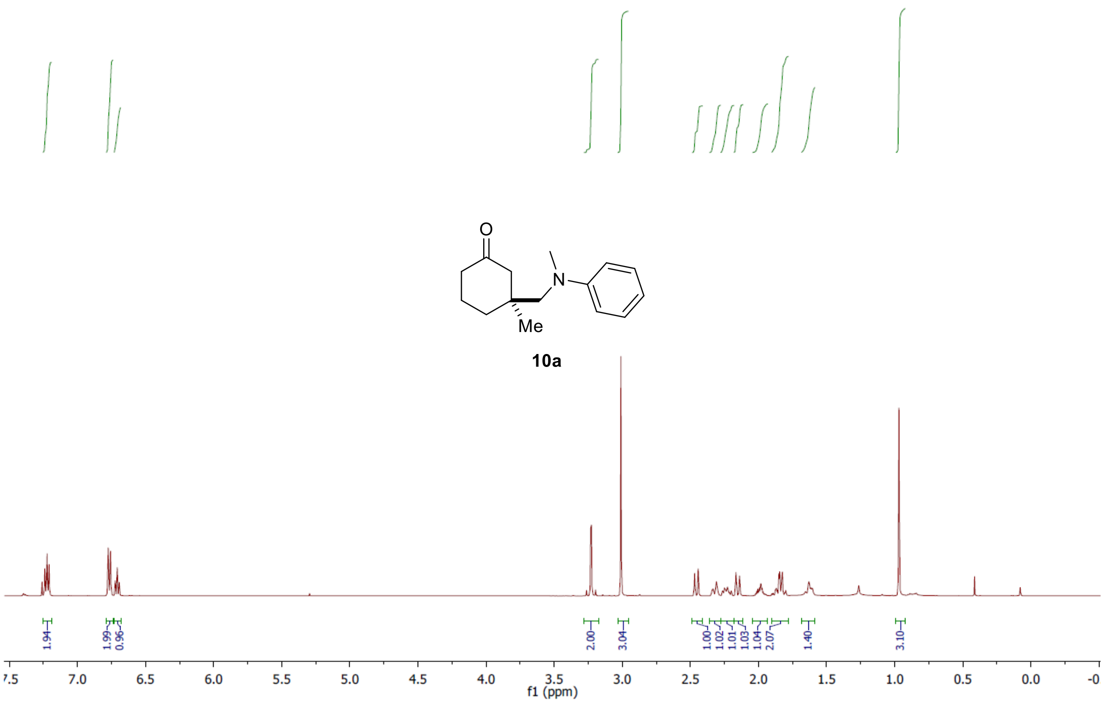

^{13}C NMR (126MHz, $CDCl_3$)

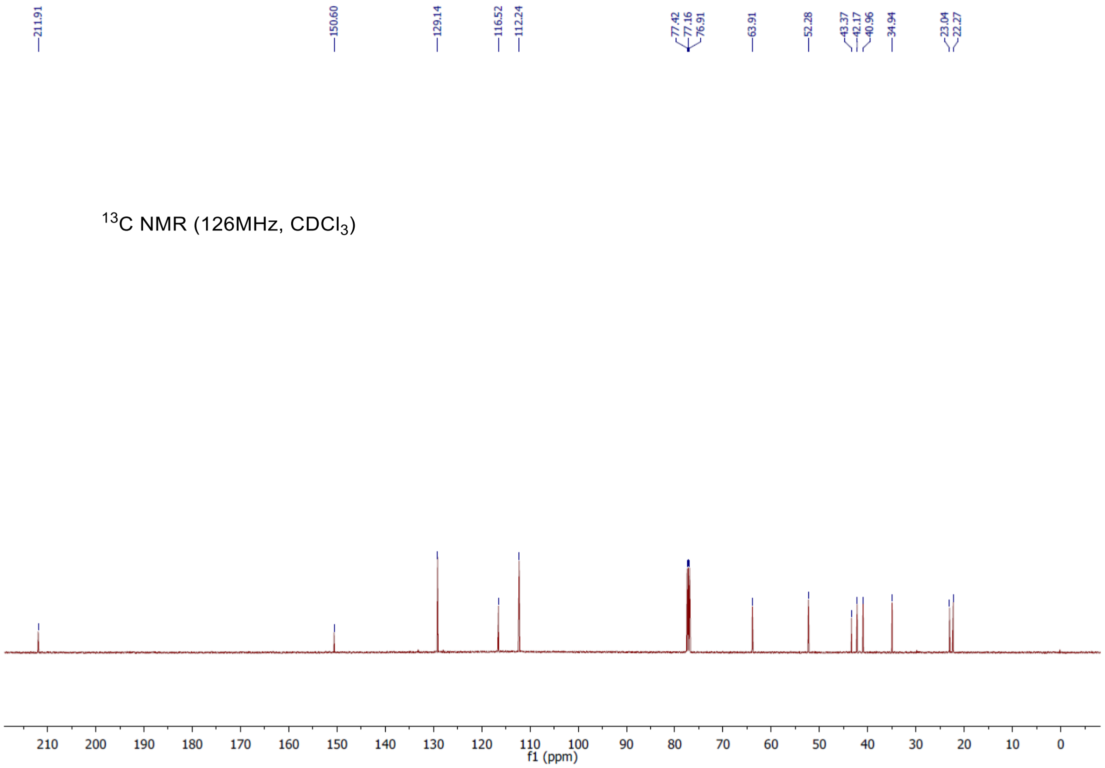

Supplementary Figure 32. ^{1}H and ^{13}C NMR spectra for compound **10a**

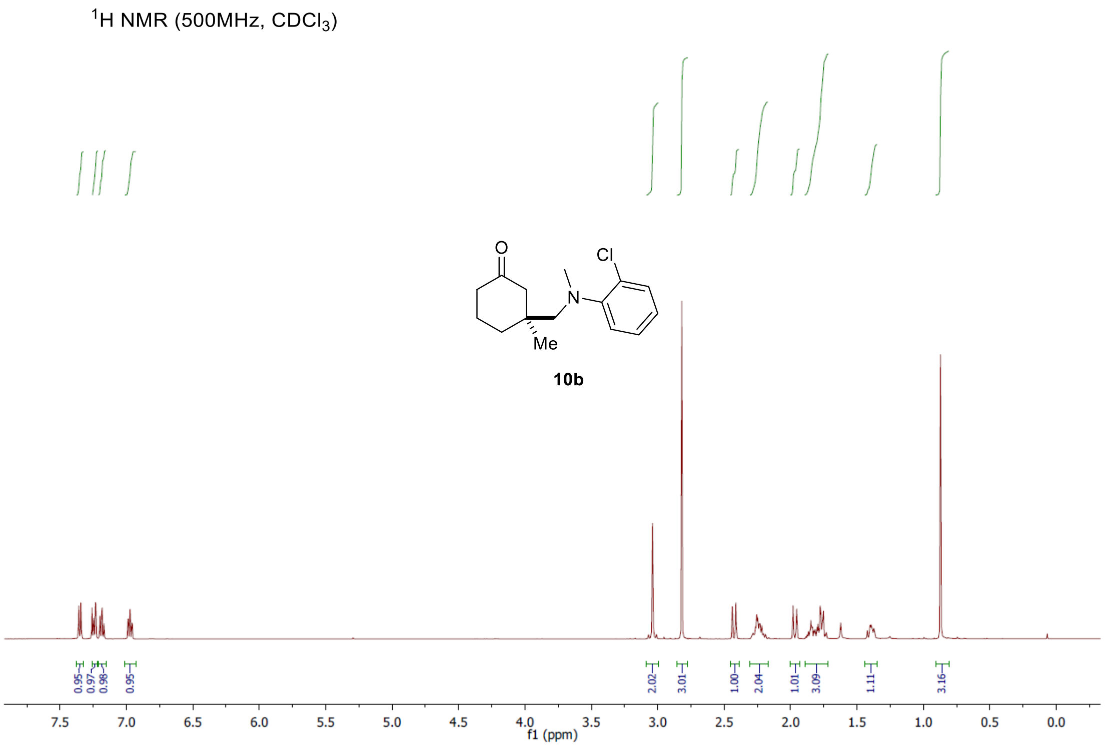

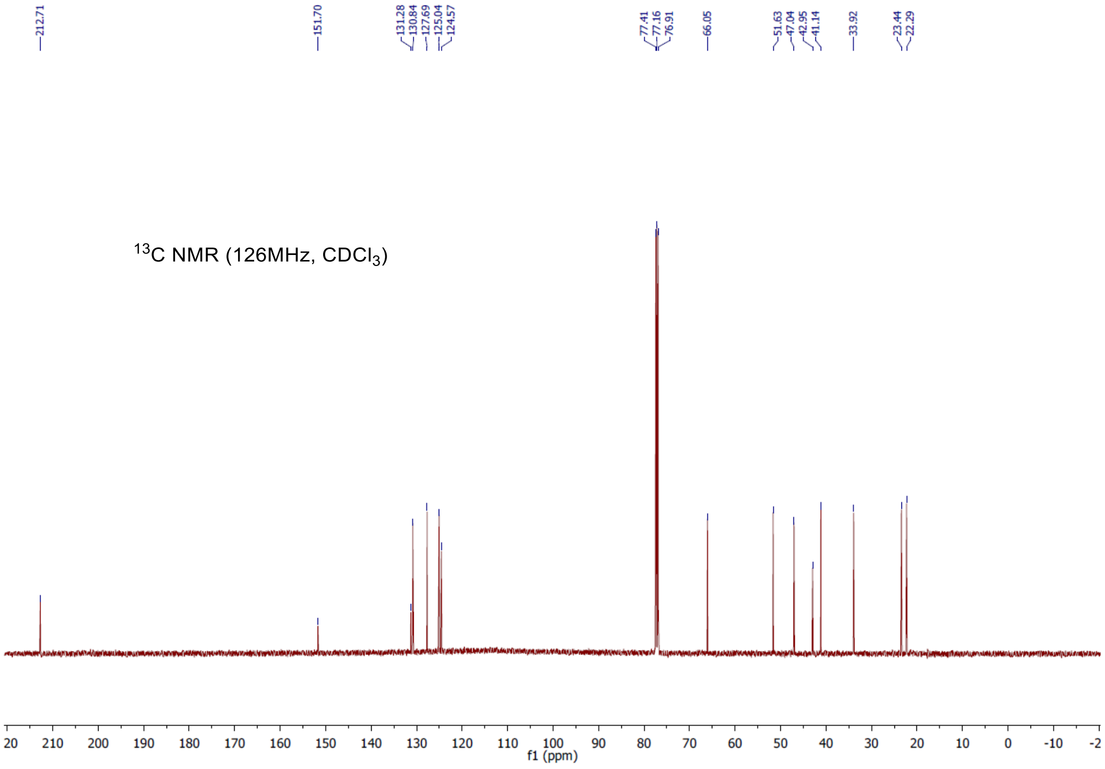

Supplementary Figure 33. ^{1}H and ^{13}C NMR spectra for compound **10b**

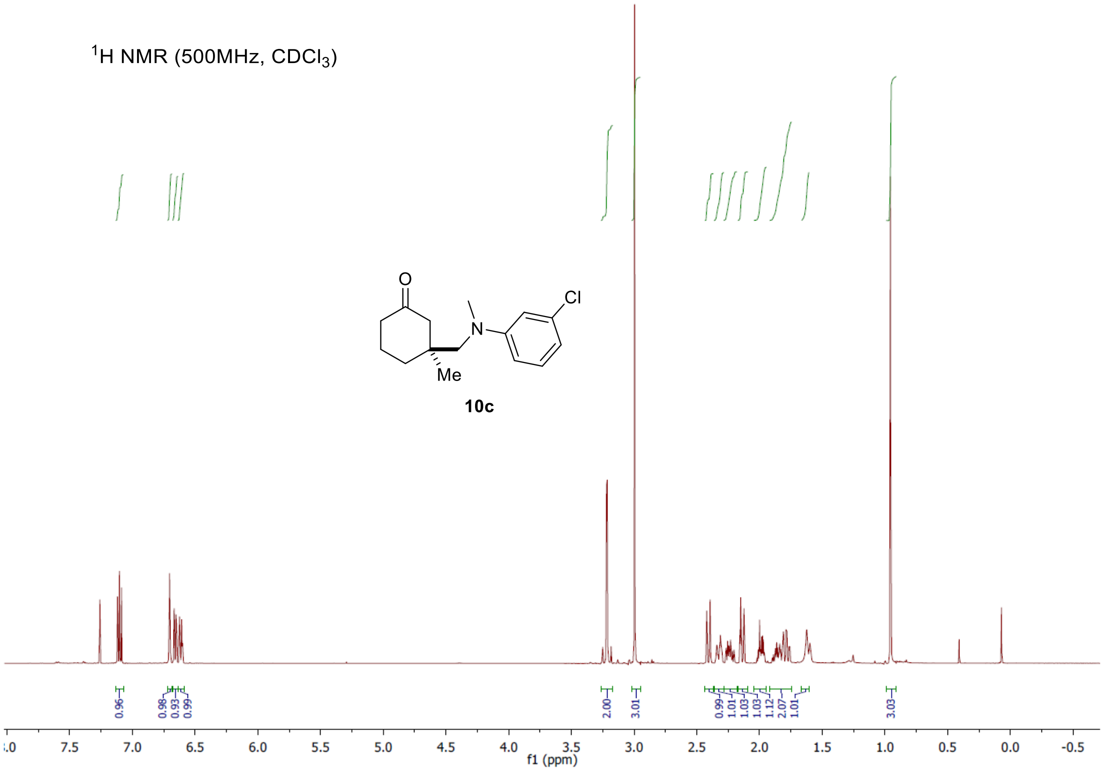

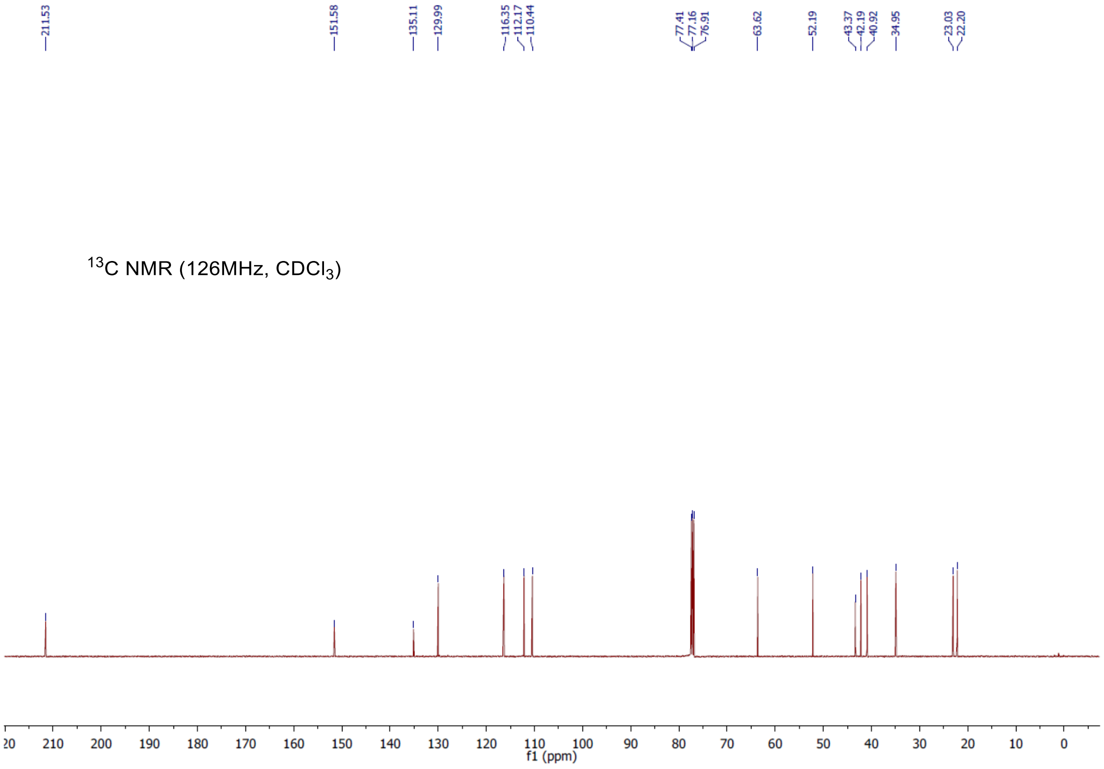

Supplementary Figure 34. ^{1}H and ^{13}C NMR spectra for compound **10c**

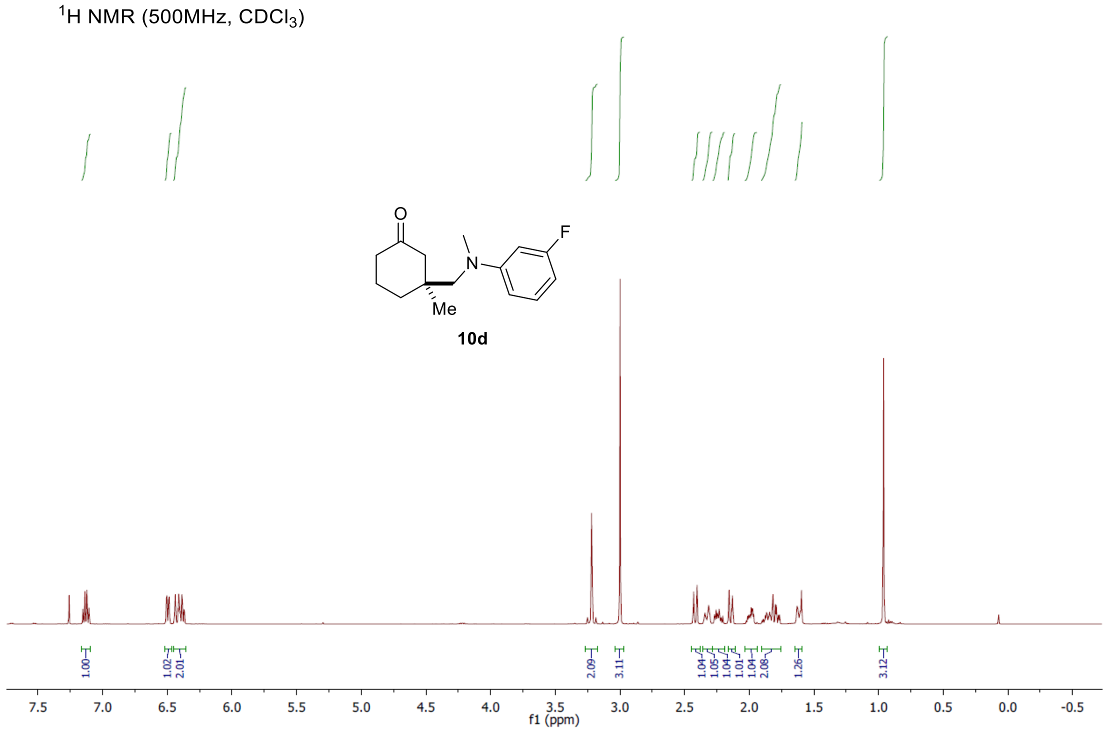

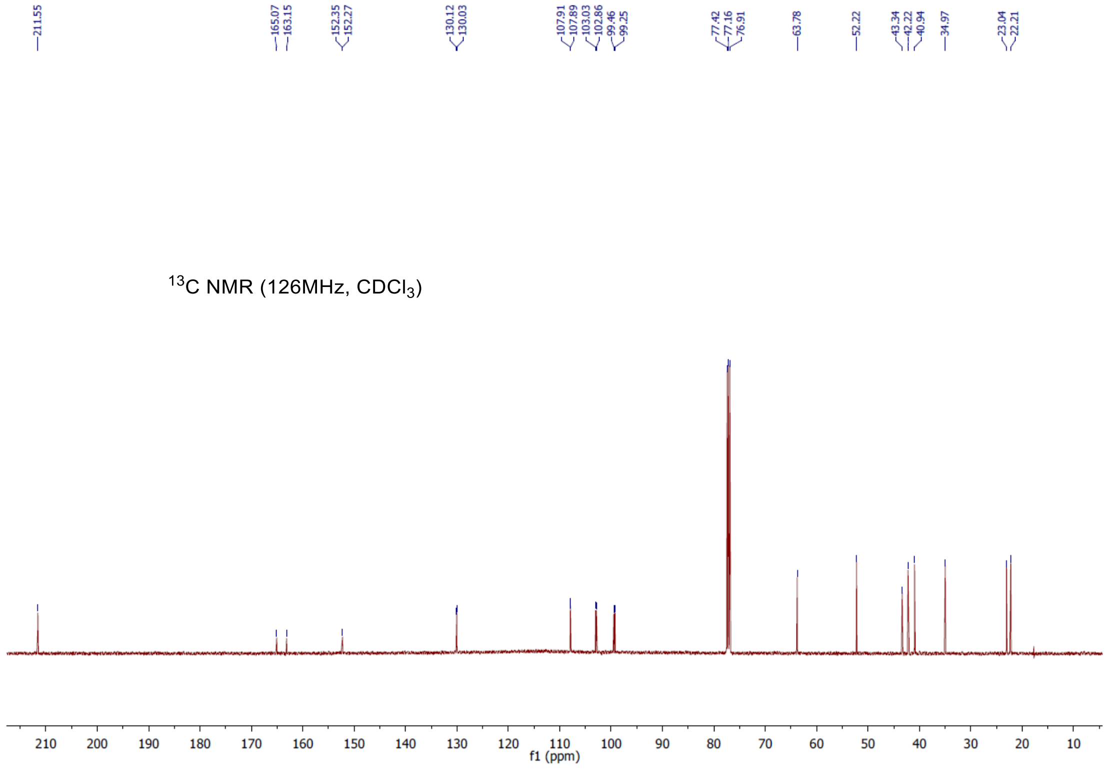

Supplementary Figure 35. ^{1}H and ^{13}C NMR spectra for compound **10d**

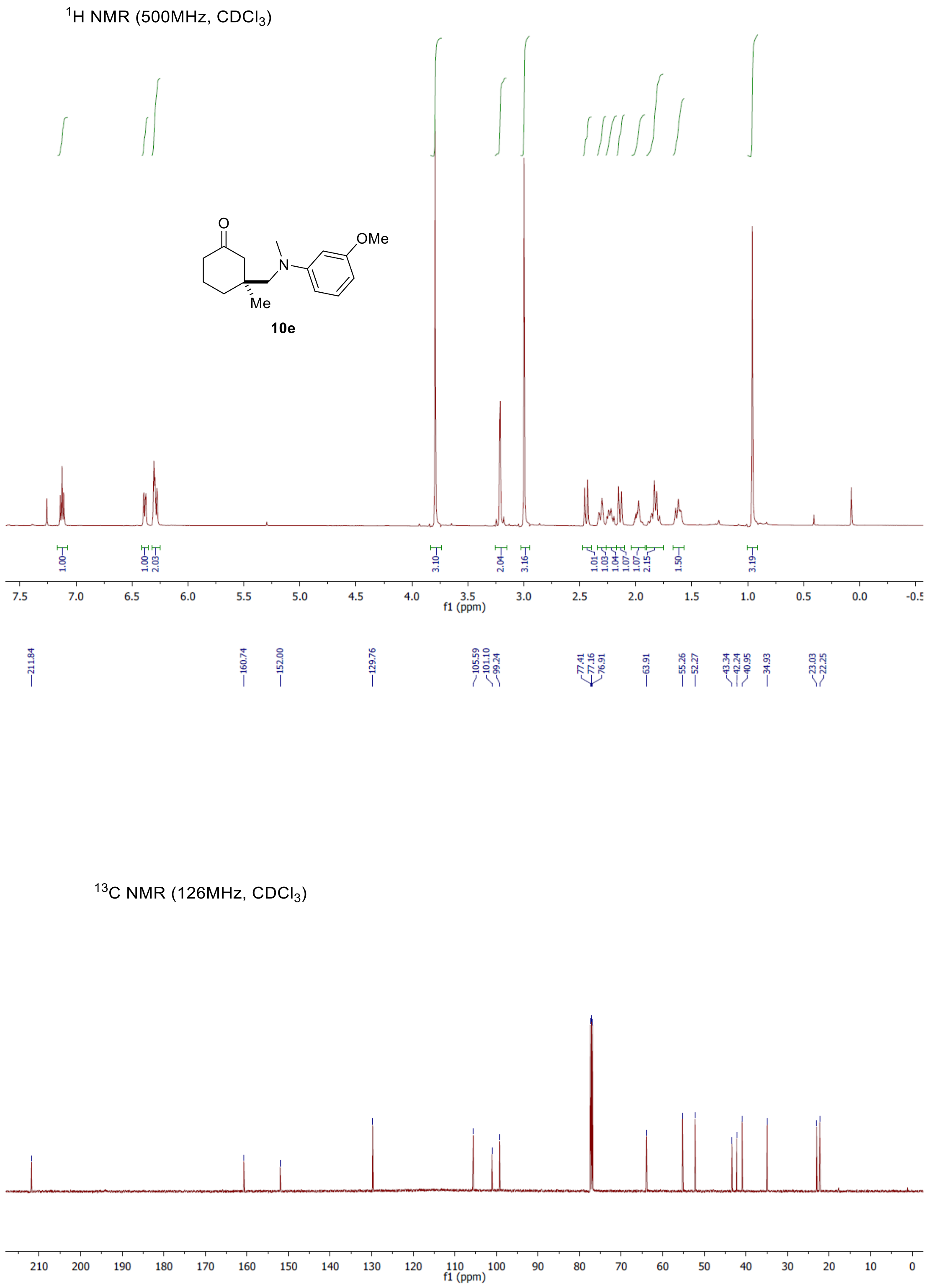

Supplementary Figure 36. ^{1}H and ^{13}C NMR spectra for compound **10e**

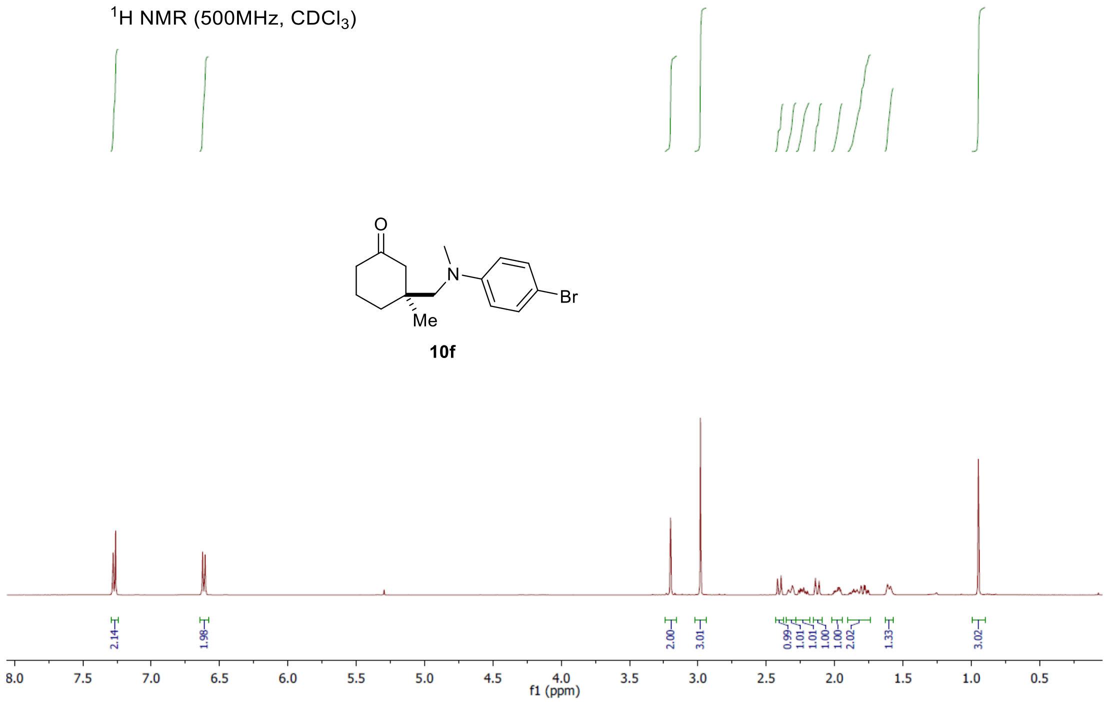

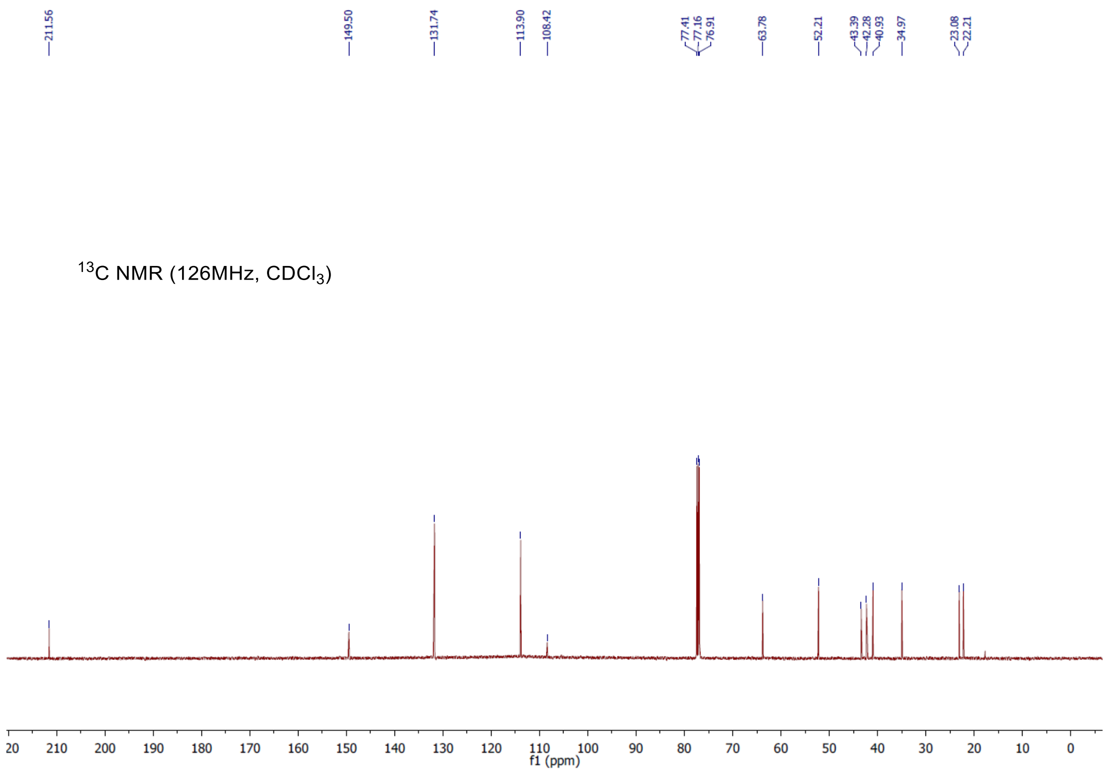

Supplementary Figure 37. ^{1}H and ^{13}C NMR spectra for compound **10f**

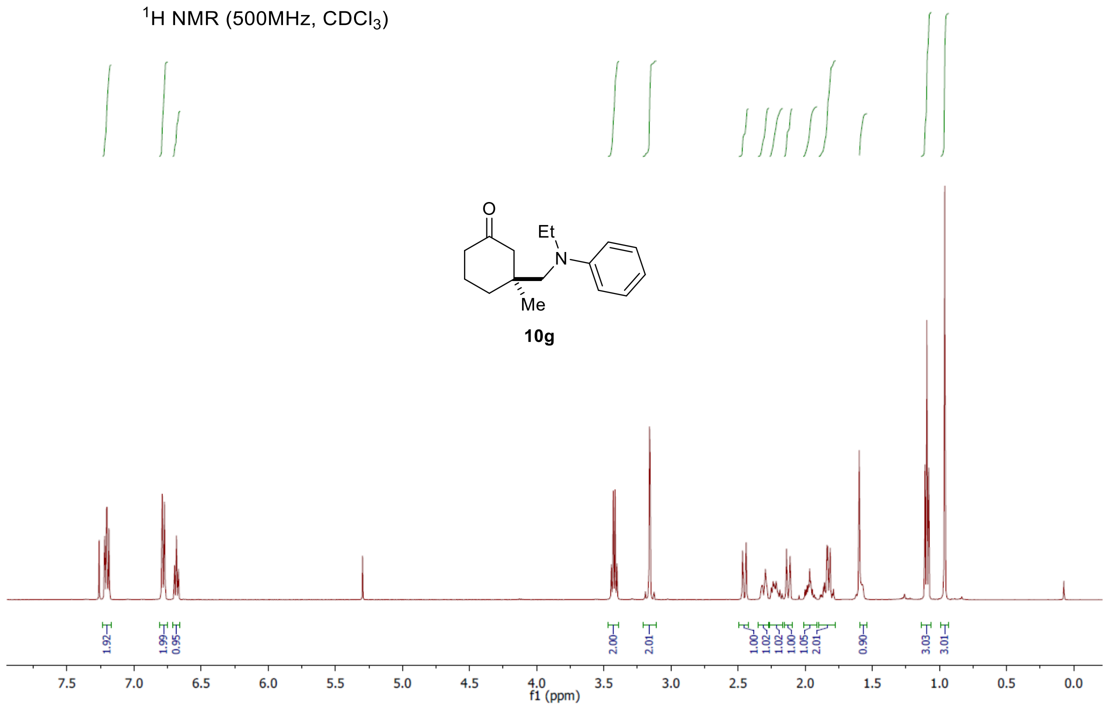

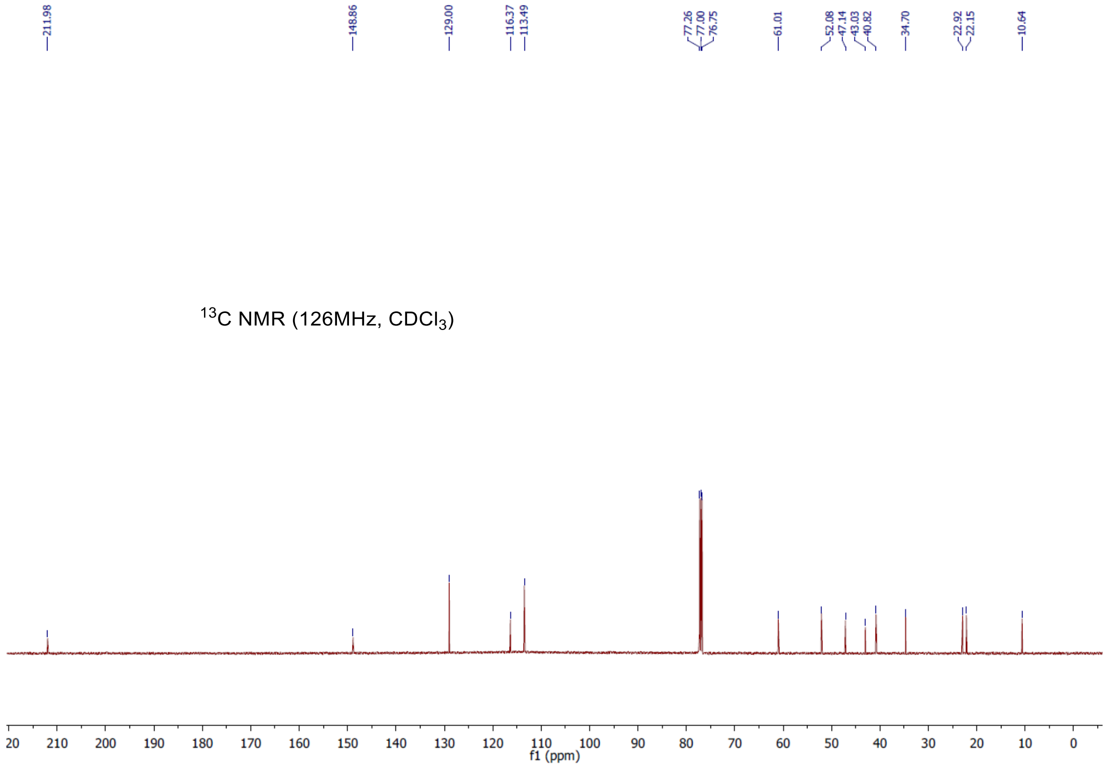

Supplementary Figure 38. ^{1}H and ^{13}C NMR spectra for compound **10g**

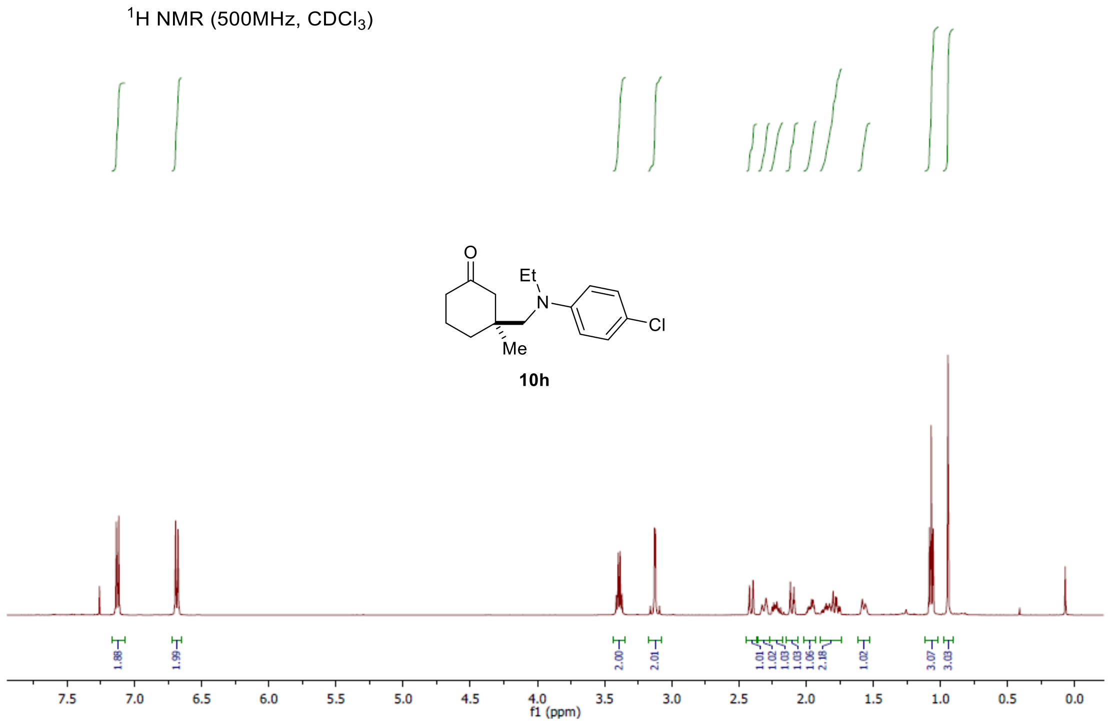

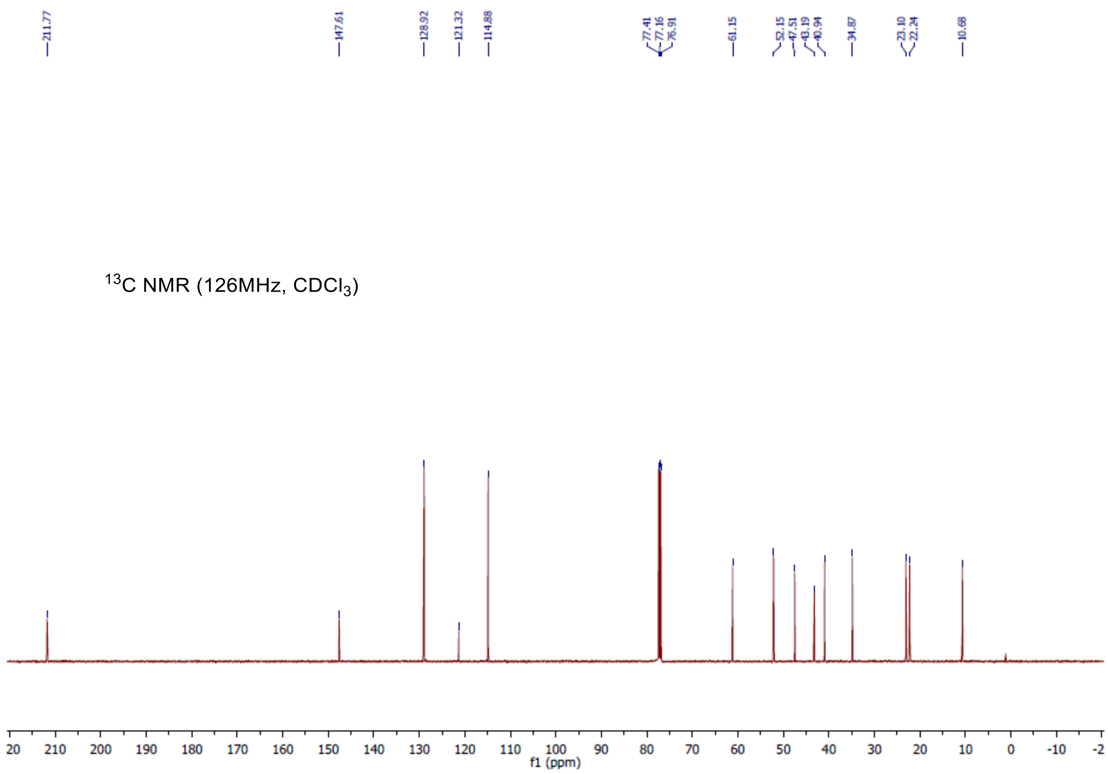

Supplementary Figure 39. ^{1}H and ^{13}C NMR spectra for compound **10h**

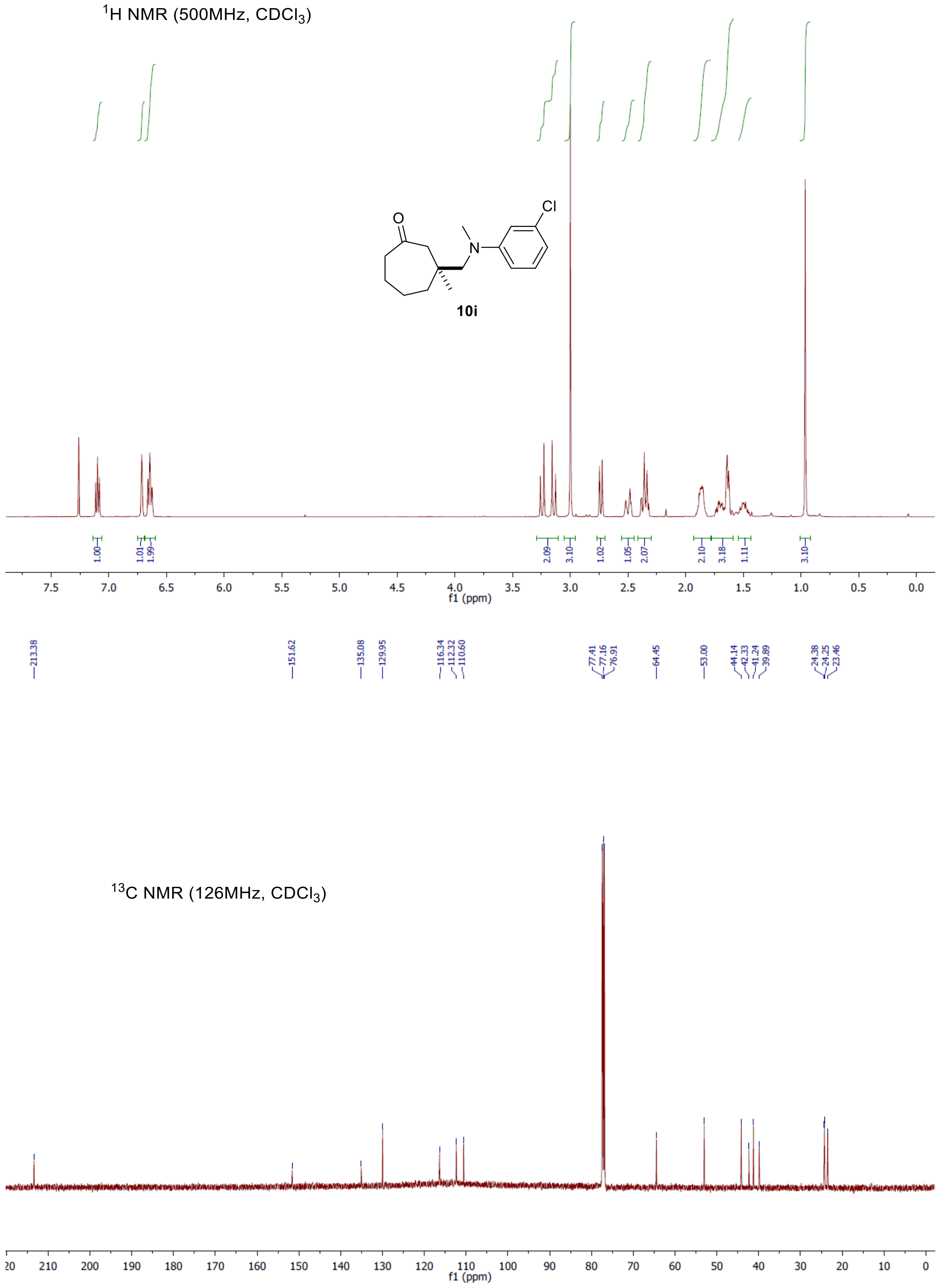

Supplementary Figure 40. ^{1}H and ^{13}C NMR spectra for compound **10i**

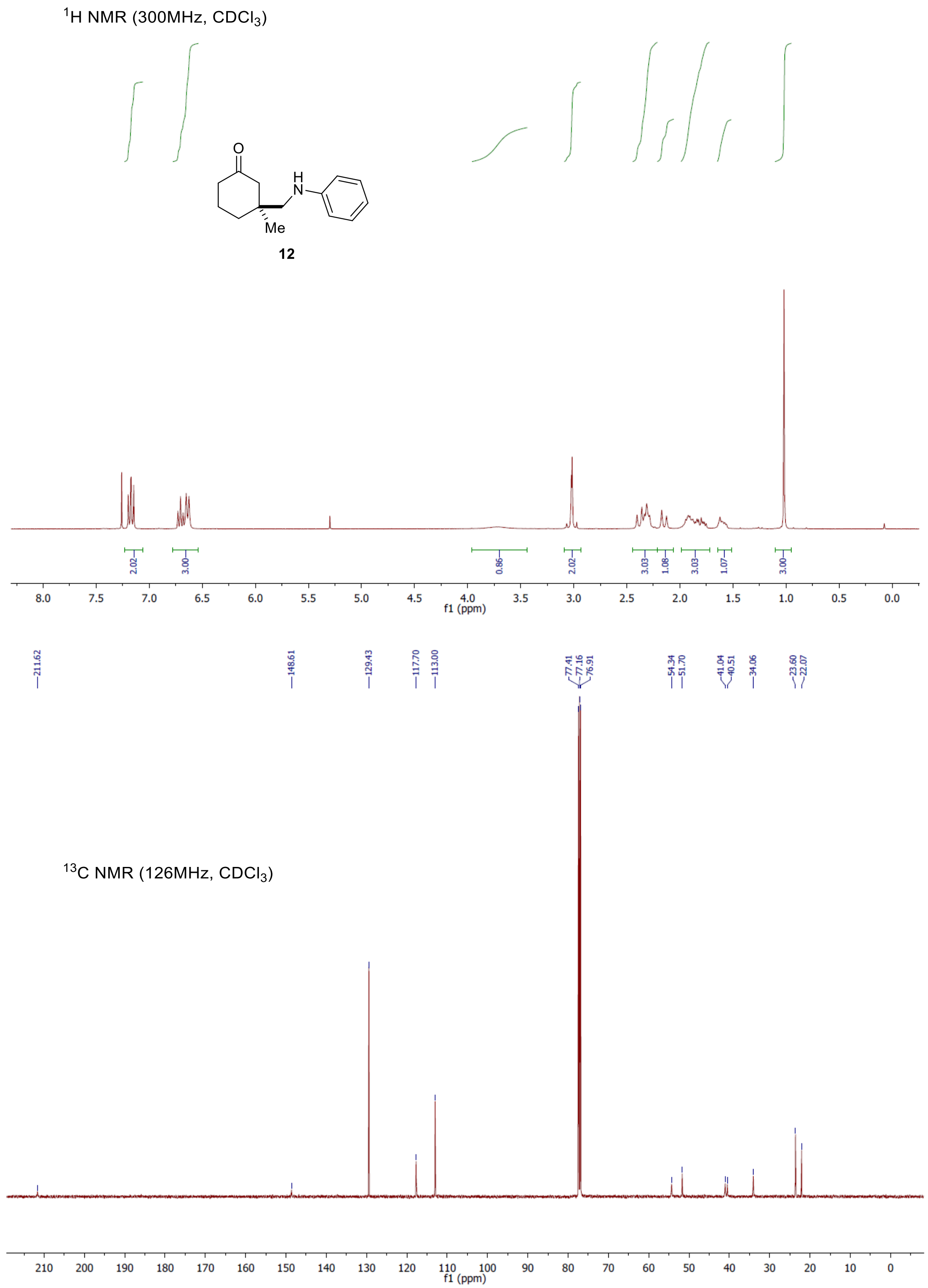

Supplementary Figure 41. ^{1}H and ^{13}C NMR spectra for compound **12**

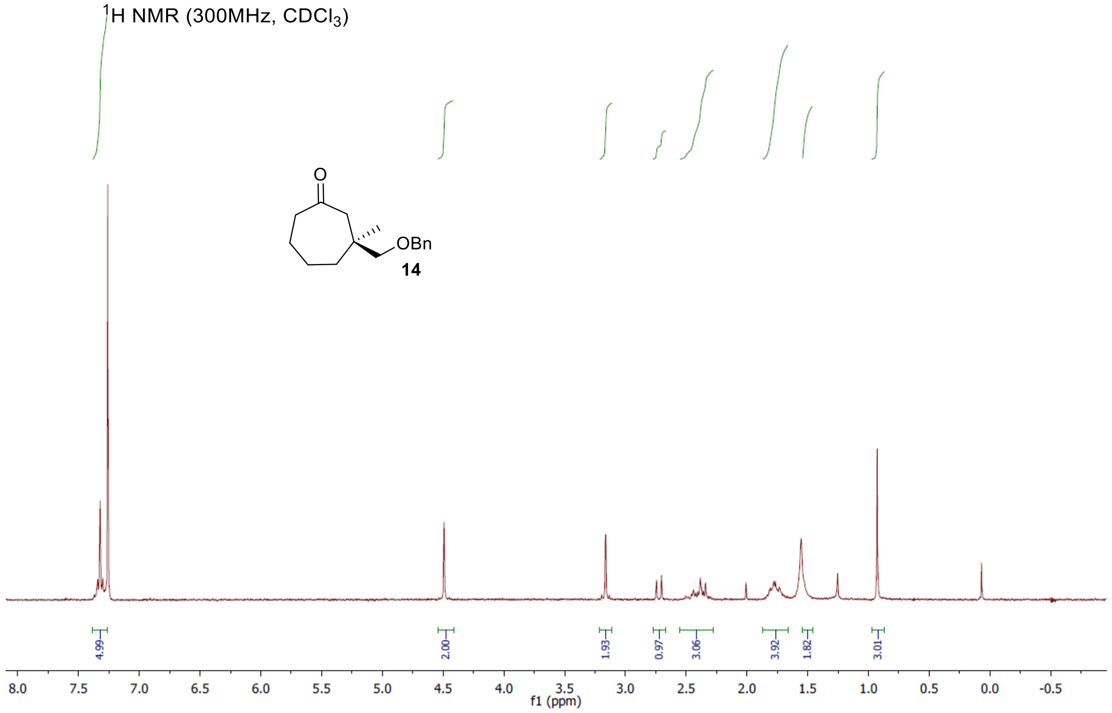

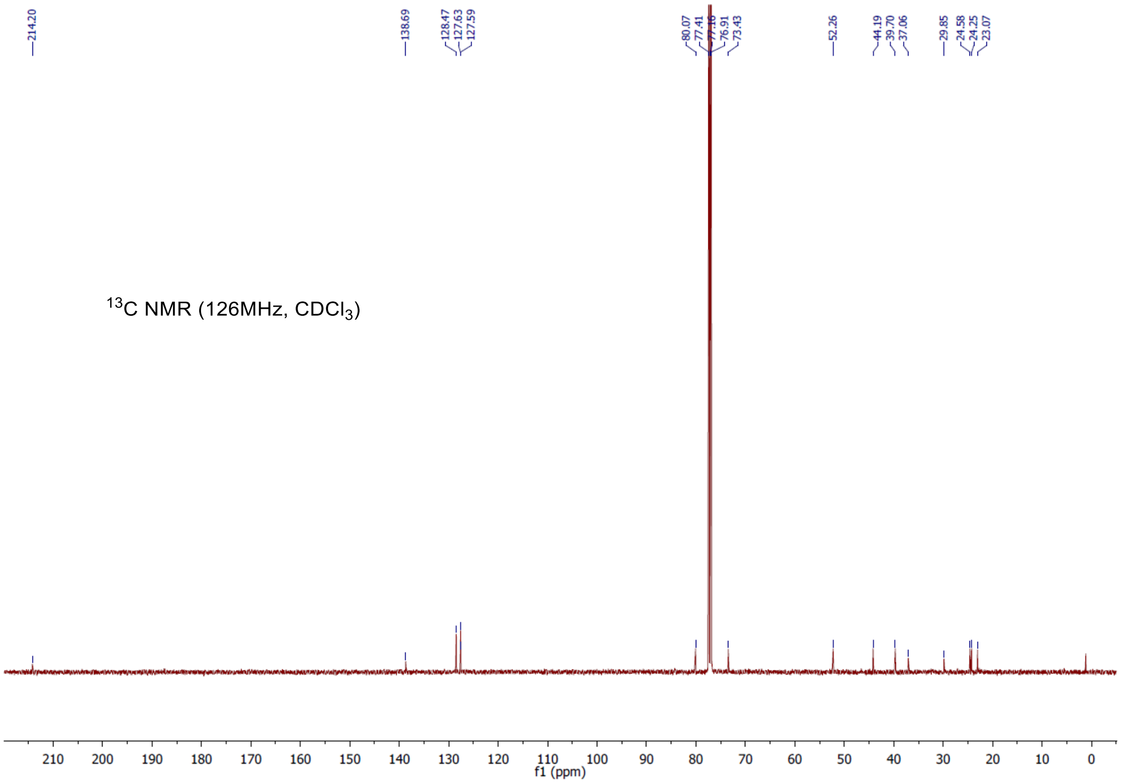

Supplementary Figure 42. ^{1}H and ^{13}C NMR spectra for compound **14**

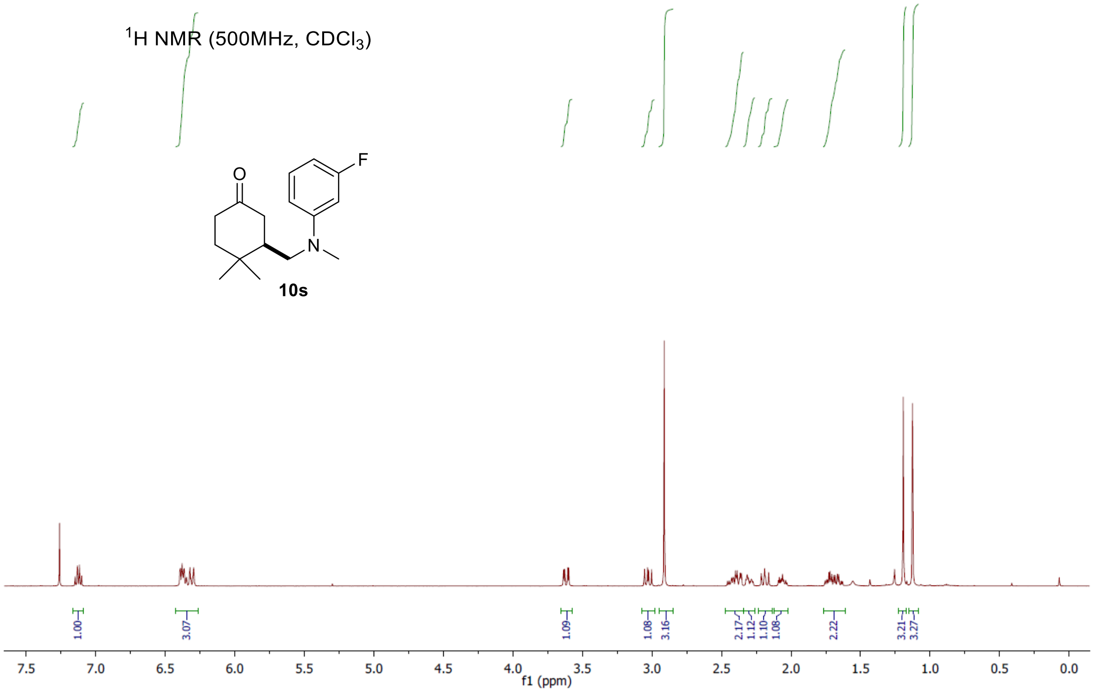

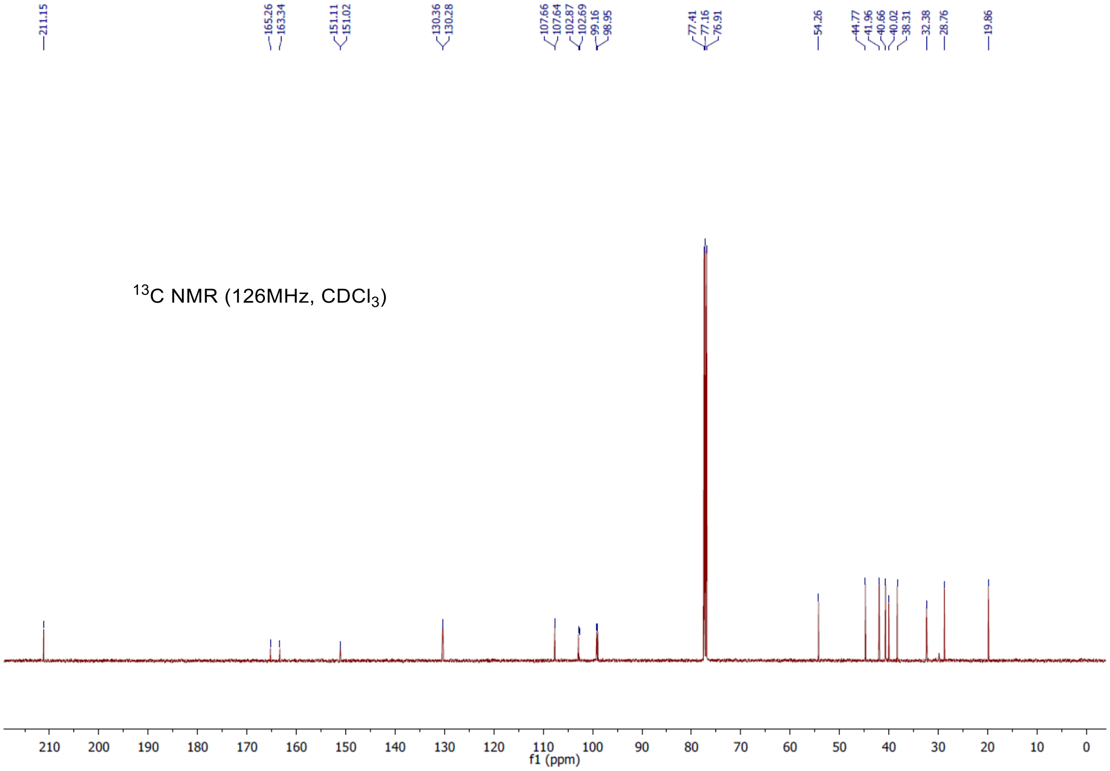

Supplementary Figure 43. ^{1}H and ^{13}C NMR spectra for compound **10s**

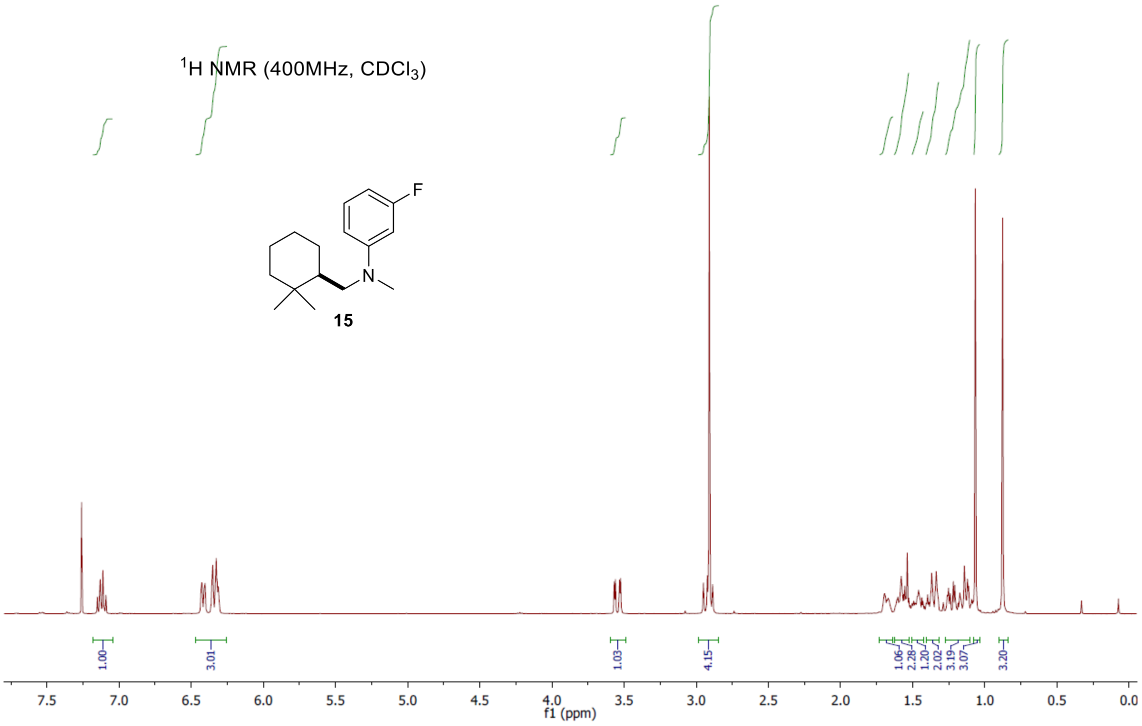

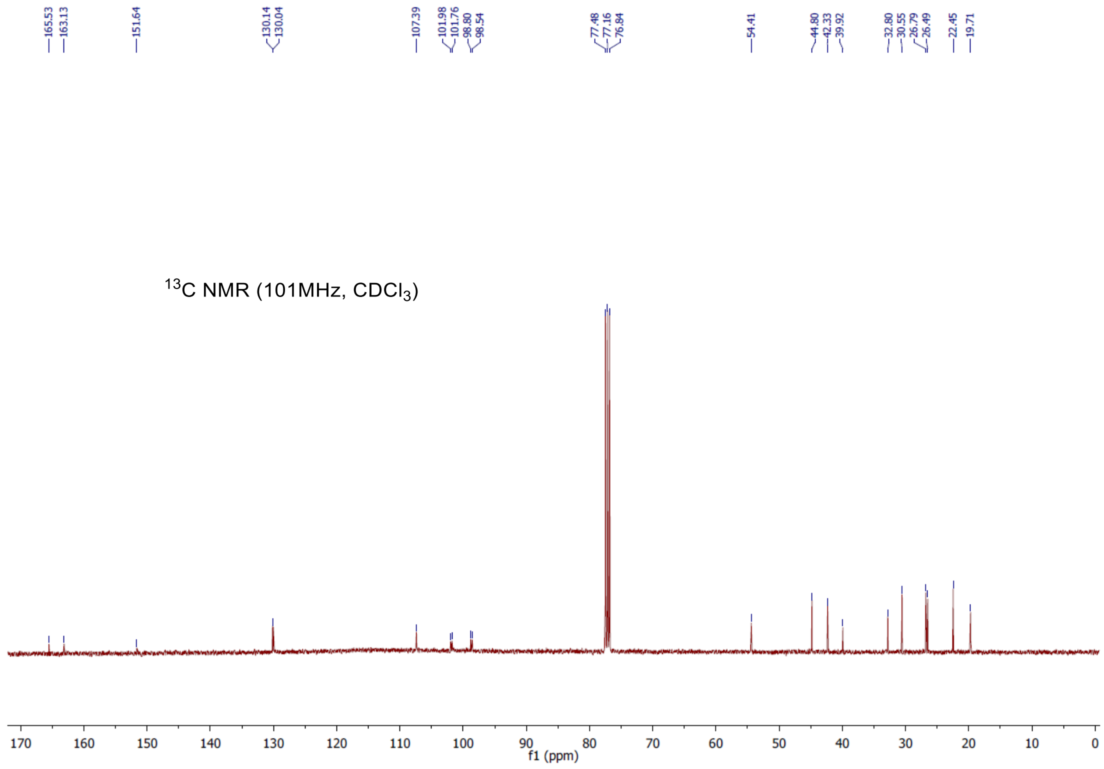

Supplementary Figure 44. ^{1}H and ^{13}C NMR spectra for compound **15**

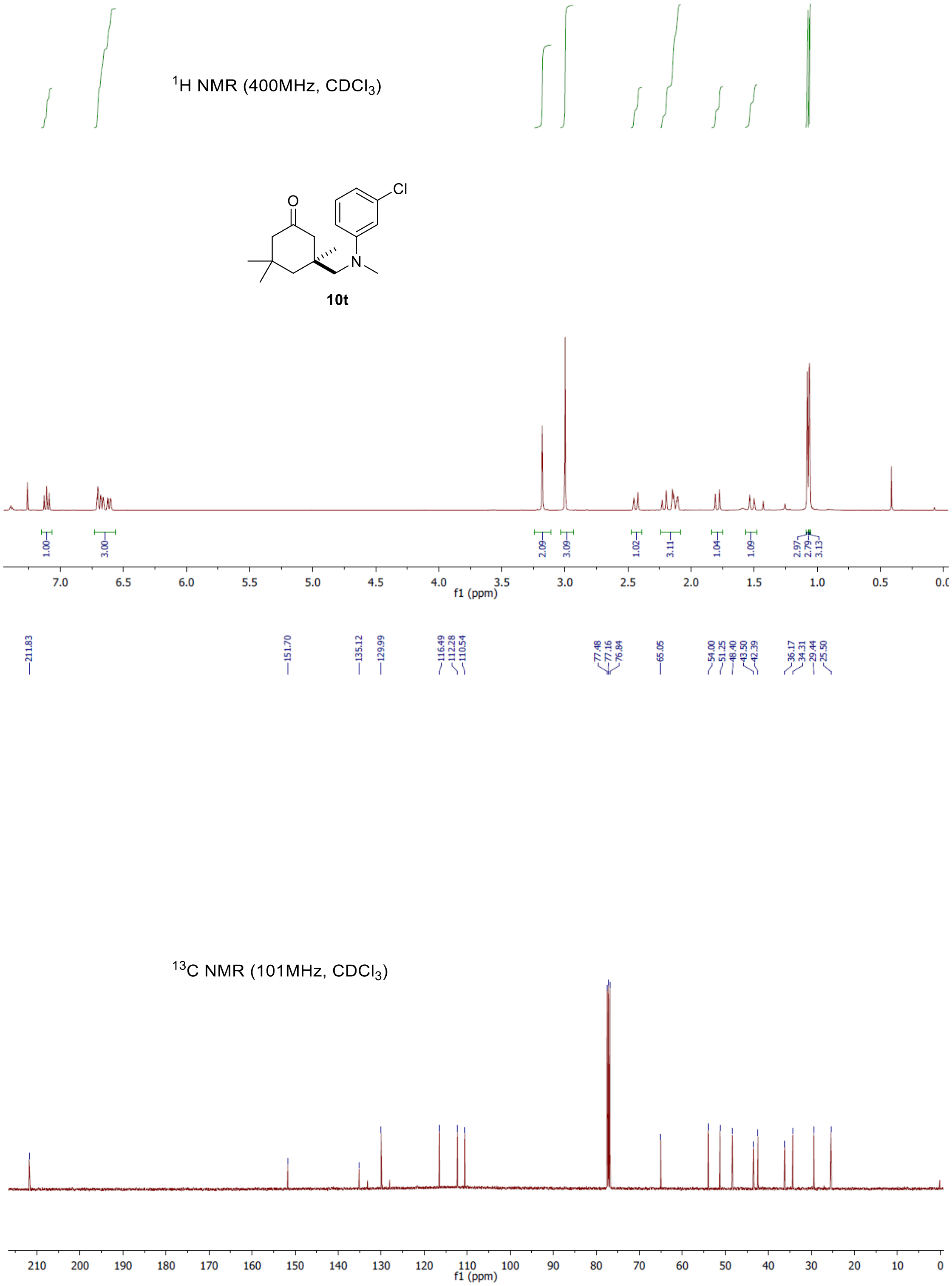

Supplementary Figure 45. ^{1}H and ^{13}C NMR spectra for compound **10t**

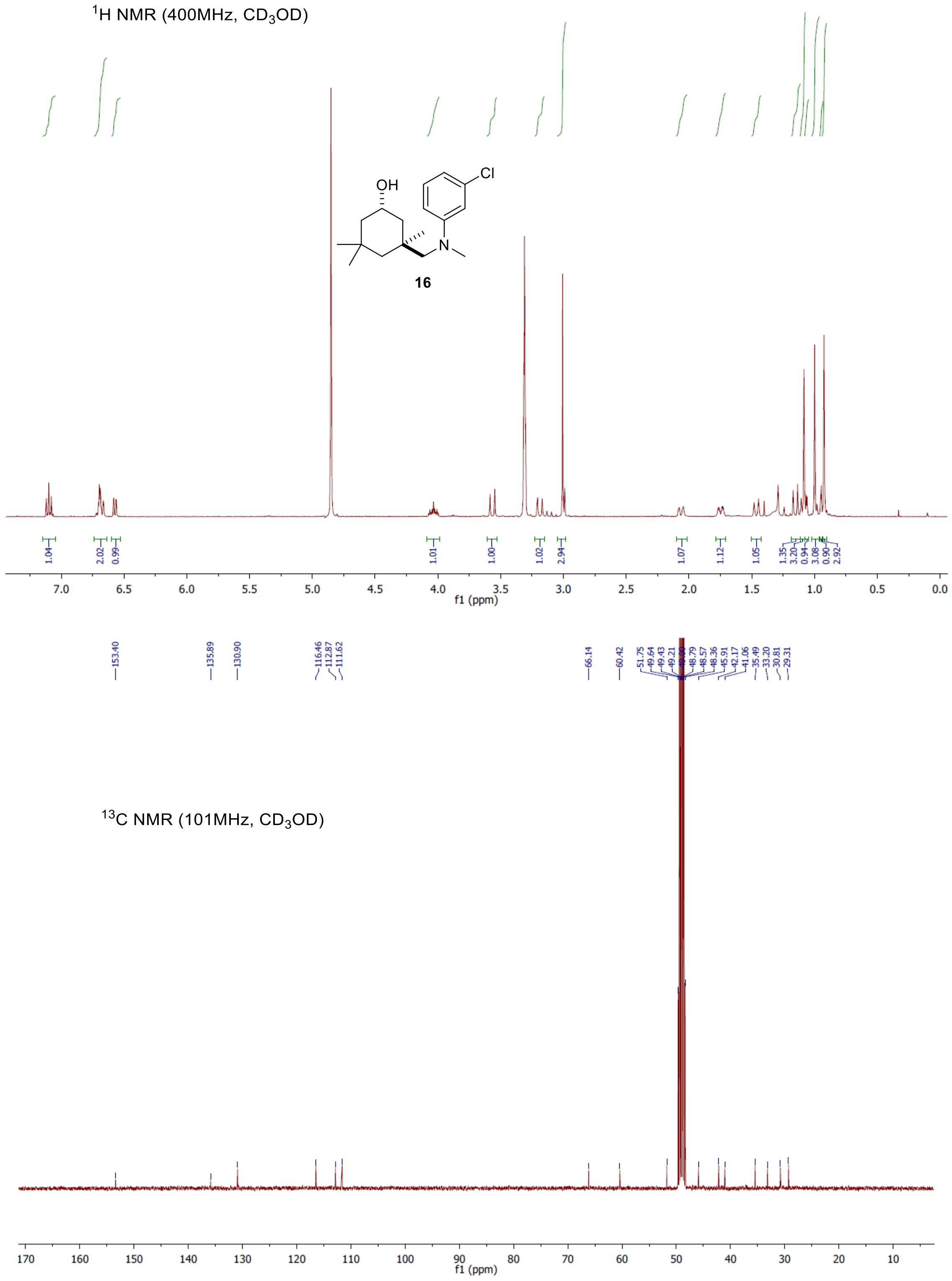

Supplementary Figure 46. ^{1}H and ^{13}C NMR spectra for compound **16**

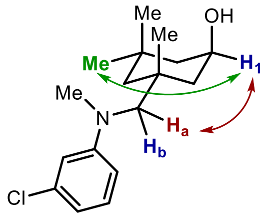

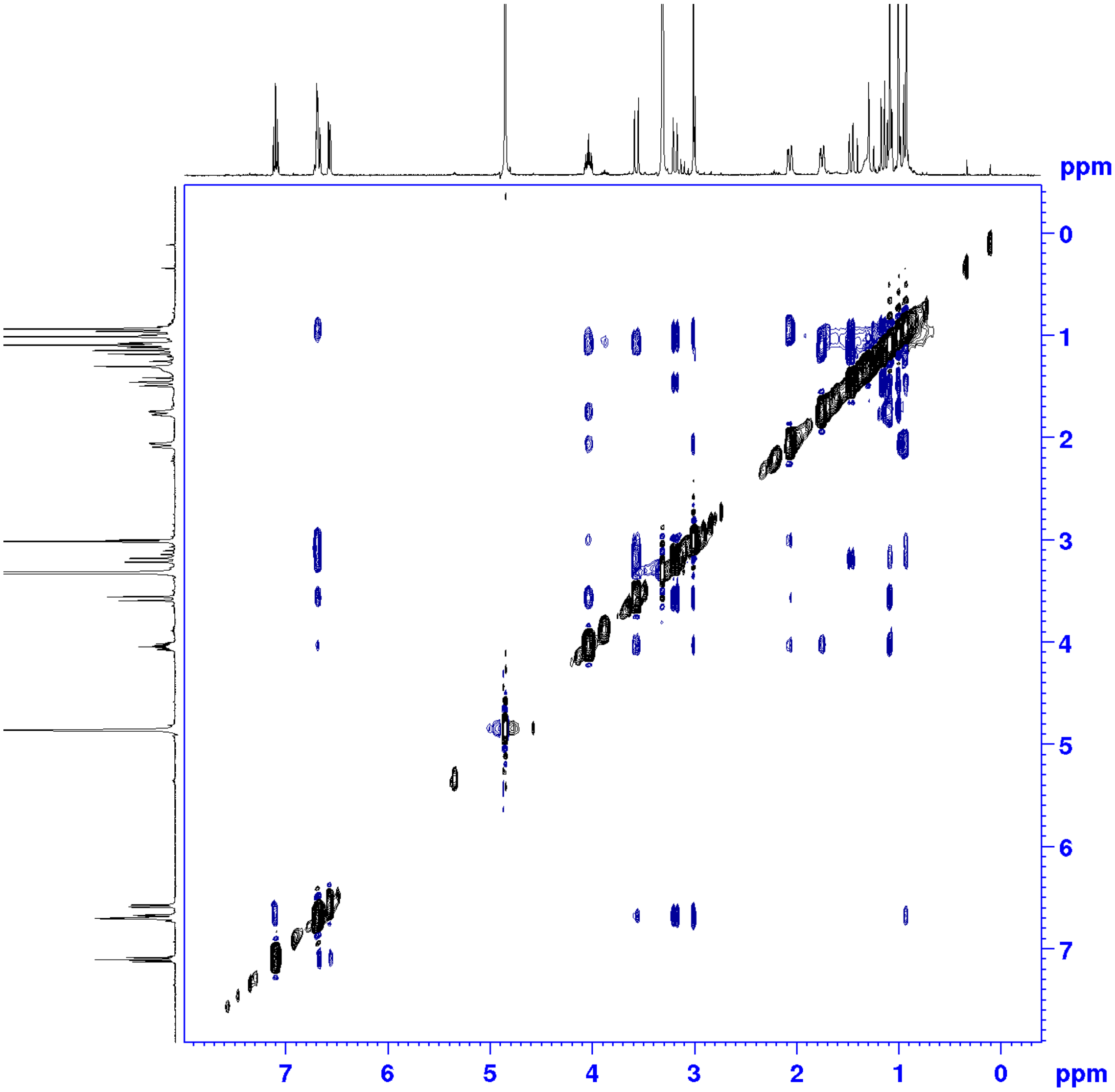

Supplementary Figure 47. $^{1}H^{1}H$ NOESY analysis of product **16** (Diagnostic nOe interactions)

Condition: HPLC (Daicel Chiralpak IC-3 column, 95:5 hexane/*i*PrOH, flow rate: 1.00 mL/min, λ = 254 nm)

Racemic sample 2a:

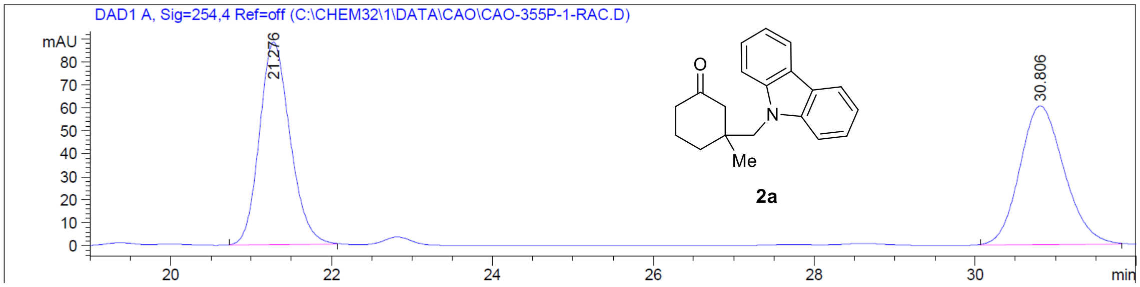

Signal 1: DAD1 A, Sig=254,4 Ref=off

Peak #	RetTime [min]	Type	Width [min]	Area [mAU*s]	Height [mAU]	Area %
1	21.276	BB	0.4072	2337.89917	88.22630	50.2017
2	30.806	BB	0.5918	2319.11108	60.42317	49.7983
Totals :				4657.01025	148.64947	

Enantioenriched sample 2a:

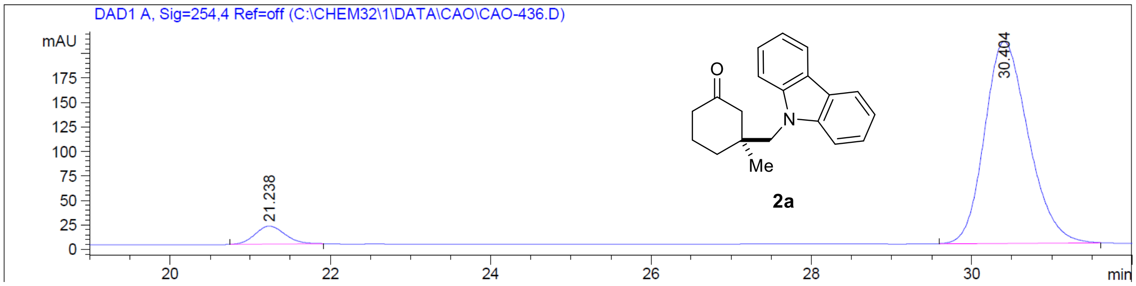

Signal 1: DAD1 A, Sig=254,4 Ref=off

Peak #	RetTime [min]	Type	Width [min]	Area [mAU*s]	Height [mAU]	Area %
1	21.238	BB	0.4063	491.93362	18.61522	5.9025
2	30.404	BB	0.5853	7842.43799	206.39705	94.0975
Totals :				8334.37161	225.01227	

Supplementary Figure 48. HPLC spectra for compound **2a**

Condition: HPLC (Daicel Chiralpak IC-3 column, 90:10 hexane/*i*PrOH, flow rate: 1.00 mL/min, λ = 254 nm)

Racemic sample 2b:

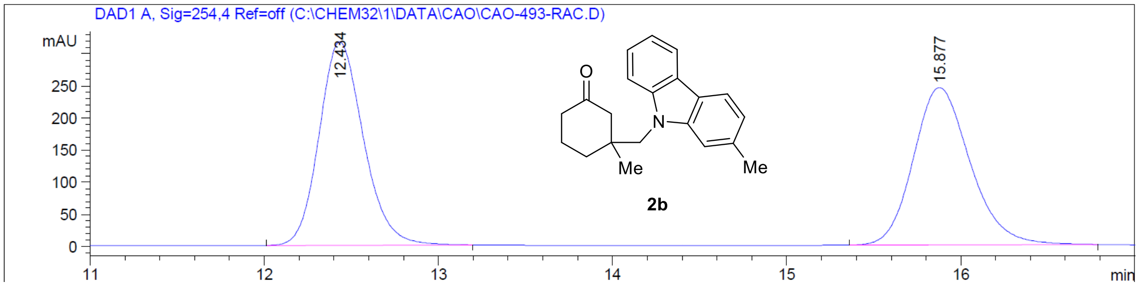

Signal 1: DAD1 A, Sig=254,4 Ref=off

Peak #	RetTime [min]	Type	Width [min]	Area [mAU*s]	Height [mAU]	Area %
1	12.434	BB	0.2724	5613.92090	317.36285	50.0993
2	15.877	BB	0.3499	5591.67773	245.35324	49.9007
Totals :				1.12056e4	562.71609	

Enantioenriched sample 2b:

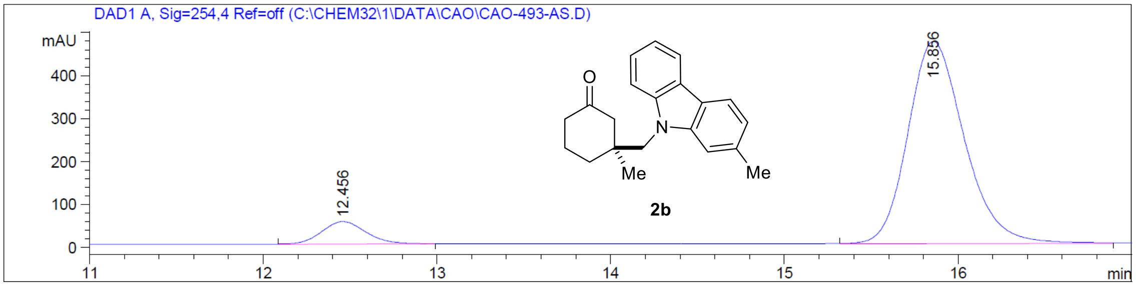

Signal 1: DAD1 A, Sig=254,4 Ref=off

Peak #	RetTime [min]	Type	Width [min]	Area [mAU*s]	Height [mAU]	Area %
1	12.456	BB	0.2765	935.13092	52.31479	8.0980
2	15.856	BB	0.3472	1.06125e4	470.43481	91.9020
Totals :				1.15476e4	522.74960	

Supplementary Figure 49. HPLC spectra for compound **2b**

Condition: HPLC (Daicel Chiralpak IC-3 column, 90:10 hexane/*i*PrOH, flow rate: 1.00 mL/min, λ = 254 nm)

Racemic sample 2c:

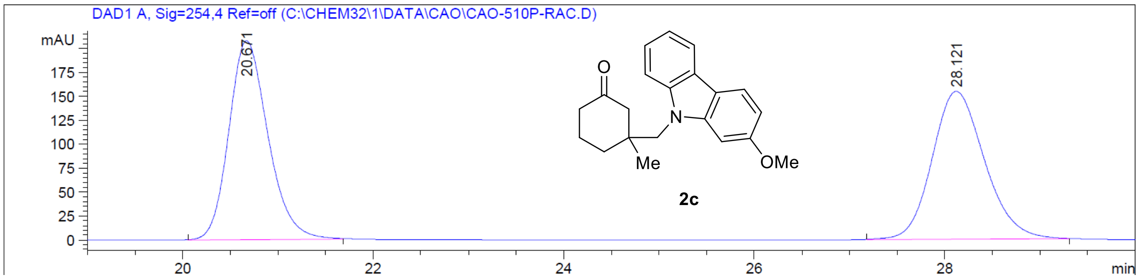

Signal 1: DAD1 A, Sig=254,4 Ref=off

Peak #	RetTime [min]	Type	Width [min]	Area [mAU*s]	Height [mAU]	Area %
1	20.671	BB	0.4383	5920.22314	207.85457	49.9584
2	28.121	BB	0.5913	5930.07568	154.66113	50.0416
Totals :				1.18503e4	362.51570	

Enantioenriched sample 2c:

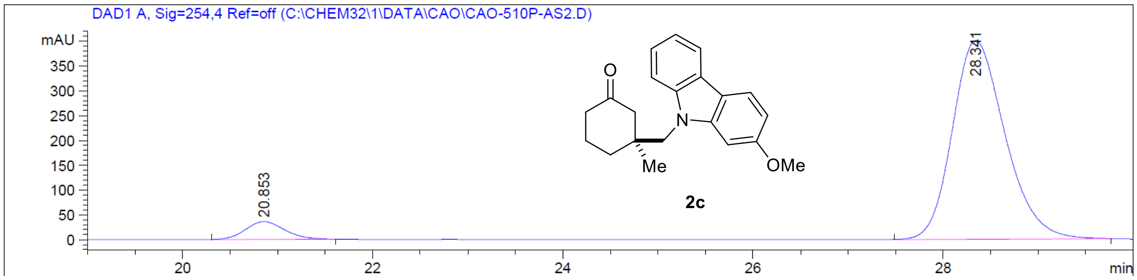

Signal 1: DAD1 A, Sig=254,4 Ref=off

Peak #	RetTime [min]	Type	Width [min]	Area [mAU*s]	Height [mAU]	Area %
1	20.853	BB	0.4430	1040.68933	36.02860	6.3133
2	28.341	BB	0.5917	1.54435e4	400.67889	93.6867
Totals :				1.64842e4	436.70750	

Supplementary Figure 50. HPLC spectra for compound **2c**

Condition: HPLC (Daicel Chiralpak IC-3 column, 90:10 hexane/*i*PrOH, flow rate: 1.00 mL/min, λ = 254 nm)

Racemic sample 2d:

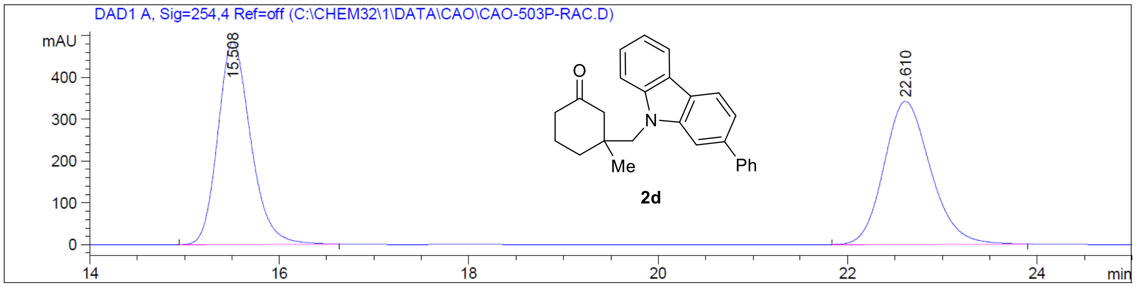

Signal 1: DAD1 A, Sig=254,4 Ref=off

Peak #	RetTime [min]	Type	Width [min]	Area [mAU*s]	Height [mAU]	Area %
1	15.508	BB	0.3695	1.16929e4	484.59494	49.9462
2	22.610	BB	0.5277	1.17180e4	342.06128	50.0538
Totals :				2.34109e4	826.65622	

Enantioenriched sample 2d:

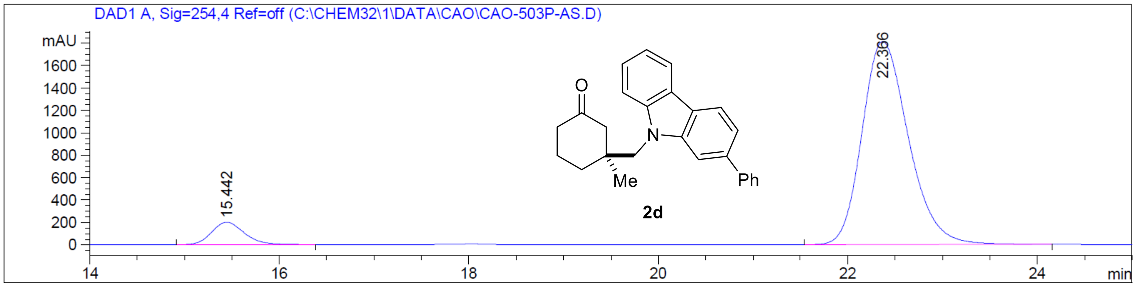

Signal 1: DAD1 A, Sig=254,4 Ref=off

Peak #	RetTime [min]	Type	Width [min]	Area [mAU*s]	Height [mAU]	Area %
1	15.442	BB	0.3695	4861.43994	201.44530	7.2977
2	22.366	BB	0.5256	6.17548e4	1812.27551	92.7023
Totals :				6.66162e4	2013.72081	

Supplementary Figure 51. HPLC spectra for compound **2d**

Condition: HPLC (Daicel Chiralpak IC-3 column, 90:10 hexane/*i*PrOH, flow rate: 1.00 mL/min, λ = 254 nm)

Racemic sample 2e:

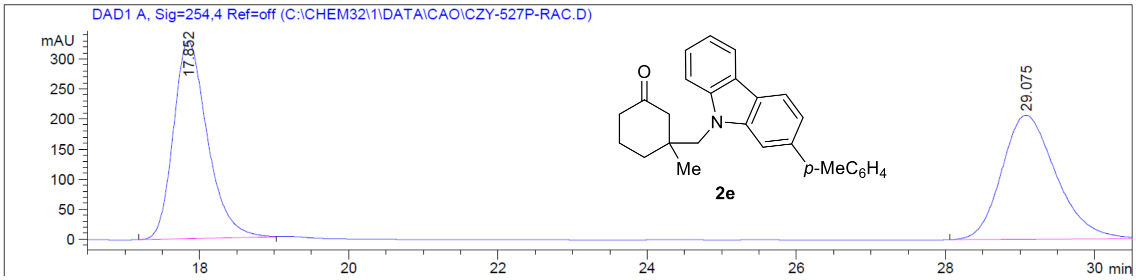

Signal 1: DAD1 A, Sig=254,4 Ref=off

Peak #	RetTime [min]	Type	Width [min]	Area [mAU*s]	Height [mAU]	Area %
1	17.852	BB	0.4826	1.03921e4	328.94534	49.7772
2	29.075	BB	0.7755	1.04851e4	206.79938	50.2228
Totals :				2.08772e4	535.74472	

Enantioenriched sample 2e:

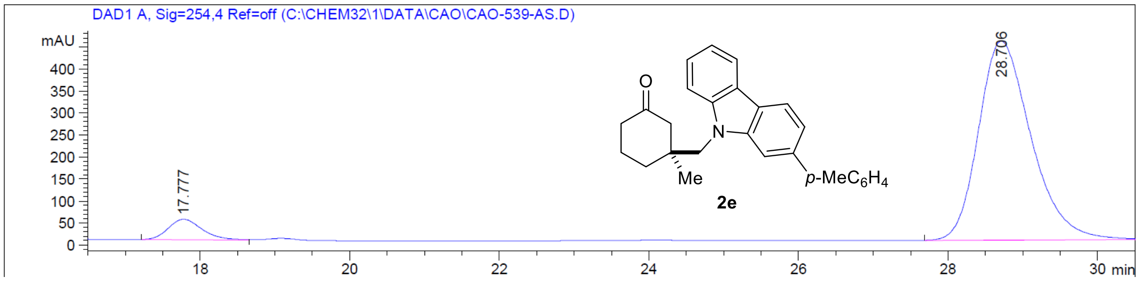

Signal 1: DAD1 A, Sig=254,4 Ref=off

Peak #	RetTime [min]	Type	Width [min]	Area [mAU*s]	Height [mAU]	Area %
1	17.777	BB	0.4843	1488.83105	46.90257	6.3231
2	28.706	BB	0.7471	2.20570e4	452.17953	93.6769
Totals :				2.35458e4	499.08210	

Supplementary Figure 52. HPLC spectra for compound **2e**

Condition: HPLC (Daicel Chiralpak IC-3 column, 90:10 hexane/*i*PrOH, flow rate: 1.00 mL/min, λ = 215 nm)

Racemic sample 2f:

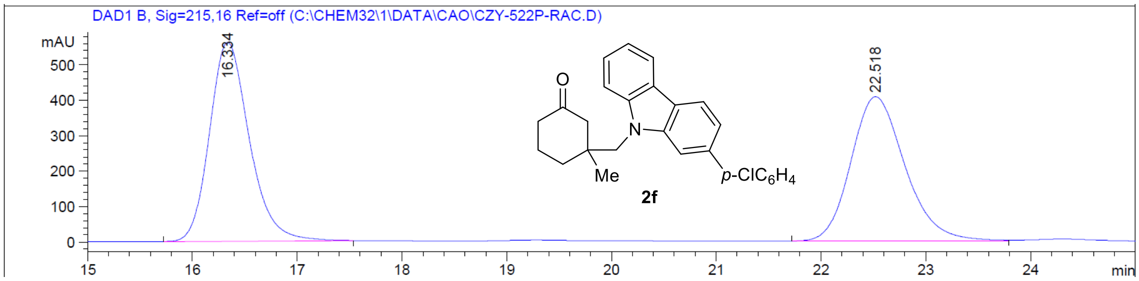

Signal 2: DAD1 B, Sig=215,16 Ref=off

Peak #	RetTime [min]	Type	Width [min]	Area [mAU*s]	Height [mAU]	Area %
1	16.334	BB	0.4062	1.48428e4	561.82562	50.2833
2	22.518	BB	0.5540	1.46755e4	407.69623	49.7167
Totals :				2.95183e4	969.52185	

Enantioenriched sample 2f:

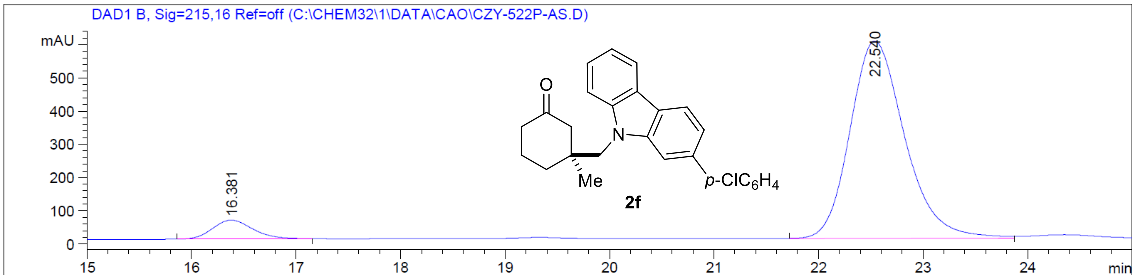

Signal 2: DAD1 B, Sig=215,16 Ref=off

Peak #	RetTime [min]	Type	Width [min]	Area [mAU*s]	Height [mAU]	Area %
1	16.381	BB	0.4149	1545.86853	57.28119	6.6782
2	22.540	BB	0.5560	2.16021e4	594.36090	93.3218
Totals :				2.31480e4	651.64209	

Supplementary Figure 53. HPLC spectra for compound **2f**

Condition: HPLC (Daicel Chiralpak IC-3 column, 90:10 hexane/*i*PrOH, flow rate: 1.00 mL/min, λ = 254 nm)

Racemic sample 2g:

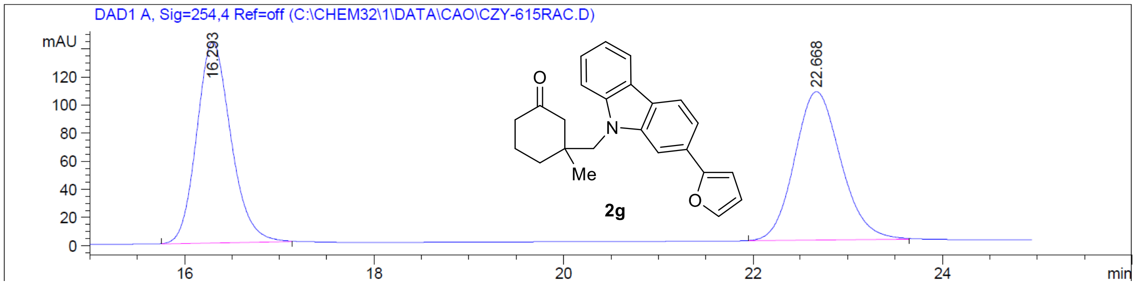

Signal 1: DAD1 A, Sig=254,4 Ref=off

Peak #	RetTime [min]	Type	Width [min]	Area [mAU*s]	Height [mAU]	Area %
1	16.293	BB	0.3869	3589.47803	142.99339	50.1061
2	22.668	BB	0.5233	3574.28174	105.48942	49.8939
Totals :				7163.75977	248.48281	

Enantioenriched sample 2g:

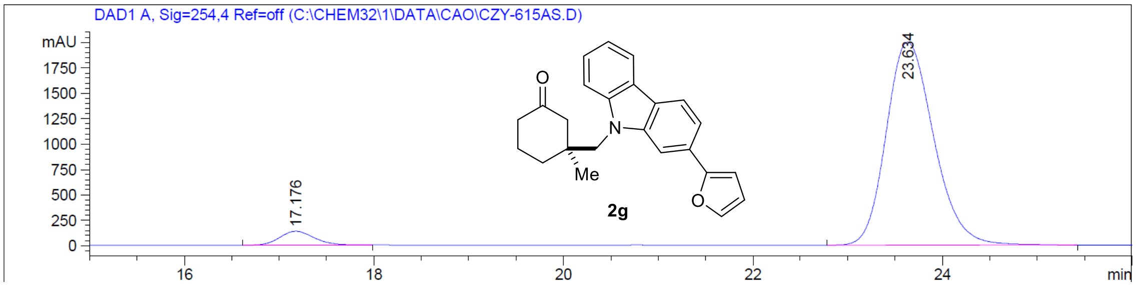

Signal 1: DAD1 A, Sig=254,4 Ref=off

Peak #	RetTime [min]	Type	Width [min]	Area [mAU*s]	Height [mAU]	Area %
1	17.176	BB	0.3909	3497.02588	137.43231	4.7850
2	23.634	BB	0.5425	6.95864e4	1997.29517	95.2150
Totals :				7.30834e4	2134.72748	

Supplementary Figure 54. HPLC spectra for compound **2g**

Condition: HPLC (Daicel Chiralpak IC-3 column, 90:10 hexane/*i*PrOH, flow rate: 1.00 mL/min, λ = 254 nm)

Racemic sample 2h:

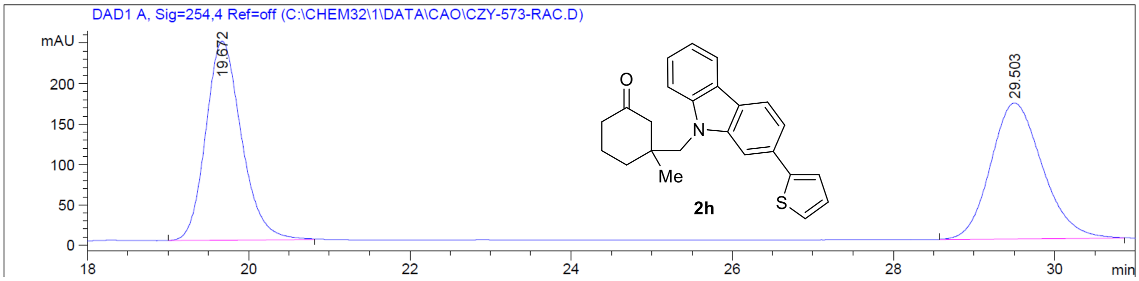

Signal 1: DAD1 A, Sig=254,4 Ref=off

Peak #	RetTime [min]	Type	Width [min]	Area [mAU*s]	Height [mAU]	Area %
1	19.672	BB	0.4722	7555.68164	246.11217	50.1735
2	29.503	BB	0.6888	7503.42480	168.09425	49.8265

Totals : 1.50591e4 414.20642

Enantioenriched sample 2h:

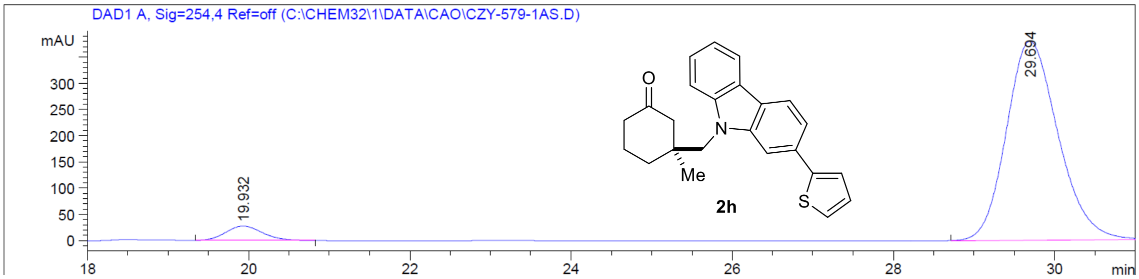

Signal 1: DAD1 A, Sig=254,4 Ref=off

Peak #	RetTime [min]	Type	Width [min]	Area [mAU*s]	Height [mAU]	Area %
1	19.932	BB	0.4930	877.72461	27.45457	4.9261
2	29.694	BB	0.6865	1.69402e4	379.67194	95.0739

Totals : 1.78179e4 407.12651

Supplementary Figure 55. HPLC spectra for compound **2h**

Condition: HPLC (Daicel Chiralpak IC-3 column, 90:10 hexane/*i*PrOH, flow rate: 1.00 mL/min, λ = 254 nm)

Racemic sample 2i:

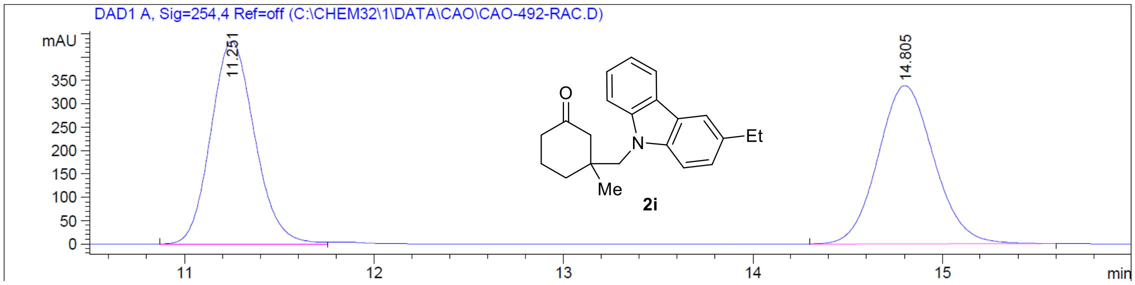

Signal 1: DAD1 A, Sig=254,4 Ref=off

Peak #	RetTime [min]	Type	Width [min]	Area [mAU*s]	Height [mAU]	Area %
1	11.251	BV	0.2495	6972.34033	433.91388	49.9675
2	14.805	BB	0.3182	6981.41211	339.12308	50.0325

Totals : 1.39538e4 773.03696

Enantioenriched sample 2i:

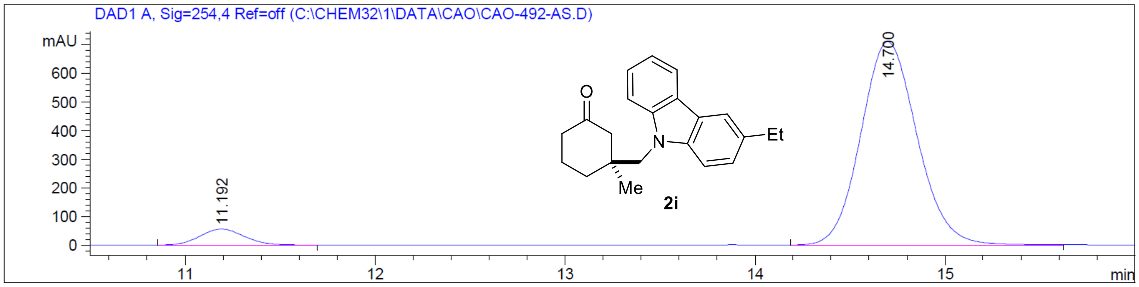

Signal 1: DAD1 A, Sig=254,4 Ref=off

Peak #	RetTime [min]	Type	Width [min]	Area [mAU*s]	Height [mAU]	Area %
1	11.192	BB	0.2548	919.95361	56.23726	5.9348
2	14.700	BB	0.3176	1.45811e4	709.97058	94.0652

Totals : 1.55011e4 766.20784

Supplementary Figure 56. HPLC spectra for compound **2i**

Condition: HPLC (Daicel Chiralpak IC-3 column, 90:10 hexane/*i*PrOH, flow rate: 1.00 mL/min, λ = 254 nm)

Racemic sample 2j:

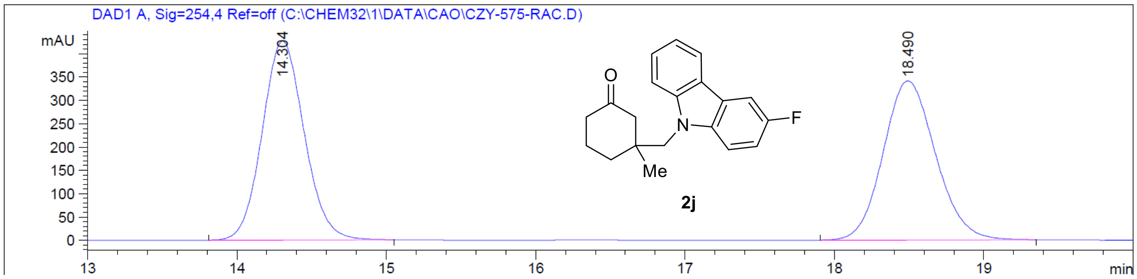

Signal 1: DAD1 A, Sig=254,4 Ref=off

Peak #	RetTime [min]	Type	Width [min]	Area [mAU*s]	Height [mAU]	Area %
1	14.304	BB	0.3021	8349.49512	427.04382	50.0603
2	18.490	BB	0.3786	8329.37793	341.45773	49.9397
Totals :				1.66789e4	768.50156	

Enantioenriched sample 2j:

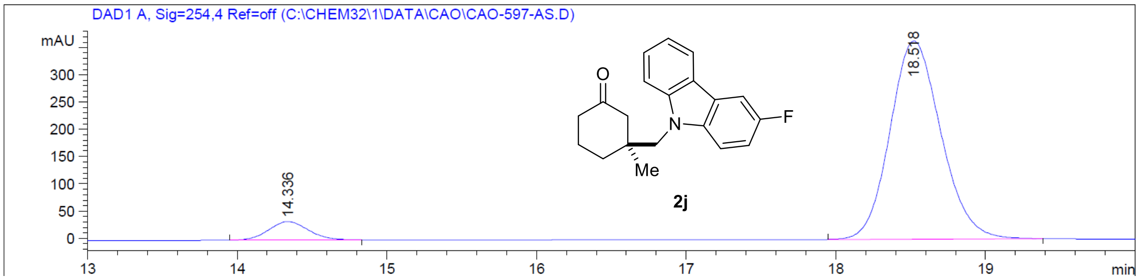

Signal 1: DAD1 A, Sig=254,4 Ref=off

Peak #	RetTime [min]	Type	Width [min]	Area [mAU*s]	Height [mAU]	Area %
1	14.336	BB	0.2872	633.24164	34.01905	6.8495
2	18.518	BB	0.3661	8611.88965	363.88864	93.1505
Totals :				9245.13129	397.90769	

Supplementary Figure 57. HPLC spectra for compound **2j**

Condition: HPLC (Daicel Chiralpak IC-3 column, 90:10 hexane/*i*PrOH, flow rate: 1.00 mL/min, λ = 254 nm)

Racemic sample 2k:

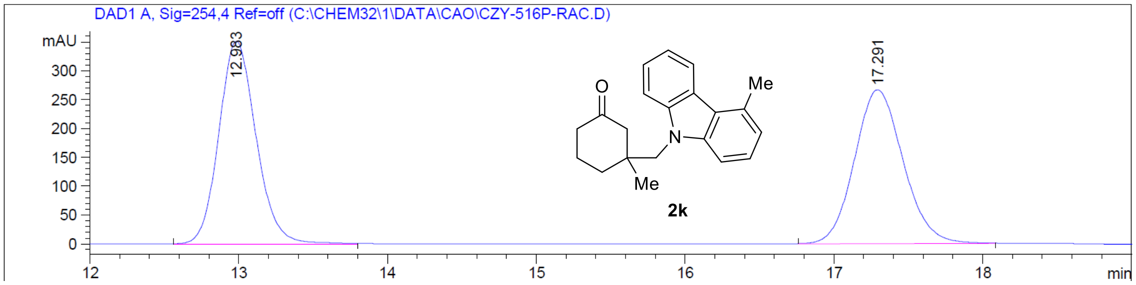

Signal 1: DAD1 A, Sig=254,4 Ref=off

Peak #	RetTime [min]	Type	Width [min]	Area [mAU*s]	Height [mAU]	Area %
1	12.983	BB	0.2666	6103.65234	351.56015	50.1745
2	17.291	BB	0.3512	6061.20898	266.68472	49.8255
Totals :				1.21649e4	618.24487	

Enantioenriched sample 2k:

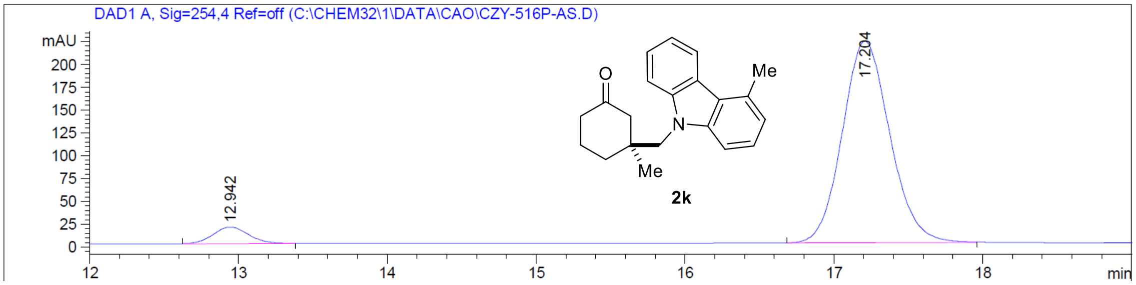

Signal 1: DAD1 A, Sig=254,4 Ref=off

Peak #	RetTime [min]	Type	Width [min]	Area [mAU*s]	Height [mAU]	Area %
1	12.942	BB	0.2586	305.01288	18.28305	5.8567
2	17.204	BB	0.3430	4902.91895	220.84532	94.1433
Totals :				5207.93182	239.12837	

Supplementary Figure 58. HPLC spectra for compound **2k**

Condition: HPLC (Daicel Chiralpak IC-3 column, 90:10 hexane/*i*PrOH, flow rate: 1.00 mL/min, λ = 254 nm)

Racemic sample 2l:

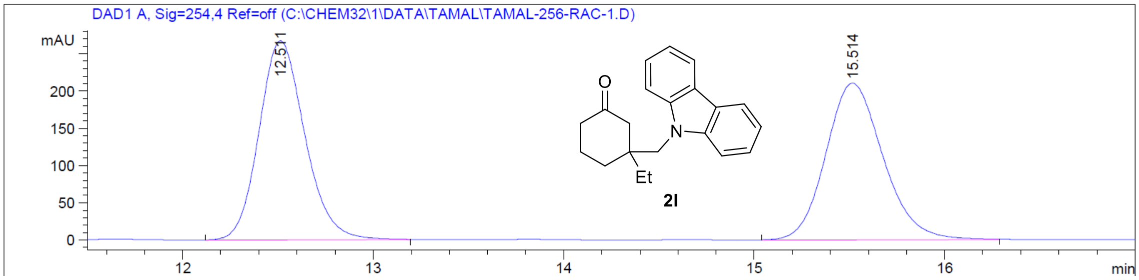

Signal 1: DAD1 A, Sig=254,4 Ref=off

Peak #	RetTime [min]	Type	Width [min]	Area [mAU*s]	Height [mAU]	Area %
1	12.511	BB	0.2569	4460.25439	267.04309	50.4417
2	15.514	BB	0.3215	4382.14355	209.93507	49.5583
Totals :				8842.39795	476.97816	

Enantioenriched sample 2l:

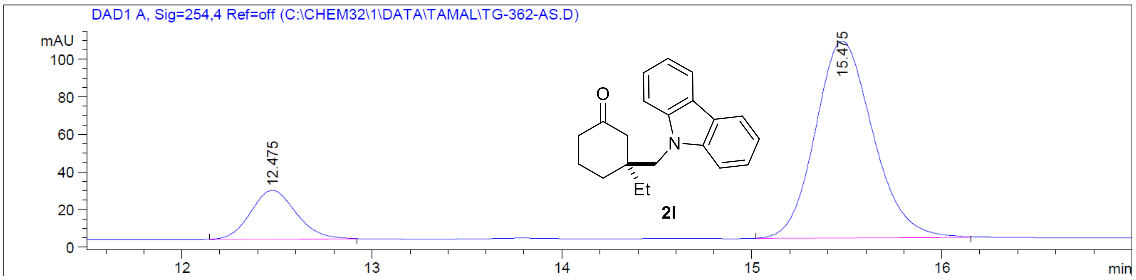

Signal 1: DAD1 A, Sig=254,4 Ref=off

Peak #	RetTime [min]	Type	Width [min]	Area [mAU*s]	Height [mAU]	Area %
1	12.475	BB	0.2571	436.55951	26.10285	16.5171
2	15.475	BB	0.3233	2206.52075	104.94261	83.4829
Totals :				2643.08026	131.04546	

Supplementary Figure 59. HPLC spectra for compound **2l**

Condition: HPLC (Daicel Chiralpak IC-3 column, 90:10 hexane/*i*PrOH, flow rate: 1.00 mL/min, λ = 254 nm)

Racemic sample 2m:

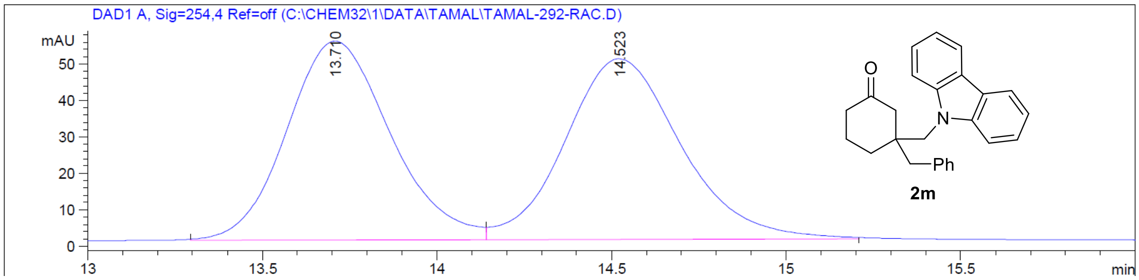

Signal 1: DAD1 A, Sig=254,4 Ref=off

Peak #	RetTime [min]	Type	Width [min]	Area [mAU*s]	Height [mAU]	Area %
1	13.710	BV	0.3192	1129.25830	54.62945	49.5546
2	14.523	VB	0.3544	1149.55847	49.60733	50.4454
Totals :				2278.81677	104.23678	

Enantioenriched sample 2m:

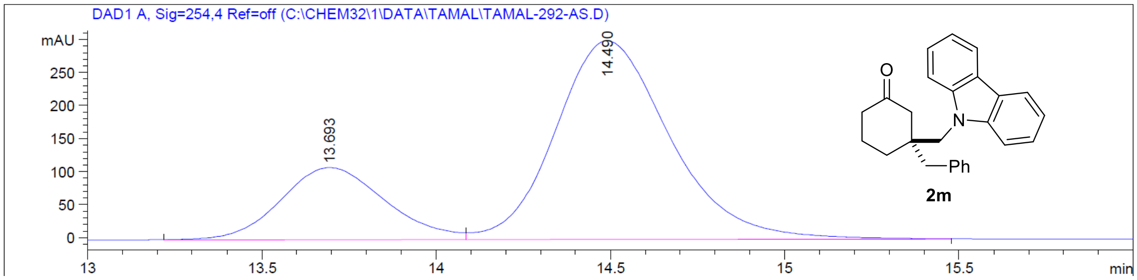

Signal 1: DAD1 A, Sig=254,4 Ref=off

Peak #	RetTime [min]	Type	Width [min]	Area [mAU*s]	Height [mAU]	Area %
1	13.693	BV	0.3223	2286.58496	109.21271	24.8951
2	14.490	VB	0.3517	6898.29297	300.60345	75.1049
Totals :				9184.87793	409.81616	

Supplementary Figure 60. HPLC spectra for compound **2m**

Condition: HPLC (Daicel Chiralpak IC-3 column, 90:10 hexane/*i*PrOH, flow rate: 1.00 mL/min, λ = 254 nm)

Racemic sample 2n:

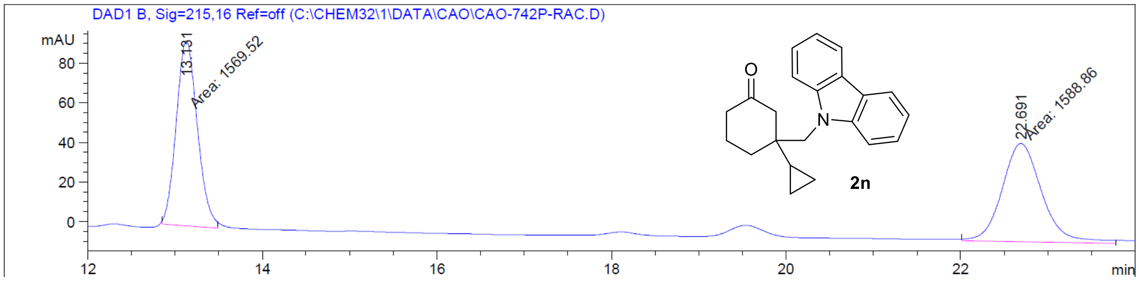

Signal 2: DAD1 B, Sig=215,16 Ref=off

Peak #	RetTime [min]	Type	Width [min]	Area [mAU*s]	Height [mAU]	Area %
1	13.131	MM	0.2797	1569.52222	93.52643	49.6939
2	22.691	MM	0.5323	1588.85950	49.74448	50.3061
Totals :				3158.38171	143.27090	

Enantioenriched sample 2n:

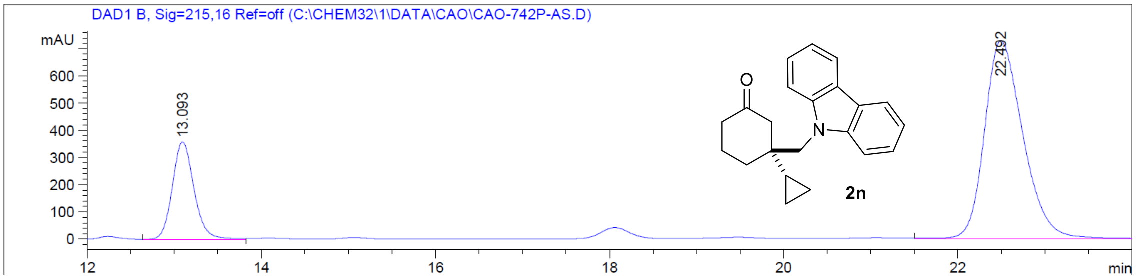

Signal 2: DAD1 B, Sig=215,16 Ref=off

Peak #	RetTime [min]	Type	Width [min]	Area [mAU*s]	Height [mAU]	Area %
1	13.093	VB	0.2657	6194.79492	358.29019	21.8159
2	22.492	VB	0.4725	2.22010e4	726.51312	78.1841
Totals :				2.83958e4	1084.80331	

Supplementary Figure 61. HPLC spectra for compound **2n**

Condition: HPLC (Daicel Chiralpak ID-3 column, 90:10 hexane/*i*PrOH, flow rate: 1.00 mL/min, λ = 254 nm)

Racemic sample 2o:

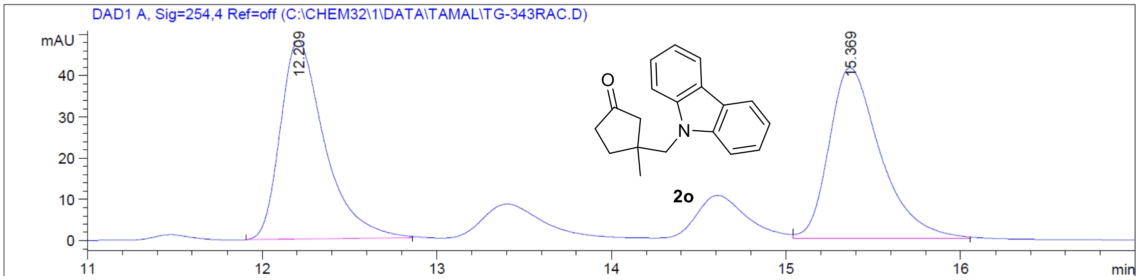

Signal 1: DAD1 A, Sig=254,4 Ref=off

Peak #	RetTime [min]	Type	Width [min]	Area [mAU*s]	Height [mAU]	Area %
1	12.209	BB	0.2655	846.99664	48.08387	50.1375
2	15.369	VB	0.3079	842.35028	41.30038	49.8625
Totals :				1689.34692	89.38425	

Enantioenriched sample 2o:

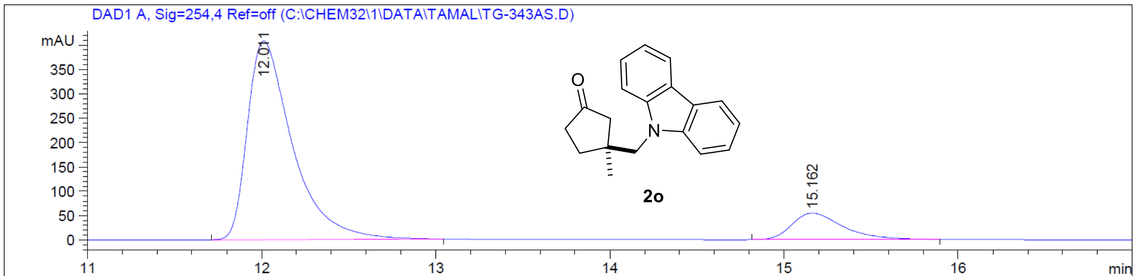

Signal 1: DAD1 A, Sig=254,4 Ref=off

Peak #	RetTime [min]	Type	Width [min]	Area [mAU*s]	Height [mAU]	Area %
1	12.011	BB	0.2698	7347.42188	408.48520	86.8085
2	15.162	BB	0.3079	1116.51660	54.75738	13.1915
Totals :				8463.93848	463.24258	

Supplementary Figure 62. HPLC spectra for compound **2o**

Condition: HPLC (Daicel Chiralpak IC-3 column, 90:10 hexane/*i*PrOH, flow rate: 1.00 mL/min, λ = 254 nm)

Racemic sample 2p:

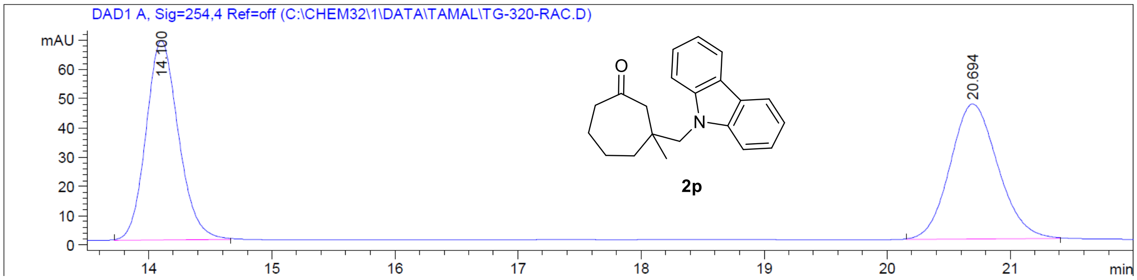

Signal 1: DAD1 A, Sig=254,4 Ref=off

Peak #	RetTime [min]	Type	Width [min]	Area [mAU*s]	Height [mAU]	Area %
1	14.100	BB	0.2815	1245.83081	68.07520	49.9693
2	20.694	BB	0.4172	1247.36206	46.17547	50.0307

Totals : 2493.19287 114.25067

Enantioenriched sample 2p:

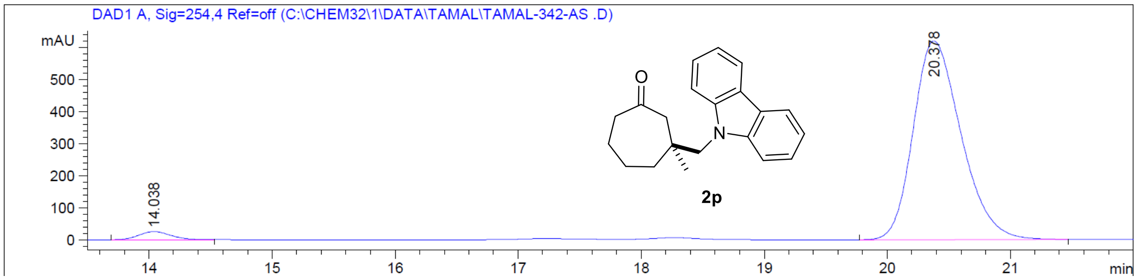

Signal 1: DAD1 A, Sig=254,4 Ref=off

Peak #	RetTime [min]	Type	Width [min]	Area [mAU*s]	Height [mAU]	Area %
1	14.038	BB	0.2800	466.03348	25.64517	2.6992
2	20.378	BB	0.4155	1.67993e4	621.22626	97.3008

Totals : 1.72653e4 646.87143

Supplementary Figure 63. HPLC spectra for compound **2p**

Condition: HPLC (Daicel Chiralpak IC-3 column, 90:10 hexane/*i*PrOH, flow rate: 1.00 mL/min, λ = 254 nm)

Racemic sample 2q:

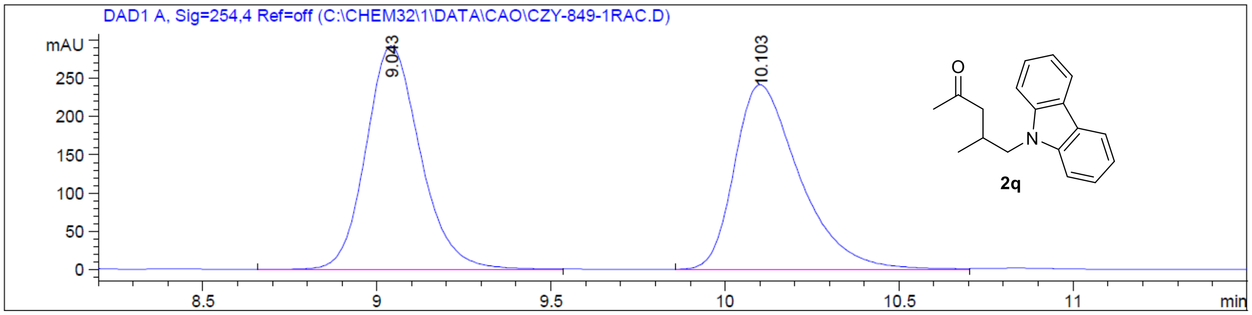

Signal 1: DAD1 A, Sig=254,4 Ref=off

Peak #	RetTime [min]	Type	Width [min]	Area [mAU*s]	Height [mAU]	Area %
1	9.043	VB	0.1667	3176.43896	292.41522	50.2434
2	10.103	BB	0.1960	3145.66284	241.48729	49.7566
Totals :				6322.10181	533.90251	

Enantioenriched sample 2q:

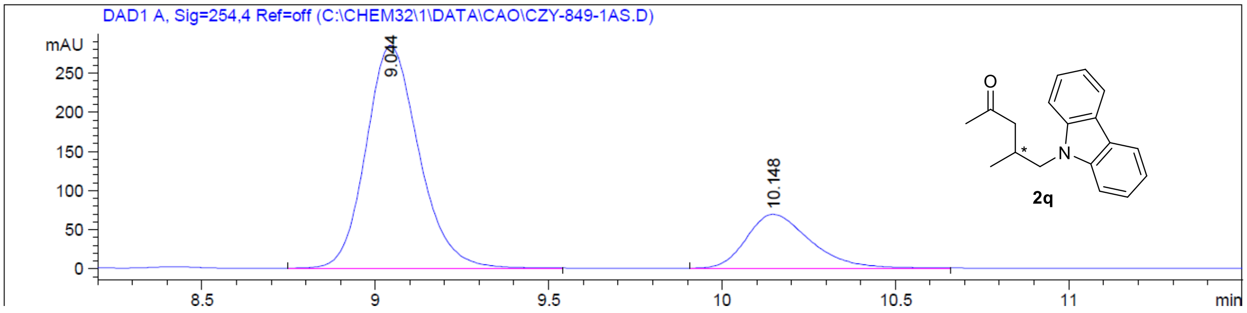

Signal 1: DAD1 A, Sig=254,4 Ref=off

Peak #	RetTime [min]	Type	Width [min]	Area [mAU*s]	Height [mAU]	Area %
1	9.044	BB	0.1640	3025.33545	284.66684	77.4145
2	10.148	BB	0.1955	882.63629	68.89301	22.5855
Totals :				3907.97174	353.55984	

Supplementary Figure 64. HPLC spectra for compound **2q**

Condition: HPLC (Daicel Chiralpak IC-3 column, 90:10 hexane/*i*PrOH, flow rate: 1.00 mL/min, λ = 215 nm)

Racemic sample 2r:

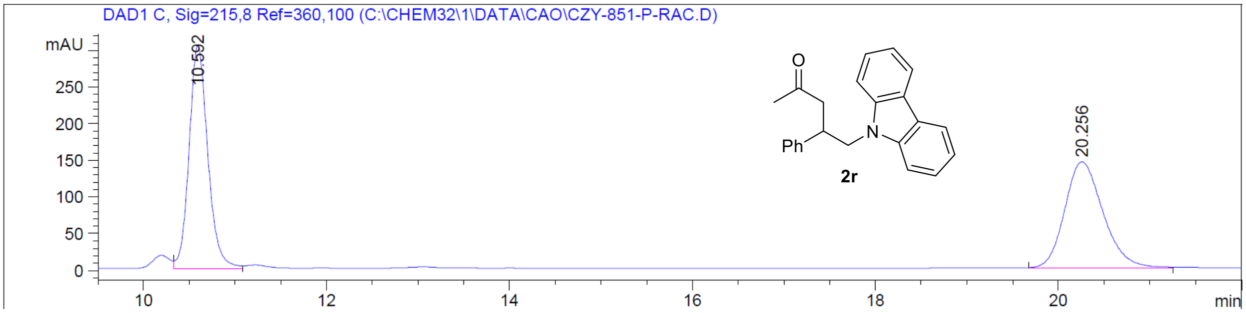

Signal 3: DAD1 C, Sig=215,8 Ref=360,100

Peak #	RetTime [min]	Type	Width [min]	Area [mAU*s]	Height [mAU]	Area %
1	10.592	VV	0.2155	4320.49512	304.50745	50.6765
2	20.256	BB	0.4469	4205.14062	144.76852	49.3235
Totals :				8525.63574	449.27597	

Enantioenriched sample 2r:

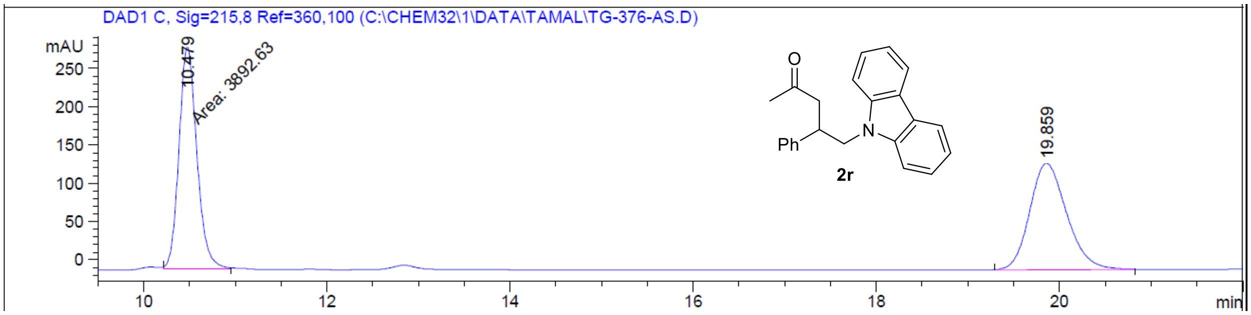

Signal 3: DAD1 C, Sig=215,8 Ref=360,100

Peak #	RetTime [min]	Type	Width [min]	Area [mAU*s]	Height [mAU]	Area %
1	10.479	MM	0.2246	3892.63135	288.90680	49.8966
2	19.859	BB	0.4316	3908.76855	139.21344	50.1034
Totals :				7801.39990	428.12024	

Supplementary Figure 65. HPLC spectra for compound **2r**

Condition: HPLC (Daicel Chiralpak IC-3 column, 90:10 hexane/*i*PrOH, flow rate: 1.00 mL/min, λ = 254 nm)

Racemic sample 10a:

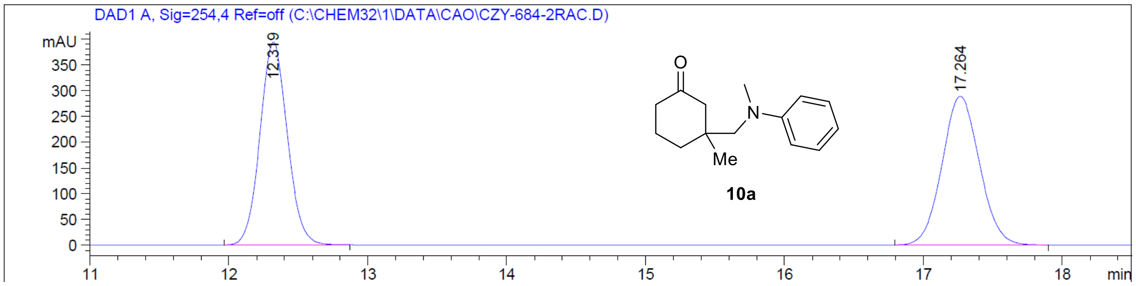

Signal 1: DAD1 A, Sig=254,4 Ref=off

Peak #	RetTime [min]	Type	Width [min]	Area [mAU*s]	Height [mAU]	Area %
1	12.319	BB	0.2142	5410.11133	393.93042	49.9329
2	17.264	BB	0.2910	5424.64746	288.92307	50.0671
Totals :				1.08348e4	682.85349	

Enantioenriched sample 10a:

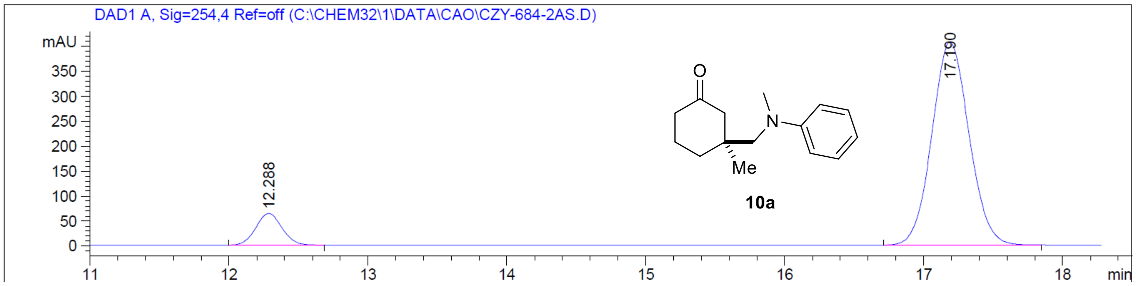

Signal 1: DAD1 A, Sig=254,4 Ref=off

Peak #	RetTime [min]	Type	Width [min]	Area [mAU*s]	Height [mAU]	Area %
1	12.288	BB	0.2038	839.46582	63.69313	9.9694
2	17.190	BB	0.2892	7580.99023	407.19931	90.0306
Totals :				8420.45605	470.89244	

Supplementary Figure 66. HPLC spectra for compound **10a**

Condition: HPLC (Daicel Chiralpak IC-3 column, 95:5 hexane/*i*PrOH, flow rate: 1.00 mL/min, λ = 254 nm)

Racemic sample 10b:

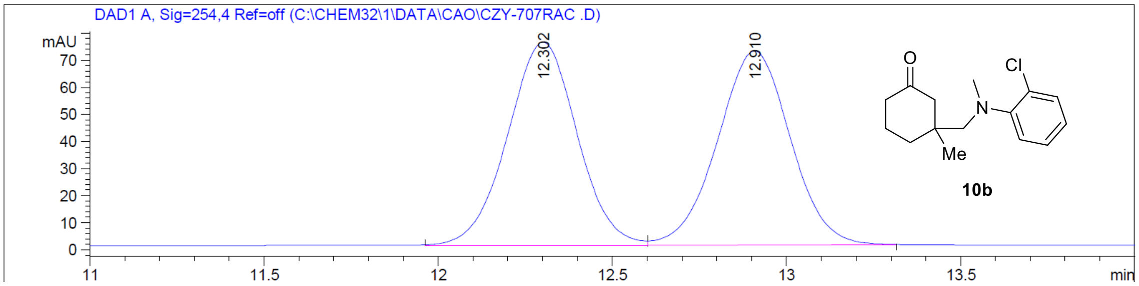

Signal 1: DAD1 A, Sig=254,4 Ref=off

Peak #	RetTime [min]	Type	Width [min]	Area [mAU*s]	Height [mAU]	Area %
1	12.302	BV	0.2160	1041.71069	75.03359	49.9322
2	12.910	VB	0.2261	1044.53784	71.63832	50.0678
Totals :				2086.24854	146.67191	

Enantioenriched sample 10b:

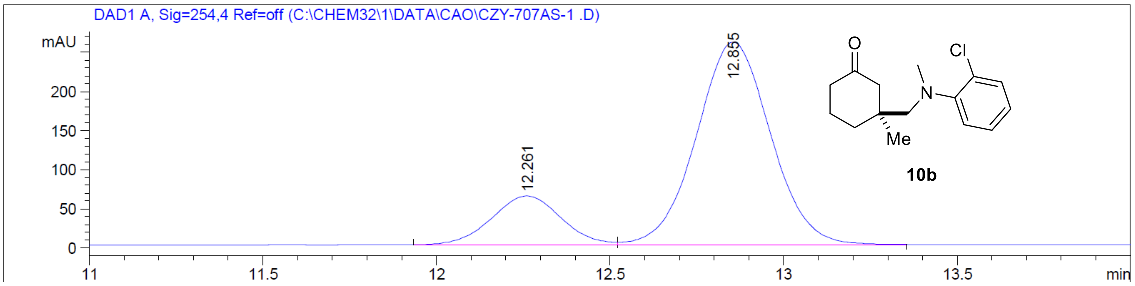

Signal 1: DAD1 A, Sig=254,4 Ref=off

Peak #	RetTime [min]	Type	Width [min]	Area [mAU*s]	Height [mAU]	Area %
1	12.261	BV	0.2160	867.07813	62.43952	18.4054
2	12.855	VB	0.2293	3843.91089	258.77542	81.5946
Totals :				4710.98901	321.21494	

Supplementary Figure 67. HPLC spectra for compound **10b**

Condition: HPLC (Daicel Chiralpak IC-3 column, 90:10 hexane/*i*PrOH, flow rate: 1.00 mL/min, λ = 254 nm)

Racemic sample 10c:

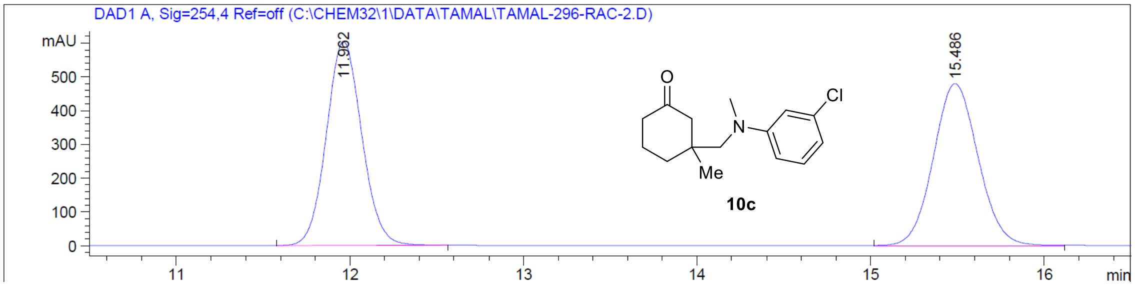

Signal 1: DAD1 A, Sig=254,4 Ref=off

Peak #	RetTime [min]	Type	Width [min]	Area [mAU*s]	Height [mAU]	Area %
1	11.962	BB	0.2215	8570.57422	604.05499	50.0217
2	15.486	BB	0.2786	8563.12891	478.90897	49.9783
Totals :				1.71337e4	1082.96396	

Enantioenriched sample 10c:

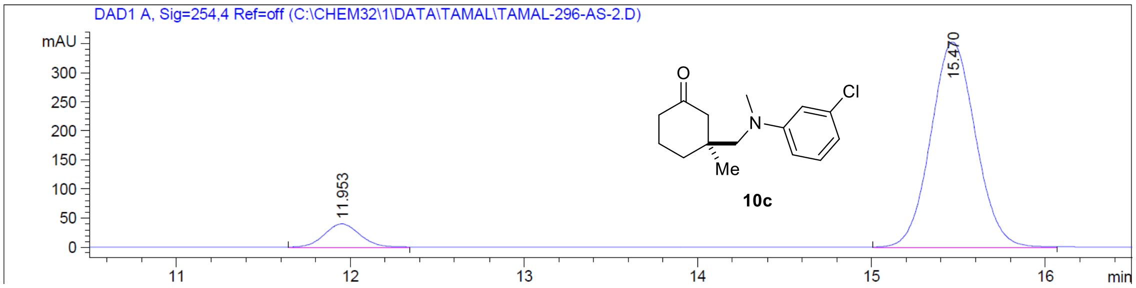

Signal 1: DAD1 A, Sig=254,4 Ref=off

Peak #	RetTime [min]	Type	Width [min]	Area [mAU*s]	Height [mAU]	Area %
1	11.953	BB	0.2212	566.16919	39.97834	8.2499
2	15.470	BB	0.2783	6296.59180	352.74298	91.7501
Totals :				6862.76099	392.72132	

Supplementary Figure 68. HPLC spectra for compound **10c**

Condition: HPLC (Daicel Chiralpak IC-3 column, 90:10 hexane/*i*PrOH, flow rate: 1.00 mL/min, λ = 254 nm)

Racemic sample 10d:

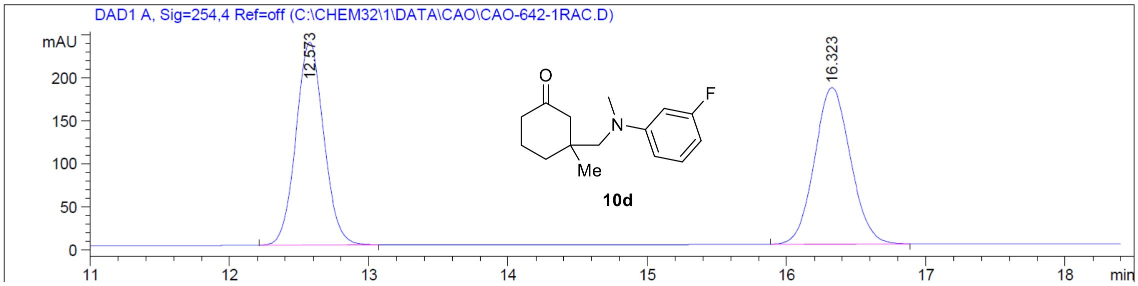

Signal 1: DAD1 A, Sig=254,4 Ref=off

Peak #	RetTime [min]	Type	Width [min]	Area [mAU*s]	Height [mAU]	Area %
1	12.573	BB	0.2145	3287.36304	236.08261	50.0839
2	16.323	BB	0.2800	3276.34668	182.04158	49.9161

Totals : 6563.70972 418.12419

Enantioenriched sample 10d:

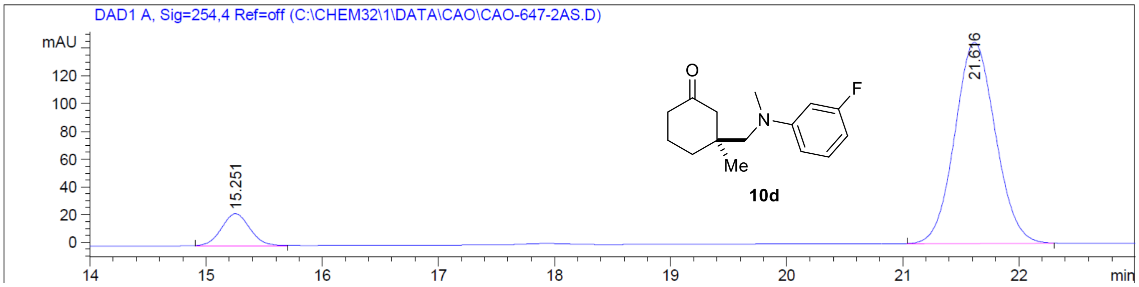

Signal 1: DAD1 A, Sig=254,4 Ref=360,100

Peak #	RetTime [min]	Type	Width [min]	Area [mAU*s]	Height [mAU]	Area %
1	12.833	BB	0.2257	3436.17041	233.41516	10.6497
2	16.624	BB	0.2988	2.88292e4	1496.39490	89.3503

Totals : 3.22653e4 1729.81006

Supplementary Figure 69. HPLC spectra for compound **10d**

Condition: HPLC (Daicel Chiralpak IC-3 column, 85:15 hexane/*i*PrOH, flow rate: 1.00 mL/min, λ = 254 nm)

Racemic sample 10e:

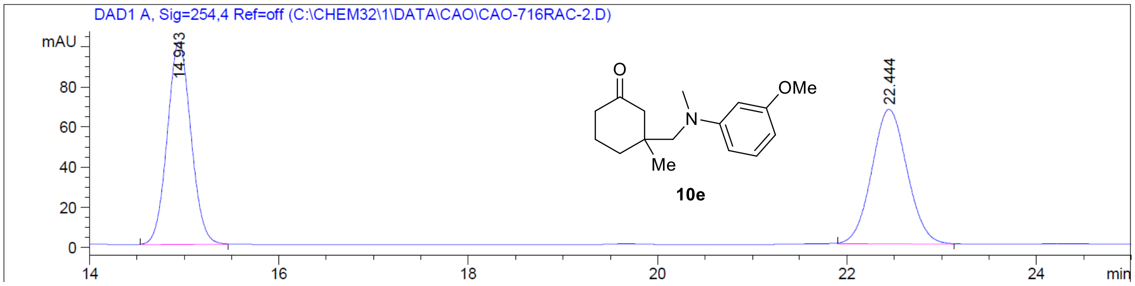

Signal 1: DAD1 A, Sig=254,4 Ref=off

Peak #	RetTime [min]	Type	Width [min]	Area [mAU*s]	Height [mAU]	Area %
1	14.943	BB	0.2703	1748.85840	100.84283	50.2020
2	22.444	BB	0.4026	1734.78662	66.88544	49.7980
Totals :				3483.64502	167.72827	

Enantioenriched sample 10e:

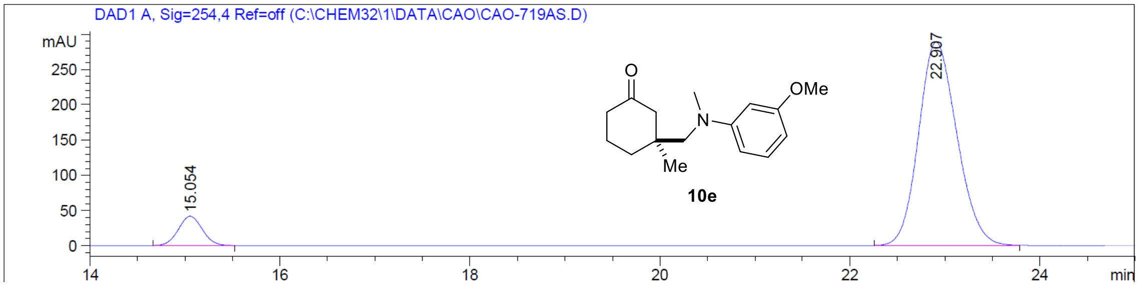

Signal 1: DAD1 A, Sig=254,4 Ref=off

Peak #	RetTime [min]	Type	Width [min]	Area [mAU*s]	Height [mAU]	Area %
1	15.054	BB	0.2724	737.66089	41.69281	8.3603
2	22.907	BB	0.4328	8085.70703	288.64108	91.6397
Totals :				8823.36792	330.33389	

Supplementary Figure 70. HPLC spectra for compound **10e**

Condition: HPLC (Daicel Chiralpak IC-3 column, 90:10 hexane/*i*PrOH, flow rate: 1.00 mL/min, λ = 254 nm)

Racemic sample 10f:

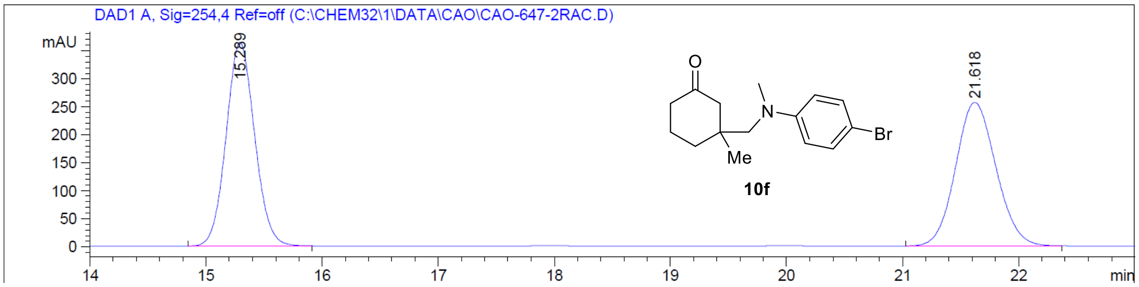

Signal 1: DAD1 A, Sig=254,4 Ref=off

Peak #	RetTime [min]	Type	Width [min]	Area [mAU*s]	Height [mAU]	Area %
1	15.289	BB	0.2657	6233.47656	364.25131	49.9975
2	21.618	BB	0.3751	6234.10938	256.93427	50.0025

Totals : 1.24676e4 621.18558

Enantioenriched sample 10f:

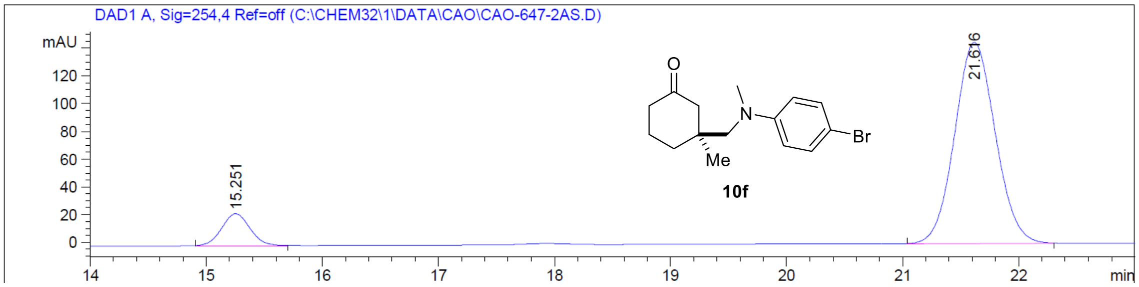

Signal 1: DAD1 A, Sig=254,4 Ref=off

Peak #	RetTime [min]	Type	Width [min]	Area [mAU*s]	Height [mAU]	Area %
1	15.251	BB	0.2614	390.80927	23.09367	9.9518
2	21.616	BB	0.3779	3536.20215	145.29626	90.0482

Totals : 3927.01141 168.38993

Supplementary Figure 71. HPLC spectra for compound **10f**

Condition: HPLC (Daicel Chiralpak IC-3 column, 90:10 hexane/*i*PrOH, flow rate: 1.00 mL/min, λ = 254 nm)

Racemic sample 10g:

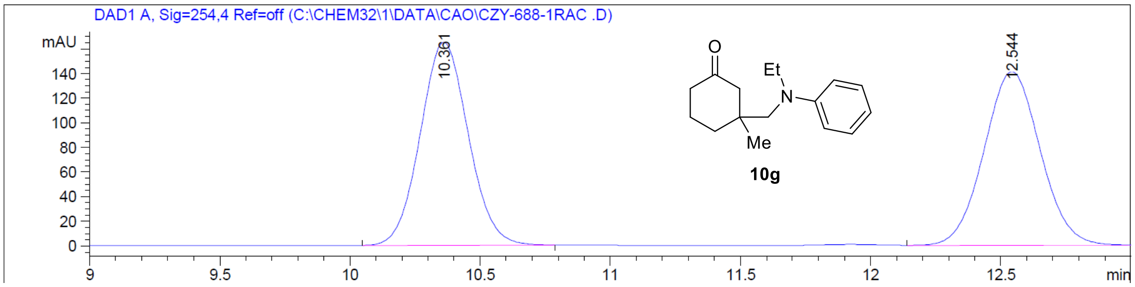

Signal 1: DAD1 A, Sig=254,4 Ref=off

Peak #	RetTime [min]	Type	Width [min]	Area [mAU*s]	Height [mAU]	Area %
1	10.361	BB	0.2000	2122.39941	165.03960	50.0063
2	12.544	VB	0.2334	2121.86743	141.13148	49.9937
Totals :				4244.26685	306.17108	

Enantioenriched sample 10g:

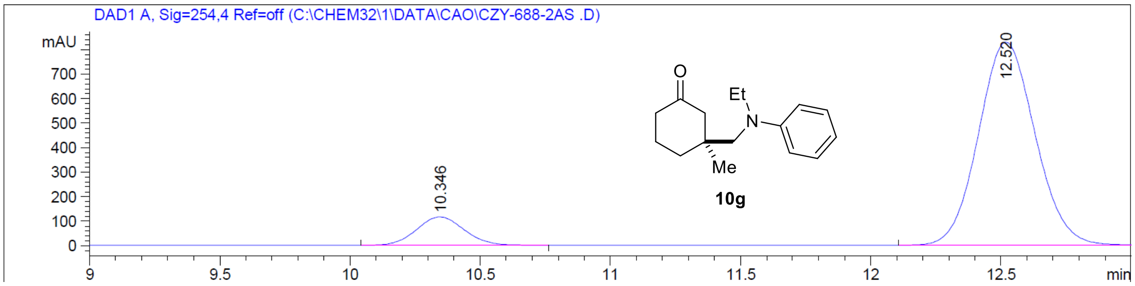

Signal 1: DAD1 A, Sig=254,4 Ref=off

Peak #	RetTime [min]	Type	Width [min]	Area [mAU*s]	Height [mAU]	Area %
1	10.346	BB	0.1994	1486.55383	116.12269	10.5908
2	12.520	BB	0.2347	1.25498e4	828.79462	89.4092
Totals :				1.40363e4	944.91730	

Supplementary Figure 72. HPLC spectra for compound **10g**

Condition: HPLC (Daicel Chiralpak IC-3 column, 90:10 hexane/*i*PrOH, flow rate: 1.00 mL/min, $\lambda = 254$ nm)

Racemic sample 10h:

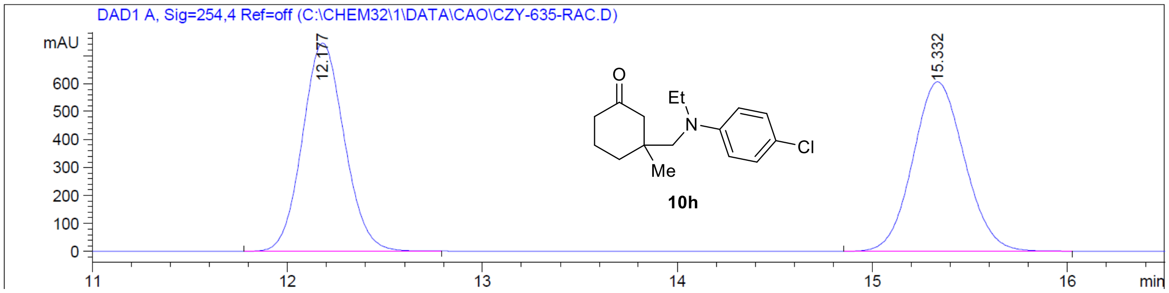

Signal 1: DAD1 A, Sig=254,4 Ref=off

Peak #	RetTime [min]	Type	Width [min]	Area [mAU*s]	Height [mAU]	Area %
1	12.177	BB	0.2324	1.11246e4	744.39746	49.9557
2	15.332	BB	0.2866	1.11443e4	605.86829	50.0443
Totals :				2.22688e4	1350.26575	

Enantioenriched sample 10h:

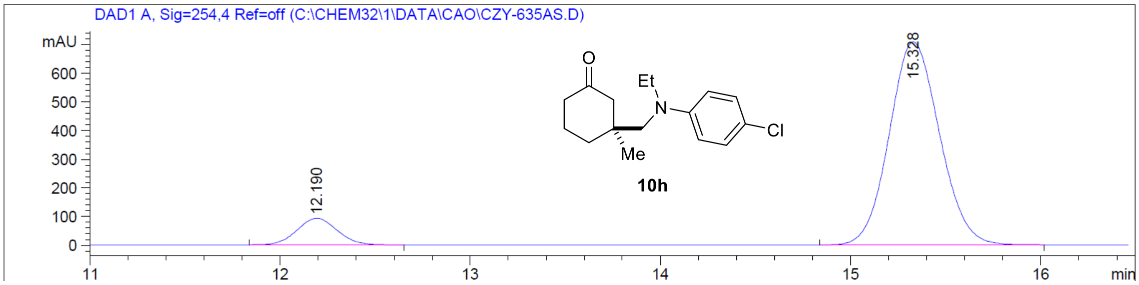

Signal 1: DAD1 A, Sig=254,4 Ref=off

Peak #	RetTime [min]	Type	Width [min]	Area [mAU*s]	Height [mAU]	Area %
1	12.190	BB	0.2292	1381.30042	93.03397	9.5818
2	15.328	BB	0.2867	1.30345e4	708.31348	90.4182
Totals :				1.44158e4	801.34745	

Supplementary Figure 73. HPLC spectra for compound **10h**

Condition: HPLC (Daicel Chiralpak IC-3 column, 90:10 hexane/*i*PrOH, flow rate: 1.00 mL/min, λ = 254 nm)

Racemic sample 10i:

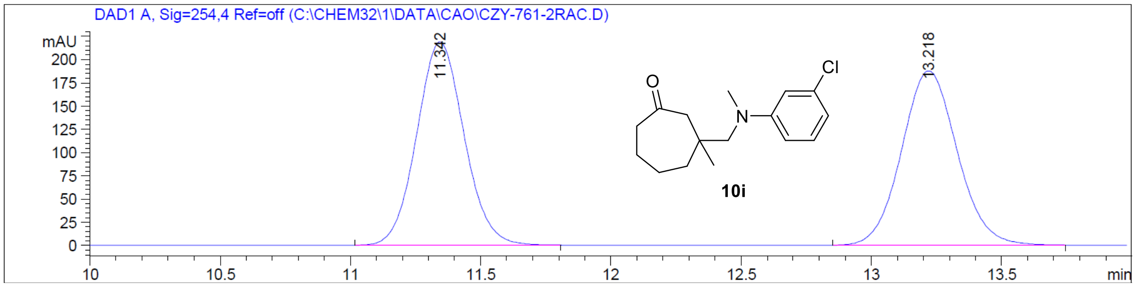

Signal 1: DAD1 A, Sig=254,4 Ref=off

Peak #	RetTime [min]	Type	Width [min]	Area [mAU*s]	Height [mAU]	Area %
1	11.342	BB	0.1962	2781.48218	219.03716	49.8587
2	13.218	BB	0.2318	2797.24536	187.77988	50.1413
Totals :				5578.72754	406.81703	

Enantioenriched sample 10i:

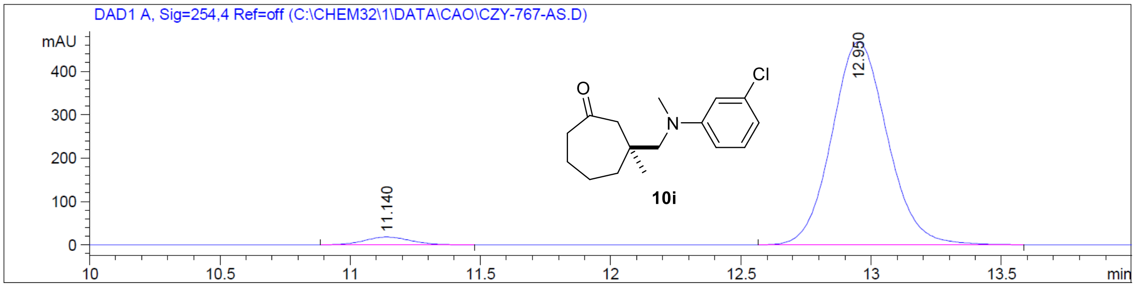

Signal 1: DAD1 A, Sig=254,4 Ref=off

Peak #	RetTime [min]	Type	Width [min]	Area [mAU*s]	Height [mAU]	Area %
1	11.140	BB	0.1958	232.55956	18.35465	3.2516
2	12.950	BB	0.2287	6919.67285	467.44443	96.7484
Totals :				7152.23241	485.79907	

Supplementary Figure 74. HPLC spectra for compound **10i**

Condition: HPLC (Daicel Chiralpak IC-3: 90/10 hexane/IPA, flow rate 1.0 mL/min, λ = 254 nm)

Racemic sample 12:

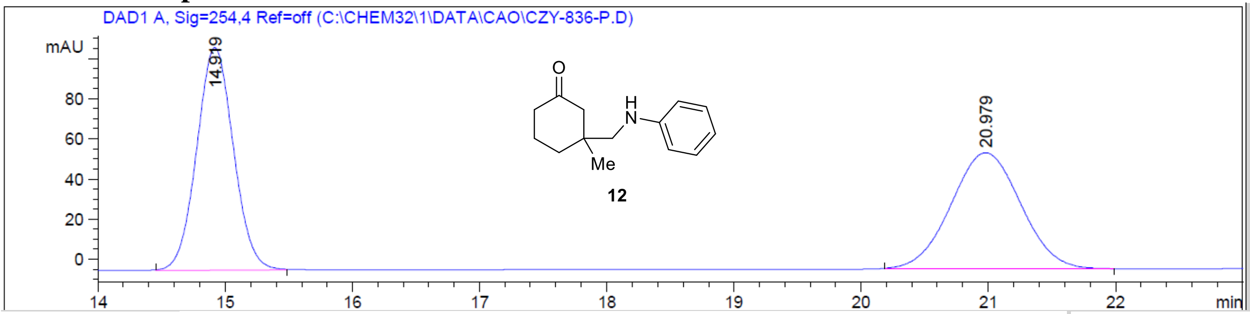

Signal 1: DAD1 A, Sig=254,4 Ref=off

Peak #	RetTime [min]	Type	Width [min]	Area [mAU*s]	Height [mAU]	Area %
1	14.919	BB	0.3115	2217.14673	110.75670	50.0947
2	20.979	BB	0.5957	2208.76733	57.81287	49.9053
Totals :				4425.91406	168.56957	

Enantioenriched sample 12:

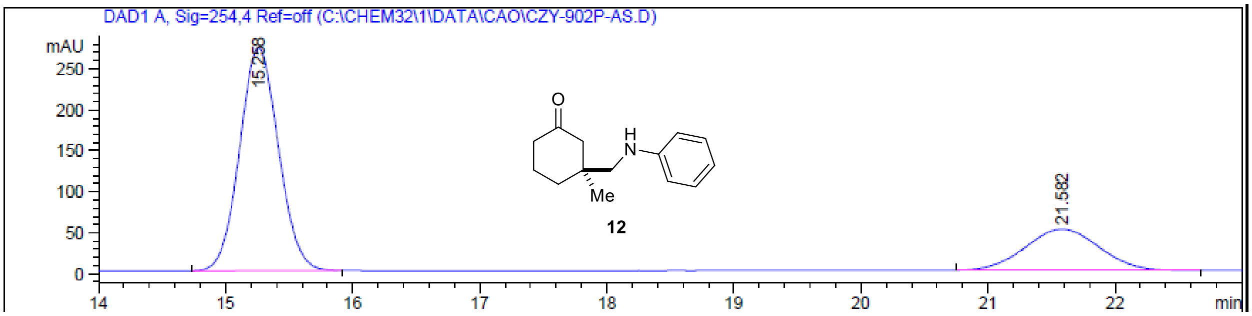

Signal 1: DAD1 A, Sig=254,4 Ref=off

Peak #	RetTime [min]	Type	Width [min]	Area [mAU*s]	Height [mAU]	Area %
1	15.258	BB	0.3306	5799.66748	272.19794	73.7264
2	21.582	BB	0.6414	2066.80225	50.51046	26.2736
Totals :				7866.46973	322.70840	

Supplementary Figure 75. HPLC spectra for compound **12**

Condition: HPLC (Daicel Chiralpak IC-3: 90/10 hexane/IPA, flow rate 1.0 mL/min, λ = 215 nm)

Racemic sample 14:

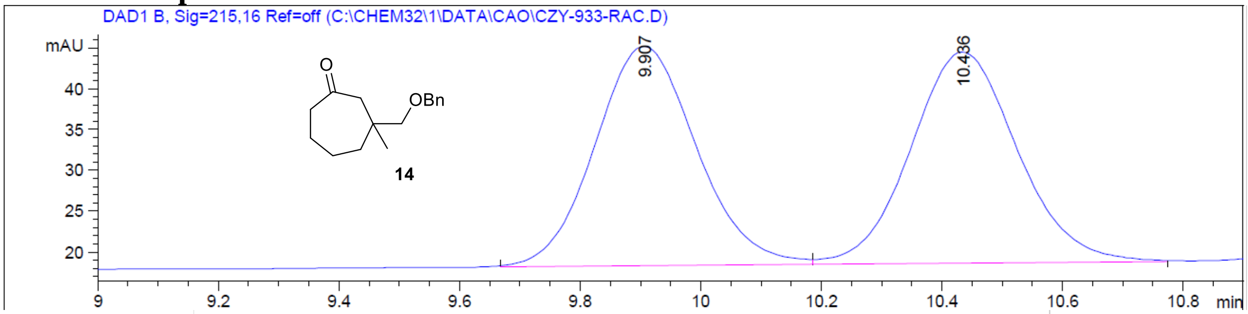

Signal 2: DAD1 B, Sig=215,16 Ref=off

Peak #	RetTime [min]	Type	Width [min]	Area [mAU*s]	Height [mAU]	Area %
1	9.907	BV	0.1806	314.76404	26.87905	49.6697
2	10.436	VB	0.1898	318.95093	25.88188	50.3303
Totals :				633.71497	52.76093	

Enantioenriched sample 14:

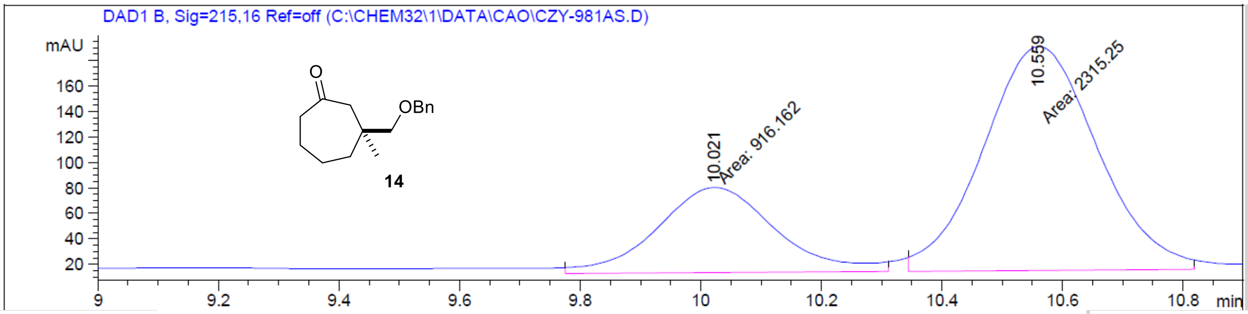

Signal 2: DAD1 B, Sig=215,16 Ref=off

Peak #	RetTime [min]	Type	Width [min]	Area [mAU*s]	Height [mAU]	Area %
1	10.021	MF	0.2276	916.16187	67.07436	28.3517
2	10.559	FM	0.2189	2315.25220	176.24866	71.6483
Totals :				3231.41406	243.32301	

Supplementary Figure 76. HPLC spectra for compound **14**

Condition: HPLC (Daicel Chiralpak IC-3: 90/10 hexane/IPA, flow rate 1.0 mL/min, λ = 254 nm)

Racemic sample 10s:

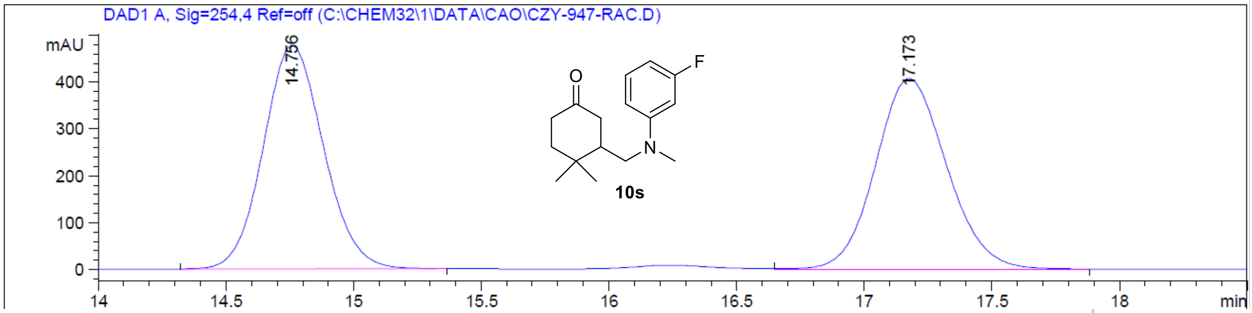

Signal 1: DAD1 A, Sig=254,4 Ref=off

Peak #	RetTime [min]	Type	Width [min]	Area [mAU*s]	Height [mAU]	Area %
1	14.756	BB	0.2542	7846.45801	476.25229	49.9141
2	17.173	VB	0.3003	7873.46045	405.90802	50.0859
Totals :				1.57199e4	882.16031	

Enantioenriched sample 10s:

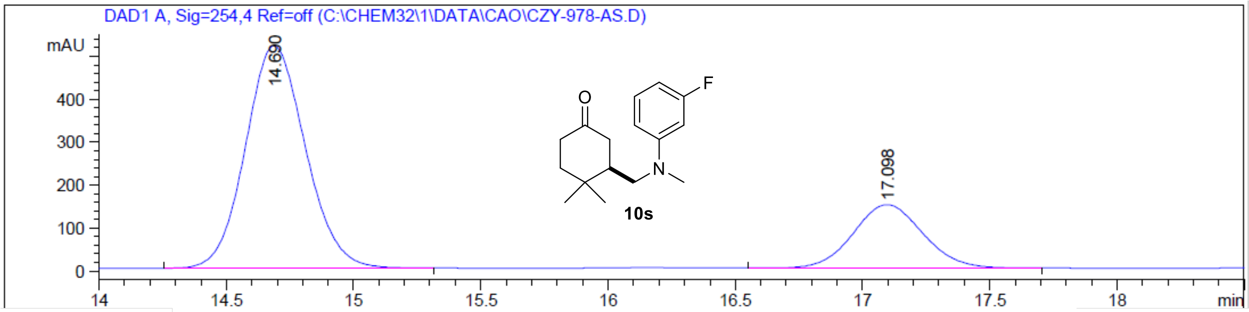

Signal 1: DAD1 A, Sig=254,4 Ref=off

Peak #	RetTime [min]	Type	Width [min]	Area [mAU*s]	Height [mAU]	Area %
1	14.690	BB	0.2534	8494.72559	517.74036	75.0072
2	17.098	VB	0.2953	2830.48071	147.85941	24.9928
Totals :				1.13252e4	665.59976	

Supplementary Figure 77. HPLC spectra for compound **10s**

Condition: UPC[2] (Acquity Trefoil IB column with a gradient (100% CO_2 to 60/40 CO_2/MeOH over 2 minutes, curve 6), flow rate 3 mL/min, λ = 230 nm)

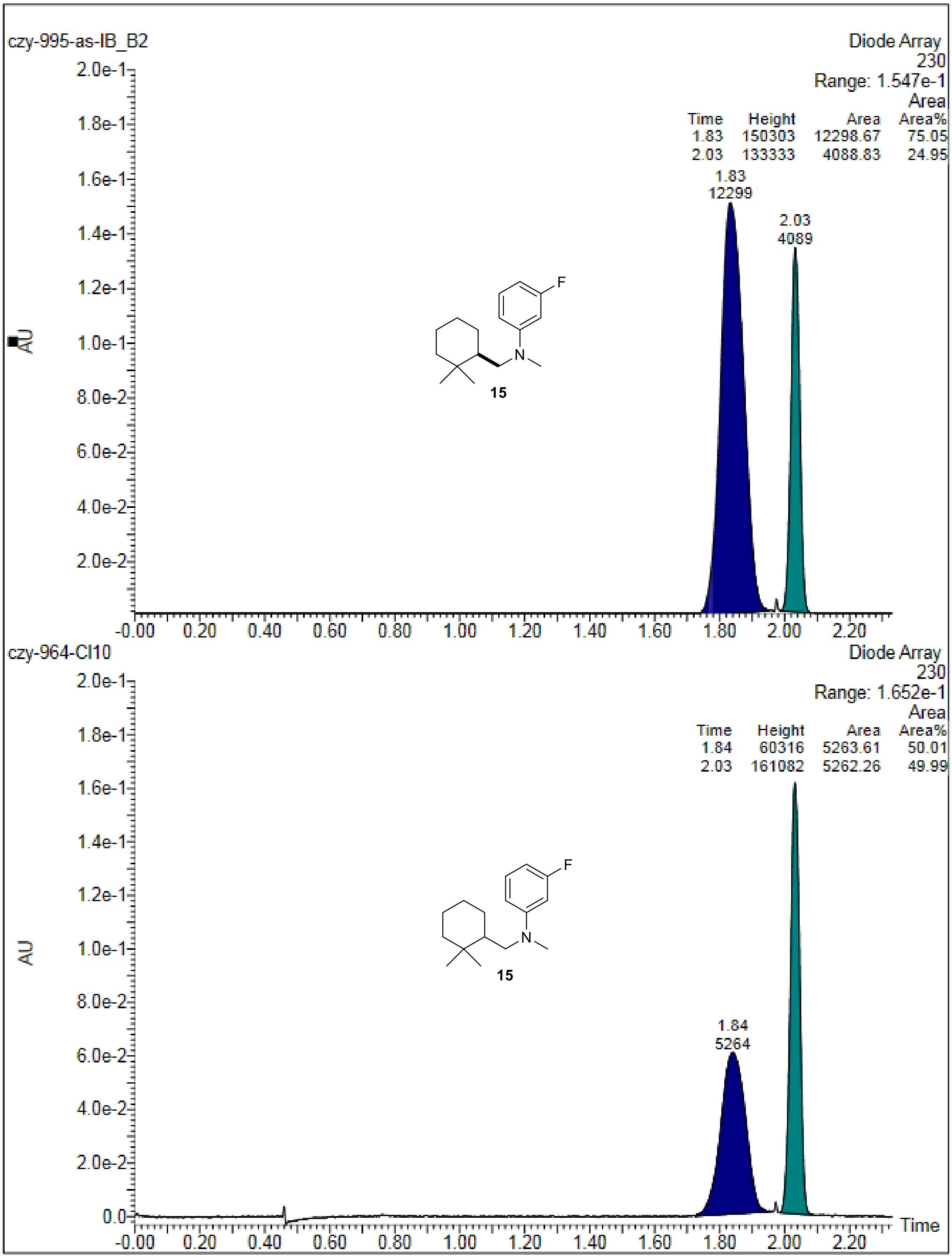

Supplementary Figure 78. UPC[2] spectra for compound **15**

Condition: HPLC (Daicel Chiralpak IC-3: 90/10 hexane/IPA, flow rate 1.0 mL/min, λ = 230 nm)

Racemic sample 10t:

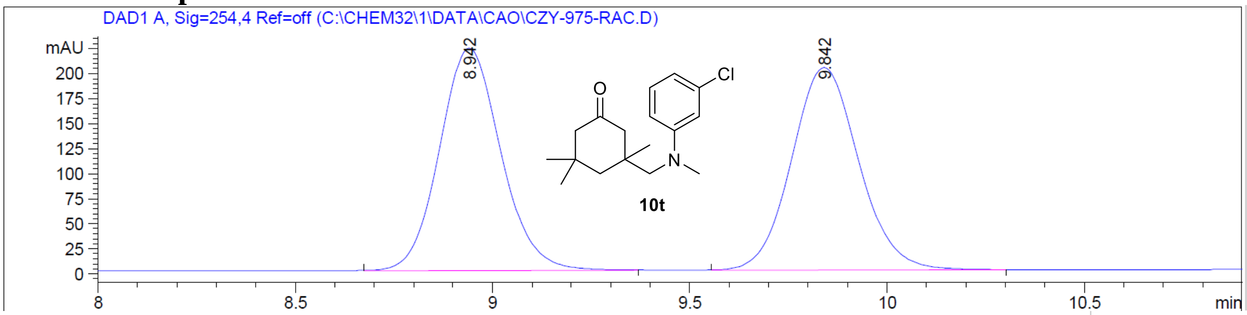

Signal 1: DAD1 A, Sig=254,4 Ref=off

Peak #	RetTime [min]	Type	Width [min]	Area [mAU*s]	Height [mAU]	Area %
1	8.942	BB	0.1681	2403.14282	222.27719	50.0216
2	9.842	BB	0.1844	2401.06616	202.42075	49.9784
Totals :				4804.20898	424.69794	

Enantioenriched sample 10t:

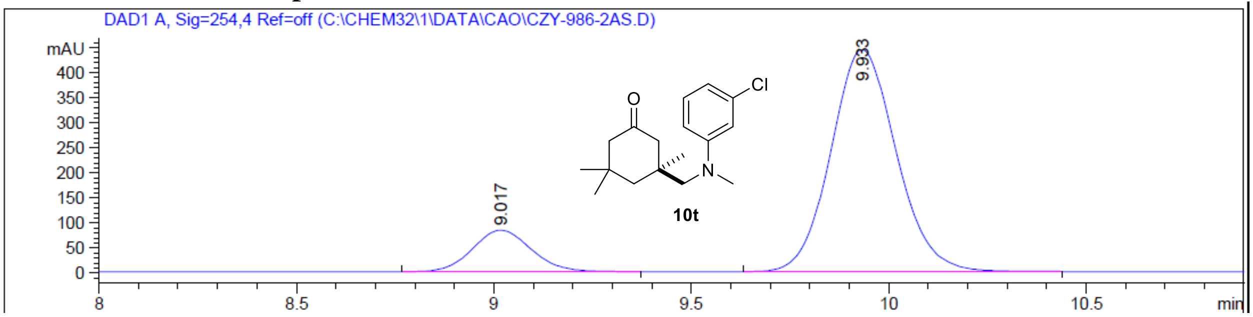

Signal 1: DAD1 A, Sig=254,4 Ref=off

Peak #	RetTime [min]	Type	Width [min]	Area [mAU*s]	Height [mAU]	Area %
1	9.017	BB	0.1616	868.96484	83.35671	14.5371
2	9.933	BB	0.1763	5108.58936	443.91086	85.4629
Totals :				5977.55420	527.26757	

Supplementary Figure 79. HPLC spectra for compound **10t**

Condition: UPC^2 (Acquity Trefoil CEL1 column with a gradient (100% CO_2 to 60/40 CO_2/MeOH over 6 minutes, curve 6), flow rate 2 mL/min, λ = 215 nm)

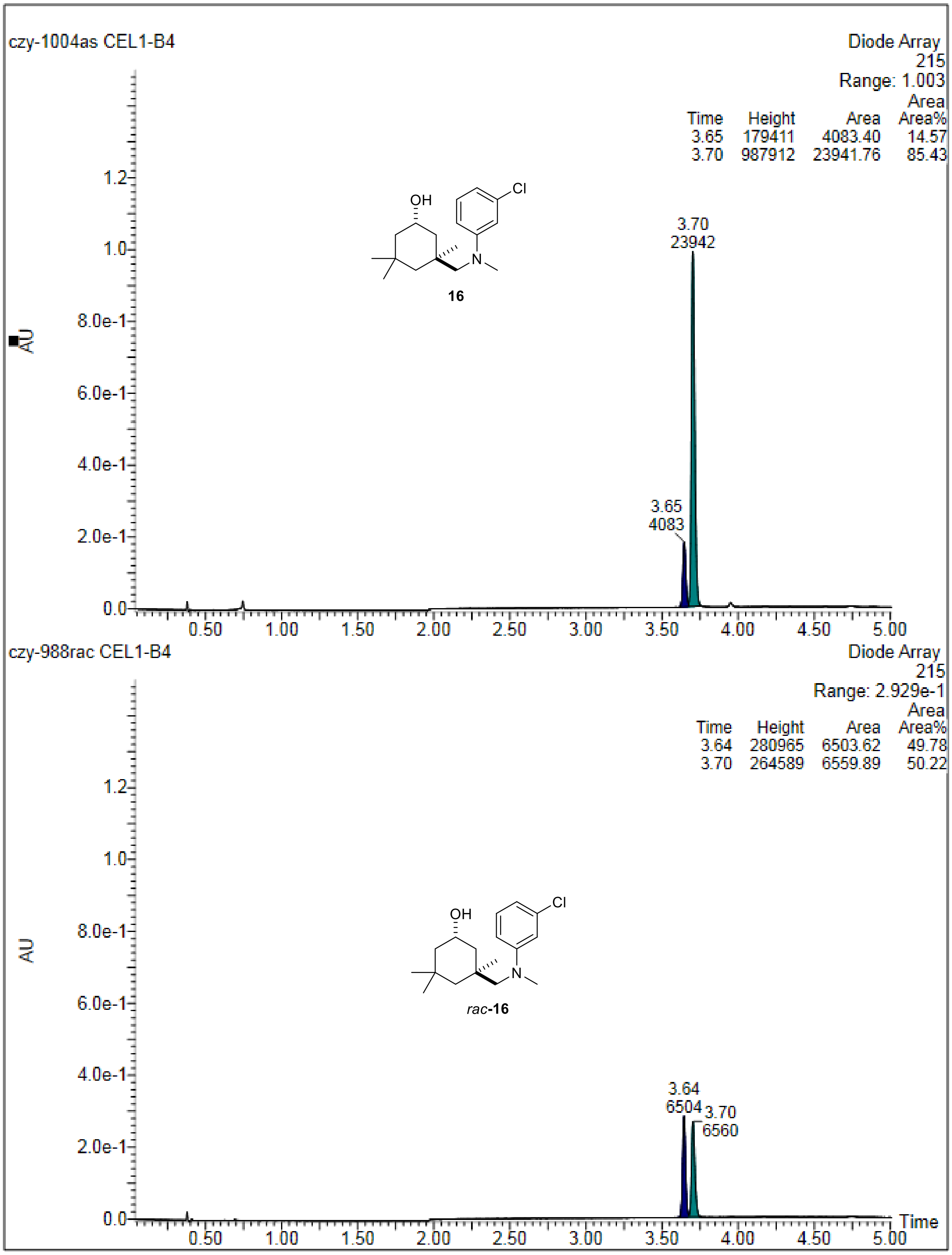

Supplementary Figure 80. UPC^2 spectra for compound **16**

Single Crystal X-ray Diffraction Data for the Product 2a

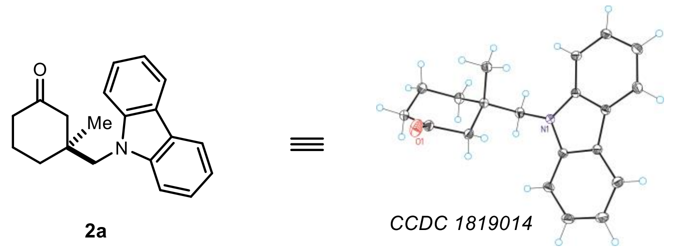

Supplementary Figure 81. Single crystal X-ray diffraction data and determination of the **a**bsolute configuration of **2a** (CCDC 1819014). Crystals of the compound **2a** were obtained by slow evaporation of a CH_3CN solution. *Data Collection*. Measurements were made on a Bruker-Nonius diffractometer equipped with an APPEX 24K CCD area detector, a FR591 rotating anode with MoKα radiation, Montel mirrors and a Cryostream Plus low temperature device (*T* = 100K). Full-sphere data collection was used with ω and φ scans.

Supplementary Tables

Supplementary Table 1. Crystal data and structure refinement for **2a**.

Empirical formula	C20 H21 N O	
Formula weight	291.38	
Temperature	100(2) K	
Wavelength	0.71073 Å	
Crystal system	Orthorhombic	
Space group	P2(1)2(1)2(1)	
Unit cell dimensions	a = 5.97270(5)Å	a= 90°.
	b = 14.47004(13)Å	b = 90°.
	c = 17.80667(17)Å	g = 90°.
Volume	1538.95(2) $Å^3$	
Z	4	
Density (calculated)	1.258 Mg/m^3	
Absorption coefficient	0.076 mm^{-1}	
F(000)	624	
Crystal size	0.2 x 0.2 x 0.05 mm^3	
Theta range for data collection	1.813 to 53.017°.	
Index ranges	-12<=h<=13,-32<=k<=25,-18<=l<=39	
Reflections collected	55982	
Independent reflections	18128[*R*(int) = 0.0170]	
Completeness to theta =53.017°	99.6%	
Absorption correction	Multi-scan	
Max. and min. transmission	0.996 and 0.766	
Refinement method	Full-matrix least-squares on F^2	
Data / restraints / parameters	18128/ 0/ 283	
Goodness-of-fit on F^2	1.031	
Final R indices [I>2sigma(I)]	R1 = 0.0310, wR2 = 0.0800	
R indices (all data)	R1 = 0.0373, wR2 = 0.0833	
Flack parameter	x =0.02(13)	
Largest diff. peak and hole	0.455 and -0.179 e.$Å^{-3}$	

Supplementary Table 2. Bond lengths [Å] and angles [°] for **2a**.

Bond lengths	Angles
O1-C16	1.2194(6)
N1-C1	1.3881(5)
N1-C12	1.3936(5)
N1-C13	1.4528(5)
C1-C2	1.3999(5)
C1-C6	1.4149(5)
C2-C3	1.3913(6)
C2-H2	0.973(12)
C3-C4	1.4053(7)
C3-H3	0.941(12)
C4-C5	1.3894(7)
C4-H4	0.949(16)
C5-C6	1.3980(5)
C5-H5	0.963(12)
C7-C8	1.3997(6)
C7-C12	1.4129(6)
C7-C6	1.4429(5)
C8-C9	1.3905(7)
C8-H8	0.966(11)
C9-C10	1.4051(8)
C9-H9	0.971(13)
C10-C11	1.3932(7)
C10-H10	0.944(16)
C11-C12	1.3979(5)
C11-H11	0.970(14)
C13-C14	1.5421(5)
C13-H12	1.006(12)
C13-H13	1.005(10)
C14-C20	1.5329(5)
C14-C19	1.5389(5)
C14-C15	1.5419(5)
C15-C16	1.5167(6)
C15-H14	0.996(13)
C15-H15	0.995(12)
C16-C17	1.5104(6)
C17-C18	1.5326(7)
C17-H16	0.929(14)
C17-H17	0.988(13)
C18-C19	1.5279(6)
C18-H18	0.971(13)
C18-H19	0.977(13)
C19-H21	0.980(11)
C19-H20	0.925(14)
C20-H22	1.026(11)
C20-H23	0.984(12)
C20-H24	0.988(12)
C1-N1-C12	107.91(3)

C1-N1-C13	125.79(3)
C12-N1-C13	125.87(3)
N1-C1-C2	129.54(4)
N1-C1-C6	109.42(3)
C2-C1-C6	121.03(4)
C3-C2-C1	117.65(4)
C3-C2-H2	122.3(7)
C1-C2-H2	120.1(7)
C2-C3-C4	121.79(4)
C2-C3-H3	122.3(7)
C4-C3-H3	115.9(7)
C5-C4-C3	120.38(4)
C5-C4-H4	116.8(9)
C3-C4-H4	122.9(9)
C4-C5-C6	118.87(4)
C4-C5-H5	121.1(7)
C6-C5-H5	120.0(7)
C8-C7-C12	120.27(4)
C8-C7-C6	133.12(4)
C12-C7-C6	106.52(3)
C5-C6-C1	120.25(4)
C5-C6-C7	133.11(4)
C1-C6-C7	106.61(3)
C9-C8-C7	118.65(4)
C9-C8-H8	119.5(6)
C7-C8-H8	121.8(6)
C8-C9-C10	120.46(4)
C8-C9-H9	122.1(9)
C10-C9-H9	117.4(9)
C11-C10-C9	121.88(4)
C11-C10-H10	120.8(10)
C9-C10-H10	117.2(10)
C10-C11-C12	117.38(4)
C10-C11-H11	121.9(8)
C12-C11-H11	120.7(8)
N1-C12-C11	129.20(4)
N1-C12-C7	109.41(3)
C11-C12-C7	121.35(4)
N1-C13-C14	116.81(3)
N1-C13-H12	108.4(7)
C14-C13-H12	107.1(6)
N1-C13-H13	109.2(6)
C14-C13-H13	108.3(6)
H12-C13-H13	106.6(9)
C20-C14-C19	110.26(3)
C20-C14-C15	109.55(3)
C19-C14-C15	108.49(3)
C20-C14-C13	110.57(3)
C19-C14-C13	106.31(3)
C15-C14-C13	111.58(3)
C16-C15-C14	110.77(3)

C16-C15-H14	107.8(7)
C14-C15-H14	111.3(7)
C16-C15-H15	107.1(7)
C14-C15-H15	108.8(7)
H14-C15-H15	110.9(11)
O1-C16-C17	122.29(4)
O1-C16-C15	121.96(4)
C17-C16-C15	115.76(4)
C16-C17-C18	113.09(3)
C16-C17-H16	102.8(9)
C18-C17-H16	110.5(9)
C16-C17-H17	106.6(8)
C18-C17-H17	114.7(8)
H16-C17-H17	108.3(11)
C19-C18-C17	111.48(3)
C19-C18-H18	109.6(7)
C17-C18-H18	108.2(7)
C19-C18-H19	110.0(8)
C17-C18-H19	111.1(8)
H18-C18-H19	106.2(11)
C18-C19-C14	112.39(3)
C18-C19-H21	107.2(7)
C14-C19-H21	110.1(7)
C18-C19-H20	111.8(8)
C14-C19-H20	109.1(8)
H21-C19-H20	106.1(11)
C14-C20-H22	111.4(6)
C14-C20-H23	109.1(7)
H22-C20-H23	110.3(9)
C14-C20-H24	111.5(7)
H22-C20-H24	107.1(9)
H23-C20-H24	107.4(10)

Supplementary Table 3. Torsion angles [°] for **2a**.

C12-N1-C1-C2	175.24(4)
C13-N1-C1-C2	2.44(7)
C12-N1-C1-C6	-3.54(4)
C13-N1-C1-C6	-176.33(3)
N1-C1-C2-C3	179.28(4)
C6-C1-C2-C3	-2.07(6)
C1-C2-C3-C4	0.98(7)
C2-C3-C4-C5	0.58(7)
C3-C4-C5-C6	-1.05(7)
C4-C5-C6-C1	-0.04(6)
C4-C5-C6-C7	177.77(4)
N1-C1-C6-C5	-179.46(4)
C2-C1-C6-C5	1.64(6)
N1-C1-C6-C7	2.21(4)
C2-C1-C6-C7	-176.69(4)

C8-C7-C6-C5	-1.68(8)
C12-C7-C6-C5	-178.07(4)
C8-C7-C6-C1	176.34(4)
C12-C7-C6-C1	-0.05(4)
C12-C7-C8-C9	1.15(6)
C6-C7-C8-C9	-174.84(4)
C7-C8-C9-C10	-0.08(7)
C8-C9-C10-C11	-0.90(7)
C9-C10-C11-C12	0.75(7)
C1-N1-C12-C11	-174.01(4)
C13-N1-C12-C11	-1.22(7)
C1-N1-C12-C7	3.51(4)
C13-N1-C12-C7	176.29(3)
C10-C11-C12-N1	177.61(4)
C10-C11-C12-C7	0.35(6)
C8-C7-C12-N1	-179.07(4)
C6-C7-C12-N1	-2.12(4)
C8-C7-C12-C11	-1.32(6)
C6-C7-C12-C11	175.63(4)
C1-N1-C13-C14	-91.11(5)
C12-N1-C13-C14	97.36(5)
N1-C13-C14-C20	60.05(4)
N1-C13-C14-C19	179.74(3)
N1-C13-C14-C15	-62.16(4)
C20-C14-C15-C16	63.91(4)
C19-C14-C15-C16	-56.51(4)
C13-C14-C15-C16	-173.30(3)
C14-C15-C16-O1	-128.88(5)
C14-C15-C16-C17	51.70(5)
O1-C16-C17-C18	134.12(5)
C15-C16-C17-C18	-46.47(5)
C16-C17-C18-C19	46.74(5)
C17-C18-C19-C14	-55.12(5)
C20-C14-C19-C18	-60.04(4)
C15-C14-C19-C18	59.94(4)
C13-C14-C19-C18	-179.93(3)

Supplementary Note 1

Synthesis of the Chiral Primary Amine Catalyst 3e

Synthesis of the carbazole precursor

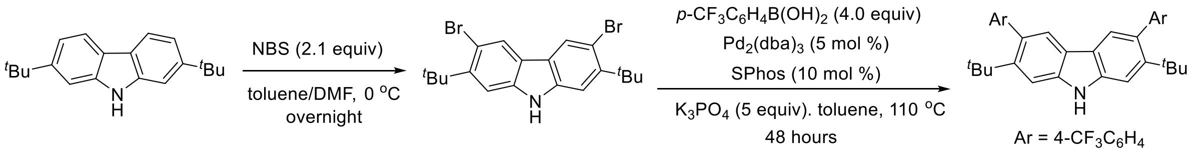

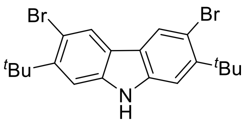

3,6-dibromo-2,7-di-*tert*-butyl-9*H*-carbazole was prepared according to the following procedure: *N*-bromosuccinimide (NBS, 1.85 g, 10.5 mmol, 2.1 equiv) dissolved in DMF (8 mL) was added dropwise over 1 hour to a stirred suspension of 2,7-di-*tert*-butyl-9*H*-carbazole (1.4 g, 5 mmol, 1 equiv) in toluene (5 mL) at 0 ºC. The reaction was stirred at 0 ºC overnight, and then the reaction mixture was poured into ice-water (50 mL). Extraction ethyl acetate and concentration under reduced pressure gave a crude mixture. The product was isolated by flash chromatography (hexane/toluene: gradient from 100:0 to 10:1), giving a white solid (2.16 g, 99% yield).

^{1}H NMR (500 MHz, $CDCl_3$): δ 8.18 (s, 2H), 7.90 (s, br, 1H), 7.50 (s, 2H), 1.60 (s, 18H).
^{13}C NMR (101 MHz, $CDCl_3$): δ 145.7, 139.5, 127.3, 121.8, 113.4, 110.0, 37.2, 30.3.
HRMS: Calculated for $C_{20}H_{22}Br_2N$ (M-H$^+$): 434.0124, found: 434.0109.

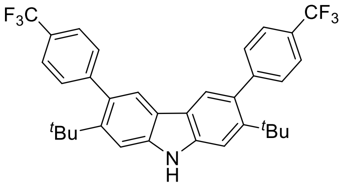

2,7-di-*tert*-butyl-3,6-bis(4-(trifluoromethyl)phenyl)-9H-carbazole was prepared according to a slightly modified literature procedure.[2] A screw-cap Schlenk tube containing a magnetic stirring bar was charged with 3,6-dibromo-2,7-di-*tert*-butyl-9*H*-carbazole (437 mg, 1.0 mmol), 2-dicyclohexylphosphino-2,6-dimethoxybiphenyl (SPhos, 41.0 mg, 10 mol %), $Pd_2(dba)_3$ (45.8 mg, 5 mol %), (4-(trifluoromethyl)phenyl)boronic acid (4.0 mmol, 4.0 equiv) and K_3PO_4 (1.06 g, 5.0 mmol, 5.0 equiv). The tube was sealed with a teflon-coated screw cap and then evacuated and backfilled with argon (three times). Dry toluene (2.0 mL) was added *via* syringe through the septum. The reaction mixture was vigorously stirred at 110 °C until the carbazole was completely consumed, as judged by TLC analysis (48 hours). The reaction mixture was then diluted with ethyl acetate (10 mL), filtered through a thin pad of silica gel (eluting with ethyl acetate) and concentrated under reduced pressure. The crude material was purified by flash chromatography on silica gel (hexane/toluene: gradient from 100:0 to 2:1), to give a pink solid. After recrystallization using ethyl acetate, the desired product was obtained as a white solid (284 mg, 50% yield).

^{1}H NMR (400 MHz, $CDCl_3$): δ 8.01 (s, 1H), 7.69-7.54 (m, 6H), 7.51-7.40 (m, 6H), 1.28 (s, 18H).
^{13}C NMR (101 MHz, $CDCl_3$): δ 150.1, 146.3, 140.2, 133.0, 131.4, 128.9 (q, J = 32.3 Hz), 124.6 (q, J = 273.0 Hz), 124.1 (q, J = 3.8 Hz), 123.8, 120.1, 108.6, 37.1, 33.03.
^{19}F NMR (376 MHz, $CDCl_3$): δ -62.34.
HRMS: Calculated for $C_{34}H_{30}F_6N$ (M-H$^+$): 566.2288, found: 566.2305.

Synthesis of catalyst 3e

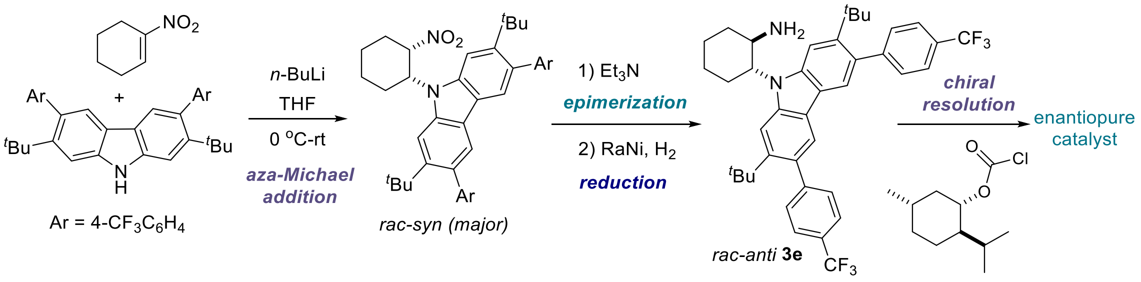

Procedure for the aza-Michael addition.

To an oven dried, argon purged 2-neck round bottomed flask fitted with an argon inlet and septum was added the substituted carbazole (1 equiv) and anhydrous THF (0.05 M). The reaction mixture was cooled to 0 ºC and *n*-BuLi (1.05 equiv) was added dropwise. The reaction was stirred at 0 ºC for 30 minutes, and then 1-nitrocyclohex-1-ene (1.2 equiv) was added to the cold solution. The solution was allowed to slowly reach ambient temperature and stirred until full consumption of the carbazole, as inferred by TLC analysis (hexane/ethyl acetate 20:1). The reaction was then quenched with saturated aqueous NH_4Cl solution and extracted with EtOAc. The organic phase was washed with water and brine, dried over $MgSO_4$ and concentrated. The crude material (*syn*/*anti* = 5:1) was continued to the next step without further purification.

Procedure for the epimerization from *syn* to *anti*.

To the crude nitroalkane (1 equiv) in a round bottomed flask was added THF (0.1 M) and triethylamine (2 equiv). The reaction mixture was heated to 60 ºC until epimerization was complete, as determined by ^1H NMR analysis of the epimeric protons (usually two days were necessary). The reaction mixture was then concentrated to dryness. The crude material was continued to the next step.

Procedure for the reduction of the nitroalkane.

To the crude *anti* nitroalkane (1 equiv), suspended in a solution of EtOAc/*i*-PrOH (1:3) in a Parr hydrogenation flask, was added Raney nickel (commercial slurry in water, 2 tsps per mmol of nitroalkane). The flask was then charged with a hydrogen atmosphere (3-3.5 bar) and shook for 24 hours. The reaction mixture was filtered through celite and rinsed with ethyl acetate (*CAUTION*: Raney nickel oxidizes exothermically, the filter cake must not be allowed to become dry) and concentrated. The residue was purified by flash chromatography (2% MeOH in DCM) to obtain the racemic aminocatalyst **3e** as a white-yellow solid (average 60% yield over 3 steps).

Procedure for the resolution of the racemic catalyst.

To an oven dried, argon purged, round-bottomed flask was added *rac*-**3e** (1.37 mmol, 1 equiv) and anhydrous tetrahydrofuran (5 mL). The solution was cooled to 0 ºC and anhydrous pyridine (154 µL, 1.92 mmol, 1.4 equiv) was added followed by dropwise addition of (1*R*)-(-)-menthyl chloroformate (0.35 mL, 1.64 mmol). The reaction was then stirred overnight at ambient temperature. The reaction was diluted with CH_2Cl_2 and washed with 2 M HCl solution, water and then brine. The organic phase was then dried over $MgSO_4$, and concentrated to an off-white solid. The residue containing both menthyl carbamates of (*S*,*S*)-**3e** and (*R*,*R*)-**3e** was separated by flash chromatography (hexane:DCM 1:1) to afford both of the enantiopure menthyl carbamates of (*R*,*R*)-**3e** (first fraction, 492 mg) and (*S*,*S*)-**3e** (second fraction, 413 mg).

Procedure for the hydrolysis of enantiopure (-)-menthyl carbamate to give (*R*,*R*)-3e.

To an argon purged Teflon vial was added the enantiopure (-)-menthyl carbamate (413 mg, 1 equiv), and 4 mL TBAF solution (1.0 M in THF). The reaction mixture was heated at 130 ºC for two days, and then cooled to ambient temperature. Flash chromatography (dichloro methylene/ethyl acetate: from 100:0 to 0:100, repeated twice) afforded the enantiopure catalyst (*R*,*R*)-**3e** as a white solid (266.6 mg, 85% yield).

^{1}H NMR (400 MHz, $CDCl_3$): δ 7.80 (s, 1H), 7.64-7.50 (m, 7H), 7.47 (d, *J* = 7.9 Hz, 4H), 4.28-4.15 (m, 1H), 3.81 (td, *J* = 10.4, 4.1 Hz, 1H), 2.50-2.36 (m, 1H), 2.31-2.21 (m, 1H), 2.10-1.93 (m, 3H), 1.68-1.40 (m, 4H), 1.31 (s, 18H).
^{13}C NMR (126 MHz, $CDCl_3$): δ 150.1, 150.0, 146.1, 145.4, 142.1, 139.3, 132.6, 132.5, 131.5, 131.4, 129.6, 128.8 (q, *J* = 32.4 Hz), 124.5 (q, *J* = 272.0 Hz), 124.1, 123.9, 123.7, 120.7, 119.5, 109.5, 106.7, 63.3, 52.5, 37.3, 37.2, 35.6, 33.1, 32.0, 29.8, 26.4, 25.6.
^{19}F NMR (376 MHz, $CDCl_3$): δ -62.35.
HPLC: The enantiomeric excess of the catalyst, was determined to be >99% by HPLC analysis on a Daicel Chiralpak IC-3 column: 97/3 hexane/isopropanol, flow rate 0.8 mL/min, λ = 254 nm: τ_{Major} = 5.91 min, τ_{Minor} = 6.32 min. $[\alpha]_D^{25}$ = +13.1 (c = 0.20, $CHCl_3$)
HRMS: Calculated for $C_{40}H_{43}F_6N_2$ ($M+H^+$): 665.3325, found: 665.3343.

Synthesis of the Organic Silane Substrates

General procedure for the synthesis of carbazole or aniline derived organic silanes 4 or 9

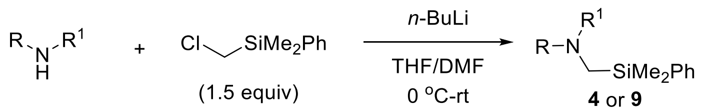

To a stirred solution of the opportune carbazole or aniline substrate (10 mmol), dissolved in 5 mL of anhydrous THF, was added anhydrous DMF (or HMPA for aniline substrate, 5 mL). After cooling to -78 °C, *n*-BuLi (2.5 M in hexane, 1.0 equiv) was added slowly, and the reaction mixture was allowed to reach ambient temperature. After cooling again to -78 °C degree, 1.5 equiv of $ClCH_2SiMe_2Ph$ were added. The reaction mixture was warmed to ambient temperature and stirring continued overnight. After consumption of the carbazole or aniline substrate, as inferred by GC-MS or TLC analysis, the reaction mixture was quenched by adding EtOAc and washed with H_2O three times. The organic phase were washed with brine, dried over Mg_2SO_4, filtered and concentrated. The crude mixture was purified by silica gel (for aniline derived silane **9**, neutral silica gel was used) column chromatography to afford the desired products.

Characterization of α-carbazole silyl substrates 4

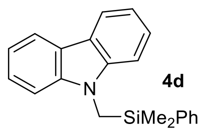

9-((dimethyl(phenyl)silyl)methyl)-9*H*-carbazole 4d
Following the procedure described above, using 9*H*-carbazole (1.67 g, 10.0 mmol), (chloromethyl)dimethyl(phenyl)silane (2.7 mL), and *n*-BuLi (10.0 mmol), silane **4d** (2.82 g, 8.93 mmol, 89%) was obtained as a white solid after column chromatography (from hexane (100%) to hexane/Et_2O (50:1)).
^{1}H NMR (500 MHz, $CDCl_3$): δ 8.11 (d, *J* = 10.0 Hz, 2H), 7.57-7.49 (m, 2H), 7.45-7.33 (m, 5H), 7.29-7.15 (m, 4H), 4.05 (s, 2H), 0.31 (s, 6H).
^{13}C NMR (126 MHz, $CDCl_3$): δ 140.8, 137.2, 133. 8, 129.8, 128.2, 125.4, 122.7, 120.3, 118.5, 109.1, 34.3, -2.9. HRMS: Calculated for $C_{21}H_{21}NNaSi$ ($M+Na^+$): 338.1335, found 338.1348.

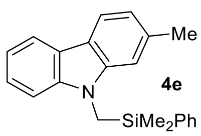

9-((dimethyl(phenyl)silyl)methyl)-2-methyl-9*H*-carbazole 4e
Following the procedure described above, using 2-Me-9*H*-carbazole (362 mg, 2.0 mmol), (chloromethyl)dimethyl(phenyl)silane (541 µL), and *n*-BuLi (2.0 mmol), silane **4e** (496 mg, 1.50 mmol, 75%) was obtained as a light yellow oil after column chromatography (from hexane (100%) to hexane/Et_2O (50:1)).
^{1}H NMR (400 MHz, $CDCl_3$): δ 8.04 (d, *J* = 7.7 Hz, 1H), 7.95 (d, *J* = 7.9 Hz, 1H), 7.56-7.49 (m, 2H), 7.44-7.32 (m, 4H), 7.22-7.13 (m, 2H), 7.00 (d, *J* = 7.9 Hz, 1H), 6.92 (s, 1H), 4.00 (s, 2H), 2.47 (s, 3H), 0.30 (s, 6H). ^{13}C NMR (101 MHz, $CDCl_3$): δ 141.3, 140.9, 137.4, 135.5, 133.8, 129.8, 128.2, 124.9, 122. 8, 120.4, 120.0, 119.9, 119.9, 118.3, 109.4, 108.9, 34.2, 22.4, -3.0.
HRMS: Calculated for $C_{22}H_{24}NSi$ ($M+H^+$): 330.1673, found 330.1663.

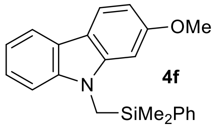

9-((dimethyl(phenyl)silyl)methyl)-2-methoxy-9*H*-carbazole 4f

Following the procedure described above, using 2-Me-9*H*-carbazole (395 mg, 2.0 mmol), (chloromethyl)dimethyl(phenyl)silane (541 µL), and *n*-BuLi (2.0 mmol), silane **4f** (555 mg, 1.60 mmol, 80%) was obtained as a white solid after column chromatography (from hexane (100%) to hexane/Et_2O (50:1)).

^{1}H NMR (500 MHz, $CDCl_3$): δ 7.99 (ddd, *J* = 7.7, 1.3, 0.7 Hz, 1H), 7.93 (dd, *J* = 8.4, 0.5 Hz, 1H), 7.55-7.48 (m, 2H), 7.42-7.30 (m, 4H), 7.22-7.13 (m, 2H), 6.79 (dd, *J* = 8.5, 2.2 Hz, 1H), 6.59 (d, *J* = 2.2 Hz, 1H), 3.98 (s, 2H), 3.78 (s, 3H), 0.31 (s, 6H).

^{13}C NMR (126 MHz, $CDCl_3$): δ 158.9, 142.1, 141.0, 137.4, 133.8, 129.8, 128.2, 124.2, 122.9, 121.0, 119.4, 118.6, 116.6, 108.8, 107.3, 93.3, 55.6, 34.3, -3.0.

HRMS: Calculated for $C_{22}H_{24}NOSi$ ($M+H^+$): 346.1622, found 346.1635.

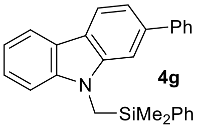

9-((dimethyl(phenyl)silyl)methyl)-2-phenyl-9*H*-carbazole 4g

Following the procedure described above, using 2-phenyl-9*H*-carbazole (972 mg, 4.0 mmol), (chloromethyl)dimethyl(phenyl)silane (1.6 mL), and *n*-BuLi (4.0 mmol), silane **4g** (1.27 g, 3.24 mmol, 81%) was obtained as a white solid after column chromatography (from hexane (100%) to hexane/Et_2O (50:1)).

^{1}H NMR (400 MHz, $CDCl_3$): δ 8.18-8.05 (m, 2H), 7.59-7.50 (m, 4H), 7.48-7.30 (m, 9H), 7.26-7.16 (m, 2H), 4.08 (s, 2H), 0.34 (s, 6H).

^{13}C NMR (101 MHz, $CDCl_3$): δ 142.4, 141.4, 141.3, 138.9, 137.2, 133.8, 129.8, 128.8, 128.3, 127.7, 127.0, 125.5, 122.5, 122.0, 120.4, 120.3, 118.7, 118.2, 109.1, 107.9, 34.4, -3.0.

HRMS: Calculated for $C_{27}H_{25}NSiNa$ ($M+Na^+$): 414.1648, found 414.1644.

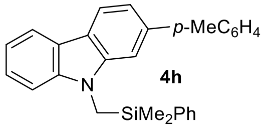

2-(4-methylphenyl)-9-((dimethyl(phenyl)silyl)methyl)-9*H*-carbazole 4h

Following the procedure described above, using 2-(4-methylphenyl)-9*H*-carbazole (772 mg, 3.0 mmol), (chloromethyl)dimethyl(phenyl)silane (810 µL), and *n*-BuLi (3.0 mmol), silane **4h** (866 mg, 2.75 mmol, 92%) was obtained as a white solid after column chromatography (from hexane (100%) to hexane/Et_2O (50:1)).

^{1}H NMR (500 MHz, $CDCl_3$): δ 8.12-8.08 (m, 2H), 7.55-7.50 (m, 2H), 7.48-7.43 (m, 2H), 7.43-7.33 (m, 5H), 7.33-7.30 (m, 1H), 7.28-7.24 (m, 3H), 7.22-7.18 (m, 1H), 4.07 (s, 2H), 2.43 (s, 3H), 0.33 (s, 6H).

^{13}C NMR (126 MHz, $CDCl_3$): δ 141.3, 139.5, 138.9, 137.3, 136.8, 133.8, 129.8, 129.5, 128.3, 127.5, 125.4, 122.6, 121.7, 120.4, 120.3, 118.6, 118.1, 109.1, 107.6, 34.3, 21.3, -2.9.

HRMS: Calculated for $C_{28}H_{27}NNaSi$ ($M+Na^+$): 428.1805, found 428.1807.

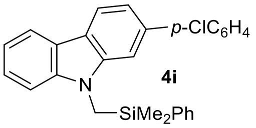

2-(4-chlorophenyl)-9-((dimethyl(phenyl)silyl)methyl)-9*H*-carbazole 4i

Following the procedure described above, using 2-(4-chlorophenyl)-9*H*-carbazole (833 mg, 3.0 mmol), (chloromethyl)dimethyl(phenyl)silane (810 µL), and *n*-BuLi (3.0 mmol), silane **4i** (1.0 g, 2.35 mmol, 78%) was obtained as a light yellow oil after column chromatography (from hexane (100%) to hexane/Et_2O (50:1)).

^{1}H NMR (400 MHz, $CDCl_3$): δ 8.14-8.06 (m, 2H), 7.54-7.48 (m, 2H), 7.46-7.30 (m, 9H), 7.30-7.13 (m, 3H), 4.08 (s, 2H), 0.33 (s, 6H).

^{13}C NMR (126 MHz, $CDCl_3$): δ 141.4, 141.2, 140.8, 137.5, 137.3, 133.9, 133.1, 129.8, 128.9, 128.9, 128.3, 125.7, 122.4, 122.2, 120.5, 120.4, 118.8, 117.9, 109.1, 107.8, 34.4, -3.0.

HRMS: Calculated for $C_{27}H_{24}NClNaSi$ ($M+Na^+$): 448.1259, found 448.1261.

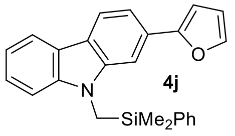

9-((dimethyl(phenyl)silyl)methyl)-2-(furan-2-yl)-9*H*-carbazole 4j

Following the procedure described above, using 2-(thiophen-2-yl)-9H-carbazole (465 mg, 2.0 mmol), (chloromethyl)dimethyl(phenyl)silane (540 µL), and *n*-BuLi (2.0 mmol), silane **4j** (404 mg, 1.32 mmol, 53%) was obtained as a yellow oil after column chromatography (from hexane (100%) to hexane/toluene (20:1)).

^{1}H NMR (400 MHz, $CDCl_3$) δ 8.06 (dd, *J* = 8.1, 0.7 Hz, 2H), 7.56-7.46 (m, 5H), 7.42- 7.31 (m, 4H), 7.23-7.13 (m, 2H), 6.64 (dd, *J* = 3.3, 0.8 Hz, 1H), 6.51 (dd, *J* = 3.4, 1.8 Hz, 1H), 4.07 (s, 2H), 0.32 (s, 6H).
^{13}C NMR (101 MHz, $CDCl_3$): δ 155.2, 141.9, 141.4, 141.1, 137.2, 133.8, 129.8, 128.4, 128.2, 125.5, 122.6, 122.0, 120.5, 120.2, 118.7, 115.1, 111.9, 109.1, 104.8, 104.5, 34.4, -3.0.
HRMS: Calculated for $C_{25}H_{24}NOSi$ (M+H$^+$): 382.1622, found 382.1621.

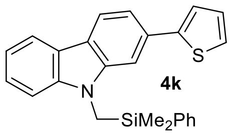

4k

9-((dimethyl(phenyl)silyl)methyl)-2-(thiophen-2-yl)-9*H*-carbazole 4k
Following the procedure described above, using 2-(thiophen-2-yl)-9H-carbazole (750 mg, 3.0 mmol), (chloromethyl)dimethyl(phenyl)silane (810 µL), and *n*-BuLi (3.0 mmol), silane **4k** (526 mg, 1.32 mmol, 44%) was obtained as a green oil after column chromatography (from hexane (100%) to hexane/Et_2O (50:1)).
^{1}H NMR (400 MHz, $CDCl_3$): δ 8.10-8.01 (m, 2H), 7.54-7.50 (m, 2H), 7.46 (dd, *J* = 8.1, 1.5 Hz, 1H), 7.42-7.32 (m, 5H), 7.30-7.26 (m, 2H), 7.25-7.15 (m, 2H), 7.10 (dd, *J* = 5.1, 3.6 Hz, 1H), 4.06 (s, 2H), 0.33 (s, 6H).
^{13}C NMR (126 MHz, $CDCl_3$): δ 145.9, 141.5, 141.2, 137.1, 133.8, 131.9, 129.9, 128.3, 128.1, 125.6, 124.5, 123.0, 122.5, 122.2, 120.6, 120.3, 118.8, 117.3, 109.1, 106.6, 34.4, -3.0.
HRMS: Calculated for $C_{25}H_{24}NSSi$ (M+H$^+$): 398.1393, found 398.1400.

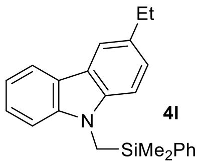

4l

9-((dimethyl(phenyl)silyl)methyl)-3-ethyl-9*H*-carbazole 4l
Following the procedure described above, using 3-Et-9*H*-carbazole (390 mg, 2.0 mmol), (chloromethyl)dimethyl(phenyl)silane (541 µL), and *n*-BuLi (2.0 mmol), silane **4l** (354 mg, 1.03 mmol, 51%) was obtained as a light yellow oil after column chromatography (from hexane (100%) to hexane/Et_2O (50:1)).
^{1}H NMR (400 MHz, $CDCl_3$): δ 8.07 (d, *J* = 7.7 Hz, 1H), 7.91 (d, *J* = 0.9 Hz, 1H), 7.55-7.51 (m, 2H), 7.44-7.32 (m, 4H), 7.25 (d, *J* = 10.7 Hz, 1H), 7.21-7.12 (m, 3H), 4.01 (s, 2H), 2.84 (q, *J* = 7.6 Hz, 2H), 1.35 (t, *J* = 7.6 Hz, 3H), 0.29 (s, 6H).
^{13}C NMR (101 MHz, $CDCl_3$): δ 141.1, 139.4, 137.3, 134.5, 133.8, 129.8, 128.2, 125.8, 125.2, 122.8, 122.6, 120.2, 119.0, 118.2, 109.0, 108.9, 34.3, 29.1, 16.6, -2.9.
HRMS: Calculated for $C_{23}H_{26}NSi$ (M+H$^+$): 344.1829, found 344.1823.

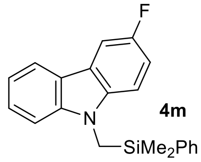

4m

9-((dimethyl(phenyl)silyl)methyl)-3-fluoro-9*H*-carbazole 4m
Following the procedure described above, using 3-fluoro-9*H*-carbazole (463 mg, 2.5 mmol), (chloromethyl)dimethyl(phenyl)silane (540 µL), and *n*-BuLi (2.5 mmol), silane **4m** (454 mg, 1.36 mmol, 54%) was obtained as a white solid after column chromatography (from hexane (100%) to hexane/Et_2O (50:1)).
^{1}H NMR (400 MHz, $CDCl_3$): δ 8.03 (ddd, *J* = 7.8, 1.2, 0.7 Hz, 1H), 7.73 (ddd, *J* = 8.9, 2.4, 0.7 Hz, 1H), 7.50-7.44 (m, 2H), 7.43-7.30 (m, 4H), 7.24-7.14 (m, 2H), 7.15-7.03 (m, 2H), 4.01 (s, 2H), 0.29 (s, 6H).
^{19}F NMR (376 MHz, $CDCl_3$): δ -125.7.
^{13}C NMR (101 MHz, $CDCl_3$): δ 157.1 (d, *J* = 235.3 Hz), 141.60, 137.1 (d, *J* = 25.2 Hz), 133.76, 129.87, 128.24, 126.02, 122.9 (d, *J* = 10.1 Hz), 122.2 (d, *J* = 4.0 Hz), 120.5, 118.4, 113.1 (d, *J* = 26.3 Hz), 109.5 (d, *J* = 9.1 Hz), 109.4, 106.0, 105.8, 34.5, -3.0.
HRMS: Calculated for $C_{21}H_{20}FNNaSi$ (M+Na$^+$): 356.1241, found 356.1235.

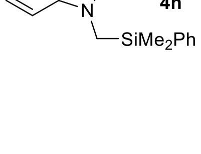

4n

9-((dimethyl(phenyl)silyl)methyl)-4-methyl-9*H*-carbazole 4n
Following the procedure described above, using 4-Me-9*H*-carbazole (318 mg, 1.75 mmol), (chloromethyl)dimethyl(phenyl)silane (473 µL), and *n*-BuLi (1.75 mmol), silane **4n** (492 mg, 1.49 mmol, 85%) was obtained as a light yellow oil after column chromatography (from hexane (100%) to hexane/Et_2O (50:1)).

^{1}H NMR (500 MHz, $CDCl_3$): δ 8.24-8.16 (m, 1H), 7.57-7.50 (m, 2H), 7.44-7.34 (m, 4H), 7.30 (dd, *J* = 8.2, 7.2 Hz, 1H), 7.25-7.18 (m, 2H), 7.13-7.07 (m, 1H), 6.98 (dt, *J* = 7.2, 0.9 Hz, 1H), 4.04 (s, 2H), 2.90 (s, 3H), 0.28 (s, 6H).
^{13}C NMR (126 MHz, $CDCl_3$): δ 140.9, 140.8, 137.3, 133.8, 133.4, 129.8, 128.2, 125.2, 124.8, 123.3, 122.6, 121.2, 120.2, 118.4, 108.9, 106.8, 34.2, 21.0, -2.9.
HRMS: Calculated for $C_{22}H_{24}NSi$ ($M+H^+$): 330.1673, found 330.1682.

Characterization of α-aniline silyl substrates 9

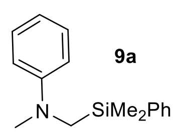
9a

***N*-((dimethyl(phenyl)silyl)methyl)-*N*-methylaniline 9a**
Following the procedure described above, using (chloromethyl)dimethyl(phenyl)silane (1.35 mL), *N*-methylaniline (535 mg, 5.0 mmol), and *n*-BuLi (5.0 mmol), silane **9a** (562 mg, 2.20 mmol, 44%) was obtained as a colorless oil after column chromatography (from hexane (100%) to hexane/toluene (10:1)).
^{1}H NMR (500 MHz, $CDCl_3$): δ 7.58-7.52 (m, 2H), 7.42-7.33 (m, 3H), 7.23-7.14 (m, 2H), 6.68-6.60 (m, 3H), 3.07 (s, 2H), 2.82 (s, 3H), 0.37 (s, 6H).
^{13}C NMR (126 MHz, $CDCl_3$): δ 150.7, 138.5, 133.8, 129.4, 129.1, 128.1, 115.5, 112.2, 43.8, 40.4, -2.6.
HRMS: Calculated for $C_{16}H_{22}NSi$ ($M+H^+$): 256.1516, found 256.1507.

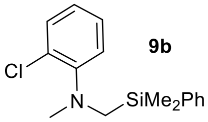
9b

2-Chloro-*N*-((dimethyl(phenyl)silyl)methyl)-*N*-methylaniline 9b
Following the procedure described above, using (chloromethyl)dimethyl(phenyl)silane (1.35 mL), 2-chloro-*N*-methylaniline (708 mg, 5.0 mmol), and *n*-BuLi (5.0 mmol), silane **9b** (753 mg, 2.6 mmol, 52%) was obtained as a colorless oil after column chromatography (from hexane (100%) to hexane/toluene (10:1)).
^{1}H NMR (500 MHz, $CDCl_3$): δ 7.58-7.53 (m, 2H), 7.37-7.29 (m, 4H), 7.16-7.07 (m, 2H), 6.94-6.88 (m, 1H), 2.85 (s, 2H), 2.70 (s, 3H), 0.34 (s, 6H).
^{13}C NMR (126 MHz, $CDCl_3$): δ 152.3, 138.9, 133.8, 130.6, 129.3, 129.2, 127.9, 127.3, 123.4, 121.7, 47.3, 45.3, -2.8.
HRMS: Calculated for $C_{16}H_{21}ClNSi$ ($M+H^+$): 290.1126, found 290.1123.

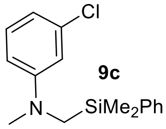
9c

3-chloro-*N*-((dimethyl(phenyl)silyl)methyl)-*N*-methylaniline 9c
Following the procedure described above, using (chloromethyl)dimethyl(phenyl)silane (1.35 mL), 3-chloro-*N*-methylaniline (708 mg, 5 mmol), and *n*-BuLi (5.0 mmol), silane **9c** (757 mg, 2.62 mmol, 52%) was obtained as a yellow oil after column chromatography (from hexane (100%) to hexane/toluene (10:1)).
^{1}H NMR (500 MHz, $CDCl_3$): δ 7.55-7.49 (m, 2H), 7.42-7.33 (m, 3H), 7.09-7.02 (m, 1H), 6.60-6.55 (m, 2H), 6.51-6.44 (m, 1H), 3.05 (s, 2H), 2.81 (s, 3H), 0.37 (s, 6H).
^{13}C NMR (126 MHz, $CDCl_3$): δ 151.4, 138.0, 135.0, 133.8, 129.9, 129.5, 128.1, 115.1, 111.7, 110.1, 43.8, 40.3, -2.7.
HRMS: Calculated for $C_{16}H_{21}ClNSi$ ($M+H^+$): 290.1126, found 290.1131.

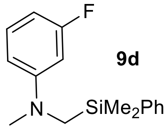
9d

***N*-((dimethyl(phenyl)silyl)methyl)-3-fluoro-*N*-methylaniline 9d**
Following the procedure described above, using (chloromethyl)dimethyl(phenyl)silane (2.7 mL), 3-fluoro-*N*-methylaniline (1.25 g, 10.0 mmol), and *n*-BuLi (10.0 mmol), silane **9d** (1.32 g, 4.80 mmol, 48%) was obtained as a yellow oil after column chromatography (from hexane (100%) to hexane/toluene (10:1)).
^{1}H NMR (500 MHz, $CDCl_3$): δ 7.57-7.50 (m, 2H), 7.44-7.34 (m, 3H), 7.14-7.03 (m, 1H), 6.41-6.35 (m, 1H), 6.35-6.24 (m, 2H), 3.06 (s, 2H), 2.81 (s, 3H), 0.37 (s, 6H).
^{13}C NMR (126 MHz, $CDCl_3$): δ 164.3 (d, *J* = 241.2 Hz), 152.1 (d, *J* = 10.7 Hz), 138.1, 133.8, 130.0 (d, *J* = 10.6 Hz), 129.5, 128.1, 107.6 (d, *J* = 1.9 Hz), 101.7 (d, *J* = 21.7 Hz), 98.8 (d, *J* = 26.1 Hz), 43.8, 40.3, -2.7.
^{19}F NMR (376 MHz, $CDCl_3$): δ -113.13.

HRMS: Calculated for $C_{16}H_{21}FNSi$ $(M+H^+)$: 274.1422, found 274.1427.

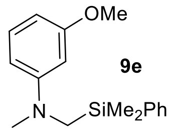

N-((dimethyl(phenyl)silyl)methyl)-3-methoxy-N-methylaniline 9e

Following the procedure described above, using (chloromethyl)dimethyl(phenyl)silane (1.35 mL), 3-methoxy-*N*-methylaniline (686 mg, 5.0 mmol), and *n*-BuLi (5.0 mmol), silane **9e** (695 mg, 2.4 mmol, 48%) was obtained as a colorless oil after column chromatography (from hexane (100%) to hexane/toluene (10:1)).

^{1}H NMR (500 MHz, $CDCl_3$): δ 7.60-7.51 (m, 2H), 7.43-7.31 (m, 3H), 7.10 (t, J = 8.3 Hz, 1H), 6.26 (ddd, J = 29.1, 8.1, 2.3 Hz, 2H), 6.19 (s, 1H), 3.76 (s, 3H), 3.07 (s, 2H), 2.82 (s, 3H), 0.37 (s, 6H).

^{13}C NMR (126 MHz, $CDCl_3$): δ 160.8, 152.0, 138.4, 133.8, 129.7, 129.4, 128.1, 105.4, 100.5, 98.5, 55.2, 43.9, 40.4, -2.6.

HRMS: Calculated for $C_{17}H_{24}NOSi$ $(M+H^+)$: 286.1622, found 286.1626.

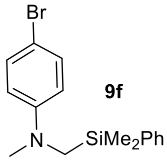

4-Bromo-N-((dimethyl(phenyl)silyl)methyl)-N-methylaniline 9f

Following the procedure described above, using (chloromethyl)dimethyl(phenyl)silane (1.35 mL), 4-bromo-*N*-methylaniline (925 mg, 5.0 mmol), and *n*-BuLi (5.0 mmol), silane **9f** (853 mg, 2.55 mmol, 51%) was obtained as a yellow oil after column chromatography (from hexane (100%) to hexane/toluene (10:1)).

^{1}H NMR (500 MHz, $CDCl_3$): δ 7.55-7.48 (m, 2H), 7.41-7.33 (m, 3H), 7.25-7.18 (m, 2H), 6.48 (d, J = 8.8 Hz, 2H), 3.03 (s, 2H), 2.79 (s, 3H), 0.35 (s, 6H).

^{13}C NMR (126 MHz, $CDCl_3$): δ 149.4, 138.1, 133.7, 131.6, 129.5, 128.1, 113.6, 107.2, 43.9, 40.4, -2.6.

HRMS: Calculated for $C_{16}H_{21}BrNSi$ $(M+H^+)$: 334.0621, found 334.0618.

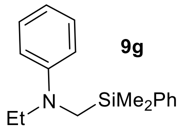

N-((dimethyl(phenyl)silyl)methyl)-N-ethylaniline 9g

Following the procedure described above, using (chloromethyl)dimethyl(phenyl)silane (1.35 mL), *N*-ethylaniline (606 mg, 5.0 mmol), and *n*-BuLi (5.0 mmol), silane **9g** (595 mg, 2.21 mmol, 44%) was obtained as a colorless oil after column chromatography (from hexane (100%) to hexane/toluene (10:1)).

^{1}H NMR (500 MHz, $CDCl_3$): δ 7.58-7.53 (m, 2H), 7.41-7.35 (m, 3H), 7.24-7.10 (m, 2H), 6.69-6.57 (m, 3H), 3.27 (q, J = 7.0 Hz, 2H), 3.02 (s, 2H), 1.03 (t, J = 7.0 Hz, 3H), 0.37 (s, 6H).

^{13}C NMR (126 MHz, $CDCl_3$): δ 149.1, 138.6, 133.8, 129.3, 129.1, 128.1, 115.1, 112.3, 46.5, 40.8, 10.7, -2.6.

HRMS: Calculated for $C_{17}H_{24}NSi$ $(M+H^+)$: 270.1673, found 270.1668.

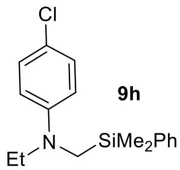

4-Chloro-N-((dimethyl(phenyl)silyl)methyl)-N-ethylaniline 9h

Following the procedure described above, using (chloromethyl)dimethyl(phenyl)silane (1.35 mL), 4-chloro-*N*-ethylaniline (1.56 g, 5.0 mmol), and *n*-BuLi (5.0 mmol), silane **9h** (589 mg, 1.94 mmol, 38%) was obtained as a colorless oil after column chromatography (from hexane (100%) to hexane/toluene (10:1)).

^{1}H NMR (400 MHz, $CDCl_3$): δ 7.55-7.47 (m, 2H), 7.41-7.32 (m, 3H), 7.12-7.01 (m, 2H), 6.57-6.43 (m, 2H), 3.22 (q, J = 7.0 Hz, 2H), 2.97 (s, 2H), 1.00 (t, J = 7.0 Hz, 3H), 0.34 (s, 6H).

^{13}C NMR (101 MHz, $CDCl_3$): δ 147.6, 138.3, 133.7, 129.5, 128.8, 128.1, 119.8, 113.4, 46.7, 41.1, 10.6, -2.7.

HRMS: Calculated for $C_{17}H_{23}ClNSi$ $(M+H^+)$: 304.1283, found 304.1284.

Supplementary Note 2

Synthesis of Iminium Ion A-2

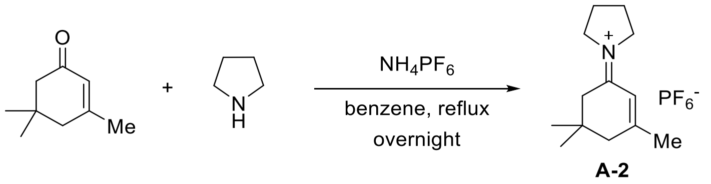

To a solution of isophorone (20.0 mmol, 1.0 equiv) and pyrrolidine (20.0 mmol, 1.0 equiv) in 25 mL benzene were added ammonium hexafluorophosphate (20.0 mmol, 1.0 equiv).[3] The suspension was refluxed overnight with continuous removal of the formed water (using a *Dean-Stark* apparatus). Then, the solvent was evaporated under reduced pressure to afford a yellow crude solid. After washing it with dry diethyl ether and acetonitrile, the iminium ion **A-2** (3.56 g, 53%) were obtained as a white solid.
^{1}H NMR (400 MHz, $CDCl_3$): δ 6.42 (d, J = 1.4 Hz, 1H), 3.99-3.81 (m, 4H), 2.63 (s, 2H), 2.34 (s, 2H), 2.24-2.12 (m, 7H), 1.09 (s, 6H).
^{13}C NMR (75 MHz, $CDCl_3$): δ 173.2, 172.4, 117.1, 53.0, 52.7, 45.4, 42.7, 32.7, 28.1, 26.6, 24.4, 24.4.
^{19}F NMR (376 MHz, $CDCl_3$): δ -73.6 (d, J = 712.6 Hz).
^{31}P NMR (162 MHz, $CDCl_3$): δ -141.51 (sept, J = 711.2 Hz).
HRMS: Calculated for $C_{13}H_{22}N$ (M-PF_6^-): 192.1747, found 192.1738.

Supplementary Note 3

Mechanism studies

Radical Trapping Experiment with Ethene-1,1-diyldibenzene

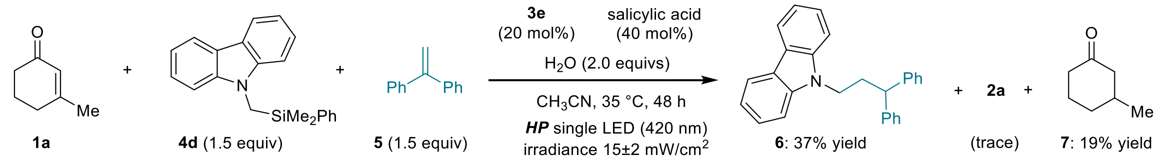

A 15 mL Schlenk tube was charged with the racemic primary amine catalyst **3e** (13.3 mg, 0.04 mmol, 20 mol%), salicylic acid (5.5 mg, 0.08 mmol, 40 mol%), 9-((dimethyl(phenyl)silyl)methyl)-9*H*-carbazole **4d** (47.3 mg, 0.15 mmol, 150 mol%), H_2O (0.2 mmol, 200 mol%), 3-methylcyclohex-2-en-1-one **1a** (11.0 mg, 0.1 mmol, 100 mol%), ethene-1,1-diyldibenzene **5** (27.0 mg, 0.15 mmol, 150 mol%), and 200 μL CH_3CN. The mixture was placed under an atmosphere of argon, cooled to –78 °C, and degassed *via* vacuum evacuation (5 min), backfilled with argon, and warmed to ambient temperature. The freeze-pump-thaw cycle was repeated three times, and then the Schlenk tube was sealed with Parafilm and placed into a 3D-printed plastic support mounted on an aluminium block fitted with a 420 nm high-power single LED (λ = 420 nm). The irradiance was fixed at 15±2 mW/cm^2, as controlled by an external power supply and measured using a photodiode light detector at the start of each reaction; the temperature was kept at 35 °C with a chiller connected to the irradiation plate (the setup is the same as in Supplementary Figure 1). Stirring was maintained for 48 hours, and then the irradiation was stopped. The reaction mixture was analyzed by NMR and GC-MS spectroscopic analysis, confirming the formation of radical addition product **6** in 37% NMR yield (using 0.1 mmol trichloroethylene as the internal standard). The use of semi-preparative HPLC method enabled us to get the pure product **6** as a white solid.
^{1}H NMR (500 MHz, $CDCl_3$): δ 8.09 (dt, J = 7.8, 1.0 Hz, 2H), 7.40 (ddd, J = 8.3, 7.1, 1.2 Hz, 2H), 7.32 (dd, J = 8.0, 6.9 Hz, 4H), 7.27 (dd, J = 8.8, 1.8 Hz, 4H), 7.25-7.19 (m, 4H), 7.16 (dd, J = 8.2, 0.9 Hz, 2H), 4.32-4.21 (m, 2H), 4.02 (t, J = 7.9 Hz, 1H), 2.67-2.54 (m, 2H).

^{13}C NMR (126 MHz, $CDCl_3$): δ 144.1, 140.4, 128.9, 127.9, 126.8, 125.7, 123.1, 120.5, 119.0, 108.8, 49.0, 41.7, 34.4.
HRMS: Calculated for $C_{27}H_{24}N$ ($M+H^+$): 362.1903, found 362.1905.
Control experiments performed in the absence of light or primary amine catalyst **3e** system did not lead to the formation of any radical addition product.

Intercepting the β-Enaminyl Radical Intermediate with 1,4-Cyclohexadiene

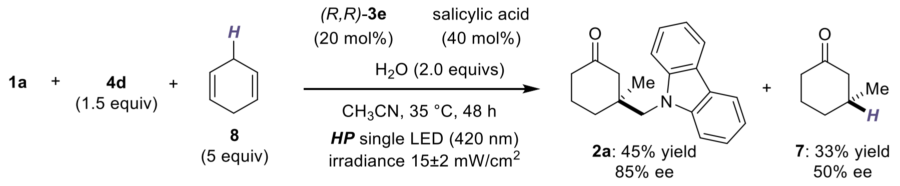

A 15 mL Schlenk tube was charged with the primary amine catalyst (*R*,*R*)-**3e** (13.3 mg, 0.04 mmol, 20 mol%), salicylic acid (5.5 mg, 0.08 mmol, 40 mol%), 9-((dimethyl(phenyl)silyl)methyl)-9*H*-carbazole **4d** (47.3 mg, 0.15 mmol, 150 mol%), H_2O (0.2 mmol, 200 mol%), 3-methylcyclohex-2-en-1-one **1a** (11.0 mg, 0.1 mmol, 100 mol%), 1,4-cyclohexadiene **8** (40.1 mg, 0.5 mmol, 500 mol%), and 200 μL of CH_3CN. The mixture was placed under an atmosphere of argon, cooled to –78 °C, and degassed *via* vacuum evacuation (5 min), backfilled with argon, and warmed to room temperature. The freeze-pump-thaw cycle was repeated three times, and then the Schlenk tube was sealed with parafilm and placed into a 3D-printed plastic support mounted on an aluminium block fitted with a 420 nm high-power single LED (λ = 420 nm). The irradiance was fixed at 15±2 mW/cm^2, as controlled by an external power supply and measured using a photodiode light detector at the start of each reaction; the temperature was kept at 35 °C with a chiller connected to the irradiation plate (the setup is the same as in Supplementary Figure 1). Stirring was maintained for 48 hours, and then the irradiation was stopped. The reaction mixture was analyzed by NMR (using 0.1 mmol of trichloroethylene as the internal standard) and GC-MS spectroscopic analysis, confirming the formation of radical addition product **2a** (45% NMR yield) and 3-methylcyclohexan-1-one **7** (33% NMR yield). Both the NMR and GC-MS spectroscopic traces of **7** are in accordance with the authentic sample, bought from Sigma-Aldrich Company.
In order to check the enantiomeric excess of product **7**, 4-methylbenzenesulfonohydrazine (37.2 mg, 0.2 mmol) and 1 mL MeOH was added to the reaction mixture, and then stirred for two hours. The crude material was purified by flash column chromatography on silicon gel (hexane/ethyl acetate: gradient from 10:1 to 4:1), to afford the corresponding cyclic hydrazone product (white solid, *Z*/*E* = 1:1). The enantioselectivity of the hydrazone was measured by Waters ACQUITY® UPC^2 instrument (condition: UPC^2, Trefoil AMY-1 column, 100% CO_2 to 60/40 CO_2/MeCN over 4 minutes, flow rate: 3.00 mL/min, λ = 230 nm, $\tau_{Major\,(Z,\,E)}$ = 4.2, 4.3 min; $\tau_{Minor\,(Z,\,E)}$ = 4.1, 4.4 min). The absolute configuration of the hydrazone was determined to be *R* by comparison with the UPC^2 traces of an authentic sample of enantiopure (*R*)-hydrazone, prepared from the condensation of 4-methylbenzenesulfonohydrazide with the commercially available (*R*)-**7** (Sigma-Aldrich Company).

Characterization of the hydrazone derived from product **7**: ^{1}H NMR (500 MHz, $CDCl_3$): δ 7.93 (s, br, 1H), 7.83 (d, *J* = 8.1 Hz, 2H), 7.28 (d, *J* = 8.1 Hz, 2H), 2.67-2.58 (m, 1H), 2.45-2.30 (m, 4H), 2.05-1.51 (m, 5H), 1.45-1.27 (m, 1H), 1.15-0.97 (m, 1H), 0.88 (t, *J* = 6.3 Hz, 3H).
^{13}C NMR (126 MHz, $CDCl_3$): δ 143.9, 135.5, 129.5, 128.1, 43.3, 35.1, 34.9, 33.8, 33.5, 32.7, 26.5, 25.7, 24.7, 21.9, 21.8, 21.7.
HRMS: Calculated for $C_{14}H_{19}N_2O_2S$ ($M-H^+$): 279.1173, found: 279.1162.

Control experiments performed in the absence of light, primary amine catalyst **3** or salicylic acid did not lead to the formation of product **7**.

Supplementary Note 4

Synthetic applications

Synthesis of 15

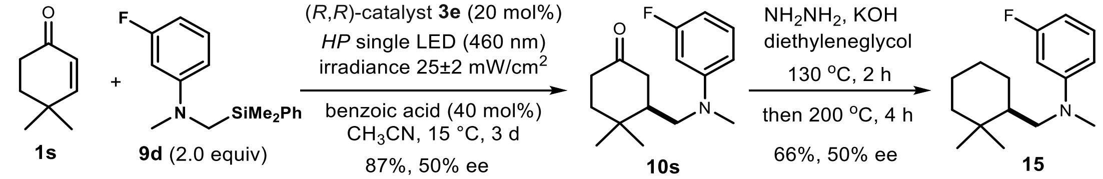

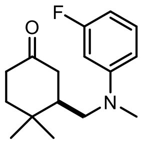

10s

(*R*)-3-(((3-fluorophenyl)(methyl)amino)methyl)-4,4-dimethylcyclohexan-1-one 10s
Prepared according to the general procedure using 3-fluoro-*N*-((dimethyl(phenyl)silyl)methyl)-*N*-methylaniline **9d** (0.2 mmol, 54.7 mg), 4,4-dimethylcyclohex-2-en-1-one **1s** (0.1 mmol, 12.4 mg), the aminocatalyst (*R*,*R*)-**3e** (0.02 mmol, 13.3 mg), benzoic acid (0.04 mmol, 4.9 mg), and 0.2 mL of CH_3CN as the solvent. Time of irradiation: 3 days. The crude mixture was purified by flash column chromatography (hexane/ethyl acetate: gradient from 10:1 to 4:1) to afford the product as a colorless oil (22.9 mg, 87% yield, 50% ee). The enantiomeric excess was determined to be 50% by HPLC analysis on a Daicel Chiralpak IC-3: 90/10 hexane/IPA, flow rate 1.0 mL/min, λ = 254 nm: τ_{Major} = 14.7 min, τ_{Minor} = 17.1 min.
$[\alpha]_D^{25}$ = -19.3 (c = 0.06, $CHCl_3$, 50% ee).
^{1}H NMR (500 MHz, $CDCl_3$): δ 7.12 (td, *J* = 8.2, 7.1 Hz, 1H), 6.54-6.23 (m, 3H), 3.62 (dd, *J* = 14.7, 3.6 Hz, 1H), 3.03 (dd, *J* = 14.7, 10.8 Hz, 1H), 2.91 (s, 3H), 2.49-2.34 (m, 2H), 2.30 (dddd, *J* = 15.1, 4.9, 4.0, 2.2 Hz, 1H), 2.19 (ddd, *J* = 14.6, 11.9, 1.0 Hz, 1H), 2.11-2.00 (m, 1H), 1.77-1.62 (m, 2H), 1.19 (s, 3H), 1.13 (s, 3H). ^{13}C NMR (126 MHz, $CDCl_3$): δ 211.2, 164.3 (d, *J* = 242.0 Hz), 151.1 (d, *J* = 10.6 Hz), 130.3 (d, *J* = 10.3 Hz), 107.7 (d, *J* = 2.1 Hz), 102.8 (d, *J* = 21.7 Hz), 99.1 (d, *J* = 26.2 Hz), 54.3, 44.8, 42.0, 40.7, 40.0, 38.3, 32.4, 28.8, 19.9.
^{19}F NMR (376 MHz, $CDCl_3$): δ -112.50.
HRMS: Calculated for $C_{16}H_{22}FNNaO$ (M+Na$^+$): 286.1578, found 285.1569.

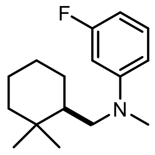

15

(*R*)-*N*-((2,2-dimethylcyclohexyl)methyl)-3-fluoro-*N*-methylaniline 15
A solution of the ketone **10s** (18.3 mg, 0.069 mmol), KOH (20 mg, 0.35 mmol, 5 equiv), and hydrazine monohydrate (20 uL, 10 equiv) in diethylene glycol (0.15 mL) was heated to 130 °C during 2 h and then heated to reflux (200 °C) for 4 h. The mixture was cooled to room temperature and direct purification via flash chromatography to afford the product as colorless oil. (11.4 mg, 66%, 50% ee). The enantiomeric excess was determined to be 91% by UPC^2 analysis on a Acquity Trefoil IB column with a gradient (100% CO_2 to 60/40 CO_2/MeOH over 2 minutes, curve 6), flow rate 3 mL/min, λ = 230 nm: τ_{Major} = 1.83 min, τ_{Minor} = 2.03 min. $[\alpha]_D^{25}$ = +15.1 (c = 0.11, $CHCl_3$, 50% ee).
^{1}H NMR (400 MHz, $CDCl_3$): δ 7.12 (td, J = 8.2, 7.1 Hz, 1H), 6.47-6.25 (m, 3H), 3.55 (dd, J = 14.6, 3.1 Hz, 1H), 2.96-2.97 (m, 4H), 1.71-1.65 (m, 1H), 1.61-1.52 (m, 2H), 1.51-1.42 (m, 1H), 1.42-1.31 (m, 2H), 1.27-1.10 (m, 3H), 1.07 (s, 3H), 0.88 (s, 3H).
^{13}C NMR (101 MHz, $CDCl_3$): δ 164.3 (d, J = 241.1 Hz), 151.6, 130.1 (d, J = 10.6 Hz), 107.4, 101.9 (d, J = 21.5 Hz), 98.7 (d, J = 26.0 Hz), 54.4, 44.8, 42.3, 39.9, 32.0, 30.6, 26.79, 26.5, 22.5, 19.7.
^{19}F NMR (376 MHz, $CDCl_3$): δ -112.89.
HRMS: Calculated for $C_{16}H_{25}FN$ (M+H$^+$): 250.1966, found 250.1958.

Synthesis of *cis*-16

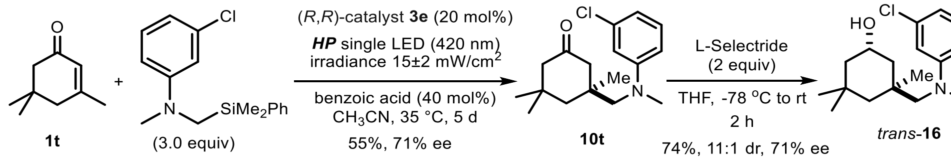

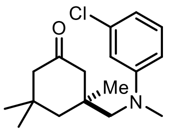

10t

(*S*)-3-(((3-chlorophenyl)(methyl)amino)methyl)-3,5,5-trimethylcyclohexan-1-one 10t Prepared according to the general procedure using 3-chloro-*N*-((dimethyl(phenyl)silyl)methyl)-*N*-methylaniline **9c** (0.3 mmol, 86.7 mg), 3,5,5-trimethylcyclohex-2-en-1-one **1t** (0.1 mmol, 13.8 mg), the aminocatalyst (*R*,*R*)-**3e** (0.02 mmol, 13.3 mg), benzoic acid (0.04 mmol, 4.9 mg), and 0.2 mL of CH_3CN as the solvent. Time of irradiation: 5 days. The crude mixture was purified by flash column chromatography (hexane/ethyl acetate: gradient from 10:1 to 4:1) to afford the product as a green oil (16.1 mg, 55% yield, 71% ee). The enantiomeric excess was determined to be 71% by HPLC analysis on a Daicel Chiralpak IC-3: 90/10 hexane/IPA, flow rate 1.0 mL/min, λ = 230 nm: τ_{Major} = 9.9 min, τ_{Minor} = 9.0 min. $[\alpha]_D^{25}$ = -8.2 (c = 0.12, $CHCl_3$, 71% ee).

^{1}H NMR (400 MHz, $CDCl_3$): δ 7.11 (dd, *J* = 8.5, 7.8 Hz, 1H), 6.85-6.50 (m, 3H), 3.18 (s, 2H), 3.00 (s, 3H), 2.56-2.36 (m, 1H), 2.36-2.00 (m, 3H), 1.79 (d, *J* = 13.9 Hz, 1H), 1.52 (d, *J* = 13.9 Hz, 1H), 1.08 (s, 3H), 1.06 (s, 3H), 1.06 (s, 3H).

^{13}C NMR (101 MHz, $CDCl_3$): δ 211.8, 151.7, 135.1, 130.0, 116.5, 112.3, 110.5, 65.1, 54.0, 51.3, 48.4, 43.5, 42.4, 36.2, 34.3, 29.4, 25.5.

HRMS: Calculated for $C_{17}H_{24}ClNNaO$ ($M+Na^+$): 316.1439, found 316.1439.

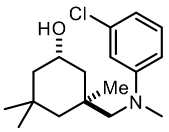

trans-16

(1*S*,3*S*)-3-(((3-chlorophenyl)(methyl)amino)methyl)-3,5,5-trimethylcyclohexan-1-ol 16 To a stirred solution of ketone **10t** (16.1 mg, 0.055 mmol) in THF (0.5 mL) was dropwise added L-selectride (1.0 M in THF, 0.11 mL, 0.11 mmol, 2.0 equiv) at -78 °C. After 5 minutes stirring, the reaction mixture was warmed to 0 °C and stirring was continued at the same temperature for 2 hours. An aqueous saturated solution of potassium sodium tartrate (4 mL) was added and the resulting mixture was vigorously stirred at room temperature for 20 minutes. CH_2Cl_2 (4 mL) was added to the mixture. The organic phase was separated, and the aqueous layer was extracted with CH_2Cl_2 (2 × 4 mL). The combined organic extracts were washed with brine (6 mL), dried over anhydrous Na_2SO_4, and concentrated under reduced pressure. The residue was purified by flash column chromatography on silica gel eluting with Et_2O/*n*-hexane (from 1:4 to 4:1) to afford the corresponding *trans*-**17** adduct (12.0 mg, 0.04 mmol, 74% yield) as a colorless liquid. The enantiomeric excess was determined to be 71% by UPC^2 analysis on a Acquity Trefoil CEL1 column with a gradient (100% CO_2 to 60/40 CO_2/MeOH over 5 minutes, curve 6), flow rate 2 mL/min, λ = 215 nm: τ_{Major} = 3.70 min, τ_{Minor} = 3.65 min. $[\alpha]_D^{25}$ = -15.6 (c = 0.12, $CHCl_3$, 71% ee).

^{1}H NMR (400 MHz, CD_3OD): δ 7.10 (t, *J* = 8.1 Hz, 1H), 6.71-6.65 (m, 2H), 6.57 (ddd, *J* = 7.8, 1.9, 0.8 Hz, 1H), 4.03 (tt, *J* = 11.1, 3.9 Hz, 1H), 3.57 (d, *J* = 15.3 Hz, 1H), 3.19 (dd, *J* = 15.4, 1.3 Hz, 1H), 3.01 (s, 3H), 2.06 (ddd, *J* = 13.2, 3.9, 2.0 Hz, 1H), 1.75 (ddt, *J* = 12.5, 4.0, 2.0 Hz, 1H), 1.46 (dt, *J* = 14.2, 1.9 Hz, 1H), 1.15 (d, *J* = 14.1 Hz, 1H), 1.08 (s, 3H), 1.06 (d, *J* = 2.7 Hz, 1H), 1.00 (s, 3H), 0.96-0.94 (m, 1H), 0.92 (s, 3H).

^{13}C NMR (101 MHz, CD_3OD): δ 153.4, 135.9, 130.9, 116.5, 112.9, 111.6, 66.1, 60.4, 51.8, 45.9, 42.2, 41.1, 35.5, 33.2, 30.8, 29.3.

HRMS: Calculated for $C_{17}H_{26}ClNNaO$ ($M+Na^+$): 318.1595, found 318.1601.

The stereochemical assignment of compound **16** was based on ^{1}H-^{1}H NOESY spectroscopic experiments performed on a 400 MHz instrument. Diagnostic interactions are shown in Supplementary Figure 47.

Supplementary Methods

The NMR spectra were recorded at 400 MHz or 500 MHz for ^{1}H, 101 or 126 MHz for ^{13}C, 286 MHz for ^{19}F, and 162 MHz for ^{31}P. The chemical shift (δ) for ^{1}H and ^{13}C are given in ppm relative to residual signals of the solvents ($CHCl_3$ @ 7.26 ppm ^{1}H NMR and 77.16 ppm ^{13}C NMR, or CD_3OD @ 3.31 ppm ^{1}H NMR and 49.00 ppm ^{13}C NMR, and tetramethylsilane @ 0 ppm). Coupling constants are given in Hertz. The following abbreviations are used to indicate the multiplicity: s, singlet; d, doublet; q, quartet; p, pentet; sept, septet; m, multiplet; br, broad signal. NMR yields were determined by adding trichloroethylene (Cl_2=ClH, δ = 6.44 ppm) as an internal standard to the crude reaction mixtures and by integration of diagnostic signals. High-resolution mass spectra (HRMS) were obtained from the ICIQ High Resolution Mass Spectrometry Unit on MicroTOF Focus and Maxis Impact (Bruker Daltonics) with electrospray ionization. UV-vis measurements were carried out on a Shimadzu UV-2401PC spectrophotometer equipped with photomultiplier detector, double beam optics and D_2 and W light sources. Cyclic voltammetry studies were carried out on an IJ-Cambria HI-730 Bipotentiostat using a three-electrode cell, offering compliance voltage up to ± 100 V (available at the counter electrode), ± 10 V scan range and ± 2 A current range.

General Procedures. All reactions were set up under an argon atmosphere in oven-dried glassware using standard Schlenk techniques, unless otherwise stated. Synthesis grade solvents were used as purchased. Anhydrous solvents were taken from a commercial SPS solvent dispenser. Chromatographic purification of products was accomplished using force-flow chromatography (FC) on silica gel (35-70 mesh). For thin layer chromatography (TLC) analysis throughout this work, Merck pre-coated TLC plates (silica gel 60 GF_{254}, 0.25 mm) were employed, using UV light as the visualizing agent. Organic solutions were concentrated under reduced pressure on a Büchi rotary evaporator (*in vacuo* at 40 ºC, ~5 mbar).

Determination of Enantiomeric Purity: HPLC analysis on chiral stationary phase was performed on an Agilent 1200-series instrument, employing Daicel Chiralpak ID and IC-3 columns, or on a Waters ACQUITY® UPC^{2} instrument, using a Trefoil AMY1, IB, CEL1 chiral column. The exact conditions for the analyses are specified within the characterization section. HPLC traces were compared to racemic samples prepared performing the reaction in the presence of the racemic carbazole-derived primary amine catalyst **3d**.

Materials. Commercial grade reagents and solvents were purchased from Sigma-Aldrich, Fluka, Alfa Aesar, and Fluorochem at the highest commercial quality and used without further purification, unless otherwise stated. Starting material, including 3-methyl-2-cyclohexenone **1a**, 3-methyl-2-cyclopentenone, linear enones **1q-r**, $ClCH_2SiMe_2Ph$, and *N*-phenylglycine were purchased from commercial source and used as received. All other cyclic enones **1** were prepared following a literature procedure.[4,5] The preparation of carbazole substituted silanes **4** and aniline-derived silanes **9** is detailed in Supplementary Note 1. The chiral primary amine catalysts **3a-3d** were prepared according to procedures reported in the literature.[1,6] (Benzyloxy)methyl substituted dihydropyridine **13** was prepared according to a reported literature precedure.[7]

General Procedure for the Photochemical Reactions

Light Illumination System and General Procedure

A 15 mL Schlenk tube was charged with the chiral carbazole-derived primary amine catalyst (*R*,*R*)-**3e** (0.04 mmol, 20 mol%), acid (0.08 mmol, 40 mol%, salicylic acid for substrate **4** and benzoic acid for substrates **9**), the organic silane **4** or **9** (0.15 mmol, 150 mol%), enone **1** (0.1 mmol, 100 mol%), H_2O (0.2 mmol, 200 mol%) and 200 µL of CH_3CN. The mixture was placed under an atmosphere of argon, cooled with liquid nitrogen, and degassed *via* vacuum evacuation (5 min), backfilled with argon, and warmed to room temperature. The freeze-pump-thaw cycle was repeated three times, and then the Schlenk tube was placed into a 3D-printed plastic support mounted on an aluminium block fitted with a 420 nm high-power single LED (λ = 420 nm). The irradiance was regulated at 15±2 mW/cm^2, as controlled by an external power supply and measured using a photodiode light detector at the start and the end of each reaction; the temperature was kept at 35 °C with a chiller connected to the irradiation plate (the setup is detailed in Supplementary Figure 1). This setup secured a reliable irradiation and temperature while keeping a distance of 1 cm between the reaction vessel and the light source. Stirring was maintained for the indicated time (generally 48 hours), and then the irradiation was stopped. The reaction volatiles were removed in vacuum and the residue was purified by column chromatography to give the products **2** or **10** in the stated yield and enantiomeric purity. The reported yield and ee are average of two runs per substrate.

Characterization of Products

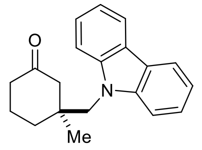

2a

(*S*)-3-((9*H*-carbazol-9-yl)methyl)-3-methylcyclohexan-1-one 2a

Prepared according to general procedure using 9-((dimethyl(phenyl)silyl)methyl)-9*H*-carbazole **4d** (0.15 mmol, 47.3 mg), 3-methyl-2-cyclohexenone (0.1 mmol, 11.0 mg), the aminocatalyst (*R*,*R*)-**3e** (0.02 mmol, 13.3 mg) salicylic acid (0.04 mmol, 5.5 mg), and 0.2 mL of CH_3CN as the solvent. Time of irradiation: 48 hours. The crude mixture was purified by flash column chromatography (hexane/ethyl acetate: gradient from 10:1 to 4:1) to afford the product as a white solid. (21.6 mg, 74% yield, 88% ee). The enantiomeric excess was determined to be 88% by HPLC analysis on a Daicel Chiralpak IC-3: 95/5 hexane/IPA, flow rate 1.0 mL/min, λ = 254 nm: τ_{Major} = 21.2 min, τ_{Minor} = 30.4 min. $[\alpha]_D^{25}$ = +2.95 (c = 0.75, $CHCl_3$, 88% ee).
Absolute configuration determined by X-ray analysis, CCDC 1819014 (see Supplementary Table 1 for details).
^{1}H NMR (500 MHz, $CDCl_3$): δ 8.11 (d, *J* = 7.7 Hz, 2H), 7.51-7.35 (m, 4H), 7.30-7.13 (m, 2H), 4.18 (q, *J* = 15.2 Hz, 2H), 2.49 (d, *J* = 13.4 Hz, 1H), 2.39-2.21 (m, 2H), 2.15-2.04 (m, 1H), 2.02-1.93 (m, 1H), 1.92-1.72 (m, 3H), 1.13 (s, 3H).
^{13}C NMR (126 MHz, $CDCl_3$): δ 211.0, 142.0, 125.9, 123.3, 120.4, 119.4, 109.9, 54.8, 52.6, 43.7, 40.6, 35.3, 23.1, 22.1.
HRMS: Calculated for $C_{20}H_{21}NONa$ (M+Na$^+$): 314.1515, found 314.1503.

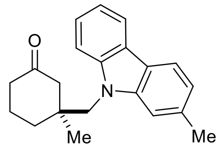

2b

(*S*)-3-methyl-3-((2-Methyl-9*H*-carbazol-9-yl)methyl)cyclohexan-1-one 2b

Prepared according to general procedure using 9-((dimethyl(phenyl)silyl)methyl)-2-methyl-9*H*-carbazole **4e** (0.15 mmol, 49.4 mg), 3-methyl-2-cyclohexenone (0.1 mmol, 11.0 mg), the aminocatalyst (*R*,*R*)-**3e** (0.02 mmol, 13.3 mg) salicylic acid (0.04 mmol, 5.5 mg) and 0.2 mL of CH_3CN as the solvent. Time of irradiation: 48 hours. The crude mixture was purified by flash column chromatography (hexane/ethyl acetate: gradient from 10:1 to 4:1) to afford the product as a colorless oil. (25.1 mg, 82% yield, 84% ee). The enantiomeric excess was determined to be 84% by HPLC analysis on a Daicel Chiralpak IC-3: 90/10 hexane/IPA, flow rate 1.0 mL/min, λ = 254 nm: τ_{Major} = 15.8 min, τ_{Minor} = 12.5 min. $[\alpha]_D^{26}$ = +2.6 (c = 1.48, $CHCl_3$, 84% ee). Absolute configuration determined in comparison to compound **2a**.
^{1}H NMR (500 MHz, $CDCl_3$): δ 8.05 (dt, *J* = 7.7, 0.9 Hz, 1H), 7.97 (d, *J* = 7.9 Hz, 1H), 7.45-7.35 (m, 2H), 7.23 (ddd, *J* = 7.9, 6.8, 1.3 Hz, 1H), 7.19 (s, 1H), 7.08 (dd, *J* = 7.9, 0.7 Hz, 1H), 4.13 (q, *J* = 15.2 Hz, 2H),

2.57 (s, 3H), 2.53-2.45 (m, 1H), 2.36-2.25 (m, 2H), 2.13-2.03 (m, 1H), 2.02-1.94 (m, 1H), 1.91-1.73 (m, 3H), 1.13 (s, 3H).
^{13}C NMR (126 MHz, $CDCl_3$): δ 211.1, 142.5, 140.0, 136.0, 125.3, 123.4, 121.0, 121.0, 120.1, 120.0, 119.3, 110.0, 109.8, 54.7, 52.6, 43.7, 40.6, 35.2, 23.1, 22.5, 22.1.
HRMS: Calculated for $C_{21}H_{23}NNaO$ (M+Na$^+$): 328.1672, found 328.1682.

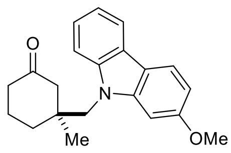

2c

(*S*)-3-((2-methoxy-9*H*-carbazol-9-yl)methyl)-3-methylcyclohexan-1-one 2c
Prepared according to general procedure using 9-((dimethyl(phenyl)silyl)methyl)-2-methoxy-9*H*-carbazole **4f** (0.15 mmol, 51.8 mg), 3-methyl-2-cyclohexenone (0.1 mmol, 11.0 mg), the aminocatalyst (*R*,*R*)-**3e** (0.02 mmol, 13.3 mg), salicylic acid (0.04 mmol, 5.5 mg), and 0.2 mL CH_3CN as the solvent. Time of irradiation: 48 hours. The crude mixture was purified by flash column chromatography (hexane/ethyl acetate: gradient from 10:1 to 4:1) to afford the product as a white solid. (26.4 mg, 82% yield, 87% ee). The enantiomeric excess was determined to be 87% by HPLC analysis on a Daicel Chiralpak IC-3: 90/10 hexane/IPA, flow rate 1.0 mL/min, λ = 254 nm: τ_{Major} = 28.3 min, τ_{Minor} = 20.8 min. $[\alpha]_D^{26}$ = +2.5 (c = 1.40, $CHCl_3$, 87% ee). Absolute configuration determined in comparison to compound **2a**.
^{1}H NMR (500 MHz, $CDCl_3$): δ 8.03-7.90 (m, 2H), 7.40-7.33 (m, 2H), 7.22 (ddd, *J* = 8.0, 6.3, 1.8 Hz, 1H), 6.90-6.83 (m, 2H), 4.10 (q, *J* = 15.2 Hz, 2H), 3.94 (s, 3H), 2.53-2.48 (m, 1H), 2.33-2.25 (m, 2H), 2.15-2.06 (m, 1H), 2.02-1.93 (m, 1H), 1.92-1.75 (m, 3H), 1.12 (s, 3H).
^{13}C NMR (126 MHz, $CDCl_3$): δ 211.0, 159.1, 143.4, 142.1, 124.6, 123.5, 121.0, 119.6, 119.5, 117.3, 109.7, 107.4, 94.8, 55.9, 54.7, 52.6, 43.7, 40.6, 35.4, 23.2, 22.1.
HRMS: Calculated for $C_{21}H_{23}NNaO_2$ (M+Na$^+$): 344.1621, found 344.1619.

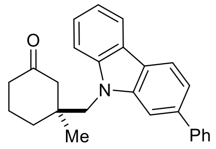

2d

(*S*)-3-methyl-3-((2-phenyl-9*H*-carbazol-9-yl)methyl)cyclohexan-1-one 2d
Prepared according to general procedure using 9-((dimethyl(phenyl)silyl)methyl)-2-phenyl-9*H*-carbazole **4g** (0.15 mmol, 58.7 mg), 3-methyl-2-cyclohexenone (0.1 mmol, 11.0 mg), the aminocatalyst (*R*,*R*)-**3e** (0.02 mmol, 13.3 mg), salicylic acid (0.04 mmol, 5.5 mg), and 0.2 mL of CH_3CN and 40 µL of toluene as solvent mixture. Time of irradiation: 48 hours. The crude mixture was purified by flash column chromatography (hexane/ethyl acetate: gradient from 10:1 to 4:1) to afford the product as a light yellow solid (27.6 mg, 75% yield, 85% ee). The enantiomeric excess was determined to be 86% by HPLC analysis on a Daicel Chiralpak IC-3: 90/10 hexane/IPA, flow rate 1.0 mL/min, λ = 254 nm: τ_{Major} = 22.4 min, τ_{Minor} = 15.4 min. $[\alpha]_D^{26}$ = +2.1 (c = 0.76, $CHCl_3$, 86% ee). Absolute configuration determined in comparison to compound **2a**.
^{1}H NMR (500 MHz, $CDCl_3$): δ 8.18-8.09 (m, 2H), 7.72-7.68 (m, 2H), 7.59 (d, *J* = 1.4 Hz, 1H), 7.53-7.35 (m, 6H), 7.31-7.24 (m, 1H), 4.22 (q, *J* = 15.3 Hz, 2H), 2.57-2.51 (m, 1H), 2.39-2.33 (m, 1H), 2.32-2.26 (m, 1H), 2.16-2.07 (m, 1H), 2.02-1.95 (m, 1H), 1.92-1.80 (m, 3H), 1.15 (s, 3H).
^{13}C NMR (126 MHz, $CDCl_3$): δ 210.9, 142.5, 142.5, 142.2, 139.5, 129.0, 127.7, 127.3, 125.9, 123.1, 122.6, 120.6, 120.4, 119.7, 119.3, 109.9, 108.5, 54.8, 52.6, 43.8, 40.6, 35.3, 23.2, 22.1.
HRMS: Calculated for $C_{26}H_{25}NNaO$ (M+Na$^+$): 390.1828, found 390.1826.

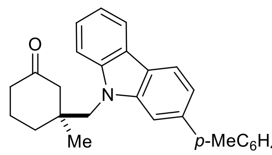

2e

(*S*)-3-((2-(4-methylphenyl)-9*H*-carbazol-9-yl)methyl)-3-methylcyclohexan-1-one 2e
Prepared according to general procedure using 9-((dimethyl(phenyl)silyl)methyl)-2-(4-methylphenyl)-9*H*-carbazole **4h** (0.15 mmol, 60.8 mg), 3-methyl-2-cyclohexenone (0.1 mmol, 11.0 mg), the aminocatalyst (*R*,*R*)-**3e** (0.02 mmol, 13.3 mg), salicylic acid (0.04 mmol, 5.5 mg), 0.2 mL of CH_3CN and 0.2 mL of 1,2-$Cl_2C_6H_4$ as solvent mixture. Time of irradiation: 72 hours. The crude mixture was purified by flash column chromatography (hexane/ethyl acetate: gradient from 10:1 to 4:1) to afford the product as a white solid (36.3 mg, 95% yield, 87% ee). The enantiomeric excess was determined to be 87% by HPLC analysis on a Daicel Chiralpak IC-3: 90/10 hexane/IPA, flow rate 1.0 mL/min, λ = 254

nm: τ_{Major} = 28.7 min, τ_{Minor} = 17.8 min. $[\alpha]_D^{25}$ = +2.09 (c = 1.24, $CHCl_3$, 87% ee). Absolute configuration determined in comparison to compound **2a**.

<u>^{1}H NMR</u> (500 MHz, $CDCl_3$): δ 8.16 – 8.08 (m, 2H), 7.63-7.59 (m, 2H), 7.57 (d, *J* = 1.4 Hz, 1H), 7.50-7.44 (m, 2H), 7.43-7.37 (m, 1H), 7.34-7.29 (m, 2H), 7.29-7.22 (m, 1H), 4.31-4.11 (m, 2H), 2.58-2.51 (m, 1H), 2.44 (s, 3H), 2.38-2.33 (m, 1H), 2.30-2.24 (m, 1H), 2.14-2.06 (m, 1H), 2.01-1.94 (m, 1H), 1.92-1.75 (m, 3H), 1.15 (s, 3H).

<u>^{13}C NMR</u> (126 MHz, $CDCl_3$): δ 211.0, 142.6, 142.5, 139.4, 139.3, 137.1, 133.2, 129.7, 128.0, 127.6, 125.8, 123.2, 122.4, 120.6, 120.4, 119.6, 119.1, 109.9, 108.2, 54.7, 52.6, 43.8, 40.6, 35.3, 23.2, 22.1, 21.3.

<u>HRMS:</u> Calculated for $C_{27}H_{27}NNaO$ (M+Na^+): 404.1985, found 404.1984.

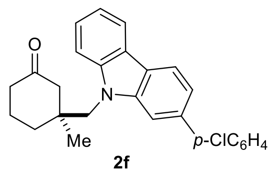

2f

(*S*)-3-((2-(4-chlorophenyl)-9*H*-carbazol-9-yl)methyl)-3-methylcyclohexan-1-one 2f

Prepared according to general procedure using 9-((dimethyl(phenyl)silyl)methyl)-2-(4-chlorophenyl)-9*H*-carbazole **4i** (0.15 mmol, 63.9 mg), 3-methyl-2-cyclohexenone (0.1 mmol, 11.0 mg), the aminocatalyst (*R*,*R*)-**3e** (0.02 mmol, 13.3 mg), salicylic acid (0.04 mmol, 5.5 mg), 0.2 mL of CH_3CN and 40 µL toluene as the solvent mixture. Time of irradiation: 48 hours. The crude mixture was purified by flash column chromatography (hexane/ethyl acetate: gradient from 10:1 to 4:1) to afford the product as a white solid (35.2 mg, 87% yield, 87% ee). The enantiomeric excess was determined to be 87% by HPLC analysis on a Daicel Chiralpak IC-3: 90/10 hexane/IPA, flow rate 1.0 mL/min, λ = 215 nm: τ_{Major} = 22.5 min, τ_{Minor} = 16.4 min. $[\alpha]_D^{25}$ = +1.72 (c = 1.17, $CHCl_3$, 87% ee). Absolute configuration determined in comparison to compound **2a**.

<u>^{1}H NMR</u> (500 MHz, $CDCl_3$): δ 8.16-8.06 (m, 2H), 7.65-7.59 (m, 2H), 7.54 (d, *J* = 1.5 Hz, 1H), 7.49-7.41 (m, 5H), 7.30-7.25 (m, 1H), 4.21 (q, *J* = 15.3 Hz, 2H), 2.54-2.47 (m, 1H), 2.37-2.26 (m, 2H), 2.17-2.07 (m, 1H), 2.01-1.94 (m, 1H), 1.91-1.79 (m, 3H), 1.14 (s, 3H).

<u>^{13}C NMR</u> (126 MHz, $CDCl_3$): δ 210.8, 142.5, 142.5, 140.6, 138.1, 133.4, 129.1, 128.9, 126.1, 123.0, 122.8, 120.7, 120.5, 119.7, 119.0, 110.0, 108.3, 54.6, 52.5, 43.8, 40.6, 35.4, 23.2, 22.1.

<u>HRMS:</u> Calculated for $C_{26}H_{25}ClNO$ (M+H^+): 402.1619, found 402.1621.

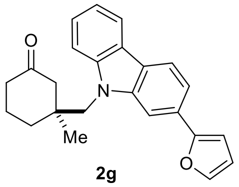

2g

(*S*)-3-((2-(furan-2-yl)-9*H*-carbazol-9-yl)methyl)-3-methylcyclohexan-1-one 2g

Prepared according to general procedure using 9-((dimethyl(phenyl)silyl)methyl)-2-(furan-2-yl)-9*H*-carbazole **4j** (0.15 mmol, 57.2 mg), 3-methyl-2-cyclohexenone (0.1 mmol, 11.0 mg), the aminocatalyst (*R*,*R*)-**3e** (0.02 mmol, 13.3 mg), salicylic acid (0.04 mmol, 5.5 mg), and 0.2 mL of CH_3CN as the solvent. Time of irradiation: 72 hours. The crude mixture was purified by flash column chromatography (hexane/ethyl acetate: gradient from 10:1 to 4:1) to afford the product as a yellow oil (15.8 mg, 44% yield, 90% ee). The enantiomeric excess was determined to be 90% by HPLC analysis on a Daicel Chiralpak IC-3: 90/10 hexane/IPA, flow rate 1.0 mL/min, λ = 254 nm: τ_{Major} = 23.6 min, τ_{Minor} = 17.2 min. $[\alpha]_D^{25}$ = +1.47 (c = 0.77, $CHCl_3$, 90% ee). Absolute configuration determined in comparison to compound **2a**.

^{1}H NMR (500 MHz, $CDCl_3$): δ 8.07 (dd, *J* = 7.9, 1.7 Hz, 2H), 7.76-7.69 (m, 1H), 7.60-7.51 (m, 2H), 7.48-7.36 (m, 2H), 7.31-7.15 (m, 1H), 6.76 (dd, *J* = 3.3, 0.7 Hz, 1H), 6.53 (dd, *J* = 3.3, 1.8 Hz, 1H), 4.21 (q, *J* = 15.3 Hz, 2H), 2.52 (d, *J* = 13.2 Hz, 1H), 2.36 (dt, *J* = 13.2, 2.1 Hz, 1H), 2.31-2.25 (m, 1H), 2.15-2.07 (m, 1H), 2.01-1.94 (m, 1H), 1.90-1.78 (m, 3H), 1.15 (s, 3H).

<u>^{13}C NMR</u> (126 MHz, $CDCl_3$): δ 211.0, 154.9, 142.5, 142.3, 142.1, 128.8, 125.9, 123.2, 122.5, 120.6, 120.3, 119.7, 116.1, 112.0, 109.9, 105.2, 105.0, 54.7, 52.6, 43.8, 40.7, 35.3, 23.2, 22.1.

<u>HRMS:</u> Calculated for $C_{24}H_{24}NO_2$ (M+H^+): 358.1802, found 358.1804.

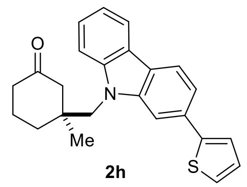
2h

(*S*)-3-methyl-3-((2-(thiophen-2-yl)-9*H*-carbazol-9-yl)methyl)cyclohexan-1-one 2h

Prepared according to general procedure using 9-((dimethyl(phenyl)silyl)methyl)-2-(thiophen-2-yl)-9*H*-carbazole **4k** (0.15 mmol, 59.6 mg), 3-methyl-2-cyclohexenone (0.1 mmol, 11.0 mg), the aminocatalyst (*R,R*)-**3e** (0.02 mmol, 13.3 mg), salicylic acid (0.04 mmol, 5.5 mg), and 0.2 mL of CH_3CN and 0.1 mL 1,2-$Cl_2C_6H_4$ as the solvent mixture. Time of irradiation: 72 hours. The crude mixture was purified by flash column chromatography (hexane/ethyl acetate: gradient from 10:1 to 4:1) to afford the product as a white solid (28.1 mg, 75% yield, 90% ee). The enantiomeric excess was determined to be 90% by HPLC analysis on a Daicel Chiralpak IC-3: 90/10 hexane/IPA, flow rate 1.0 mL/min, λ = 254 nm: τ_{Major} = 29.7 min, τ_{Minor} = 19.9 min. $[\alpha]_D^{25}$ = +2.0 (c = 1.55, $CHCl_3$, 90% ee). Absolute configuration determined in comparison to compound **2a**.

^{1}H NMR (500 MHz, $CDCl_3$): δ 8.09-8.05 (m, 2H), 7.61 (d, *J* = 1.4 Hz, 1H), 7.53 (dd, *J* = 8.1, 1.5 Hz, 1H), 7.45 (ddd, *J* = 8.3, 7.0, 1.2 Hz, 1H), 7.43-7.38 (m, 2H), 7.32 (dd, *J* = 5.1, 1.1 Hz, 1H), 7.26 (td, *J* = 7.4, 1.0 Hz, 1H), 7.14 (dd, *J* = 5.1, 3.6 Hz, 1H), 4.28-4.08 (m, 2H), 2.55-2.49 (m, 1H), 2.38-2.22 (m, 2H), 2.16-2.07 (m, 1H), 2.02-1.94 (m, 1H), 1.93-1.71 (m, 3H), 1.14 (s, 3H).

^{13}C NMR (126 MHz, $CDCl_3$): δ 210.8, 145.6, 142.5, 142.4, 132.4, 128.3, 126.0, 124.8, 123.3, 123.1, 122.8, 120.7, 120.4, 119.7, 118.2, 109.9, 107.2, 54.6, 52.5, 43.7, 40.6, 35.3, 23.2, 22.1.

HRMS: Calculated for $C_{24}H_{23}NNaOS$ $(M+Na^+)$: 396.1393, found 396.1393.

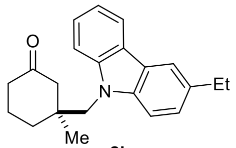
2i

(*S*)-3-methyl-3-((3-ethyl-9*H*-carbazol-9-yl)methyl)cyclohexan-1-one 2i

Prepared according to general procedure using 9-((dimethyl(phenyl)silyl)methyl)-3-ethyl-9*H*-carbazole **4l** (0.15 mmol, 51.5 mg), 3-methyl-2-cyclohexenone (0.1 mmol, 11.0 mg), the aminocatalyst (*R,R*)-**3e** (0.02 mmol, 13.3 mg) salicylic acid (0.04 mmol, 5.5 mg), and 0.2 mL of CH_3CN as solvent. Time of irradiation: 48 hours. The crude mixture was purified by flash column chromatography (hexane/ethyl acetate: gradient from 10:1 to 4:1) to afford the product as a white solid (27.2 mg, 85% yield, 88% ee). The enantiomeric excess was determined to be 88% by HPLC analysis on a Daicel Chiralpak IC-3: 90/10 hexane/IPA, flow rate 1.0 mL/min, λ = 254 nm: τ_{Major} = 14.70 min, τ_{Minor} = 11.19 min. $[\alpha]_D^{25}$ = +3.0 (c = 1.38, $CHCl_3$, 88% ee). Absolute configuration determined in comparison to compound **2a**.

^{1}H NMR (500 MHz, $CDCl_3$): δ 8.09 (d, *J* = 7.7 Hz, 1H), 7.93 (d, *J* = 0.8 Hz, 1H), 7.46-7.42 (m, 1H), 7.40-7.36 (m, 1H), 7.35-7.28 (m, 2H), 7.23 (ddd, *J* = 7.9, 7.0, 1.1 Hz, 1H), 4.14 (q, *J* = 15.2 Hz, 2H), 2.85 (q, *J* = 7.6 Hz, 2H), 2.55-2.44 (m, 1H), 2.37-2.21 (m, 2H), 2.12-2.04 (m, 1H), 2.00-1.93 (m, 1H), 1.90-1.75 (m, 3H), 1.36 (t, *J* = 7.6 Hz, 3H), 1.12 (s, 3H).

^{13}C NMR (126 MHz, $CDCl_3$): δ 211.1, 142.2, 140.5, 135.5, 126.1, 125.7, 123.4, 123.2, 120.3, 119.2, 119.1, 109.8, 109.7, 54.9, 52.6, 43.7, 40.6, 35.2, 29.0, 23.1, 22.1, 16.6.

HRMS: Calculated for $C_{22}H_{25}NNaO$ $(M+Na^+)$: 342.1828, found 342.1843.

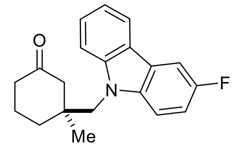
2j

(*S*)-3-((3-fluoro-9*H*-carbazol-9-yl)methyl)-3-methylcyclohexan-1-one 2j

Prepared according to general procedure using 9-((dimethyl(phenyl)silyl)methyl)-3-fluoro-9*H*-carbazole **4m** (0.15 mmol, 50.0 mg), 3-methyl-2-cyclohexenone (0.1 mmol, 11.0 mg), the aminocatalyst (*R,R*)-**3e** (0.02 mmol, 13.3 mg), salicylic acid (0.04 mmol, 5.5 mg), and 0.3 mL of CH_3CN as solvent. Time of irradiation: 48 hours. The crude mixture was purified by flash column chromatography (hexane/ethyl acetate: gradient from 10:1 to 4:1) to afford the product as a white solid (24.3 mg, 78% yield, 86% ee). The enantiomeric excess was determined to be 86% by HPLC analysis on a Daicel Chiralpak IC-3: 90/10 hexane/IPA, flow rate 1.0 mL/min, λ = 254 nm: τ_{Major} = 18.5 min, τ_{Minor} = 14.3 min. $[\alpha]_D^{25}$ = +3.3 (c = 1.27, $CHCl_3$, 86% ee). Absolute configuration determined in comparison to compound **2a**.

^{1}H NMR (500 MHz, $CDCl_3$): δ 8.06-8.00 (m, 1H), 7.74 (dd, *J* = 8.7, 2.5 Hz, 1H), 7.49-7.45 (m, 1H), 7.42-7.38 (m, 1H), 7.34-7.30 (m, 1H), 7.27-7.22 (m, 1H), 7.19 (td, *J* = 9.0, 2.6 Hz, 1H), 4.23-4.02 (m, 2H), 2.49-2.42 (m, 1H), 2.33-2.25 (m, 2H), 2.16-2.05 (m, 1H), 2.04-1.92 (m, 1H), 1.93-1.72 (m, 3H), 1.11 (s, 3H).

^{19}F NMR (376 MHz, $CDCl_3$): δ -124.8.
^{13}C NMR (126 MHz, $CDCl_3$): δ 210.7, 157.5 (d, *J* = 236.3 Hz), 142.8, 138.3, 126.5, 123.8 (d, *J* = 9.4 Hz), 122.8 (d, *J* = 4.1 Hz), 120.6, 119.4, 113.6 (d, *J* = 25.4 Hz), 110.4 (d, *J* = 8.9 Hz), 110.1, 106.1 (d, *J* = 23.7 Hz), 54.9, 52.5, 43.7, 40.6, 35.3, 23.1, 22.1.
HRMS: Calculated for $C_{20}H_{20}FNNaO$ (M+Na$^+$): 332.1424, found 332.1421.

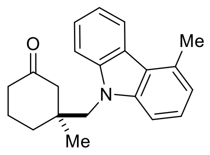
2k

(*S*)-3-methyl-3-((4-methyl-9*H*-carbazol-9-yl)methyl)cyclohexan-1-one 2k
Prepared according to general procedure using 9-((dimethyl(phenyl)silyl)methyl)-4-methyl-9*H*-carbazole **4n** (0.15 mmol, 49.4 mg), 3-methyl-2-cyclohexenone (0.1 mmol, 11.0 mg), the aminocatalyst (*R*,*R*)-**3e** (0.02 mmol, 13.3 mg) salicylic acid (0.04 mmol, 5.5 mg) and 0.2 mL of CH_3CN as the solvent. Time of irradiation: 48 hours. The crude mixture was purified by flash column chromatography (hexane/ethyl acetate: gradient from 10:1 to 4:1) to afford the product as a white solid (25.0 mg, 82% yield, 88% ee). The enantiomeric excess was determined to be 88% by HPLC analysis on a Daicel Chiralpak IC-3: 90/10 hexane/IPA, flow rate 1.0 mL/min, λ = 254 nm: τ_{Major} = 17.2 min, τ_{Minor} = 12.9 min. $[\alpha]_D^{26}$ = +4.0 (c = 1.09, $CHCl_3$, 88% ee). Absolute configuration determined in comparison to compound **2a**.
^{1}H NMR (500 MHz, $CDCl_3$): δ 8.21 (dt, *J* = 7.9, 0.9 Hz, 1H), 7.49-7.41 (m, 2H), 7.36 (dd, *J* = 8.3, 7.2 Hz, 1H), 7.30-7.25 (m, 2H), 7.04 (dt, *J* = 7.2, 0.9 Hz, 1H), 4.26-4.13 (q, *J* = 15.0 Hz, 2H), 2.90 (s, 3H), 2.50-2.46 (m, 1H), 2.38-2.18 (m, 2H), 2.13-2.03 (m, 1H), 2.01-1.91 (m, 1H), 1.91-1.76 (m, 3H), 1.13 (s, 3H).
^{13}C NMR (126 MHz, $CDCl_3$): δ 211.0, 142.1, 141.9, 133.6, 125.6, 125.2, 123.9, 122.8, 121.7, 121.1, 119.4, 109.6, 107.5, 54.7, 52.6, 43.8, 40.6, 35.3, 23.2, 22.1, 21.1.
HRMS: Calculated for $C_{21}H_{23}NNaO$ (M+Na$^+$): 328.1672, found 328.1674.

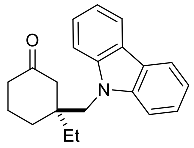
2l

(*S*)-3-((9*H*-carbazol-9-yl)methyl)-3-ethylcyclopentan-1-one 2l
Prepared according to general procedure using 9-((dimethyl(phenyl)silyl)methyl)-9*H*-carbazole **4d** (0.15 mmol, 47.3 mg), 3-ethylcyclohex-2-en-1-one (0.1 mmol, 12.4 mg), the aminocatalyst (*R*,*R*)-**3e** (0.02 mmol, 13.3 mg), salicylic acid (0.04 mmol, 5.5 mg), and 0.2 mL of CH_3CN as the solvent. Time of irradiation: 48 hours. The crude mixture was purified by flash column chromatography (DCM/ethyl acetate: gradient from 100:0 to 50:1) to afford the product as a white solid (23.2 mg, 76% yield, 67% ee). The enantiomeric excess was determined to be 67% by HPLC analysis on a Daicel Chiralpak IC-3: 90/10 hexane/IPA, flow rate 1.0 mL/min, λ = 254 nm: τ_{Major} = 15.5 min, τ_{Minor} = 12.5 min. $[\alpha]_D^{25}$ = +3.92 (c = 1.09, $CHCl_3$, 67% ee). Absolute configuration determined in comparison to compound **2a**.
^{1}H NMR (500 MHz, $CDCl_3$): δ 8.11 (d, *J* = 7.7 Hz, 2H), 7.46 (ddd, *J* = 8.3, 7.0, 1.2 Hz, 2H), 7.40 (d, *J* = 8.2 Hz, 2H), 7.26 (td, *J* = 7.8, 7.4, 1.0 Hz, 2H), 4.30 (d, *J* = 15.3 Hz, 1H), 4.11 (d, *J* = 15.3 Hz, 1H), 2.40 (s, 2H), 2.27-2.17 (m, 1H), 2.02-1.86 (m, 3H), 1.84-1.66 (m, 2H), 1.66-1.49 (m, 2H), 1.10 (t, *J* = 7.5 Hz, 3H).
^{13}C NMR (126 MHz, $CDCl_3$): δ 211.4, 142.1, 125.9, 123.3, 120.4, 119.5, 109.8, 51.8, 51.4, 45.8, 40.4, 31.6, 28.1, 21.5, 7.9. HRMS: Calculated for $C_{21}H_{23}NNaO$ (M+Na$^+$): 328.1672, found 328.1668.

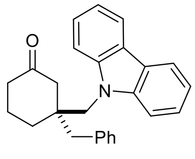
2m

(*R*)-3-((9H-carbazol-9-yl)methyl)-3-benzylcyclohexan-1-one 2m
Prepared according to general procedure using 9-((dimethyl(phenyl)silyl)methyl)-9*H*-carbazole **4d** (0.15 mmol, 47.3 mg), 3-benzylcyclohex-2-en-1-one (0.1 mmol, 18.6 mg), the aminocatalyst (*R*,*R*)-**3e** (0.02 mmol, 13.3 mg), salicylic acid (0.04 mmol, 5.5 mg), and 0.2 mL of CH_3CN as the solvent. Time of irradiation: 48 hours. The crude mixture was purified by flash column chromatography (DCM/ethyl acetate: gradient from 100:0 to 50:1) to afford the product as a white solid (22.0 mg, 60% yield, 50% ee). The enantiomeric excess was determined to be 50% by HPLC analysis on a Daicel Chiralpak IC-3: 90/10 hexane/IPA, flow rate 1.0 mL/min, λ = 254 nm: τ_{Major} = 14.5 min, τ_{Minor} = 13.7 min. $[\alpha]_D^{25}$ = +37.2 (c = 0.12, $CHCl_3$, 50% ee). Absolute configuration determined in comparison to compound **2a**.

^{1}H NMR (500 MHz, $CDCl_3$): δ 8.08 (dd, *J* = 7.7, 1.2 Hz, 2H), 7.46-7.37 (m, 4H), 7.36-7.29 (m, 3H), 7.29-7.20 (m, 2H), 7.19-7.14 (m, 2H), 4.31-4.07 (m, 2H), 2.99-2.75 (m, 2H), 2.60-2.46 (m, 1H), 2.39 (d, *J* = 13.4 Hz, 1H), 2.32-2.21 (m, 1H), 2.12-1.91 (m, 4H), 1.91-1.80 (m, 1H).
^{13}C NMR (126 MHz, $CDCl_3$): δ 211.6, 142.1, 136.5, 131.0, 128.7, 127.1, 125.9, 123.4, 120.4, 119.5, 109.8, 52.0, 50.1, 47.1, 42.3, 40.4, 33.2, 21.8.
HRMS: Calculated for $C_{26}H_{25}NNaO$ (M+Na^+): 390.1828, found 390.1809.

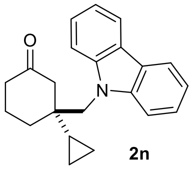
2n

(*S*)-3-((9*H*-carbazol-9-yl)methyl)-3-cyclopropylcyclohexan-1-one 2n
Prepared according to general procedure using 9-((dimethyl(phenyl)silyl)methyl)-9*H*-carbazole **4d** (0.15 mmol, 47.3 mg), 3-cyclopropylcyclohex-2-en-1-one (0.1 mmol, 13.6 mg), the aminocatalyst (*R*,*R*)-**3e** (0.02 mmol, 13.3 mg), salicylic acid (0.04 mmol, 5.5 mg), and 0.2 mL of CH_3CN as the solvent. Time of irradiation: 72 hours. The crude mixture was purified by flash column chromatography (hexane/ethyl acetate: gradient from 10:1 to 4:1) to afford the product as a colorless oil (24.8 mg, 78% yield, 56% ee). The enantiomeric excess was determined to be 56% by HPLC analysis on a Daicel Chiralpak IC-3: 90/10 hexane/IPA, flow rate 1.0 mL/min, λ = 215 nm: τ_{Major} = 22.5 min, τ_{Minor} = 13.1 min. $[\alpha]_D^{25}$ = +2.0 (c = 1.20, $CHCl_3$, 56% ee). Absolute configuration determined in comparison to compound **2a**
^{1}H NMR (500 MHz, $CDCl_3$): δ 8.10 (dt, *J* = 7.8, 1.0 Hz, 2H), 7.55-7.37 (m, 4H), 7.29-7.10 (m, 2H), 4.26-4.12 (m, 2H), 2.34-2.26 (m, 1H), 2.27-2.20 (m, 1H), 2.17-2.08 (m, 2H), 2.07-1.98 (m, 1H), 1.97-1.86 (m, 3H), 0.85-0.77 (m, 1H), 0.58-0.51 (m, 1H), 0.49-0.39 (m, 3H).
^{13}C NMR (126 MHz, $CDCl_3$): δ 211.8, 142.1, 125.8, 123.3, 120.4, 119.4, 109.9, 53.1, 46.6, 44.2, 40.6, 35.8, 21.3, 19.0, 2.9, -0.6.
HRMS: Calculated for $C_{22}H_{23}NNaO$ (M+Na^+): 340.1672, found 340.1688.

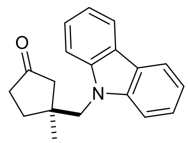
2o

(*S*)-3-((9*H*-carbazol-9-yl)methyl)-3-methylcyclopentan-1-one 2o
Prepared according to general procedure using 9-((dimethyl(phenyl)silyl)methyl)-9*H*-carbazole **4d** (0.15 mmol, 47.3 mg), 3-methylcyclopent-2-en-1-one (0.1 mmol, 9.6 mg), the aminocatalyst (*R*,*R*)-**3e** (0.02 mmol, 13.3 mg), benzoic acid (0.04 mmol, 4.9 mg), and 0.2 mL of CH_3CN as the solvent. Time of irradiation: 96 hours. The crude mixture was purified by flash column chromatography (DCM/ethyl acetate: gradient from 100:0 to 50:1) to afford the product as a white solid (12.5 mg, 45% yield, 74% ee). The enantiomeric excess was determined to be 74% ee by HPLC analysis on a Daicel Chiralpak ID-3: 90/10 hexane/IPA, flow rate 1.0 mL/min, λ = 254 nm: τ_{Major} = 15.2 min, τ_{Minor} = 12.0 min. $[\alpha]_D^{24}$ = -4.0 (c = 0.63, $CHCl_3$, 74% ee). Absolute configuration determined in comparison to compound **2a**.
^{1}H NMR (500 MHz, $CDCl_3$): δ 8.10 (dt, *J* = 7.8, 0.9 Hz, 2H), 7.46 (ddd, *J* = 8.3, 7.1, 1.2 Hz, 2H), 7.38 (dt, *J* = 8.3, 0.8 Hz, 2H), 7.26 (ddd, *J* = 7.9, 6.4, 1.0 Hz, 2H), 4.32 (s, 2H), 2.44-2.01 (m, 5H), 1.95-1.86 (m, 1H), 1.26 (s, 3H).
^{13}C NMR (126 MHz $CDCl_3$): δ 217.7, 141.8, 126.0, 123.3, 120.5, 119.5, 109.4, 51.6, 50.7, 44.0, 36.5, 33.7, 25.0. HRMS: Calculated for $C_{19}H_{19}NNaO$ (M+Na^+): 300.1359, found 300.1368.

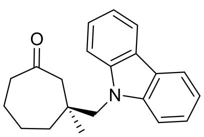
2p

(*S*)-3-((9*H*-carbazol-9-yl)methyl)-3-methylcycloheptan-1-one 2p
Prepared according to general procedure using 9-((dimethyl(phenyl)silyl)methyl)-9*H*-carbazole **4d** (0.15 mmol, 47.3 mg), 3-methylcyclohept-2-en-1-one (0.1 mmol, 12.4 mg), the aminocatalyst (*R*,*R*)-**3e** (0.02 mmol, 13.3 mg), benzoic acid (0.04 mmol, 5.5 mg), and 0.2 mL of CH_3CN as the solvent. Time of irradiation: 48 hours. The crude mixture was purified by flash column chromatography (DCM/ethyl acetate: gradient from 100:0 to 50:1) to afford the product as a colorless oil (22.6 mg, 74% yield, 95% ee). The enantiomeric excess was determined to be 95% by HPLC analysis on a Daicel Chiralpak IC-3: 90/10 hexane/IPA, flow rate 1.0 mL/min, λ = 254 nm: τ_{Major} = 20.4 min, τ_{Minor} = 14.0 min. $[\alpha]_D^{23}$ = -3.2 (c = 0.87, $CHCl_3$, 95% ee). Absolute configuration determined in comparison to compound **2a**.

^{1}H NMR (500 MHz, $CDCl_3$): δ 8.09 (d, *J* = 7.8 Hz, 2H), 7.48-7.40 (m, 4H), 7.27-7.21 (m, 2H), 4.15 (s, 2H), 2.90 (d, *J* = 11.7 Hz, 1H), 2.59-2.44 (m, 2H), 2.35 (ddd, *J* = 18.0, 11.5, 4.6 Hz, 1H), 1.95-1.75 (m, 4H), 1.70-1.58 (m, 1H), 1.52-1.41 (m, 1H), 1.10 (s, 3H).
^{13}C NMR (126 MHz, $CDCl_3$): δ 212.9, 142.1, 125.7, 123.4, 120.3, 119.3, 110.1, 55.9, 53.4, 44.1, 41.7, 40.4, 24.3, 24.1, 23.7. HRMS: Calculated for $C_{21}H_{23}NNaO$ ($M+Na^+$): 328.1672, found 328.1666.

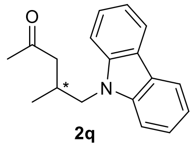
2q

5-(9*H*-carbazol-9-yl)-4-methylpentan-2-one 2q
Prepared according to general procedure using 9-((dimethyl(phenyl)silyl)methyl)-9*H*-carbazole **4d** (0.15 mmol, 47.3 mg), (*E*)-pent-3-en-2-one (0.1 mmol, 8.4 mg), the aminocatalyst (*R*,*R*)-**3e** (0.02 mmol, 13.3 mg), salicylic acid (0.04 mmol, 5.5 mg), and 0.2 mL of CH_3CN as the solvent. Time of irradiation: 48 hours. The crude mixture was purified by flash column chromatography (hexane/ethyl acetate: gradient from 10:1 to 4:1) to afford the product as a colorless oil (20.6 mg, 78% yield, 55% ee). The enantiomeric excess was determined to be 55% by HPLC analysis on a Daicel Chiralpak IC-3: 90/10 hexane/IPA, flow rate 1.0 mL/min, λ = 254 nm: τ_{Major} = 9.0 min, τ_{Minor} = 10.1 min. $[\alpha]_D^{25}$ = +15.4 (c = 0.10, $CHCl_3$, 55% ee).
^{1}H NMR (300 MHz, $CDCl_3$): δ 8.10 (d, *J* = 7.8 Hz, 2H), 7.54-7.39 (m, 4H), 7.33-7.16 (m, 2H), 4.28 (dd, *J* = 14.6, 6.9 Hz, 1H), 4.07 (dd, *J* = 14.6, 8.0 Hz, 1H), 2.81 (dq, *J* = 13.9, 6.8 Hz, 1H), 2.61-2.33 (m, 2H), 2.08 (s, 3H), 0.99 (d, *J* = 6.8 Hz, 3H).
^{13}C NMR (101 MHz, $CDCl_3$): δ 207.7, 140.9, 125.9, 123.0, 120.4, 119.1, 109.2, 48.7, 48.3, 30.6, 29.7, 18.5. HRMS: Calculated for $C_{18}H_{20}NO$ ($M+H^+$): 266.1539, found 266.1533.

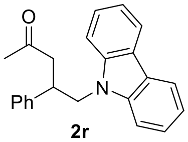
2r

5-(9*H*-carbazol-9-yl)-4-phenylpentan-2-one 2r
Prepared according to general procedure using 9-((dimethyl(phenyl)silyl)methyl)-9*H*-carbazole **4d** (0.15 mmol, 47.3 mg), (*E*)-4-phenylbut-3-en-2-one (0.1 mmol, 8.4 mg), the aminocatalyst (*R*,*R*)-**3e** (0.02 mmol, 13.3 mg), salicylic acid (0.04 mmol, 5.5 mg), and 0.2 mL of CH_3CN as the solvent. Time of irradiation: 48 hours. The crude mixture was purified by flash column chromatography (hexane/ethyl acetate: gradient from 10:1 to 4:1) to afford the product as a colorless oil (21.9 mg, 68% yield, racemic). The enantiomeric excess was determined to be 0% by HPLC analysis on a Daicel Chiralpak IC-3: 90/10 hexane/IPA, flow rate 1.0 mL/min, λ = 215 nm: τ_1 = 10.5 min, τ_2 = 19.9 min.
^{1}H NMR (400 MHz, $CDCl_3$): δ 8.06 (dt, *J* = 7.7, 1.0 Hz, 2H), 7.40 (ddd, *J* = 8.3, 7.0, 1.2 Hz, 2H), 7.32 (dt, *J* = 8.3, 0.9 Hz, 2H), 7.25-7.11 (m, 7H), 4.54 (dd, *J* = 14.6, 7.5 Hz, 1H), 4.33 (dd, *J* = 14.6, 7.3 Hz, 1H), 3.93 (p, *J* = 7.2 Hz, 1H), 2.96 (dd, *J* = 17.4, 6.9 Hz, 1H), 2.85 (dd, *J* = 17.4, 7.2 Hz, 1H), 2.01 (s, 3H).
^{13}C NMR (101 MHz, $CDCl_3$): δ 206.7, 141.7, 140.7, 128.9, 127.7, 127.3, 125.8, 122.9, 120.3, 119.1, 109.1, 49.4, 46.6, 40.7, 30.5. HRMS: Calculated for $C_{23}H_{21}NONa$ ($M+Na^+$): 350.1515, found 350.1519.

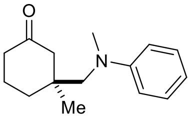
10a

(*S*)-3-methyl-3-((methyl(phenyl)amino)methyl)cyclohexan-1-one 10a
Prepared according to general procedure using *N*-((dimethyl(phenyl)silyl)methyl)-*N*-methylaniline **9a** (0.15 mmol, 38.3 mg), 3-methyl-2-cyclohexenone (0.1 mmol, 11.0 mg), the aminocatalyst (*R*,*R*)-**3e** (0.02 mmol, 13.3 mg), benzoic acid (0.04 mmol, 4.9 mg), and 0.2 mL of CH_3CN as the solvent. Time of irradiation: 48 hours. The crude mixture was purified by flash column chromatography (hexane/ethyl acetate: gradient from 10:1 to 4:1) to afford the product as a colorless oil (19.0 mg, 82% yield, 80% ee). The enantiomeric excess was determined to be 80% by HPLC analysis on a Daicel Chiralpak IC-3: 90/10 hexane/IPA, flow rate 1.0 mL/min, λ = 254 nm: τ_{Major} = 17.2 min, τ_{Minor} = 12.3 min. $[\alpha]_D^{25}$ = +35.2 (c = 0.11, $CHCl_3$, 80% ee). This compound is known, the spectroscopic data matched with previous report.[1]
^{1}H NMR (500 MHz, $CDCl_3$): δ 7.24-7.19 (m, 2H), 6.78-6.74 (m, 2H), 6.73-6.67 (m, 1H), 3.23 (d, *J* = 2.6 Hz, 2H), 3.01 (s, 3H), 2.46 (d, *J* = 13.2 Hz, 1H), 2.36-2.28 (m, 1H), 2.27-2.19 (m, 1H), 2.15 (dt, *J* = 13.2, 2.1 Hz, 1H), 2.04-1.94 (m, 1H), 1.92-1.76 (m, 2H), 1.68-1.59 (m, 1H), 0.97 (s, 3H).
^{13}C NMR (126 MHz, $CDCl_3$): δ 211.9, 150.6, 129.1, 116.5, 112.2, 63.9, 52.3, 43.4, 42.2, 41.0, 34.9, 23.0, 22.3.

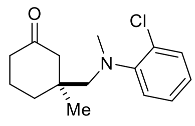

10b

(*S*)-3-(((2-chlorophenyl)(methyl)amino)methyl)-3-methylcyclohexan-1-one 10b
Prepared according to general procedure using 2-chloro-*N*-((dimethyl(phenyl)silyl)methyl)-*N*-methylaniline **9b** (0.20 mmol, 58.0 mg), 3-methyl-2-cyclohexenone (0.1 mmol, 11.0 mg), the aminocatalyst (*R,R*)-**3e** (0.02 mmol, 13.3 mg), benzoic acid (0.04 mmol, 4.9 mg), and 0.2 mL of CH_3CN as the solvent. Time of irradiation: 72 hours. The crude mixture was purified by flash column chromatography (hexane/ethyl acetate: gradient from 10:1 to 4:1) to afford the product as a colorless oil (16.6 mg, 62% yield, 63% ee). The enantiomeric excess was determined to be 63% by HPLC analysis on a Daicel Chiralpak IC-3: 95/5 hexane/IPA, flow rate 1.0 mL/min, λ = 254 nm: τ_{Major} = 12.8 min, τ_{Minor} = 12.3 min. $[\alpha]_D^{25}$ = +38.5 (c = 0.11, $CHCl_3$, 63% ee).
^{1}H NMR (500 MHz, $CDCl_3$): δ 7.35 (dd, *J* = 8.0, 1.5 Hz, 1H), 7.24 (dd, *J* = 8.1, 1.6 Hz, 1H), 7.18 (td, *J* = 7.7, 1.5 Hz, 1H), 7.01-6.92 (m, 1H), 3.04 (d, *J* = 1.4 Hz, 2H), 2.82 (s, 3H), 2.43 (d, *J* = 13.4 Hz, 1H), 2.31-2.16 (m, 2H), 1.97 (dt, *J* = 13.4, 1.8 Hz, 1H), 1.90-1.68 (m, 3H), 1.46-1.34 (m, 1H), 0.87 (s, 3H).
^{13}C NMR (126 MHz, $CDCl_3$): δ 212.7, 151.7, 131.3, 130.8, 127.7, 125.0, 124.6, 66.1, 51.6, 47.0, 43.0, 41.1, 33.9, 23.4, 22.3.
HRMS: Calculated for $C_{15}H_{20}ClNNaO$ (M+Na$^+$): 288.1126, found 288.1119.

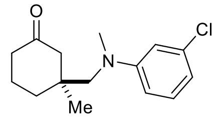

10c

(*S*)-3-(((3-chlorophenyl)(methyl)amino)methyl)-3-methylcyclohexan-1-one 10c
Prepared according to general procedure using 3-chloro-*N*-((dimethyl(phenyl)silyl)methyl)-*N*-methylaniline **9c** (0.15 mmol, 43.4 mg), 3-methyl-2-cyclohexenone (0.1 mmol, 11.0 mg), the aminocatalyst (*R,R*)-**3e** (0.02 mmol, 13.3 mg), benzoic acid (0.04 mmol, 4.9 mg), and 0.2 mL of CH_3CN as the solvent. Time of irradiation: 48 hours. The crude mixture was purified by flash column chromatography (hexane/ethyl acetate: gradient from 10:1 to 4:1) to afford the product as a colorless oil (21.0 mg, 79% yield, 84% ee). The enantiomeric excess was determined to be 84% by HPLC analysis on a Daicel Chiralpak IC-3: 90/10 hexane/IPA, flow rate 1.0 mL/min, λ = 254 nm: τ_{Major} = 15.5 min, τ_{Minor} = 11.9 min. $[\alpha]_D^{25}$ = +30.9 (c = 0.10, $CHCl_3$, 84% ee).
^{1}H NMR (500 MHz, $CDCl_3$): δ 7.10 (dd, *J* = 8.5, 7.8 Hz, 1H), 6.70 (dd, *J* = 2.6, 1.9 Hz, 1H), 6.66 (ddd, *J* = 7.8, 1.9, 0.8 Hz, 1H), 6.61 (ddd, *J* = 8.5, 2.7, 0.8 Hz, 1H), 3.26-3.17 (m, 2H), 3.00 (s, 3H), 2.44-2.37 (m, 1H), 2.35-2.29 (m, 1H), 2.27-2.19 (m, 1H), 2.14 (dt, *J* = 13.2, 2.1 Hz, 1H), 2.04-1.94 (m, 1H), 1.91-1.73 (m, 2H), 1.66-1.61 (m, 1H), 0.96 (s, 3H).
^{13}C NMR (126 MHz, $CDCl_3$): δ 211.5, 151.6, 135.1, 130.0, 116.4, 112.2, 110.4, 63.6, 52.2, 43.4, 42.2, 40.9, 35.0, 23.0, 22.2. HRMS: Calculated for $C_{15}H_{21}NOCl$ (M+H$^+$): 358.1802, found 358.1804.

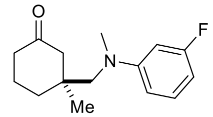

10d

(*S*)-3-(((3-fluorophenyl)(methyl)amino)methyl)-3-methylcyclohexan-1-one 10d
Prepared according to general procedure using 3-fluoro-*N*-((dimethyl(phenyl)silyl)methyl)-*N*-methylaniline **9d** (0.15 mmol, 41.0 mg), 3-methyl-2-cyclohexenone (0.1 mmol, 11.0 mg), the aminocatalyst (*R,R*)-**3e** (0.02 mmol, 13.3 mg), benzoic acid (0.04 mmol, 4.9 mg), and 0.2 mL of CH_3CN as the solvent. Time of irradiation: 48 hours. The crude mixture was purified by flash column chromatography (hexane/ethyl acetate: gradient from 10:1 to 4:1) to afford the product as a colorless oil (20.2 mg, 81% yield, 79% ee). The enantiomeric excess was determined to be 79% by HPLC analysis on a Daicel Chiralpak IC-3: 90/10 hexane/IPA, flow rate 1.0 mL/min, λ = 254 nm: τ_{Major} = 16.6 min, τ_{Minor} = 12.8 min. $[\alpha]_D^{25}$ = +24.3 (c = 0.11, $CHCl_3$, 79% ee).
^{1}H NMR (500 MHz, $CDCl_3$): δ 7.13 (td, *J* = 8.2, 7.2 Hz, 1H), 6.51-6.47 (m, 1H), 6.44-6.35 (m, 2H), 3.22 (d, *J* = 1.9 Hz, 2H), 3.00 (s, 3H), 2.42 (d, *J* = 13.2 Hz, 1H), 2.35-2.29 (m, 1H), 2.27-2.19 (m, 1H), 2.14 (dt, *J* = 13.2, 2.1 Hz, 1H), 2.04-1.95 (m, 1H), 1.92-1.74 (m, 2H), 1.65-1.60 (m, 1H), 0.96 (s, 3H).
^{13}C NMR (126 MHz, $CDCl_3$): δ 211.6, 164.1 (d, *J* = 241.6 Hz), 152.3 (d, *J* = 10.6 Hz), 130.1 (d, *J* = 10.5 Hz), 107.9 (d, *J* = 2.2 Hz), 103.0 (d, *J* = 21.7 Hz), 99.4 (d, *J* = 26.4 Hz), 63.8, 52.2, 43.3, 42.2, 40.9, 35.0, 23.0, 22.2. ^{19}F NMR (376 MHz, $CDCl_3$): δ -112.6.
HRMS: Calculated for $C_{15}H_{20}FNNaO$ (M+Na$^+$): 272.1421, found 272.1419.

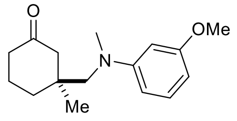

10e

(*S*)-3-(((3-methoxyphenyl)(methyl)amino)methyl)-3-methylcyclohexan-1-one 10e

Prepared according to general procedure using *N*-((dimethyl(phenyl)silyl)methyl)-3-methoxy-*N*-methylaniline **9e** (0.20 mmol, 57.1 mg), 3-methyl-2-cyclohexenone (0.1 mmol, 11.0 mg), the aminocatalyst (*R*,*R*)-**3e** (0.02 mmol, 13.3 mg), benzoic acid (0.04 mmol, 4.9 mg), and 0.2 mL of CH_3CN as the solvent. Time of irradiation: 72 hours. The crude mixture was purified by flash column chromatography (hexane/ethyl acetate: gradient from 10:1 to 4:1) to afford the product as a colorless oil (18.6 mg, 71% yield, 83% ee). The enantiomeric excess was determined to be 83% by HPLC analysis on a Daicel Chiralpak IC-3: 85/15 hexane/IPA, flow rate 1.0 mL/min, λ = 254 nm: τ_{Major} = 22.9 min, τ_{Minor} = 15.0 min. $[\alpha]_D^{25}$ = +27.6 (c = 0.11, $CHCl_3$, 83% ee).

<u>^{1}H NMR</u> (500 MHz, $CDCl_3$): δ 7.12 (t, *J* = 8.2 Hz, 1H), 6.39 (dd, *J* = 8.4, 2.5 Hz, 1H), 6.34-6.23 (m, 2H), 3.79 (s, 3H), 3.21 (d, *J* = 2.3 Hz, 2H), 3.00 (s, 3H), 2.44 (d, *J* = 13.2 Hz, 1H), 2.34-2.26 (m, 1H), 2.27-2.18 (m, 1H), 2.14 (d, *J* = 13.2 Hz, 1H), 2.04-1.92 (m, 1H), 1.90-1.75 (m, 2H), 1.67-1.52 (m, 1H), 0.96 (s, 3H).

<u>^{13}C NMR</u> (126 MHz, $CDCl_3$): δ 211.8, 160.7, 152.0, 129.8, 105.6, 101.1, 99.2, 63.9, 55.3, 52.3, 43.3, 42.2, 41.0, 34.9, 23.0, 22.3.

<u>HRMS:</u> Calculated for $C_{16}H_{23}NNaO_2$ (M+Na$^+$): 284.1621, found 284.1615.

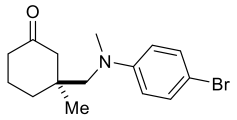

10f

(*S*)-3-(((4-bromophenyl)(methyl)amino)methyl)-3-methylcyclohexan-1-one 10f

Prepared according to general procedure using 4-bromo-*N*-((dimethyl(phenyl)silyl)methyl)-*N*-methylaniline **9f** (0.15 mmol, 50.1 mg), 3-methyl-2-cyclohexenone (0.1 mmol, 11.0 mg), the aminocatalyst (*R,R*)-**3e** (0.02 mmol, 13.3 mg), benzoic acid (0.04 mmol, 4.9 mg), and 0.2 mL of CH_3CN as the solvent. Time of irradiation: 48 hours. The crude mixture was purified by flash column chromatography (hexane/ethyl acetate: gradient from 10:1 to 4:1) to afford the product as a colorless oil (25.7 mg, 83% yield, 80% ee). The enantiomeric excess was determined to be 80% ee by HPLC analysis on a Daicel Chiralpak IC-3: 90/10 hexane/IPA, flow rate 1.0 mL/min, λ = 254 nm: τ_{Major} = 21.6 min, τ_{Minor} = 15.3 min. $[\alpha]_D^{25}$ = +31.4 (c = 0.10, $CHCl_3$, 80% ee). This compound is known, the spectroscopic data matched with previous report.[1]

<u>^{1}H NMR</u> (500 MHz, $CDCl_3$): δ 7.33-7.17 (m, 2H), 6.66-6.52 (m, 2H), 3.20 (d, *J* = 1.0 Hz, 2H), 2.98 (s, 3H), 2.40 (d, *J* = 13.2 Hz, 1H), 2.35-2.28 (m, 1H), 2.27-2.19 (m, 1H), 2.13 (dt, *J* = 13.2, 2.1 Hz, 1H), 2.03-1.93 (m, 1H), 1.92-1.72 (m, 2H), 1.66-1.56 (m, 1H), 0.95 (s, 3H).

<u>^{13}C NMR</u> (126 MHz, $CDCl_3$): δ 211.6, 149.5, 131.7, 113.9, 108.4, 63.8, 52.2, 43.4, 42.3, 40.9, 35.0, 23.1, 22.2.

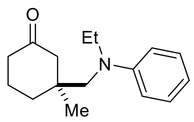

10g

(*S*)-3-((ethyl(phenyl)amino)methyl)-3-methylcyclohexan-1-one 10g

Prepared according to general procedure using *N*-((dimethyl(phenyl)silyl)methyl)-*N*-ethylaniline **9g** (0.15 mmol, 40.4 mg), 3-methyl-2-cyclohexenone (0.1 mmol, 11.0 mg), the aminocatalyst (*R,R*)-**3e** (0.02 mmol, 13.3 mg), benzoic acid (0.04 mmol, 4.9 mg), and 0.2 mL of CH_3CN as the solvent. Time of irradiation: 48 hours. The crude mixture was purified by flash column chromatography (hexane/ethyl acetate: gradient from 10:1 to 4:1) to afford the product as a colorless oil (14.0 mg, 57% yield, 79% ee). The enantiomeric excess was determined to be 79% by HPLC analysis on a Daicel Chiralpak IC-3: 90/10 hexane/IPA, flow rate 1.0 mL/min, λ = 254 nm: τ_{Major} = 12.5 min, τ_{Minor} = 10.4 min. $[\alpha]_D^{25}$ = +33.0 (c = 0.10, $CHCl_3$, 79% ee).

<u>^{1}H NMR</u> (500 MHz, $CDCl_3$): δ 7.24-7.14 (m, 2H), 6.80-6.76 (m, 2H), 6.68 (tt, *J* = 7.2, 1.0 Hz, 1H), 3.42 (q, *J* = 6.9 Hz, 2H), 3.16 (d, *J* = 1.7 Hz, 2H), 2.48-2.43 (m, 1H), 2.34-2.27 (m, 1H), 2.26-2.18 (m, 1H), 2.13 (dt, *J* = 13.2, 2.1 Hz, 1H), 2.01-1.92 (m, 1H), 1.90-1.77 (m, 2H), 1.60-1.55 (m, 1H), 1.09 (t, *J* = 6.9 Hz, 3H), 0.96 (s, 3H).

<u>^{13}C NMR</u> (126 MHz, $CDCl_3$): δ 212.0, 148.9, 129.0, 116.4, 113.5, 61.0, 52.1, 47.1, 43.0, 40.8, 34.7, 22.9, 22.2, 10.6. <u>HRMS:</u> Calculated for $C_{16}H_{24}NO$ (M+H$^+$): 246.1852, found 246.1851.

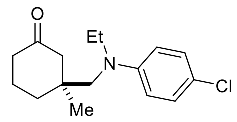

10h

(*S*)-3-(((4-chlorophenyl)(ethyl)amino)methyl)-3-methylcyclohexan-1-one 10h
Prepared according to general procedure using 4-chloro-*N*-((dimethyl(phenyl)silyl)methyl)-*N*-ethylaniline **9h** (0.15 mmol, 45.6 mg), 3-methyl-2-cyclohexenone (0.1 mmol, 11.0 mg), the aminocatalyst (*R*,*R*)-**3e** (0.02 mmol, 13.3 mg), benzoic acid (0.04 mmol, 4.9 mg), and 0.2 mL of CH_3CN as the solvent. Time of irradiation: 48 hours. The crude mixture was purified by flash column chromatography (hexane/ethyl acetate: gradient from 10:1 to 4:1) to afford the product as a colorless oil (22.2 mg, 79% yield, 81% ee). The enantiomeric excess was determined to be 81% by HPLC analysis on a Daicel Chiralpak IC-3: 90/10 hexane/IPA, flow rate 1.0 mL/min, λ = 254 nm: τ_{Major} = 15.3 min, τ_{Minor} = 12.2 min. $[\alpha]_D^{25}$ = +33.8 (c = 0.11, $CHCl_3$, 81% ee).
^{1}H NMR (500 MHz, $CDCl_3$): δ 7.17-7.06 (m, 2H), 6.72-6.65 (m, 2H), 3.39 (q, *J* = 7.0 Hz, 2H), 3.13 (d, *J* = 3.3 Hz, 2H), 2.47-2.38 (m, 1H), 2.35-2.27 (m, 1H), 2.26-2.18 (m, 1H), 2.10 (dt, *J* = 13.2, 2.1 Hz, 1H), 2.00-1.92 (m, 1H), 1.90-1.72 (m, 2H), 1.60-1.53 (m, 1H), 1.07 (t, *J* = 7.0 Hz, 3H), 0.94 (s, 3H).
^{13}C NMR (126 MHz, $CDCl_3$): δ 211.8, 147.6, 128.9, 121.3, 114.9, 61.2, 52.2, 47.5, 43.2, 40.9, 34.9, 23.1, 22.2, 10.7.
HRMS: Calculated for $C_{16}H_{23}ClNO$ (M+H$^+$): 280.1463, found 280.1472.

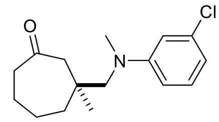

10i

(*S*)-3-(((3-chlorophenyl)(methyl)amino)methyl)-3-methylcycloheptan-1-one 10i
Prepared according to general procedure using *N*-((dimethyl(phenyl)silyl)methyl)-3-chloro-*N*-methylaniline **9c** (0.15 mmol, 43.5 mg), 3-methyl-2-cyclohexenone (0.1 mmol, 11.0 mg), the aminocatalyst (*R*,*R*)-**3e** (0.02 mmol, 13.3 mg), benzoic acid (0.04 mmol, 4.9 mg), and 0.2 mL of CH_3CN as the solvent. Time of irradiation: 48 hours. The crude mixture was purified by flash column chromatography (hexane/ethyl acetate: gradient from 10:1 to 4:1) to afford the product as a colorless oil (22.1 mg, 79% yield, 93% ee). The enantiomeric excess was determined to be 93% by HPLC analysis on a Daicel Chiralpak IC-3: 90/10 hexane/IPA, flow rate 1.0 mL/min, λ = 254 nm: τ_{Major} = 12.95 min, τ_{Minor} = 11.14 min. $[\alpha]_D^{25}$ = +26.5 (c = 0.12, $CHCl_3$, 93% ee).
^{1}H NMR (500 MHz, $CDCl_3$): δ 7.10 (t, *J* = 8.2 Hz, 1H), 6.73-6.70 (m, 1H), 6.67-6.60 (m, 2H), 3.31-3.10 (m, 2H), 3.00 (s, 3H), 2.76-2.71 (m, 1H), 2.55-2.45 (m, 1H), 2.42-2.30 (m, 2H), 1.94-1.80 (m, 2H), 1.76-1.59 (m, 3H), 1.56-1.45 (m, 1H), 0.96 (s, 3H).
^{13}C NMR (126 MHz, $CDCl_3$): δ 213.4, 151.6, 135.1, 130.0, 116.3, 112.3, 110.6, 64.5, 53.0, 44.1, 42.3, 41.2, 39.9, 24.4, 24.3, 23.5.
HRMS: Calculated for $C_{16}H_{22}ClNNaO$ (M+Na$^+$): 302.1282, found 302.1287.

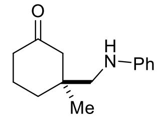

12

(*S*)-3-methyl-3-((phenylamino)methyl)cyclohexan-1-one 12
Prepared according to general procedure using phenylglycine **11** (0.20 mmol, 30.4 mg), 3-methyl-2-cyclohexenone (0.1 mmol, 11.0 mg), the aminocatalyst (*R*,*R*)-**3e** (0.02 mmol, 13.3 mg), benzoic acid (0.04 mmol, 4.9 mg), and 0.2 mL of CH_3CN as the solvent. Time of irradiation: 48 hours. The crude mixture was purified by flash column chromatography (hexane/ethyl acetate: gradient from 10:1 to 4:1) to afford the product as a yellow oil. (13.3 mg, 61% yield, 47% ee). The enantiomeric excess was determined to be 47% by HPLC analysis on a Daicel Chiralpak IC-3: 90/10 hexane/IPA, flow rate 1.0 mL/min, λ = 254 nm: τ_{Major} = 15.3 min, τ_{Minor} = 21.6 min. $[\alpha]_D^{25}$ = -10.6 (c = 0.14, $CHCl_3$, 47% ee). Absolute configuration determined in comparison to compound **2a**.
This compound is known, the spectroscopic data matched with previous report.[8]
^{1}H NMR (300 MHz, $CDCl_3$): δ 7.22-7.10 (m, 2H), 6.76-6.59 (m, 3H), 3.72 (s, br, 1H), 3.02 (d, *J* = 2.5 Hz, 2H), 2.47-2.25 (m, 3H), 2.20-2.10 (m, 1H), 1.99-1.72 (m, 3H), 1.69-1.52 (m, 1H), 1.02 (s, 3H).
^{13}C NMR (126 MHz, $CDCl_3$): δ 211.6, 148.6, 129.4, 117.7, 113.0, 54.3, 51.7, 41.0, 40.5, 34.1, 23.6, 22.1.

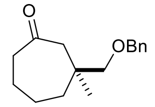

14

(*S*)-3-methyl-3-((phenylamino)methyl)cycloheptan-1-one 14

Prepared according to general procedure using diethyl 4-((benzyloxy)methyl)-2,6-dimethyl-1,4-dihydropyridine-3,5-dicarboxylate **13** (0.30 mmol, 112.0 mg), 3-methylcyclohept-2-en-1-one (0.1 mmol, 12.4 mg), the aminocatalyst (*R*,*R*)-**3d** (0.02 mmol, 11.0 mg), benzoic acid (0.08 mmol, 9.8 mg), and 0.2 mL of CH_3CN as the solvent. Time of irradiation: 4 days. The crude mixture was purified by flash column chromatography (hexane/ethyl acetate: gradient from 10:1 to 4:1) to afford the product as a colorless oil. (10.3 mg, 42% yield, 43% ee). The enantiomeric excess was determined to be 43% by HPLC analysis on a Daicel Chiralpak IC-3: 90/10 hexane/IPA, flow rate 1.0 mL/min, λ = 215 nm: τ_{Major} = 10.56 min, τ_{Minor} = 10.02 min. $[\alpha]_D^{25}$ = -10.0 (c = 0.11, $CHCl_3$, 43% ee). Absolute configuration determined in comparison to compound **2a**.

^{1}H NMR (300 MHz, $CDCl_3$): δ 7.38-7.22 (m, 5H), 4.49 (s, 2H), 3.16 (d, *J* = 1.3 Hz, 2H), 2.76-2.68 (m, 1H), 2.56-2.23 (m, 3H), 1.85-1.68 (m, 4H), 1.55-1.50 (m, 2H), 0.93 (s, 3H).

^{13}C NMR (126 MHz, $CDCl_3$): δ 214.2, 138.7, 128.5, 127.6, 127.6, 80.1, 73.4, 52.3, 44.2, 39.7, 37.1, 29.9, 24.6, 24.3, 23.1.

HRMS: Calculated for $C_{16}H_{22}NaO_2$ ($M+Na^+$): 269.1512, found 269.1520.

Supplementary References

1 Murphy, J. J., Bastida, D., Paria, S., Fagnoni, M. & Melchiorre, P. Asymmetric catalytic formation of quaternary carbons by iminium ion trapping of radicals. *Nature* **532**, 218-222 (2016).

2 Barer, T. E., Walker, S. D., Martinelli, J. R. & Buchwald, S. L. Catalysts for Suzuki−Miyaura coupling processes: scope and studies of the effect of ligand structure. *J. Am. Chem. Soc.* **127**, 4685-4696 (2005).

3 Hörmann, F. M., Chung, T. S., Rodriguez, E., Jakob, M. & Bach, T. Evidence for triplet sensitization in the visible-light-induced [2+2] photocycloaddition of eniminium ions. *Angew. Chem., Int. Ed.* **57**, 827-831 (2018).

4 Kehrli, S., Martin, D., Rix, D., Mauduit, M. & Alexakis, A. Formation of quaternary chiral centers by N-heterocyclic carbine-Cu-catalyzed asymmetric conjugate addition reactions with Grignard reagents on trisubstituted cyclic enones. *Chem. Eur. J.* **16**, 9890-9904 (2010).

5 Mueller, D. & Alexakis, A. Formation of quaternary stereogenic centers by copper-catalyzed asymmetric conjugate addition reactions of alkenylaluminums to trisubstituted enones. *Chem. Eur. J.* **19**, 15226-15239 (2013).

6 Bahamonda, A., Murphy, J. J., Savarese, M., Brémond, É., Cavalli, A. & Melchiorre P. Studies on the enantioselective iminium ion trapping of radicals triggered by an electron-relay mechanism. *J. Am. Chem. Soc.* **139**, 4559-4567 (2017).

7 Verrier, C., Alandini, N., Pezzetta, C., Moliterno, M., Buzzetti, L., Hepburn, H. B., Vega-Peñaloza, Silvi, M. & Melchiorre, P. Direct stereoselective installation of alkyl fragments at the β-carbon of enals via excited iminium ion catalysis. *ACS Catal.* **8**, 1062-1066 (2018).

8 Millet, A., Lefebvre, Q. & Rueping, M. Visible-light photoredox-catalyzed Giese reaction: decarboxylative addition of amino acid derived α-amino radicals to electron-deficient olefins. *Chem. Eur. J.* **22**, 13464-13468 (2016).
